# Supplementary material for: Direct ring-strain loading for visible-light accelerated bioorthogonal ligation via diarylsydnone-dibenzo[b,f ][1,4,5]thiadiazepine photo-click reactions
Source: Commun Chem. 2020 Mar 4;3:29. doi: 10.1038/s42004-020-0273-6 (PMC9814081; doi:10.1038/s42004-020-0273-6)
Supplement: Supplementary file 2 — Supplementary Information [file 42004_2020_273_MOESM2_ESM.pdf]

Supplementary Information for

Direct ring-strain loading for visible-light accelerated bioorthogonal ligation via diarylsydnone-dibenzo[*b*,*f*][1,4,5]thiadiazepine photo-click reactions

Jingshuo Gao,[†] Qin Xiong,[†] Xueting Wu,[†] Jiajie Deng,[†] Xiaocui Zhang,[†] Xiaohu Zhao,[†] Pengchi Deng*[,‡] and Zhipeng Yu*[,†]

[†]*Key Laboratory of Green Chemistry and Technology of Ministry of Education, College of Chemistry and* [‡]*Analytical & Testing Center, Sichuan University, 29 Wangjiang Road, Chengdu 610064, P. R. China*

E-mail: pcdeng@yahoo.com *and* zhipengy@scu.edu.cn

Contents

Supplementary Methods……………………………………………………………………......4

Determination of the photo-quantum yields of the photoconversion of **1d** and **1g**……….……..23

Photo-activated cycloaddition of DASyd **1d**/**1g** with a range of DBDZs in ACN/H_2O (1:1) with irradiation of the 311nm, 405 nm or 311 + 405 nm light sources……………………………….28

Screening of DASyd reagent for photo-click reaction with dibenzo[*b*,*f*][1,4,5]thiadiazepine (**DBTD**) to isolate of the MAI product and compare the photo-conversion under different irradiation conditions and under diluted conditions…………………………………………..40

Photophysical properties and stability investigation of the MAI cycloadduct in water containing solution phase and its resistance test against nucleophile additions by several nucleophilic reagents and GSH……………………………………………………………………………..73

Monitoring the background thermo-cycloaddition reactions of **1d** or **1g** with (*Z*)-**DBTD** in dark at 25 ºC by HPLC-MS analysis…………………………………………………………………74

The experiment apparatus and procedure for the *PSS* measurement with 405 nm laser continuous illumination recorded by ^{1}H NMR *in-situ*…………………………………………………….92

Photo-switching kinetic and photo-antifatigue studies of the **DBTD** under irradiation of 405 nm laser……………………………………………………………………………………………94

Determination of photo-switching quantum yield of the **DBTD** under irradiation of the 405 nm laser…………………………………………………………………………………………..101

Demonstration of the spatiotemporally controlled isomerization of the **DBTD** at low temperature…………………………………………………………………………………..105

Kinetic study of photo-induced 1,3-dipolar cycloaddition between the NI intermediate from tetrazole-1d (**TAZ-1d**) or DASyd **1d** versus methacrylamide (MAA)………………………...106

The competition reaction between the **DBTD** vs. MAA toward **1d** via NMR *in-situ* recording to demonstrate the acceleration through 405 nm illumination…………………………………….109

The competition reaction between the **DBTD** vs. **TCO** toward **1d** via NMR *in-situ* recording……………………………………………………………………………………..112

Chemical modification of proteins by **DBTD-NHS**…………………………………………119

Photo-activated cycloaddition reaction of Lyso-DBTD with DASyd **1d** resolved by HPLC MS…………………………………………………………………………………………....121

Specificity study of photo-activated DASyd **1d** toward Lysozyme (Lyso) resolved by LC-MS……………………………………………………………………………………123
The LC-MS/MS analysis for DASyd **1d** photo-ligation toward DBTD-K residue on lysozyme…………………………………………………………………………..126
The LC-MS/MS spectra and analysis of the modified peptide covering the key residue-K51……………………………………………………………………………..…..126
Fluorescence changes after photo-click conjugation of **1g-Cy3** to the **DBTD**, and subsequent reduction with either GSH or TCEP……………………………………………………130
Chemical modification of Cetuximab (chimeric monoclonal antibody) and Panitumumab (fully human mAb) by **DBTD-NHS** and FITC……………………………………………………132
Spatiotemporally resolved fluorescence labeling of the live A549 or A431cells………………135
Cell viability of A549 and A431 cells after incubation with the **DBTD**……………….………138
Crystal data and structure refinement for **3e**…………………………..…………………..140
Crystal data and structure refinement for (*Z*)-**DBTD** …………..………………………………142
Supplementary Reference…………………………………………………………………144
^{1}H, ^{19}F and ^{13}C NMR Spectra……………………………………………………………..145

Supplementary Methods

General Information – Chemical Synthesis

Unless otherwise indicated, all solvents and starting materials were purchased from commercial sources and used directly without further purification. The C-H activation cross-coupling reactions were set up in glovebox and carried out under nitrogen atmosphere in Schlenk tubes. Anhydrous solvents, purchased from Acros Organics (DMF and THF), were used as received. Commercially available chemicals were obtained from Adamas, Acros Organics, Aldrich Chemical Co., Alfa Aesar and TCI and used as received unless otherwise stated. The ^{1}H, ^{13}C and ^{19}F NMR spectra were recorded on a Brüker Avance 400 or 600 or 800 spectrometer (^{1}H: 400 or 600 or 800 MHz, ^{13}C: 101 or 150 or 201 MHz, ^{19}F: 376 MHz). Chemical shifts (δ) for ^{1}H and ^{13}C NMR spectra are given in ppm relative to TMS. The residual solvent signals were used as references for ^{1}H and ^{13}C NMR spectra and the chemical shifts converted to the TMS scale ($CDCl_3$, 7.26 ppm for ^{1}H NMR and 77.16 ppm for ^{13}C NMR; CD_3OH 3.31 ppm for ^{1}H NMR and 49.05 ppm for ^{13}C NMR; DMSO-d_6, 2.50 ppm for ^{1}H NMR and 39.5 ppm for ^{13}C NMR); CF_3Ph, -62.72 ppm for ^{19}F NMR. Shifts multiplicity was reported as follows: s = singlet, d = doublet, t = triplet, q = quartet, m = multiplet, brs. = broad.

General Information – Spectra Acquisition

UV-Vis absorption spectra were recorded by using 1 cm quartz cuvettes on a Thermo NANODROP 2000C Spectrophotometer. Exact ESI mass spectra were recorded on a SHIMADZU LCMS-IT-TOF. LC-ESI-MS were obtained on a Thermo LTQ-XL mass spectrometer.

The cell imaging experiments were carried out on an Olympus IX83 live cell fluorescence microscope. The cells were stained with a commercially available NucBlue ™ Live Ready Probe ™ Reagent for cell nuclear fluorescent imaging and identification.

The kinetic data of cycloaddition reaction between MAA and DASyd **1d** were recorded in real-time by a HORIBA Fluoromax-4 Spectrofluorometer Detector, and a deuterium arc & halide

lamp was used as the light source (Purchased from Shanghai Wenyi Photoelectric Technology Co., Ltd. China). The absorbance data of photo-switching kinetic and photo-antifatigue performance were recorded on an in-house assembled instrument based on a fast-response modular spectrometer.

General Information – Light Sources

The photo-irradiation power density of various light sources in photo-chemical transformation experiments were measured by an optical power meter produced by Thorlabs: a 311 nm UV lamp (10.8 mW cm^{-2}), a 311 nm LED array (21.2 mW cm^{-2}), a 405 nm LED array (13.4 mW cm^{-2}), a solid-state 405 nm laser for in cuvette stimulation (250 mW cm^{-2}) and an optical fiber (1 mm diameter, quartz) guided 405 nm laser source for *in-situ* NMR study of the *PSS* (135 mW at the fiber output port).

Experimental Procedures.

General Procedure for Palladium(II)-XPhos complex catalyzed C-H activation cross-coupling for the Direct Arylation of N^3-Aryl-Sydnones.

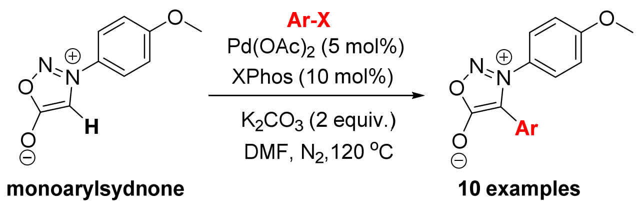

A flask equipped with a reflux condenser was charged with a mixture of monoarylsydnone (1.0 eq.), aryl halide (1.6 eq.), palladium(II) acetate (5 mol%), XPhos (10 mol%) and potassium carbonate (2-3 eq.) in DMF (0.1–0.5 M) under an atmosphere of nitrogen and heated at 80 – 120 °C for 3 hours. The reaction was allowed to cool to ambient temperature and water was added. The resulting mixture was extracted with EtOAc/hexanes (9/1) and the combined organic layers was dried over $MgSO_4$ and concentrated *in vacuo*. Flash silica chromatography (eluting solvent 20%-100% EtOAc in hexanes) afforded the desired DASyds. The compounds could be further purified by recrystallization from ethanol or EtOAc/hexanes solution (DASyds **1c**, **1d**, **1f**, **1g**, **1k** were synthesized according to our previous work).[1]

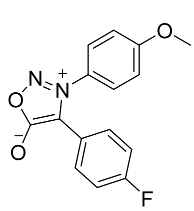

4-Fluorophenyl-3-(4-methoxyphenyl)sydnone (1a): monoarylsydnone (50.0 mg, 0.260 mmol) and 4-fluorobromobenzene (54.6 mg, 0.312 mmol) were subjected to the general conditions affording **1a** as a white solid (45.3 mg, 65%):^{1}H NMR (400 MHz, $CDCl_3$) δ 7.42 – 7.35 (m, 2H), 7.33 – 7.27 (m, 2H), 7.06 – 7.02 (m, 2H), 7.02 – 6.95 (m, 2H), 3.90 (s, 3H). ^{19}F NMR (376 MHz, $CDCl_3$) δ -111.07. ^{13}C NMR (101 MHz, $CDCl_3$) δ 167.1, 162.5 (d, J = 251.5 Hz), 162.2, 129.3 (d, J = 9.1 Hz), 127.1, 126.2, 120.8, 115.9 (d, J = 22.2 Hz), 115.3, 107.0, 55.8. HRMS (ESI) calcd. for $C_{15}H_{12}FN_2O_3^+$ 287.0826 [M+H$^+$], found 287.0825.

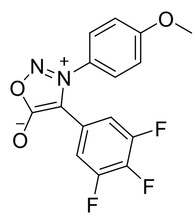

3,4,5-Trifluorophenyl-3-(4-methoxyphenyl)sydnone (1b): monoarylsydnone (50 mg, 0.260 mmol) and 3,4,5-trifluorobromobenzene (65.8 mg, 0.312 mmol) were subjected to the general conditions affording **1b** as a white solid (43.6 mg, 52%): ^{1}H NMR (400 MHz, $CDCl_3$) δ 7.38 – 7.31 (m, 2H), 7.07 – 7.00 (m, 2H), 6.97 – 6.89 (m, 2H), 3.86 (s, 3H). ^{19}F NMR (376 MHz, $CDCl_3$) δ -132.36, -158.59. ^{13}C NMR (101 MHz, $CDCl_3$) δ 166.4, 162.6, 151.3 (qd, J = 254.5 Hz), 139.4 (m, J = 257.6 Hz), 126.4, 126.2, 120.8 (m, J = 15.2 Hz), 115.6, 111.0 (dd, J = 17.2 Hz), 104.9, 55.9. HRMS (ESI) calcd. for $C_{15}H_{10}F_3N_2O_3^+$ 323.0638 [M+H$^+$], found 323.0635.

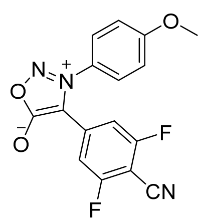

3,5-Difluoro-4-(nitrile)phenyl-3-(4-methoxyphenyl)sydnone (1h): monoarylsydnone (50.0 mg, 0.260 mmol) and 3,5-difluoro-4-nitrile-bromobenzene (68.0 mg, 0.312 mmol) were subjected to the general conditions affording **1h** as a white solid (60.0 mg, 70%): ^{1}H NMR (400 MHz, $CDCl_3$) δ 7.39 – 7.33 (m, 2H), 7.11 – 7.06 (m, 2H), 7.02 – 6.97 (m, 2H), 3.89 (s, 3H). ^{19}F NMR (376 MHz, $CDCl_3$) δ -102.03. ^{13}C NMR (101 MHz, $CDCl_3$) δ 165.7, 163.05, 163.01 (dd, J = 261.6 Hz), 132.3 (t, J = 11.1 Hz), 126.2, 115.9, 108.8 (dd, J = 23.2 Hz), 108.8, 104.1, 91.3, 91.1, 56.0. HRMS (ESI) calcd. for $C_{16}H_{10}F_2N_3O_3^+$ 330.0685 [M+H$^+$], found 330.0681.

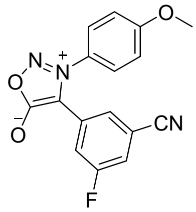

3-Fluoro-5-(nitrile)phenyl-3-(4-methoxyphenyl)sydnone (1i): monoarylsydnone **1a** (200 mg, 1.04 mmol) and 3-bromo-5-fluorobenzonitrile (250 mg, 1.25 mmol) were subjected to the general conditions affording **1i** as a white solid (181.7 mg, 56%): ^{1}H NMR (400 MHz, $CDCl_3$) δ 7.44 – 7.40 (m, 2H), 7.39 – 7.38 (m, 1H), 7.37 – 7.33 (m, 1H), 7.26 – 7.21 (m, 1H), 7.15 – 7.09 (m, 2H), 3.94 (s, 3H). ^{19}F NMR (376 MHz, $CDCl_3$) δ -107.46. ^{13}C NMR (101 MHz, $CDCl_3$) δ 166.2, 162.8, 162.1 (d, J = 251.5 Hz), 128.6 (d, J = 9.1 Hz), 126.3, 126.1, 125.6 (d, J = 3.0 Hz), 118.0 (d, J = 25.3 Hz), 117.9 (d, J = 24.2 Hz), 116.8 (d, J = 3.0 Hz), 115.8, 114.5 (d, J = 10.1 Hz),

104.4 (d, *J* = 3.0 Hz), 55.93. HRMS (ESI) calcd. for $C_{16}H_{11}FN_3O_3^+$ 312.0779 [M+H$^+$], found 312.0777.

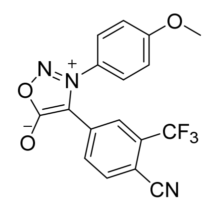

3-(Trifluoromethyl)-4-nitrilephenyl-3-(4-methoxyphenyl)sydnone (1j): monoarylsydnone **1a** (200 mg, 1.042 mmol) and 4-bromo-2-trifluoromethyl-benzonitrile (312.5 mg, 1.25 mmol) were subjected to the general conditions affording **1j** as a white solid (239.4 mg, 64%): ^{1}H NMR (400 MHz, $CDCl_3$) δ 7.75 – 7.71 (m, 2H), 7.65 (dd, *J* = 8.3, 1.8 Hz, 1H), 7.45 – 7.40 (m, 2H), 7.15 – 7.10 (m, 2H), 3.94 (s, 3H). ^{19}F NMR (376 MHz, $CDCl_3$) δ -62.44. ^{13}C NMR (101 MHz, $CDCl_3$) δ 166.0, 163.0, 134.9, 133.2 (q, *J* = 33.3 Hz), 129.8, 128.7, 126.4, 126.2, 123.6 (q, *J* = 5.05 Hz), 123.2, 115.9, 115.0, 108.5, 104.7, 56.0. HRMS (ESI) calcd. for $C_{17}H_{11}F_3N_3O_3^+$ 362.0747 [M+H$^+$], found 362.0743.

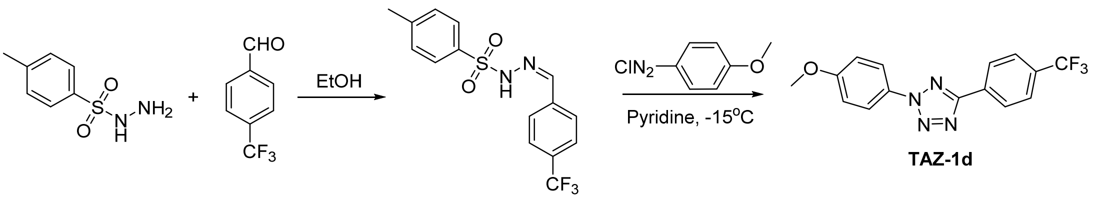

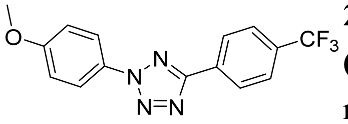

2-(4-Methoxyphenyl)-5-(4-(trifluoromethyl)phenyl)-2*H*-tetrazole (TAZ-1d): A 3 mL solution of the *p*-toluenesulfonylhydrazide (200 mg, 1.08 mmol) was added the corresponding aldehyde (1 equiv.) and Then anhydrous magnesium sulfate 500 mg was added and allowed to stand at room temperature for 8 hours. The suspension was filtered, washed with ethyl acetate, and the filtrate was evaporated in vacuo. The residue was used for the next step without purification. Then, the resulting hydrazone (1 equiv.) was dissolved in a 3 mL pyridine solution and cooling to 258 K. The *p*-methoxyaniline (1.2 equiv.) was dissolved in a 2 mL ethanol solution and placed at 258 K, acidified with concentrated hydrochloric acid (3 mL), then added with 1 mL aqueous solution of sodium nitrite (1.2 equiv.) and shaken evenly to form the diazonium salt. The mixture was added dropwise to the pre-cooling pyridine solution and then left at room temperature stirred 8 hours. The solid was filtered and washed with ethanol to yield a white solid (254.6 mg, 74%). ^{1}H NMR (400 MHz, $CDCl_3$) δ 8.37 (d, *J* = 8.1 Hz, 2H), 8.16-8.07 (m, 2H), 7.79 (d, *J* = 8.2 Hz, 2H), 7.12-7.03 (m, 2H), 3.91 (s, 3H). ^{19}F NMR (376 MHz, $CDCl_3$) δ -62.85. ^{13}C NMR (101 MHz, $CDCl_3$) δ 163.9, 160.8, 132.3 (q, *J* = 33.3 Hz), 130.8, 130.4, 127.4, 126.1 (q, *J* = 4.0 Hz), 124.0 (q, *J*

=273.7 Hz), 121.6, 114.9, 55.8. HRMS (ESI) calcd. for $C_{15}H_{12}F_3N_4O^+$ [M+H$^+$], 321.0958; found 321.1694.

The synthetic method of the DBDZs (2a-2c, DBTD):[2]

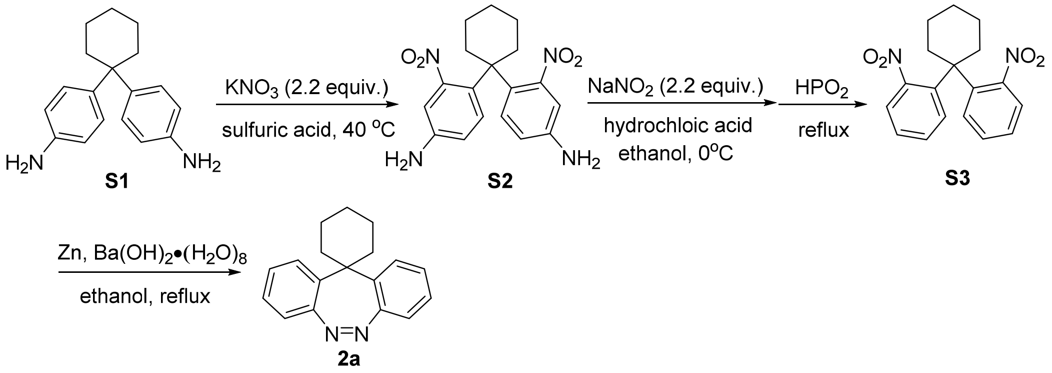

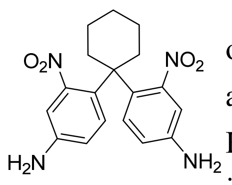

4,4'-(Cyclohexane-1,1-diyl)bis(3-nitroaniline) (S2): Preparing a suspension of the 4,4'-(cyclohexane-1,1-diyl)dianiline (**S1**) (1.00 g, 3.75 mmol) in sulfuric acid, the mixture was heated at 40 °C until the white sold dissolved, then the KNO_3 (702 mg, 8.26 mmol) dissolved in sulfuric acid was added dropwise into **S1,** stirring for 6 h, and the mixture was poured into ice and neutralized with 20% NaOH solution to pH = 8 at 0 °C. The precipitate was filtrated and washed with hexanes/EtOAc = 9/1, then the crude product (675.6 mg, 51%) was used for next step without further purification.

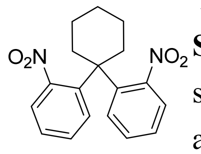

2,2'-(Cyclohexane-1,1-diyl)bis(nitrobenzene) (S3): To a stirring suspension of **S2** (500 mg, 3.75 mmol) and 200 μL 4 M HCl solution in ethanol at 0 °C and the suspension of $NaNO_2$ (702 mg, 8.26 mmol) in ethanol was added, stirring for 1 h, and the mixture was warmed to room temperature for another 2 h, then 2 mL hypophosphorous acid was added and the mixture was refluxed for 6 h. The crude product was purified by flash chromatography on silica gel (eluting with 25% EtOAc in hexanes) to yield a tan solid (126 mg, 34%). ^{1}H NMR (400 MHz, DMSO-*d₆*) δ 7.66 (dd, J = 8.1, 1.3 Hz, 2H), 7.63 – 7.57 (m, 2H), 7.51 – 7.43 (m, 4H), 2.25 (brs, 4H), 1.43 (brs, 6H). ^{13}C NMR (101 MHz, DMSO-d_6) δ 150.9, 135.4, 130.9, 130.5, 128.2, 124.4, 46.3, 36.1, 25.4, 22.3. HRMS (ESI) calcd. for $C_{18}H_{19}N_2O_4^+$ 327.1339 [M+H$^+$], found 327.1338.

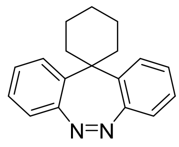

Spiro[cyclohexane-1,11'-dibenzo[*c,f*][1,2]diazepine] (2a): To a stirring suspension of **S3** (162 mg, 0.5 mmol), zinc powder (16.3 mg, 2.48 mmol) and $Ba(OH)_2•(H_2O)_8$ (276 mg, 0.875 mmol) in ethanol, the mixture was refluxed for 2.5 h. The reaction was allowed to cool to ambient temperature and the liquid was filtrated to remove residual and concentrated in vacuum. The crude product was purified by flash chromatography on silica gel (eluting with 10% EtOAc in hexanes) to yield a yellow solid (10 mg, 7%).^{1}H NMR (400 MHz, $CDCl_3$) δ 7.95 – 7.88 (m, 2H), 7.47 – 7.35 (m, 6H), 2.53 – 2.44 (m, 2H), 1.99 – 1.86 (m, 2H), 1.52 (brs, 2H), 1.47 – 1.38 (m, 2H), 1.15 – 1.04 (m, 2H). ^{13}C NMR (101 MHz, $CDCl_3$) δ 148.3, 139.7, 130.9, 130.0, 126.8, 124.4, 42.8, 32.9, 30.9, 26.5, 23.5, 23.22. HRMS (ESI) calcd. for $C_{18}H_{19}N_2^+$ 263.1541 [M+H$^+$], found 263.1543.

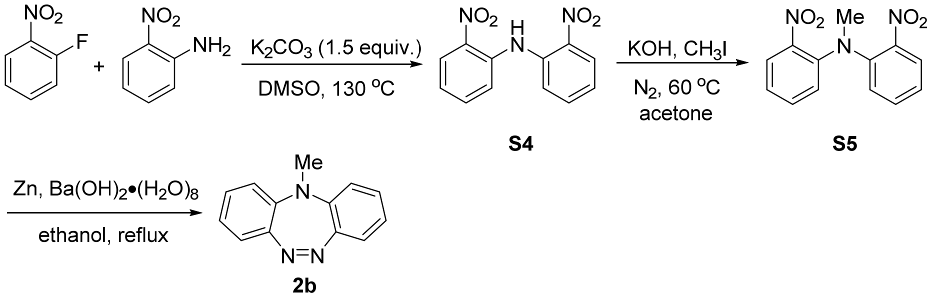

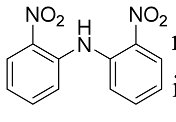

Bis(2-nitrophenyl)amine (S4): 2-nitroaniline (2.00 g, 14.2 mmol), 1-fluoro-2-nitrobenzene (1.96 g, 14.2 mmol), and K_2CO_3 (2.93 g, 21.3 mmol) were charged in a 500 mL round-bottom flask. DMSO (10 mL) was added to the mixture at room temperature, and the reaction mixture was heated at 130 ºC for 20 h. After the addition of water (20 mL), yellow precipitate was generated. After collection of the precipitates by filtration, the resulting product was washed by water (30 mL), and dried in vacuum oven, yielding an orange powder (3.40 g, 93%). ^{1}H NMR (400 MHz, $CDCl_3$) δ 10.94 (s, 1H), 8.13 (dd, J = 8.4, 1.5 Hz, 2H), 7.51 (dd, J = 8.4, 1.5 Hz, 2H), 7.49 – 7.42 (m, 2H), 7.05 – 6.99 (m, 2H).

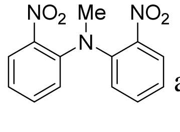

***N*-methyl-2-nitro-*N*-(2-nitrophenyl)aniline (S5):** To **S4** (500 mg, 1.93 mmol) and KOH (227.3 mg, 4.05 mmol) was added 5 mL of acetone at room temperature

under N_2 atmosphere. After stirring the reaction mixture at 60 ºC for 20 min, CH_3I (301.2 mg, 2.12 mmol) was slowly added to the reaction mixture, and the mixture was stirred for 52 h. After filtration, the filtrate was concentrated by rotary evaporator and H_2O (10 mL) was added to the residue. The desired product was extracted by CH_2Cl_2 (20 mL × 4). The combined organic layer was dried over $MgSO_4$ and concentrated under reduced pressure to give an orange solid (474 mg, 90%). ^{1}H NMR (400 MHz, $CDCl_3$) δ 7.79 (dd, J = 8.1, 1.6 Hz, 2H), 7.55 (td, J = 7.2, 1.6 Hz, 2H), 7.29 – 7.23 (m, 2H), 7.18 (td, J = 7.3, 1.3 Hz, 2H), 3.42 (s, 3H).

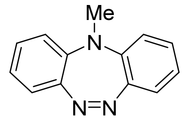

11-Methyl-11*H*-dibenzo[*c,f*][1,2,5]triazepine (2b): To a stirring suspension of **S5** (2.00 g, 7.32 mmol), zinc powder (2.85 g, 43.9 mmol) and $Ba(OH)_2$•$(H_2O)_8$ (4.04 g, 12.8 mmol) in ethanol (10 mL), the mixture refluxed for 2.5 h. The reaction was allowed to cool to ambient temperature and the liquid was filtrated to remove residual. The crude product was purified by flash chromatography on silica gel (eluting with 10% EtOAc in hexanes) to yield a yellow solid (183.6 mg, 12%).^{1}H NMR (400 MHz, $CDCl_3$) δ 7.49 (dd, J = 7.8, 1.7 Hz, 2H), 7.22 – 7.16 (m, 2H), 7.06 (td, J = 7.6, 1.2 Hz, 2H), 6.68 (dd, J = 8.2, 1.2 Hz, 2H), 3.02 (s, 3H). ^{13}C NMR (101 MHz, $CDCl_3$) δ 148.7, 145.2, 131.0, 130.7, 124.1, 117.4, 35.7. HRMS (ESI) calcd. for $C_{13}H_{12}N_3^+$ 210.1026 [M+H$^+$], found 210.1024.

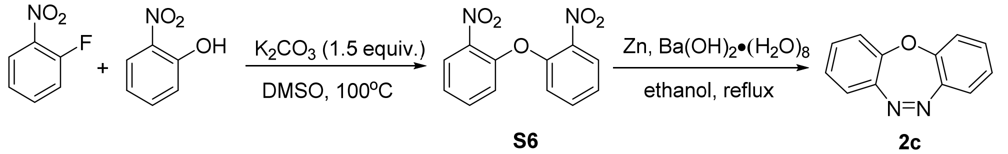

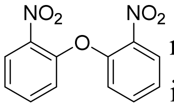

2,2'-Oxybis(nitrobenzene) (S6): 2-nitrophenol (2.00 g, 14.2 mmol), 1-fluoro-2-nitrobenzene (1.97 g, 14.2 mmol), and K_2CO_3 (2.93 g, 21.3 mmol) were charged in a 500 mL round-bottom flask. DMSO (10 mL) was added to the mixture at room temperature, and the reaction mixture was heated at 100 ºC for 16 h. After the addition of water (20 mL), yellow precipitates were generated. After collection of the precipitates by filtration, the resulting product was washed by water (30 mL), and dried at vacuum oven. **S4** was obtained as a yellow powder (2.50 g, 91%). All the resonances signal of ^{1}H and ^{13}C NMR spectra of the product were consistent with reported values.[3] ^{1}H NMR (400 MHz, DMSO-d_6) δ 8.16 (dd, J = 8.1, 1.6 Hz, 2H), 7.81 – 7.72 (m, 2H), 7.50 – 7.43 (m, 2H), 7.28 (dd, J = 8.3, 1.1 Hz, 2H).

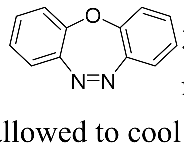

Dibenzo[*b*,*f*][1,4,5]oxadiazepine (2c): To a stirring suspension of **S6** (1.00 g, 3.84 mmol), zinc powder (1.25 g, 19.2 mmol) and $Ba(OH)_2•(H_2O)_8$ (2.11 g, 6.72 mmol) in ethanol (10 mL), the mixture refluxed for 2.5 h. The reaction was allowed to cool to ambient temperature and the liquid was filtrated to remove residual. The crude product was purified by flash chromatography on silica gel (eluting with 10% EtOAc in hexanes) to yield a yellow solid (156 mg, 21.4%). ^{1}H NMR (400 MHz, $CDCl_3$) δ 7.82 (dd, *J* = 7.7, 1.8 Hz, 2H), 7.39 (td, *J* = 7.7, 1.8 Hz, 2H), 7.32 (td, *J* = 7.6, 1.4 Hz, 2H), 7.06 (dd, *J* = 8.0, 1.4 Hz, 2H). ^{13}C NMR (101 MHz, $CDCl_3$) δ 152.5, 143.3, 132.6, 131.2, 125.8, 120.5. HRMS (ESI) calcd. for $C_{12}H_9N_2O^+$ 197.0709 [M+H$^+$], found 197,0708.

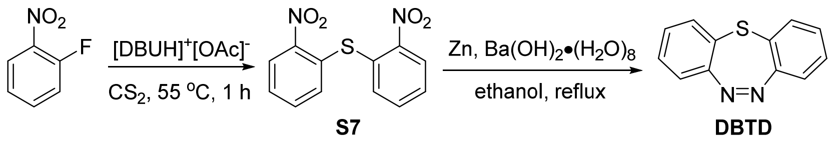

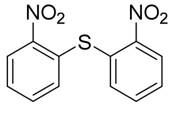

Bis(2-nitrophenyl)sulfane (S7): 1-fluoro-2-nitrobenzene (200 mg, 1.42 mmol), and CS_2 (54.0 mg, 0.710 mmol) were charged in a 100 mL round-bottom flask. The [DBUH]$^+$[OAc]$^-$ (3.00 g, 14.2 mmol) was added to the mixture at room temperature, and the reaction mixture was heated at 55 ºC for 1 h. After the addition of diethyl ether (50 mL × 5), filtrate was rotary evaporated to gain the crude product. The crude product was purified by flash chromatography on silica gel (eluting with 25% EtOAc in hexanes) to yield a yellow solid (195 mg, 99.7%). ^{1}H NMR (400 MHz, $CDCl_3$) δ 8.12 (dd, *J* = 8.0, 1.7 Hz, 2H), 7.54 (td, *J* = 7.6, 1.7 Hz, 2H), 7.48 (td, *J* = 7.7, 1.6 Hz, 2H), 7.30 (dd, *J* = 7.8, 1.6 Hz, 2H).

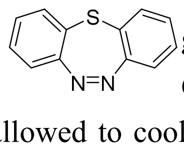

Dibenzo[*b*,*f*][1,4,5]thiadiazepine (DBTD): To a stirring suspension of **S7** (1.00 g, 3.60 mmol), zinc powder (1.84 g, 28.2 mmol) and $Ba(OH)_2•(H_2O)_8$ (1.99 g, 6.30 mmol) in ethanol (10 mL), the mixture refluxed for 2.5 h. The reaction was allowed to cool to ambient temperature and stirred for another 5 h in air. After the liquid was filtrated to remove residual, the crude product was then purified by flash chromatography on silica gel (eluting with 25% EtOAc in hexanes) to yield a yellow solid (231.5 mg, 30%). ^{1}H NMR (400 MHz, $CDCl_3$) δ 7.61 (dd, *J* = 7.9, 1.4 Hz, 2H), 7.44 (td, *J* = 7.6, 1.5 Hz, 2H), 7.34 (dd, *J* = 7.8, 1.5 Hz, 2H), 7.27 (td, *J* = 7.5, 1.4 Hz, 2H). ^{13}C NMR (101 MHz, $CDCl_3$) δ 151.8, 131.7,

131.1, 129.7, 129.2, 127.5. HRMS (ESI) calcd. for $C_{12}H_9N_2S^+$ 213.0481 [M+H$^+$], found 213.0479.

[DBUH]$^+$[OAc]$^-$ ionic liquid: To a stirring suspension of DBU (1.0 g, 6.6 mmol) at 0 ºC, acetic acid (1 eq.) was injected into DBU slowly, then the mixture was stirred at ambient temperature for 10 h, and dried in vacuum oven for 24 h to obtain a transparent yellow liquid as the desired ionic liquid.

Photo-induced cycloaddition of various DASyds with DBTD

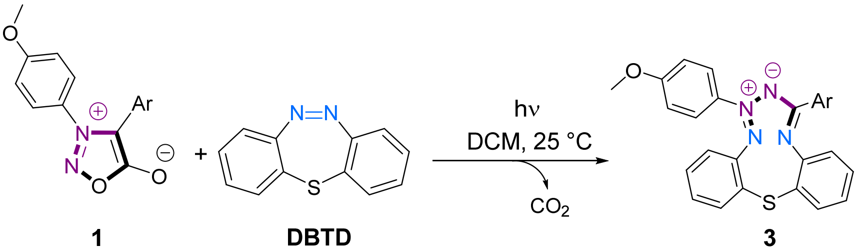

1a: Ar = *p*-FC_6H_4
1b: Ar = *m*-2F-*p*-FC_6H_2
1c: Ar = *m*-2F-*p*-$CF_3C_6H_2$
1d: Ar = *p*-$CF_3C_6H_4$
1e: Ar = *m*-$2CF_3C_6H_3$
1f: Ar = *p*-$COOEtC_6H_4$
1g: Ar = 6-COOMe-2-$C_{12}H_6$
1h: Ar = *m*-2F-*p*-CNC_6H_2
1i: Ar = 5-F-3-CNC_6H_3
1j: Ar = *m*-CF_3-*p*-CNC_6H_3

General Conditions: A stirred solution of DASyd (1 eq.) and **DBTD** (1 eq.) in DCM was irradiated with the 311 nm UV lamp and the 405 nm LED array in quartz round-bottom flask at room temperature for 2 h. The solvent was then evaporated, and the residue was purified by silica gel flash chromatography (eluting with 10% EtOAc in hexanes) to give the desired cycloaddition products.

3a: **1a** (33.7 mg, 0.12 mmol) and **DBTD** (25.0 mg, 0.12 mmol) in 120 mL DCM were subjected to the general conditions affording **3a** as a crimson solid (36.6 mg, 67%): ^{1}H NMR (400 MHz, $CDCl_3$) δ 7.84 – 7.78 (m, 2H), 7.77 – 7.71 (m, 2H), 7.47 (dd, J = 7.9, 1.3 Hz, 1H), 7.39 (dd, J = 7.8, 1.4 Hz, 1H), 7.00 (td, J = 7.6, 1.4 Hz, 1H), 6.97 – 6.90 (m, 2H), 6.87 (td, J = 7.7, 1.5 Hz,

1H), 6.85 – 6.79 (m, 3H), 6.76 (td, J = 7.7, 1.4 Hz, 1H), 6.54 (dd, J = 7.9, 1.4 Hz, 1H), 6.36 (dd, J = 8.0, 1.4 Hz, 1H), 3.85 (s, 3H). ^{19}F NMR (376 MHz, $CDCl_3$) δ -109.26. ^{13}C NMR (101 MHz, $CDCl_3$) δ 164.6 (d, J = 252.5 Hz), 163.4, 162.7, 158.1, 146.5, 145.7, 139.2, 130.7, 130.2, 129.7, 127.2, 126.5, 126.1, 125.5, 125.0, 123.6, 121.4, 119.1, 118.6, 114.9 (d, J = 22.2 Hz), 113.5, 55.7. HRMS (ESI) calcd. for $C_{26}H_{20}FN_4OS^+$ 455.1336 [M+H$^+$], found 455.1350.

3b: **1b** (38.6 mg, 0.12 mmol) and **DBTD** (25.0 mg, 0.12 mmol) in 120 mL DCM were subjected to the general conditions affording **3b** as a crimson solid (34.2 mg, 58%): ^{1}H NMR (800 MHz, $CDCl_3$) δ 7.79 (d, J = 8.6 Hz, 2H), 7.53 (d, J = 8.0 Hz, 1H), 7.50 – 7.44 (m, 2H), 7.42 (d, J = 7.8 Hz, 1H), 7.02 (t, J = 7.6 Hz, 1H), 6.96 (t, J = 7.7 Hz, 1H), 6.92 – 6.82 (m, 4H), 6.49 (d, J = 7.8 Hz, 1H), 6.40 (d, J = 7.5 Hz, 1H), 3.88 (s, 3H). ^{19}F NMR (376 MHz, $CDCl_3$) δ -134.52, -156.44. ^{13}C NMR (201 MHz, $CDCl_3$) δ 163.0, 156.3, 150.7 (dd, J = 249.2, 10.1 Hz), 146.11, 145.63, 141.45 (td, J = 255.3, 16.1 Hz), 138.9, 131.02, 131.00, 130.3, 127.5, 126.5, 125.7, 125.0, 123.9, 120.9, 118,7, 118.6, 118.3, 113.6, 111.7 (d, J = 20.1 Hz), 55.77. HRMS (ESI) calcd. for $C_{26}H_{18}F_3N_4OS^+$ 491.1148 [M+H$^+$], found 491.1144.

3c: **1c** (44.6 mg, 0.12 mmol) and **DBTD** (25.0 mg, 0.12 mmol) in 120 mL DCM were subjected to the general conditions affording **3c** as a crimson solid (41.6 mg, 64%): ^{1}H NMR (800 MHz, $CDCl_3$) δ 7.75 (d, J = 8.8 Hz, 2H), 7.52 (dd, J = 8.0, 1.2 Hz, 1H), 7.44 (d, J = 11.0 Hz, 2H), 7.41 (dd, J = 7.9, 1.3 Hz, 1H), 7.00 (td, J = 7.6, 1.3 Hz, 1H), 6.99 – 6.96 (m, 1H), 6.90 – 6.87 (m, 1H), 6.85 (dd, J = 7.7, 1.3 Hz, 1H), 6.85 – 6.82 (m, 2H), 6.44 (dd, J = 8.0, 1.3 Hz, 1H), 6.40 (d, J = 8.0 Hz, 1H), 3.86 (s, 3H). ^{19}F NMR (376 MHz, $CDCl_3$) δ -56.47, -110.85. HRMS (ESI) calcd. for $C_{27}H_{18}F_5N_4OS^+$ 541.1116 [M+H$^+$], found 541.1110.

3d: **1d** (40.3 mg, 0.12 mmol) and **DBTD** (25.0 mg, 0.12 mmol) in 120 mL DCM were subjected to the general conditions affording **3d** as a crimson solid (41.2 mg, 68%): ^{1}H NMR (400 MHz, $CDCl_3$) δ 7.88 (d, J = 8.1 Hz, 2H), 7.80 (d, J = 8.8 Hz, 2H), 7.55 – 7.46 (m, 3H), 7.40 (dd, J = 7.8, 1.4 Hz, 1H), 7.01 (td, J = 7.6, 1.5 Hz, 1H), 6.93 – 6.87 (m, 1H), 6.87 – 6.81 (m, 3H), 6.79 – 6.72 (m, 1H), 6.56 – 6.50 (m, 1H), 6.37 (d, J = 8.0 Hz, 1H), 3.85 (s, 3H). ^{19}F NMR (376 MHz, $CDCl_3$) δ -62.78. ^{13}C NMR (101 MHz, $CDCl_3$) δ 162.9, 158.0, 146.5, 145.8, 139.1, 138.1, 132.4 (q, J = 32.3 Hz), 130.9, 130.3, 127.8, 127.4, 126.6, 126.4, 125.5, 125.0, 124.8 (q, J = 4.04 Hz), 124.0 (q, J = 273.71 Hz), 123.8, 121.2, 119.0, 118.5, 113.6, 55.7. HRMS (ESI) calcd. for $C_{27}H_{20}F_3N_4OS^+$ 505.1304 [M+H$^+$], found 505.1312.

3e: 1e (48.5 mg, 0.12 mmol) and **DBTD** (25.0 mg, 0.12 mmol) in 120 mL DCM were subjected to the general conditions affording **3e** as a crimson solid (48.1 mg, 69%): ^{1}H NMR (800 MHz, $CDCl_3$) δ 8.19 (d, J = 1.7 Hz, 2H), 7.86 (s, 1H), 7.84 – 7.80 (m, 2H), 7.52 (dd, J = 7.9, 1.2 Hz, 1H), 7.42 (dd, J = 7.8, 1.3 Hz, 1H), 7.01 (td, J = 7.6, 1.4 Hz, 1H), 6.88 – 6.83 (m, 4H), 6.71 (td, J = 7.6, 1.3 Hz, 1H), 6.50 (dd, J = 7.9, 1.3 Hz, 1H), 6.32 (dd, J = 8.0, 1.3 Hz, 1H), 3.87 (s, 3H). ^{19}F NMR (376 MHz, $CDCl_3$) δ -62.72. ^{13}C NMR (201 MHz, $CDCl_3$) δ 163.1, 156.9, 146.0, 145.8, 138.8, 137.2, 131.3 (q, J = 34.2 Hz), 131.0, 130.4, 127.54, 127.49, 126.5, 126.4, 125.8, 125.1, 124.07, 124.05, 123.2 (q, J =271.4 Hz), 120.7, 118.3, 118.1, 113.7, 55.8. HRMS (ESI) calcd. for $C_{28}H_{19}F_6N_4OS^+$ 573.1178 [M+H$^+$], found 573.1186.

3f: 1f (40.8 mg, 0.12 mmol) and **DBTD** (25.0 mg, 0.12 mmol) in 120 mL DCM were subjected to the general conditions affording **3f** as a crimson solid (44.6 mg, 73%): ^{1}H NMR (800 MHz, $CDCl_3$) δ 7.93 – 7.89 (m, 2H), 7.85 – 7.79 (m, 4H), 7.47 (dd, *J* = 8.0, 1.2 Hz, 1H), 7.40 (dd, *J* = 7.8, 1.3 Hz, 1H), 7.00 (td, *J* = 7.7, 1.4 Hz, 1H), 6.88 – 6.81 (m, 4H), 6.74 – 6.71 (m, 1H), 6.53 (dd, *J* = 7.8, 1.3 Hz, 1H), 6.34 (d, *J* = 8.0 Hz, 1H), 4.38 (q, *J* = 7.1 Hz, 2H), 3.85 (s, 3H), 1.40 (t, *J* = 7.1 Hz, 3H). ^{13}C NMR (201 MHz, $CDCl_3$) δ 166.3, 162.8, 158.4, 146.4, 145.9, 139.2, 138.7, 132.3, 130.8, 130.3, 129.1, 127.4, 127.3, 126.7, 126.3, 125.4, 125.0, 123.7, 121.1, 118.9, 118.5, 113.6, 61.2, 55.7, 14.3. HRMS (ESI) calcd. for $C_{29}H_{25}N_4O_3S^+$ 509.1642 [M+H$^+$], found 509.1652.

3g: 1g (45.1 mg, 0.12 mmol) and **DBTD** (25.0 mg, 0.12 mmol) in 120 mL DCM were subjected to the general conditions affording 3g as a crimson solid (53 mg, 81%): ^{1}H NMR (800 MHz, CDCl3) δ 8.57 (s, 1H), 8.33 (s, 1H), 8.05 (d, J = 8.5 Hz, 1H), 7.92 – 7.78 (m, 5H), 7.49 (d, J = 7.9 Hz, 1H), 7.41 (d, J = 7.9 Hz, 1H), 7.03 (t, J = 7.5 Hz, 1H), 6.86 (d, J = 8.7 Hz, 3H), 6.80 (t, J = 7.7 Hz, 1H), 6.65 – 6.55 (m, 2H), 6.39 (brs, 1H), 3.98 (s, 3H), 3.86 (s, 3H). ^{13}C NMR (201 MHz, CDCl3) δ 167.1, 162.8, 158.8, 146.7, 145.8, 139.3, 134.9, 134.4, 133.8, 130.9, 130.66, 130.64, 130.3, 129.2, 128.9, 128.4, 127.6, 127.3, 126.6, 126.2, 125.5, 125.3, 125.1, 123.7, 121.4, 119.2, 118.6, 113.6, 55.8, 52.3. HRMS (ESI) calcd. for $C_{32}H_{25}N_4O_3S^+$ 545.1642 [M+H+], found 545.1664.

3h: 1h (39.5 mg, 0.12 mmol) and **DBTD** (25.0 mg, 0.12 mmol) in 120 mL DCM were subjected to the general conditions affording **3h** as a crimson solid (41.8 mg, 70%): ^{1}H NMR (800 MHz, $CDCl_3$) δ 7.82 – 7.68 (m, 2H), 7.56 – 7.44 (m, 3H), 7.41 (d, J = 7.8 Hz, 1H), 7.04 – 6.94 (m, 2H), 6.92 – 6.82 (m, 4H), 6.50 – 6.31 (m, 2H), 3.87 (s, 3H). ^{19}F NMR (376 MHz, $CDCl_3$) δ -103.89. ^{13}C NMR (201 MHz, $CDCl_3$) δ 163.3, 162.7 (dd, J = 259.3 Hz,), 155.8, 145.9, 145.7, 143.3,

138.6, 131.3, 130.5, 127.8, 126.8, 126.7, 125.9, 125,0, 124.3, 120.5, 118.3, 118.0, 113.8, 110.8, 109.2, 93.7, 55.8. HRMS (ESI) calcd. for $C_{27}H_{18}F_2N_5OS^+$ 498.1195 [M+H$^+$], found 498,1197.

3i: 1i (37.3 mg, 0.12 mmol) and **DBTD** (25.0 mg, 0.12 mmol) in 120 mL DCM were subjected to the general conditions affording **3i** as a crimson solid (50.1 mg, 87%): ^{1}H NMR (800 MHz, $CDCl_3$) δ 7.89 (d, 1H), 7.77 (d, J = 8.8 Hz, 2H), 7.75 – 7.71 (m, 1H), 7.52 (dd, J = 7.9, 1.2 Hz, 1H), 7.41 (dd, J = 7.8, 1.3 Hz, 1H), 7.35 – 7.31 (m, 1H), 7.00 (td, J = 7.7, 1.4 Hz, 1H), 6.93 (td, J = 7.7, 1.3 Hz, 1H), 6.88 – 6.79 (m, 4H), 6.46 (dd, J = 7.9, 1.3 Hz, 1H), 6.38 (d, J = 8.0 Hz, 1H), 3.86 (s, 3H). ^{19}F NMR (376 MHz, $CDCl_3$) δ -110.08. ^{13}C NMR (201 MHz, $CDCl_3$) δ 163.1, 161.9 (d, J = 260.4 Hz), 156.3, 146.1, 145.7, 138.9 (d, J = 8.0 Hz,), 138.7, 131.2, 130.4, 127.6, 127.2, 126.6, 126.5, 125.8, 125,0, 124.1, 120.8 (t, J = 12.1 Hz,), 120.8 (t, J = 12.1 Hz,), 119.2, 118.5, 118.1, 117.4, 113.7, 113.3 (d, J = 8.0 Hz,), 55.8. HRMS (ESI) calcd. for $C_{27}H_{19}FN_5OS^+$ 480.1289 [M+H$^+$], found 480.1291.

3j: 1j (43.3 mg, 0.12 mmol) and **DBTD** (25.0 mg, 0.12 mmol) in 120 mL DCM were subjected to the general conditions affording **3j** as a crimson solid (48.3 mg, 76%): ^{1}H NMR (800 MHz, Chloroform-*d*) δ 8.14 (s, 1H), 8.07 (dd, J = 8.0, 1.6 Hz, 1H), 7.79 (d, J = 8.7 Hz, 2H), 7.72 (d, J = 8.0 Hz, 1H), 7.51 (dd, J = 8.0, 1.2 Hz, 1H), 7.42 (dd, J = 7.9, 1.3 Hz, 1H), 7.01 (td, J = 7.6, 1.3 Hz, 1H), 6.93 – 6.88 (m, 1H), 6.88 – 6.82 (m, 3H), 6.76 (t, J = 7.6 Hz, 1H), 6.47 (dd, J = 7.9, 1.3 Hz, 1H), 6.33 (d, J = 8.0 Hz, 1H), 3.87 (s, 3H). ^{19}F NMR (376 MHz, $CDCl_3$) δ -61.78. ^{13}C NMR (201 MHz, $CDCl_3$) δ 163.2, 156.6, 146.0, 145.8, 139.7, 138.7, 134.4, 132.5 (q, J = 34.2 Hz,), 131.2, 130.6, 130.5, 127.6, 126.64, 126.59, 125.8, 125.6, 125.0, 124.2, 122.3 (q, J = 273.4 Hz,), 120.7, 118.4, 118.1, 115.4, 113.7, 111.3, 55.8. HRMS (ESI) calcd. for $C_{28}H_{19}F_3N_5OS^+$ 530.1257 [M+H$^+$], found 530.1252.

Synthesis of DBTD-Br

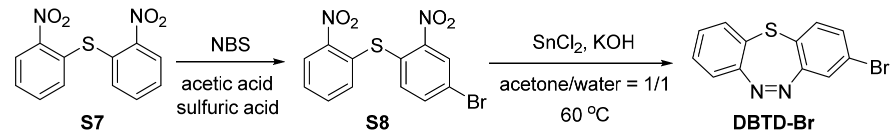

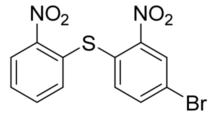

(4-Bromo-2-nitrophenyl)(2-nitrophenyl)sulfane (S8): S7 (1.00 g, 3.6 mmol) was dissolved in acetic acid (3 mL) then sulfuric acid (3 mL) was added, and the powder of NBS was added step-by-step (0.2 eq. each portion) until the raw material was converted fully. The crude product, **S8**, was then purified by flash chromatography on silica gel (eluting with 25% EtOAc in hexanes) to yield a yellow solid (511 mg, 40%). ^{1}H NMR (400 MHz, $CDCl_3$) δ 8.27 (d, J = 2.2 Hz, 1H), 8.12 (dd, J = 8.0, 1.6 Hz, 1H), 7.63 (dd, J = 8.5, 2.2 Hz, 1H), 7.61 – 7.50 (m, 2H), 7.34 (dd, J = 7.7, 1.5 Hz, 1H), 7.11 (d, J = 8.5 Hz, 1H). ^{13}C NMR (101 MHz, $CDCl_3$) δ 149.8, 149.2, 136.6, 134.4, 134.2, 133.7, 131.4, 130.4, 129.2, 128.4, 125.6, 121.6. HRMS (ESI) calcd. for $C_{12}H_6BrN_2O_4S^-$ 352.9237 [M-H$^+$], found 352.9236.

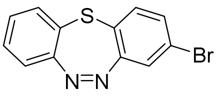

3-Bromodibenzo[*b,f*][1,4,5]thiadiazepine (DBTD-Br): To a stirring suspension of **S8** (100 mg, 0.28 mmol), $SnCl_2$ (187 mg, 0.99 mmol) and KOH (157 mg, 2.80 mmol) in acetone : water = 1 : 1 (3 mL), the mixture refluxed for 2.5 h. The reaction was allowed to cool to ambient temperature and stirred for another 5 h in air. After the liquid was filtrated to remove residual, the crude product was purified by flash chromatography on silica gel (eluting with 25% EtOAc in hexanes), after recrystallization, yielding a yellow solid (8.2 mg, 10%). ^{1}H NMR (400 MHz, $CDCl_3$) δ 7.61 (dd, J = 7.9, 1.3 Hz, 1H), 7.56 (dd, J = 8.4, 2.1 Hz, 1H), 7.51 (d, J = 2.0 Hz, 1H), 7.50 – 7.41 (m, 2H), 7.36 – 7.27 (m, 2H). ^{13}C NMR (101 MHz, $CDCl_3$) δ 151.7, 150.4, 134.2, 132.71, 132.4, 131.9, 130.3, 130.0, 129.6, 128.9, 127.7, 123.7. HRMS (ESI) calcd. for $C_{12}H_8BrN_2S^+$ 290.9586 [M+H$^+$], found 290.9587.

Synthesis of DBTD-NHS

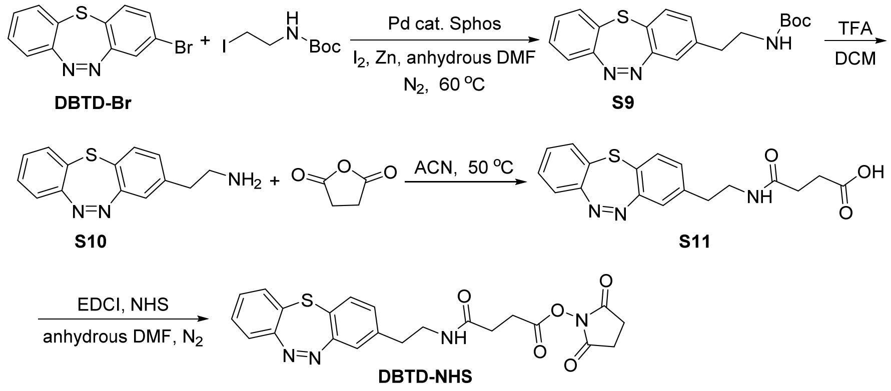

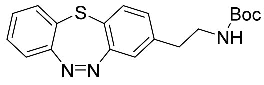

S9: To a stirring suspension of **DBTD-Br** (150 mg, 0.520 mmol), Pd(dba)$_2$ (14.9 mg, 0.025 mmol), Sphos (20.5 mg, 0.05 mmol) and dry DMF (1 mL) in 50 mL round-bottom flask under N_2 protection, a stirred Negishi reagent composed of *tert*-butyl (2-iodoethyl)carbamate (352.3 mg, 1.30 mmol), I_2 (97 mg, 0.36 mmol), zinc (246.9 mg, 3.90 mmol) and anhydrous DMF (3 mL × 3) in a 50 mL round-bottom flask for 10 min under N_2 atmosphere was added via the filtered injected into the prior flask. The reaction mixture was then heated at 60 ºC for 24 h. After added water (10 mL), the reaction mixture was extracted by EtOAc (30 mL × 3), the crude product was purified by flash chromatography on silica gel (eluting with 25% EtOAc in hexanes) to yield a yellow solid (95 mg, 52%). ^{1}H NMR (400 MHz, $CDCl_3$) δ 7.60 (dd, J = 7.9, 1.4 Hz, 1H), 7.55 (d, J = 8.0 Hz, 1H), 7.44 (td, J = 7.6, 1.6 Hz, 1H), 7.33 (dd, J = 7.8, 1.6 Hz, 1H), 7.28 (dd, J = 7.3, 1.5 Hz, 1H), 7.25 (d, J = 1.6 Hz, 1H), 7.17 (d, J = 1.8 Hz, 1H), 4.56 (s, 1H), 3.35 (q, J = 6.8 Hz, 2H), 2.78 (t, J = 7.1 Hz, 2H), 1.42 (s, 9H). ^{13}C NMR (101 MHz, $CDCl_3$) δ 155.8, 151.8, 150.4, 141.5, 131.9, 131.8, 131.1, 131.0, 129.73, 129.70, 129.2, 128.0, 127.7, 79.5, 41.4, 35.6, 28.4. HRMS (ESI) calcd. for $C_{19}H_{22}N_3O_2S^+$ 356.1427 [M+H$^+$], found 356.1424.

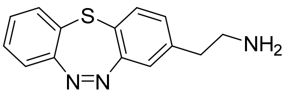

S10: To a stirring solution of **S9** (90 mg, 0.25 mmol) in DCM (3 mL) at 0 ºC, TFA (2 mL) was added, then the mixture reacted at room temperature for 3 h. After that, the residual TFA was neutralized by $NaHCO_3$ aqueous solution. The organic layer was extracted by DCM (12 mL × 3) and combined. And the crude product was purified by flash chromatography on silica gel (eluting with 20% methanol in DCM) to yield a yellow solid (53 mg, 82%). ^{1}H NMR (400 MHz, CD_3OD-d_4) δ 7.60 – 7.57 (m, 1H), 7.57 – 7.54 (m, 1H), 7.54 – 7.49 (m, 1H), 7.44 (dd, J = 8.1, 1.9 Hz, 1H), 7.40 – 7.32 (m, 3H), 3.17 (t, J = 7.6 Hz, 2H), 2.95 (t, J = 7.6 Hz, 2H). ^{13}C NMR (101 MHz, CD_3OD-d_4)

δ 151.6, 150.6, 139.4, 131.7, 131.6, 131.5, 130.7, 130.0, 129.8, 129.4, 127.5, 126.9, 39.9, 32.3. HRMS (ESI) calcd. for $C_{14}H_{14}N_3S^+$ 256.0903 [M+H$^+$], found 256.0899.

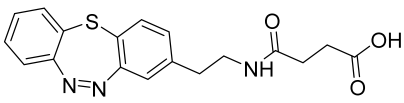

S11: To a stirring solution of **S10** (50 mg, 0.2 mmol) in ACN (3 mL), succinic anhydride (29.4 mg, 0.30 mmol) was added, after the mixture reacted at room temperature for 24 h, the crude product was purified by flash chromatography on silica gel (eluting with 10% methanol in DCM) to yield a yellow solid (68.2 mg, 98%). ^{1}H NMR (600 MHz, DMSO-d_6) δ 12.10 (s, 1H), 7.95 – 7.91 (m, 1H), 7.64 (dd, *J* = 7.9, 1.3 Hz, 1H), 7.60 – 7.54 (m, 2H), 7.44 (dd, *J* = 7.9, 1.4 Hz, 1H), 7.42 – 7.38 (m, 2H), 7.29 (d, *J* = 1.7 Hz, 1H), 3.26 (q, *J* = 6.7 Hz, 2H), 2.71 (t, *J* = 7.0 Hz, 2H), 2.39 (t, *J* = 7.0 Hz, 2H), 2.27 (t, *J* = 7.0 Hz, 2H). ^{13}C NMR (151 MHz, DMSO-d_6) δ 174.3, 174.1, 171.4, 151.7, 150.2, 143.0, 132.3, 132.2, 130.68, 130.65, 130.4, 130.3, 127.7, 127.5, 34.7, 30.5, 29.6, 29.2. HRMS (ESI) calcd. for $C_{18}H_{16}N_3O_3S^-$ 354.0918 [M-H$^+$], found 354.0917.

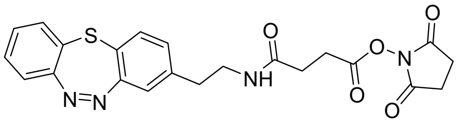

DBTD-NHS: To a stirring solution of **S11** (50 mg, 0.14 mmol) in anhydrous DMF (1 mL) under N_2, EDCI · HCl (28.3 mg, 0.15 mmol) and NHS (32.2 mg, 0.28 mmol) was added, after the mixture reacted at room temperature for 6 h, the crude product was extracted with EtOAc (2 mL × 3), then purified by flash chromatography on silica gel (eluting with 10% methanol in DCM) to yield a yellow solid (68.2 mg, 98%). HRMS (ESI) calcd. for $C_{23}H_{22}N_3O_5S^+$ 453.1227 [M+H$^+$], found 453.1223. ^{1}H NMR (400 MHz, $CDCl_3$) *δ* 7.60 (dd, *J* = 7.9, 1.4 Hz, 1H), 7.54 (d, *J* = 8.0 Hz, 1H), 7.47 – 7.41 (m, 1H), 7.33 (dd, *J* = 7.8, 1.6 Hz, 1H), 7.30 – 7.27 (m, 1H), 7.26 – 7.23 (m, 1H), 7.16 (d, *J* = 1.8 Hz, 1H), 5.74 (s, 1H), 3.50 (q, *J* = 6.7 Hz, 2H), 2.96 (t, *J* = 6.9 Hz, 2H), 2.84 (brs, 4H), 2.79 (t, *J* = 7.0 Hz, 2H), 2.54 (t, *J* = 7.0 Hz, 2H).^{13}C NMR (151 MHz, $CDCl_3$) *δ* 170.0, 169.0, 168.1, 151.9, 150.4, 141.2, 131.9, 131.8, 131.2, 131.0, 129.8, 129.7, 129.2, 128.0, 127.6, 40.5, 34.9, 31.0, 27.0, 25.6. HRMS (ESI) calcd. for $C_{22}H_{21}N_4O_5S^+$ 453.1227 [M+H$^+$], found 453.1226.

Synthesis of 1g-Cy3

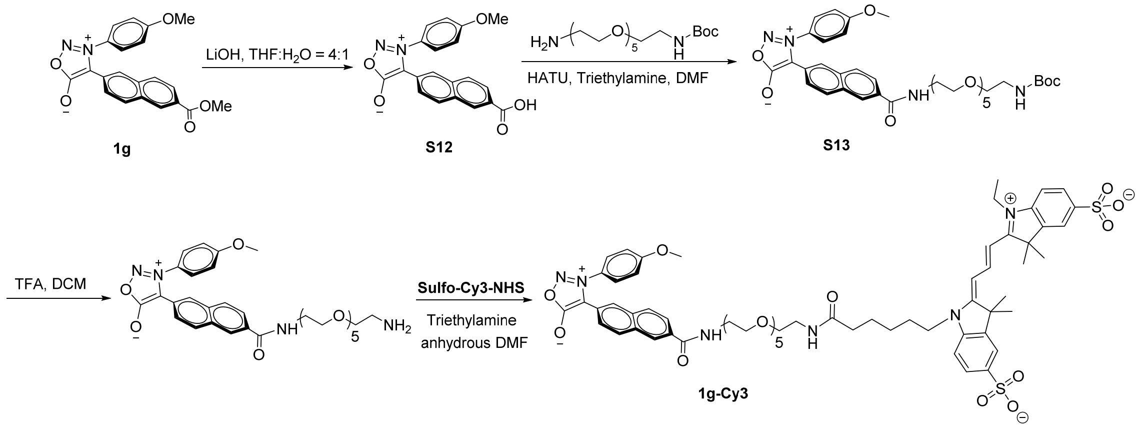

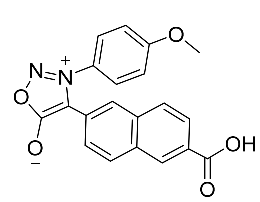

S12: To a stirring suspension of **1g** (120 mg, 0.32 mmol) in THF/H_2O = 4/1 (5 mL), LiOH (22.9 mg, 0.96 mmol) was added. The reaction mixture was stirred at room temperature overnight. Then the mixture was extracted with EtOAc (3 mL × 3) and concentrated under vacuum to give the crude product. The crude product was purified by flash chromatography on silica gel (eluting with 10% MeOH in DCM) to yield a white powder (118 mg, 95%). ^{1}H NMR (400 MHz, DMSO-*d*6) *δ* 8.48 (s, 1H), 8.06 – 7.98 (m, 2H), 7.94 (d, *J* = 8.7 Hz, 1H), 7.83 (d, *J* = 8.6 Hz, 1H), 7.70 – 7.63 (m, 2H), 7.18 (dd, *J* = 9.5, 2.8 Hz, 3H), 3.84 (s, 3H). ^{13}C NMR (101 MHz, DMSO-*d*6) *δ* 166.5, 161.8, 133.8, 131.6, 129.5, 129.4, 129.30, 129.26, 127.8, 127.1, 127.0, 126.5, 126.4, 124.6, 123.6, 115.3, 108.0, 55.9. HRMS (ESI) calcd. for $C_{20}H_{13}N_2O_5^-$ 361.0830 [M-H$^+$], found 361.0829.

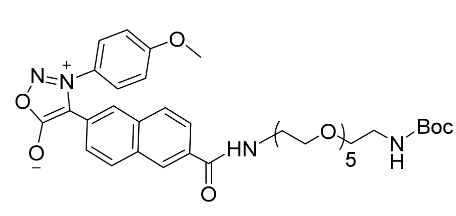

S13: To a stirring solution of **S12** (15 mg, 0.041 mmol) in anhydrous DMF (2 mL), HATU (18.9 mg, 0.050 mmol), *tert*-butyl (17-amino-3,6,9,12,15-pentaoxaheptadecyl)-carbamate (Boc-NH-PEG$_5$-$CH_2CH_2NH_2$, 18.9 mg, 0.050 mmol) and Et_3N (17.1 µL, 0.123 mmol) was added to react until the raw material was converted completely. Then the mixture was extracted by EtOAc (12 mL × 3) and the crude product was purified by flash chromatography on silica gel (eluting with 10% EtOH in DCM) to yield a white solid (29.4 mg, 98.0%). ^{1}H NMR (600 MHz, DMSO-*d*6) *δ* 8.73 – 8.66 (m, 1H), 8.39 (d, *J* = 1.7 Hz, 1H), 8.03 (d, *J* = 1.7 Hz, 1H), 7.97 – 7.89 (m, 3H), 7.71 – 7.66 (m, 2H), 7.24 (dd, *J* = 8.7, 1.8 Hz, 1H), 7.21 – 7.17 (m, 2H), 6.78 – 6.70 (m, 1H), 3.86 (s,

3H), 3.60 – 3.37 (m, 22H), 3.08 – 3.01 (m, 2H), 1.36 (s, 9H). ^{13}C NMR (151 MHz, DMSO) δ 166.9, 166.5, 162.8, 162.2, 156.0, 134.0, 133.0, 131.8, 129.5, 128.6, 127.7, 127.5, 127.4, 126.8, 125.5, 125.3, 124.2, 115.7, 108.3, 78.0, 70.24, 70.22, 70.19, 70.16, 70.10, 69.9, 69.6, 69.3, 56.3, 40.5, 38.7, 36.3, 31.2, 28.7, 14.4. HRMS (ESI) calcd. for $C_{37}H_{49}N_4O_{11}^+$ 725.3392 [M+H$^+$], found 725.3377.

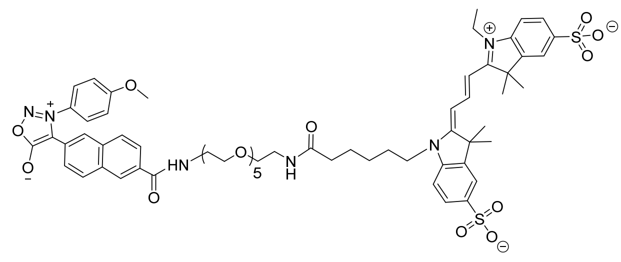

1g-Cy3: To a stirring suspension of **S13** (10 mg, 0.012 mmol) in DCM (2 mL), the TFA (1 mL) was added at 0 ºC, then the mixture reacted at room temperature for 1 h. After getting rid of the TFA by air flow, the residual TFA was neutralized by $NaHCO_3$ aqueous solution, the organic layer was extracted by DCM (5 mL × 3) and purified by flash chromatography on silica gel (eluting with 60% EtOH in DCM) to get a white solid crude primary amine product (8.02 mg, 93.0%). Then, the crude product (8.0 mg, 0.016 mmol) was dissolved in anhydrous DMF, the **Sulfo-Cy3-NHS** (9.28 mg, 0.0160 mmol) and TEA (10 μL) were added under N_2 atmosphere, after the reaction was reacted for 12 h at room temperature, the mixture was concentrated and purified by reversed phase HPLC via a Gemini C18 HPLC Column to yield a red solid (12.6 mg, 80.0%). ^1H NMR (600 MHz, D_2O) δ 8.14 – 8.04 (m, 1H), 7.94 (s, 1H), 7.76 – 7.63 (m, 4H), 7.51 (d, J = 8.3 Hz, 1H), 7.44 – 7.21 (m, 3H), 7.10 – 7.01 (m, 2H), 6.95 (s, 2H), 6.76 (s, 1H), 6.60 (s, 2H), 6.20 – 6.03 (m, 2H), 3.92 – 3.65 (m, 4H), 3.64 – 3.22 (m, 27H), 3.09 (s, 2H), 1.95 (s, 2H), 1.35 (s, 17H), 1.16 – 0.89 (m, 5H). HRMS (ESI) calcd. for $C_{63}H_{75}N_6O_{16}S_2^-$ 1235.4686 [M-H$^+$], found 1235.4702.

CCK-8 assays

The A549 or A431 cells were seeded in 96-well plates with a concentration of 6,000 cells per well. After 24 hours, the **DBTD** was added with final concentrations of 0, 7.5, 15, 30 and 60 μM in dark or irradiation with the 405 nm LED array for 30s, respectively. Then, the A549 or A431 cells were further cultured for 24 hours. The CCK-8 assay was then carried out. For each well, 10 μL CCK-8 solution was added. After 1.5 hours' incubation at 37 ºC, the absorbance at 450 nm was then measured by a plate reader to indicate the cell viability.

(a)

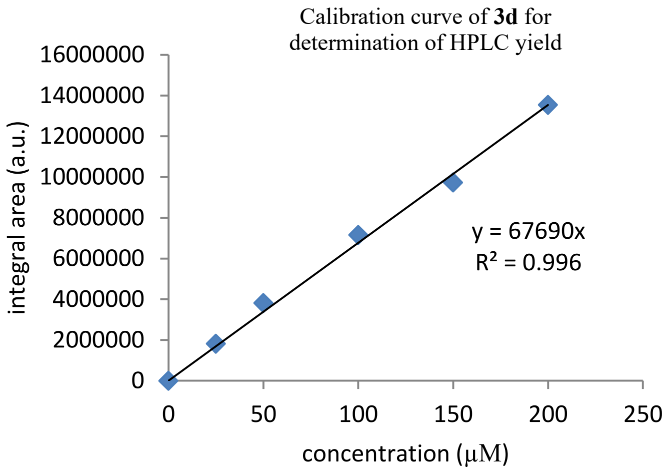
Calibration curve of **3d** for determination of HPLC yield
integral area (a.u.)
16000000
14000000
12000000
10000000
8000000
6000000
4000000
2000000
0
0
50
100
150
200
250
concentration (μM)
y = 67690x
R² = 0.996

(b)

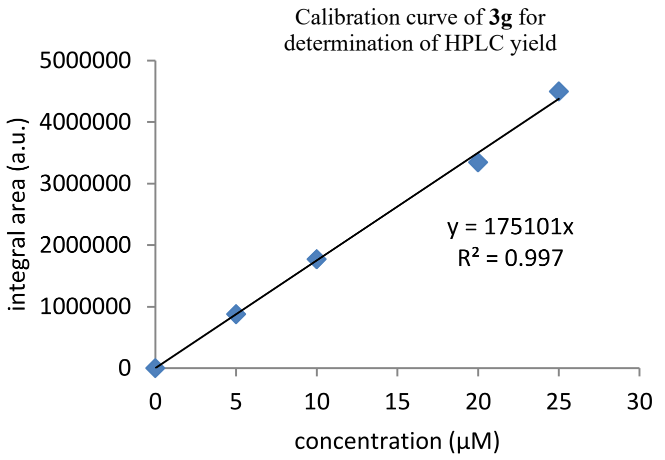
Calibration curve of **3g** for determination of HPLC yield
integral area (a.u.)
5000000
4000000
3000000
2000000
1000000
0
0
5
10
15
20
25
30
concentration (μM)
y = 175101x
R² = 0.997

(c)

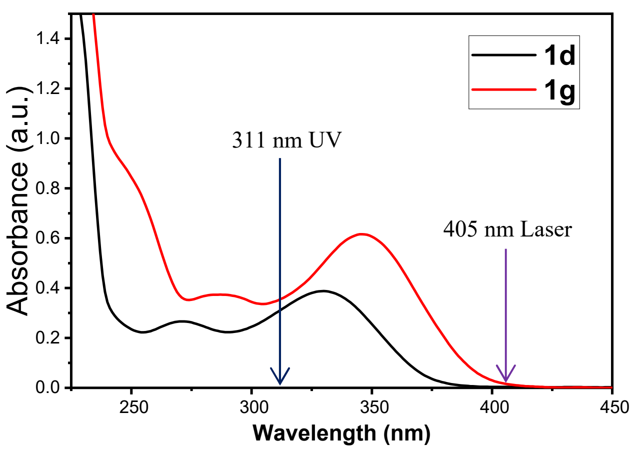
Absorbance (a.u.)
1.4
1.2
1.0
0.8
0.6
0.4
0.2
0.0
250
300
350
400
450
Wavelength (nm)
1d
1g
311 nm UV
405 nm Laser

Supplementary Figure 1. HPLC analyses for generation of the calibration curves of the MAI, **3d** or **3g**: the concentration of **3d** or **3g** as a function of the integral area of corresponding peak in HPLC traces was plotted by monitoring absorbance at 254 nm in solvent of ACN/H_2O (1:1, v/v). The linear calibration curves of (**a**) **3d** (monitored at 25 μM, 50 μM, 100 μM, 150 μM, 200 μM, respectively) and (**b**) **3g** (monitored at 5 μM, 10 μM, 20 μM, 25 μM, respectively) were fitted. All data sets were averaged out of at least three replicates. (**c**) The UV-vis spectra of **1d** and **1g** at 30 μM in ACN/H_2O (1:1, v/v) to illustrate their difference in photo-inducibility at 311 or 405 nm light.

Supplementary Note 1. Determination of photo-quantum yields for photoconversion of the 1d and 1g

The photolysis quantum yields of **1d** and **1g** were determined by using potassium ferrioxalate-based chemical actinometer.[4] In brief, a 250 μL fresh solution of 6 mM potassium ferrioxalate in 0.1 N H_2SO_4 aqueous solution was irradiated with the 311 nm UV lamp (single wavelength output after an optical filter) or the CW solid-state 405 nm laser in a quartz cuvette (0.2 cm × 1.0 cm optical path) for specified times before quenching by addition of 4.75 mL of NaOAc/HOAc buffer (pH = 4.3) and 5 mL of 0.1% 1,10-phenanthroline solution in water to develop the characteristic color at 510 nm. The mixture was stirred for 30 min before UV-Vis measurement. All the work was carried out in the dark and the samples were also protected from light with aluminum foil during handling. All the operation procedures were also the same for both the chemical actinometer and tested samples (direct measurement without developing process), including light sources, experimental setup, volume of the solution and the cuvette. The quantum yield for a test compound was calculated based on the following equations:

The incident monochromic photon flux I_0:

$$I_0 = \frac{\mathrm{d}\,[Act]}{\mathrm{d}\,t} \times [1/(1-10^{-Abs_c})]/\Phi_c = \frac{\mathrm{d}\,[DASyd]}{\mathrm{d}\,t} \times [1/(1-10^{-Abs_t})]/\Phi_t,$$ [5,6] **Supplementary Equation 1**

Because at the initial photo-conversion stage: $\frac{\mathrm{d}\,[Act]}{\mathrm{d}\,t} = \frac{\mathrm{d}\,Abs_{product}}{\mathrm{d}\,t}\left(\frac{1}{\varepsilon_{product}l}\right)$, therefore:

$$I_0 = (40\times k_c/\varepsilon_{510}l) \times [1/(1-10^{-Abs_c})]/\Phi_c = (k_t/\triangle\varepsilon_p l) \times [1/(1-10^{-Abs_t})]/\Phi_t,$$ **Supplementary Equation 2**

The subscript "*c*" represents to the parameters of the chemical actinometer

The subscript "*t*" represents to the parameters of the tested diarylsydnone, and "*p*" for the pyrazoline. Therefore:

$$\Phi_{reac.} = \Phi_t = [(1-10^{-\varepsilon_c c_c l})/(1-10^{-\varepsilon_t c_t l})] \times [k_t/(40 \times k_c)] \times (\varepsilon_{510}/\triangle\varepsilon_p) \times \Phi_c,$$ **Supplementary Equation 3**

where ε_c and ε_t were extinction coefficients of the standard chemical actinometer and test samples (**1d** and **1g** at 311 nm or **1g** at 405 nm), respectively. $l = 0.2$ cm;

k_t and k_c were slopes of linear fitting line of product formation in plots of absorbance changes versus time at the observing wavelength for the test compound and the standard chemical actinometer, respectively. The zeroth order photo-conversion rate (k_c and k_t) could be only applied at very low conversion (as low as possible) of the starting materials because the absorption of light by the products formed under such condition is minimal. Noteworthy, the addition of the buffer solution and the developer during the color readout of the actinometer conversion resulted in a 40-fold dilution, therefore the k_c need to be multiplied by 40;

c_c and c_t were concentrations of the standard actinometer and the test compound, respectively;

ε_{510}[7] and $\triangle\varepsilon_p$ were extinction coefficients of the Fe^{2+}-(1,10-phenanthroline)$_3$ complex at 510 nm for the actinometer and the difference in extinction coefficients of the pyrazoline products compared to the origin DASyd at monitoring wavelength, respectively.

The quantum yields of **1d** and **1g** in ACN/H_2O (1:1) with 311 nm light-induced transformation were determined. The quantum yield of **1g** in ACN/H_2O (1:1) with 405 nm light-induced transformation was determined.

(a)

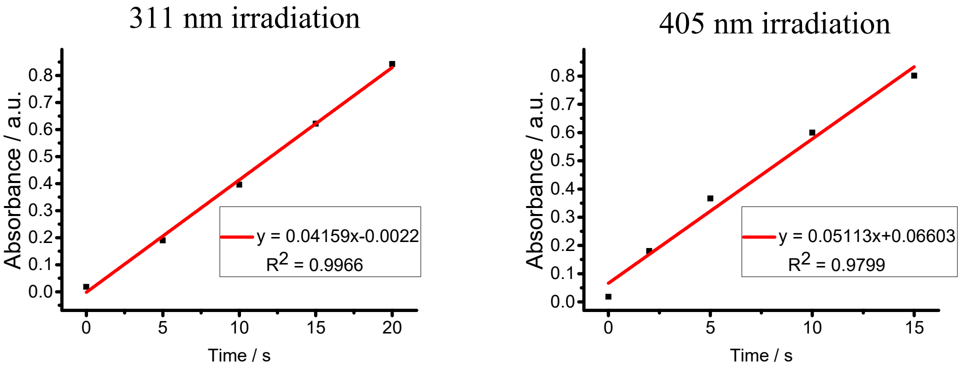

(b)

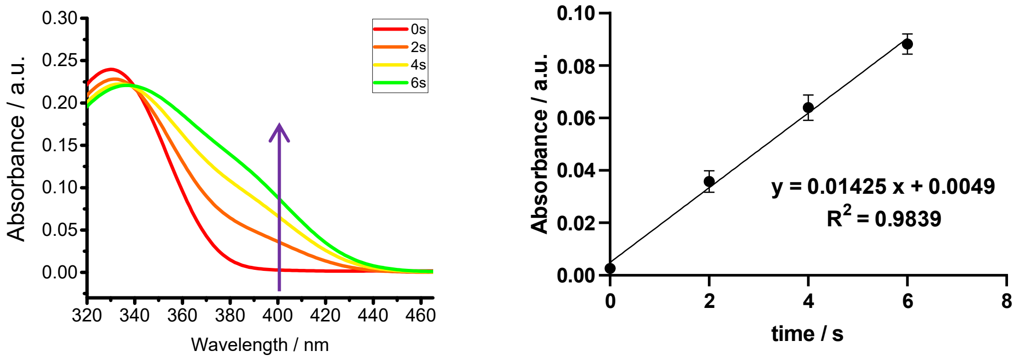

(c)

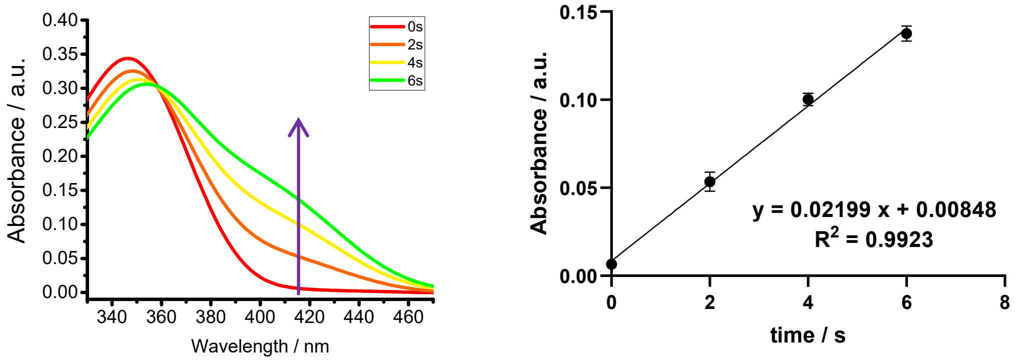

(**d**)

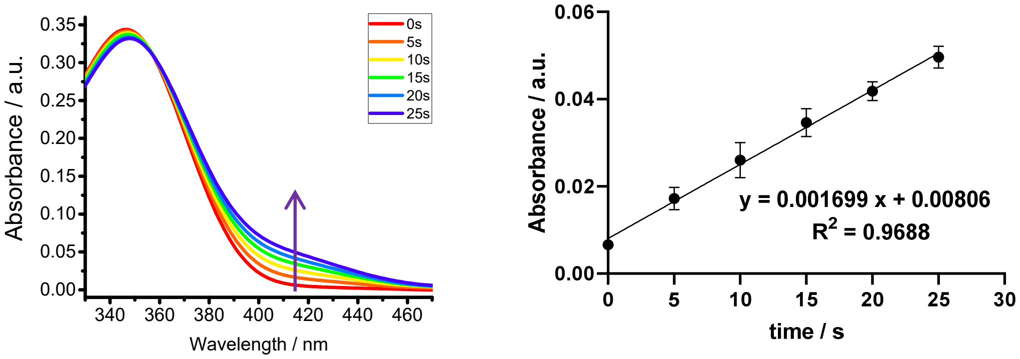

Supplementary Figure 2. Determination of the quantum yields using a potassium ferrioxalate-based chemical actinometer. (**a**) Time-course of absorbance change of formation of the Fe^{2+}-(1,10-phenanthroline)$_3$ complex at 510 nm was induced by the 311 nm UV lamp (left) or 405 nm laser (right) irradiation to the actinometer with a linear fitting curve. Time-course of absorbance changes of product formation from DASyd (**b**) **1d** at 311 nm (monitored at 400 nm for absorbance changes); (**c**) from **1g** at 311nm (monitored at 400 nm for absorbance changes); (**d**) from **1g** at 405 nm (monitored at 415 nm for absorbance changes) to corresponding pyrazoline products with a linear fitting curve. A solution of 20 μM DASyds **1d** and **1g** and 800 μM Methyl methacrylate (MMA) in ACN/H_2O (1:1) in the same quartz cuvette were photo-irradiated for a specified time before absorbance measurement, respectively. Error bars denote standard deviation from three experimental replicates (n = 3).

(a)

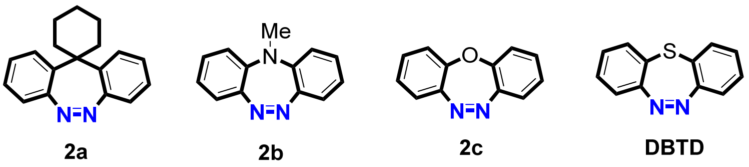
Me
N
O
S
N=N
2a
2b
2c
DBTD

(b)

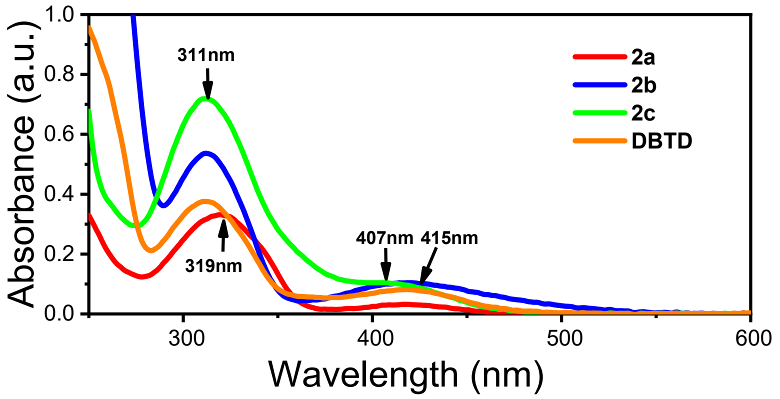
Absorbance (a.u.)
Wavelength (nm)
2a
2b
2c
DBTD
311nm
319nm
407nm 415nm

(c)

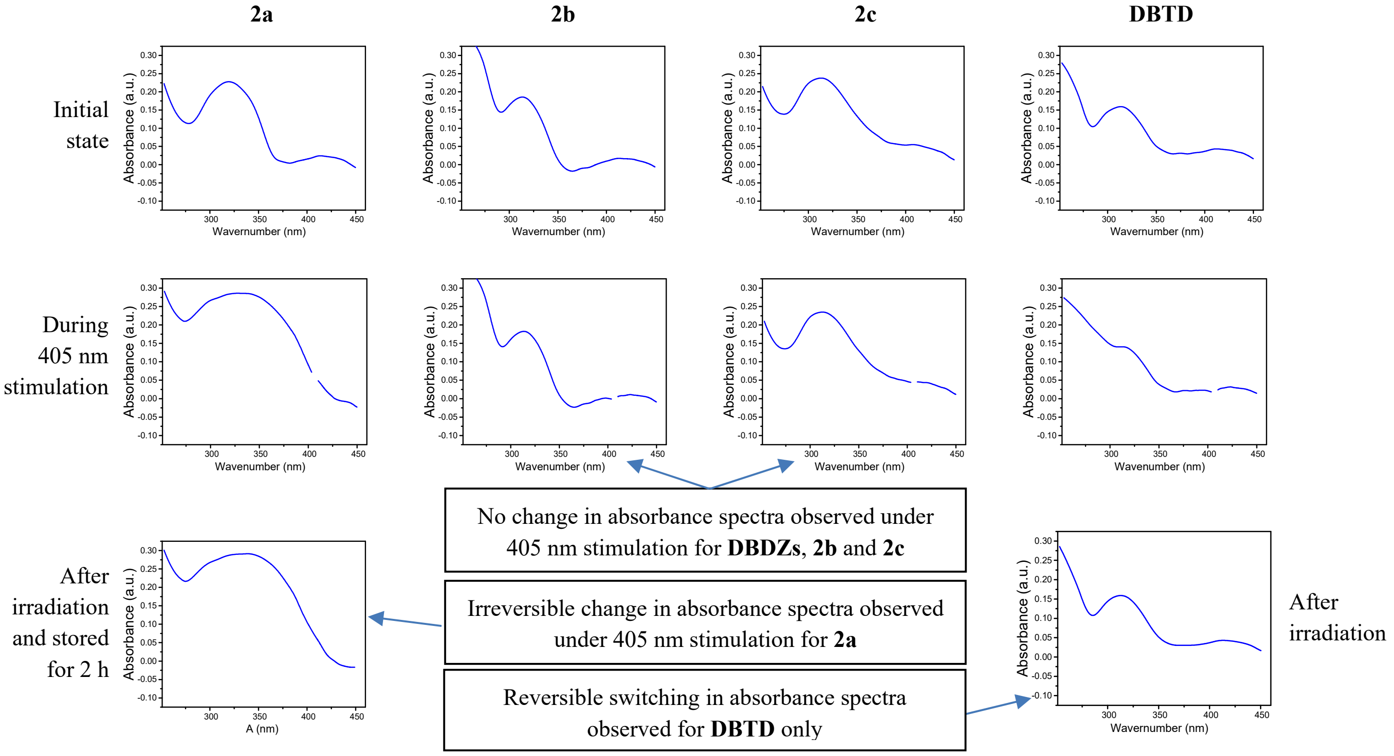
2a
2b
2c
DBTD
Initial state
During 405 nm stimulation
After irradiation and stored for 2 h
After irradiation
Absorbance (a.u.)
Wavenumber (nm)
A (nm)
No change in absorbance spectra observed under 405 nm stimulation for DBDZs, 2b and 2c
Irreversible change in absorbance spectra observed under 405 nm stimulation for 2a
Reversible switching in absorbance spectra observed for DBTD only

Supplementary Figure 3. (**a**) The structural formula of the dibenzo[1,2]diazepines (DBDZs) used in the photo-switching performance studies. (**b**) The UV-vis spectra of the DBDZs without photo-stimulation at 100 μM in ACN/H_2O (1:1, v/v). (**c**) The absorbance spectra of **2a, 2b, 2c** and **DBTD** before/after irradiation with the 405 nm laser at 50 μM in ACN/H_2O (1:1, v/v). The spectra of **2a** showed an irreversible evolution after irradiation of the 405 nm light. On the other hand, the **DBTD** exhibited reversible responses toward the stimulation of 405 nm laser.

Photo-activated cycloaddition of DASyd 1d/1g with a range of DBDZs in ACN/H2O (1:1) with irradiation of the 311nm, 405 nm or 311 + 405 nm light sources.

DBDZ, 2a:

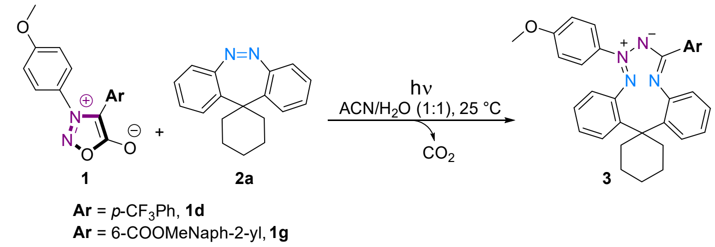

(a)

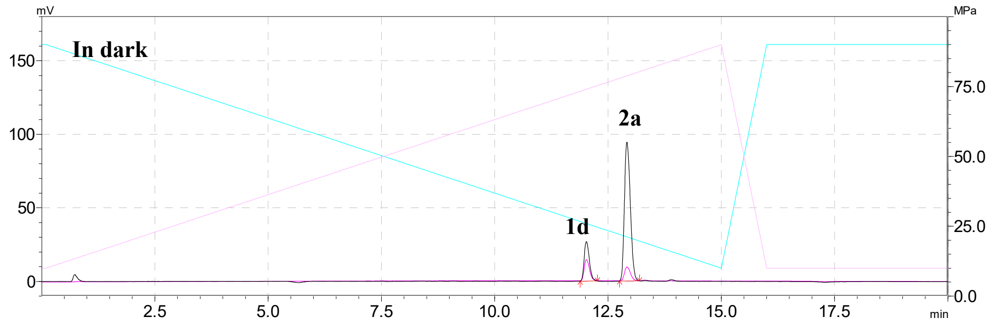

Peak No.	Time	Area	Height	%Area
1	12.022	214565	26746	20.04
2	12.916	856264	94358	79.96

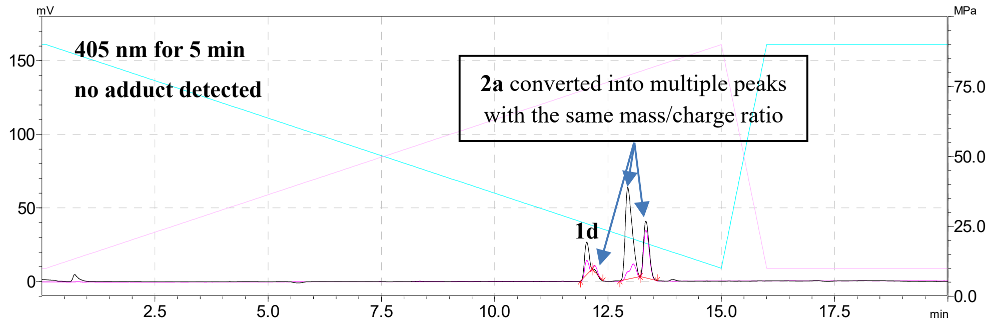

Peak No.	Time	Area	Height	%Area
1	12.030	150409	22144	13.06
2	12.179	2846	868	0.25
3	12.935	667003	62276	57.93
4	13.334	331051	38500	28.75

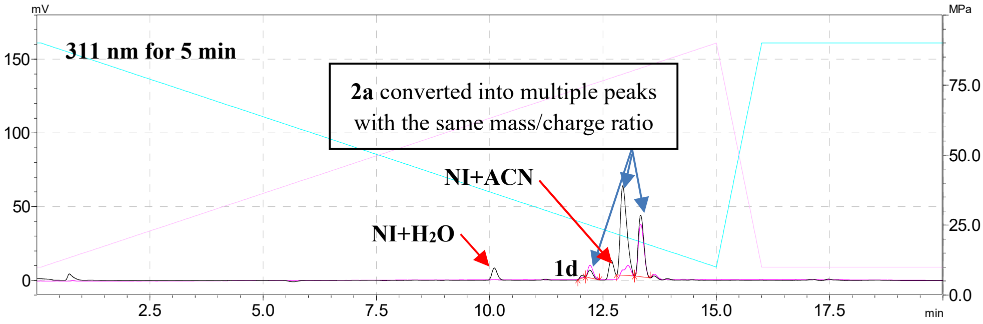

Peak No.	Time	Area	Height	%Area
1	12.037	10582	1866	1.05
2	12.209	40085	5249	3.96
3	12.933	608255	60627	60.05
4	13.329	354066	41698	34.95

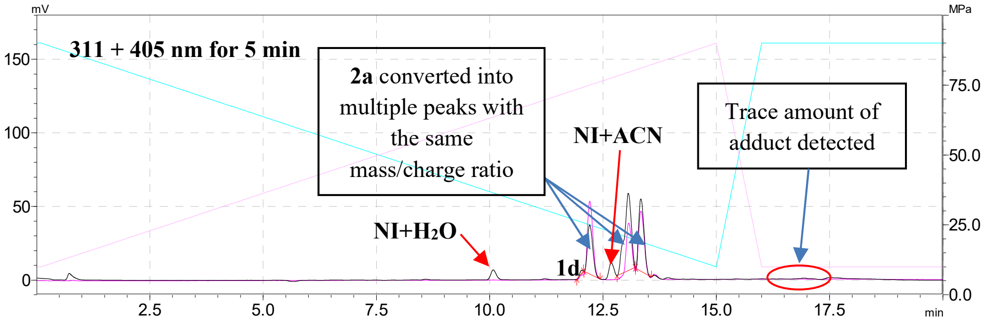

Peak No.	Time	Area	Height	%Area
1	12.034	8471	1838	0.68
2	12.205	272172	33375	21.85
3	13.057	562349	52541	45.15
4	13.334	402620	49019	32.32

(b)

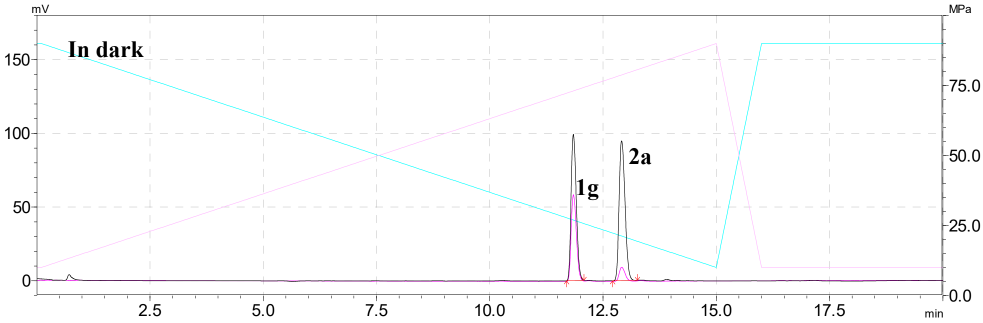

Peak No.	Time	Area	Height	%Area
1	11.845	795181	99045	47.92
2	12.910	864084	94707	52.08

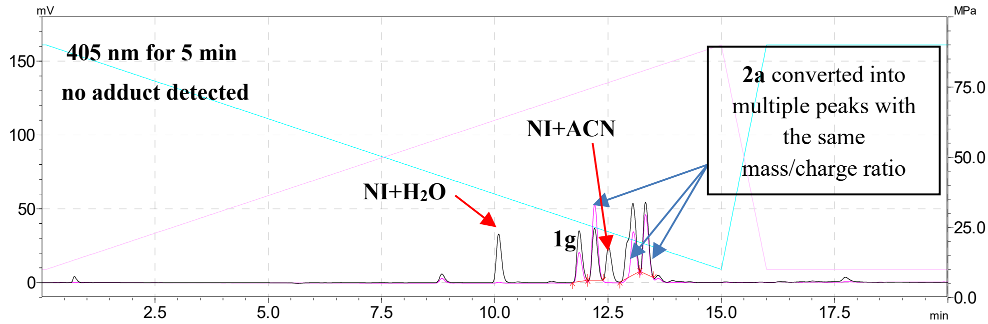

Peak No.	Time	Area	Height	%Area
1	11.863	277389	34345	18.22
2	12.202	288107	35177	18.92
3	13.048	562329	48591	36.93
4	13.327	394736	48401	25.93

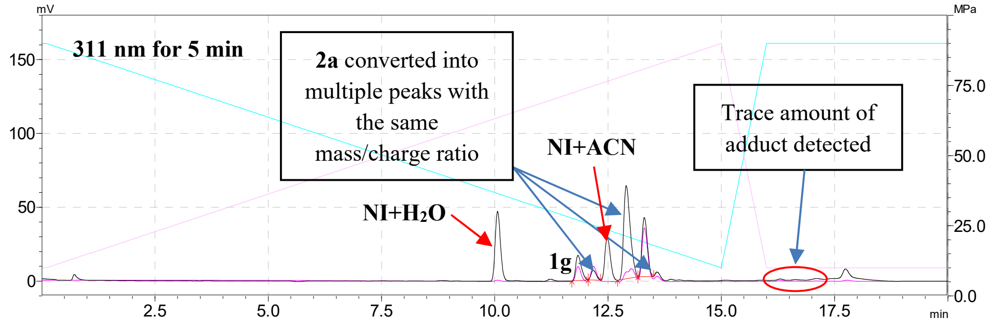

Peak No.	Time	Area	Height	%Area
1	11.837	148223	17003	12.69
2	12.175	51089	6451	4.37
3	12.901	635131	62953	54.38
4	13.300	333541	39983	28.56

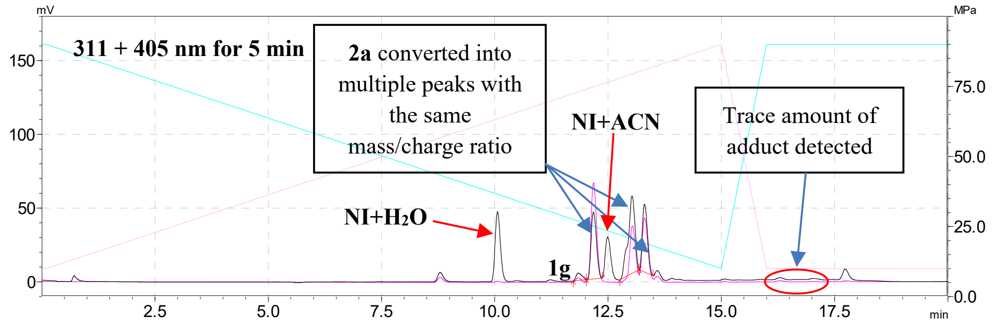

Peak No.	Time	Area	Height	%Area
1	11.846	44842	4953	3.33
2	12.178	371661	45127	27.61
3	13.029	556890	52444	41.37
4	13.305	372808	45985	27.69

Supplementary Figure 4. (**a**) HPLC analysis for the photo-induced intermolecular cycloaddition reaction between DASyd **1d** and **2a**. (**b**) HPLC analysis for the photo-induced intermolecular cycloaddition reaction between DASyd **1g** and **2a**. Two reactions were in ACN/H_2O (1:1, v/v): HPLC trace of reaction mixture after 5 min photo-irradiation with the 405 nm laser, the 311 nm UV lamp or the 311 + 405 nm combination, respectively. The concentration of the DASyd and **2a** used in the reaction were 10 μM and 50 μM, respectively. The conversion from **1** to **3** was calculated based on the absorbance at 254 nm, [NI = nitrile imine]. There were some isomers of **2a** detected after irradiation with 311 nm or 405 nm light. The mass identification of **2a** was confirmed by LC-MS: MS (ESI) calcd. for **2a** $C_{18}H_{19}N_2^+$ 263.15 [M+H$^+$], found 263.12.

DBDZ, 2b:

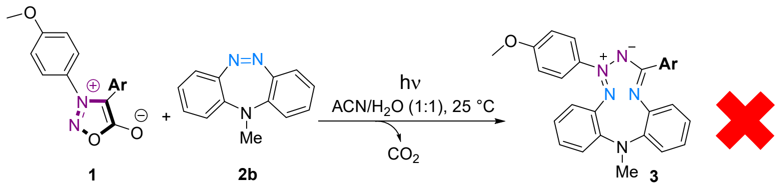

Ar = *p*-CF_3Ph, **1d**
Ar = 6-COOMeNaph-2-yl, **1g**

(a)

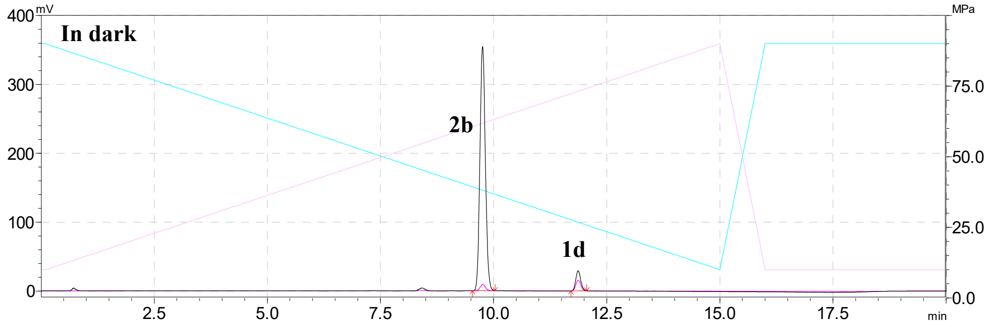

Peak No.	Time	Area	Height	%Area
1	9.756	2983229	353819	93.11
2	11.867	220855	28992	6.893

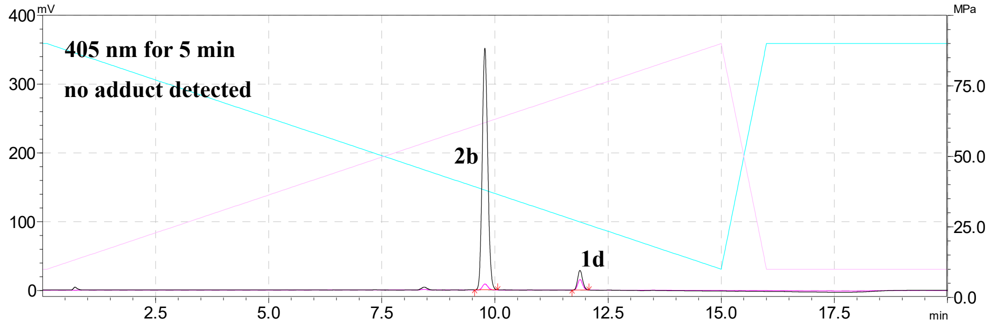

Peak No.	Time	Area	Height	%Area
1	9.777	2988060	350224	93.17
2	11.878	219062	28359	6.83

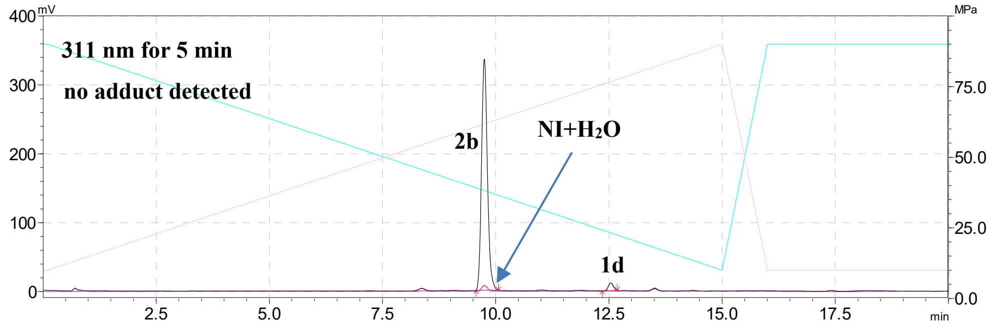

Peak No.	Time	Area	Height	%Area
1	9.749	3026297	334970	97.30
2	12.541	84065	10981	2.70

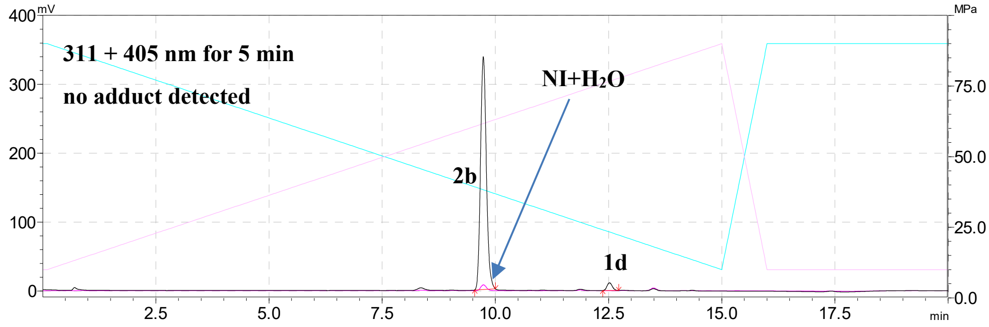

Peak No.	Time	Area	Height	%Area
1	9.733	3033220	337603	97.44
2	12.519	79626	11120	2.56

(b)

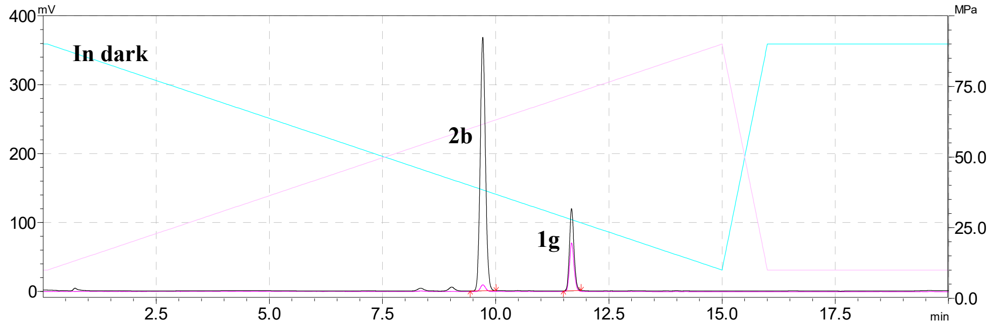

Peak No.	Time	Area	Height	%Area
1	9.714	2951287	367662	78.01
2	11.675	831904	118982	21.99

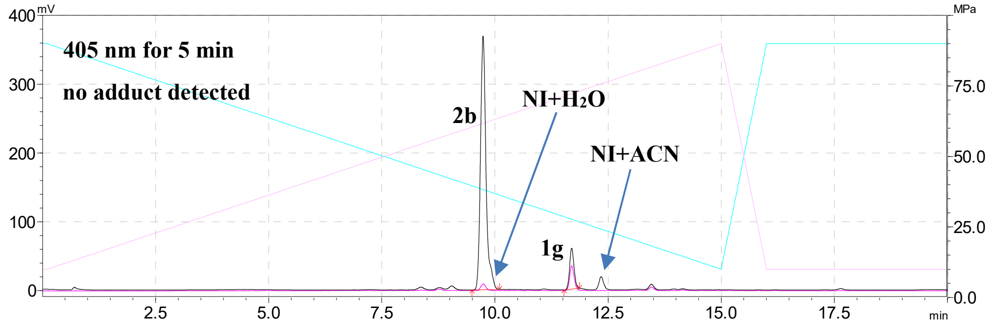

Peak No.	Time	Area	Height	%Area
1	9.737	3147961	367820	88.61
2	11.696	404820	59294	11.39

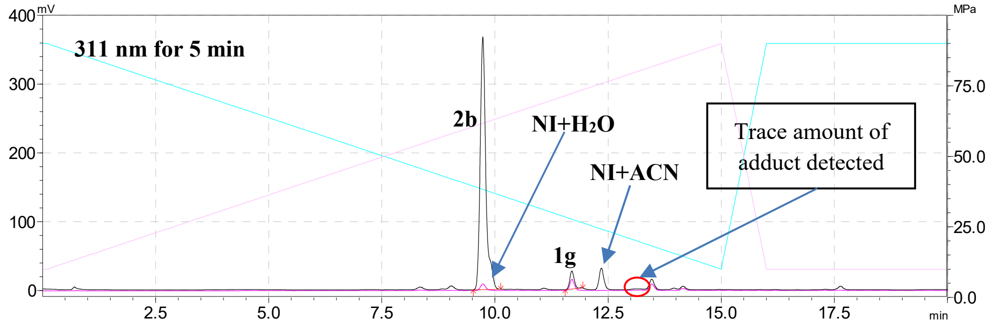

Peak No.	Time	Area	Height	%Area
1	9.731	3210997	367108	94.72
2	11.700	179016	26236	5.28

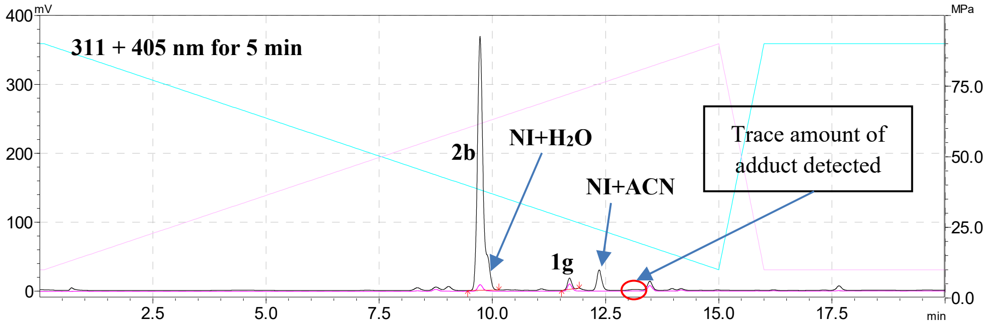

Peak No.	Time	Area	Height	%Area
1	9.726	3272813	367857	96.97
2	11.704	102265	16333	3.03

Supplementary Figure 5. (**a**) HPLC analysis for the photo-induced intermolecular cycloaddition reaction between DASyd **1d** and **2b**. (**b**) HPLC analysis for the photo-induced intermolecular cycloaddition reaction between DASyd **1g** and **2b**. Two reactions were in ACN/H_2O (1:1, v/v): HPLC trace of reaction mixture after 5min photo-irradiation with the 405 nm laser, the 311 nm UV lamp or the 311 + 405 nm combination, respectively. The concentration of DASyd and **2b** used in the reaction were 10 μM and 50 μM, respectively. The conversion from **1** to **3** was calculated based on the absorbance at 254 nm, [NI = nitrile imine].

DBDZ, 2c:

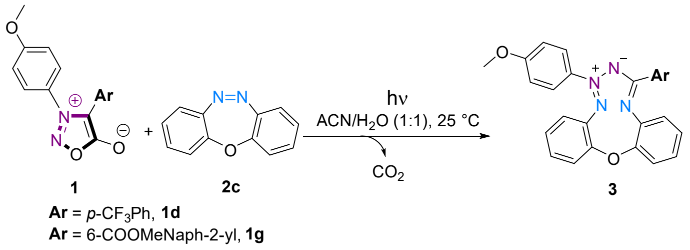

(a)

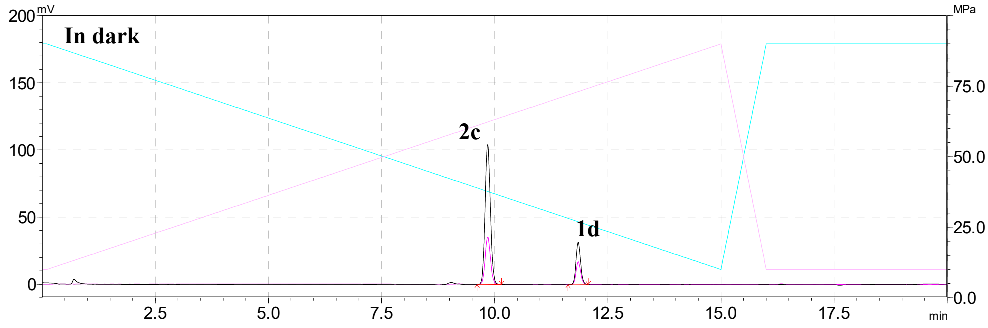

Peak No.	Time	Area	Height	%Area
1	9.845	853199	103785	79.20
2	11.844	224033	31465	20.80

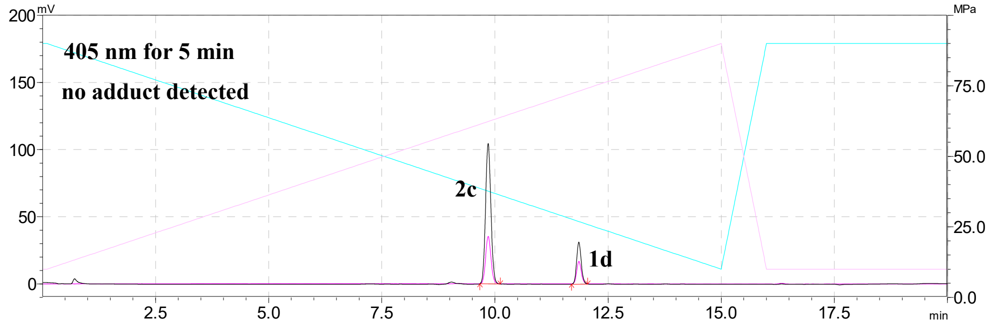

Peak No.	Time	Area	Height	%Area
1	9.850	859386	104409	79.54
2	11.853	221010	31148	20.46

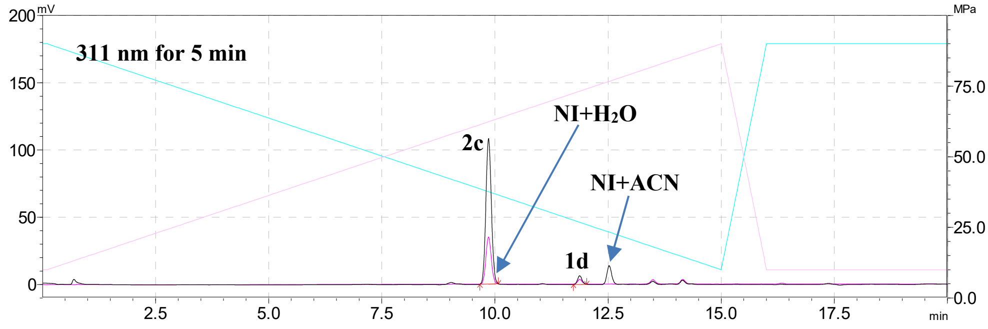

Peak No.	Time	Area	Height	%Area
1	9.859	909573	108129	95.31
2	11.871	44736	6441	4.69

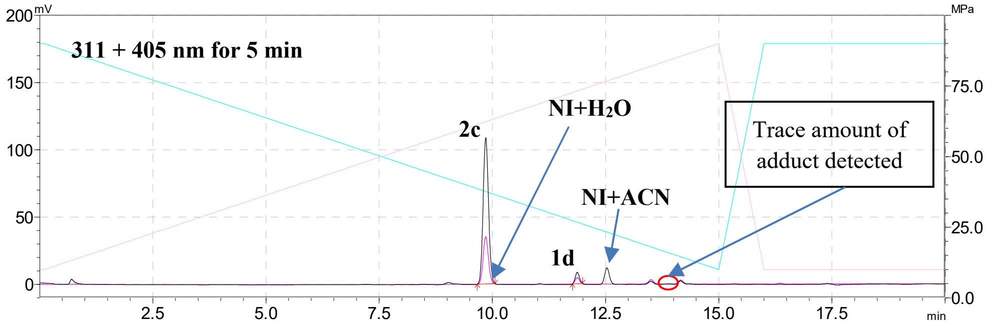

Peak No.	Time	Area	Height	%Area
1	9.854	901085	108432	95.29
2	11.874	44528	8445	4.71

(b)

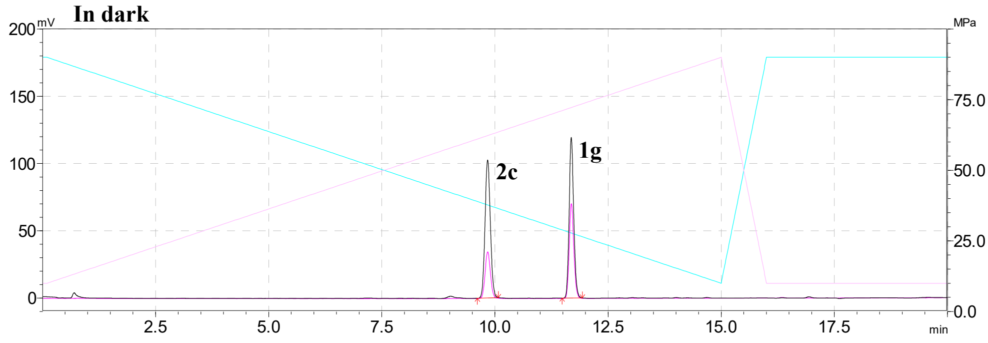

Peak No.	Time	Area	Height	%Area
1	9.838	842261	102396	49.93
2	11.687	844796	118849	50.07

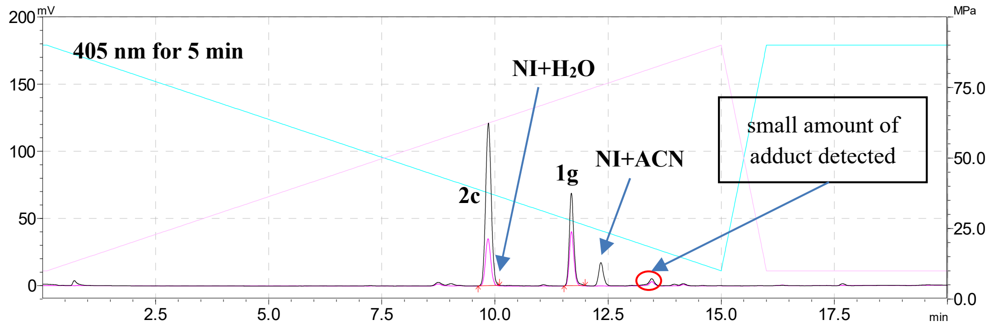

Peak No.	Time	Area	Height	%Area
1	9.856	1038758	120921	68.00
2	11.689	488916	68833	32.00

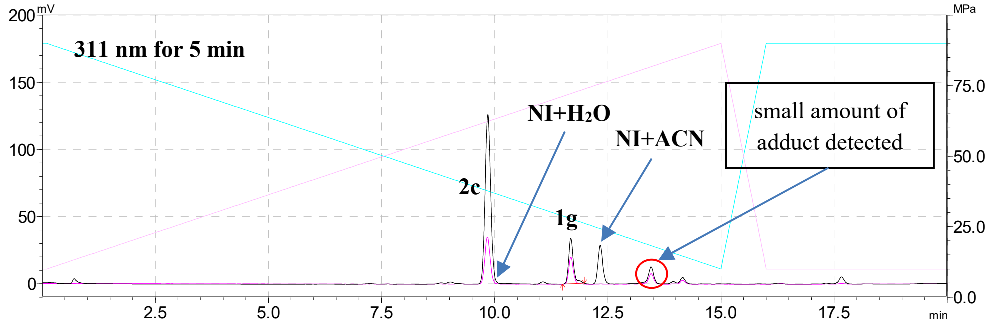

Peak No.	Time	Area	Height	%Area
1	11.680	251555	33850	100

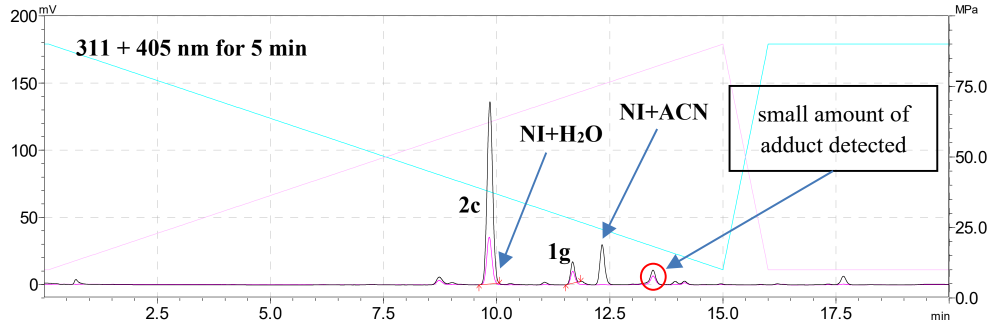

Peak No.	Time	Area	Height	%Area
1	9.851	1167813	135308	91.87
2	11.680	103293	15905	8.13

Supplementary Figure 6. (**a**) HPLC analysis for the photo-induced intermolecular cycloaddition reaction between DASyd **1d** and **2c**. (**b**) HPLC analysis for the photo-induced intermolecular cycloaddition reaction between DASyd **1g** and **2c**. Two reactions were in ACN/H_2O (1:1, v/v): HPLC trace of reaction mixture after 5 min photo-irradiation with the 405 nm laser, the 311 nm UV lamp or the 311 + 405 nm combination, respectively. The concentration of DASyd and **2c** used in the reaction were 10 μM and 50 μM, respectively. The conversion from **1** to **3** was calculated based on the absorbance at 254 nm, [NI = nitrile imine].

Supplementary Table 1. Screening of DASyd reagent for photo-click reaction with dibenzo[*b*,*f*][1,4,5]thiadiazepine (**DBTD**) to isolate of the **MAI** product and compare the photo-conversion under different irradiation conditions and under diluted conditions.[a]

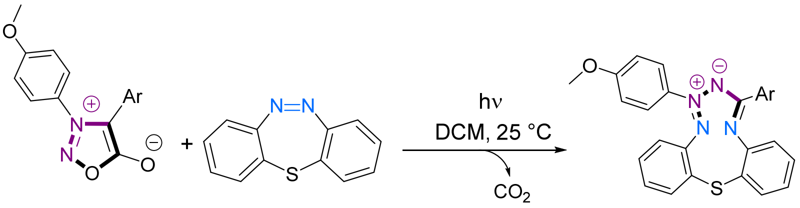

1 DBTD 3

Entry[a]	DASyds		Isolated Yield (%) at 1mM concentration on 0.12 mmol scale[b]	HPLC conversion at 10 μM concentration[d]	
		Ar		311 nm	311 + 405 nm
1	1a	*p*-FC_6H_4	67	91.3%	94.5%
2	1b	*m*-2F-*p*-FC_6H_2	58	97.2%	97.4%
3	1c	*m*-2F-*p*-$CF_3C_6H_2$	64	93.0%	95.3%
4	1d	*p*-$CF_3C_6H_4$	68	93.8%	95.8%
5	1e	*m*-2$CF_3C_6H_3$	69	96.3%	97.8%
6	1f	*p*-COOEtC_6H_4	73[c]	>99.9%	>99.9%
7	1g	6-COOMe-2-$C_{12}H_6$	81[c]	99.5%	99.8%
8	1h	*m*-2F-*p*-CNC_6H_2	70	99.5%	98.5%
9	1i	*m*-F-*m*-CNC_6H_3	87	98.0%	97.3%
10	1j	*m*-CF_3-*p*-CNC_6H_3	76	95.5%	98.0%

[a]Reactions were conducted with 0.12 mmol of DASyd **1** and 1 eq. **DBTD** in DCM (120 mL) by irradiating via the 311 + 405 nm LED array simultaneously in quartz flask for 1.5 h. [b]Isolated yield (Clean reactions under the preparation scale. Due to the photo-attenuation effect, the conversion of DASyd was not complete, and unconverted starting material was recycled. There was slightly photo-degradation of the desired product **3** detected under continuous photo-

irradiation). [c]Only the 405 nm LED array was used for 2 h. [d]Reactions were conducted in quartz test tube and analyzed by HPLC-MS (For details, please see in Figure S7-16). The reaction mixture was prepared for each DASyd mixed with **DBTD** (final concentration, 10 μM DASyd and 50 μM **DBTD** in ACN:H_2O = 1:1, v/v; 0.6% DMSO, 1 mL volume) to react 5 min with the 311 nm or the 311 + 405 nm combined light, respectively. In addition to the desired MAI cycloadducts, small amounts of hydrolysis products and acetonitrile cycloadducts could also be detected by HPLC-MS.

Sydnone **1a**:

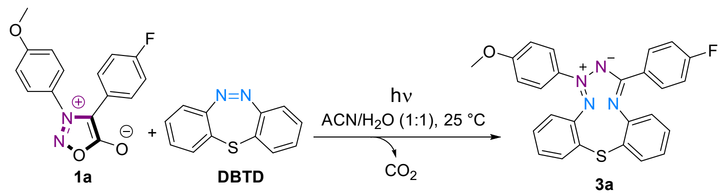

(**a**) For 30 s irradiation time

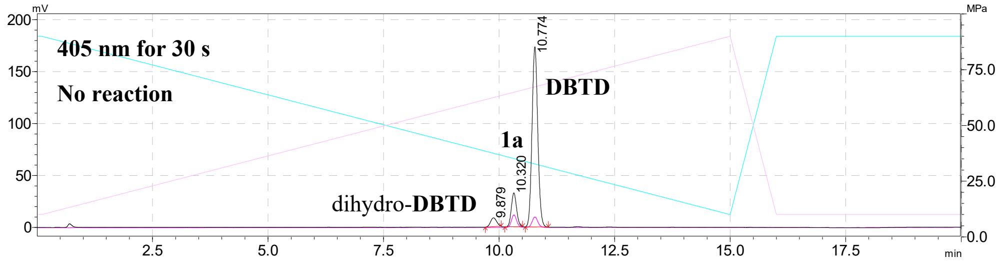

Peak No.	Time	Area	Height	%Area
1	9.879	77056	8514	4.20
2	10.320	256729	32480	13.98
3	10.774	1502617	173230	81.82

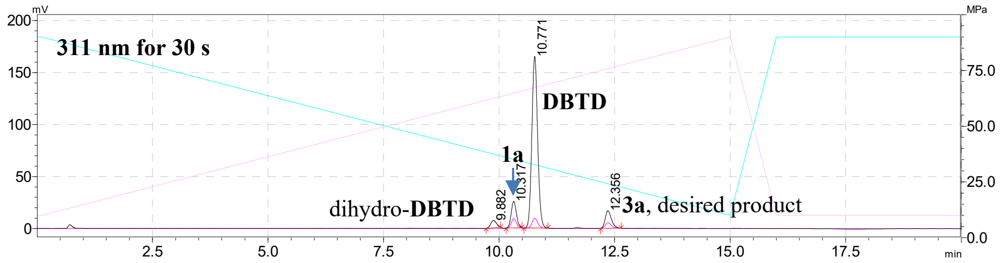

Peak No.	Time	Area	Height	%Area
1	9.882	61272	7065	3.31
2	10.317	197907	25358	10.7
3	10.771	1433763	164635	77.54
4	12.356	156192	16831	8.45

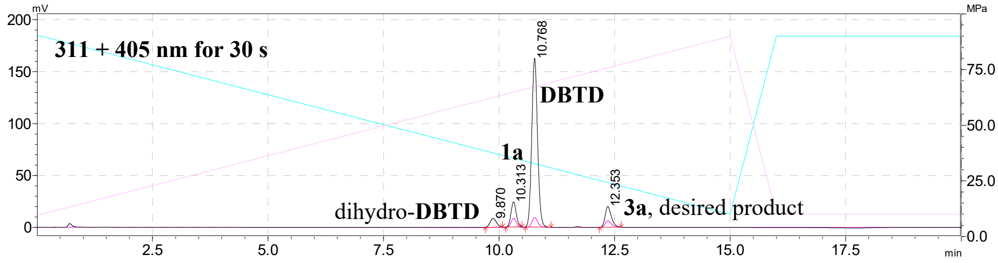

Peak No.	Time	Area	Height	%Area
1	9.870	79301	8407	4.24
2	10.313	189028	24019	10.10
3	10.768	1416923	162113	75.72
4	12.353	185997	19810	9.94

(**b**) For 5 min irradiation time

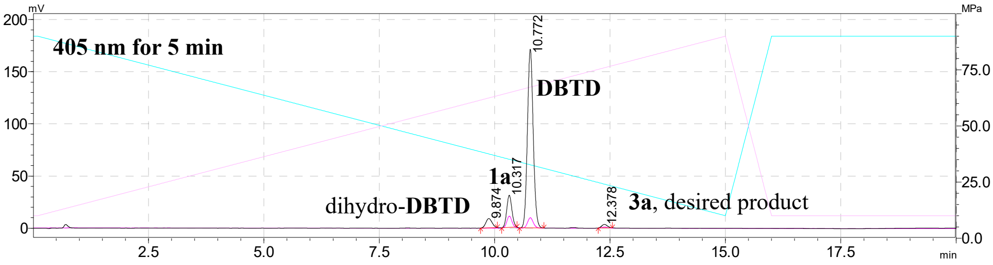

Peak No.	Time	Area	Height	%Area
1	9.874	82632	8866	4.49
2	10.317	239825	30751	13.03
3	10.772	1489409	171124	80.92
4	12.378	28659	3350	1.56

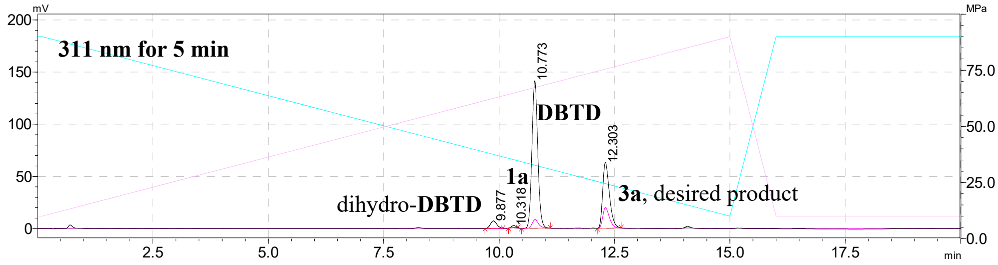

Peak No.	Time	Area	Height	%Area
1	9.877	67879	7169	3.46
2	10.318	15389	2280	0.78
3	10.773	1246738	141322	63.51
4	12.303	633046	62708	32.25

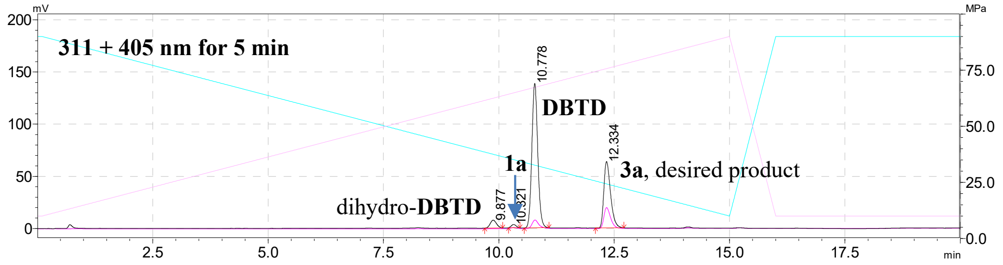

Peak No.	Time	Area	Height	%Area
1	9.877	71335	7514	3.63
2	10.321	24640	3221	1.25
3	10.778	1230069	138197	62.51
4	12.334	641784	63509	32.61

Supplementary Figure 7. HPLC analysis for the photo-induced intermolecular cycloaddition reaction between DASyd **1a** and **DBTD** in ACN/H_2O (1:1, v/v): (**a**) HPLC trace of reaction mixture after 30s photo-irradiation with 405 nm laser, 311 nm UV lamp and 311 + 405 nm combination, respectively; (**b**) HPLC trace of reaction mixture after 5 min photo-irradiation with 405 nm, 311 nm and 311 + 405 nm lamp, respectively. The concentration of **1a** and **DBTD** used in the reaction were 10 µM and 50 µM, respectively. The conversion from **1a** to **3a** was calculated based on the absorbance at 254 nm, [NI = nitrile imine]. Dihydro-**DBTD** was the reduced product of **DBTD** for $C_{12}H_{11}N_2S^+$ 215.06 [M+H$^+$], found 215.03. The mass identification of **3a** was confirmed by LC-MS: MS (ESI) calcd. for **3a** $C_{26}H_{20}FN_4OS^+$ 455.13 [M+H$^+$], found 454.80.

Sydnone **1b**:

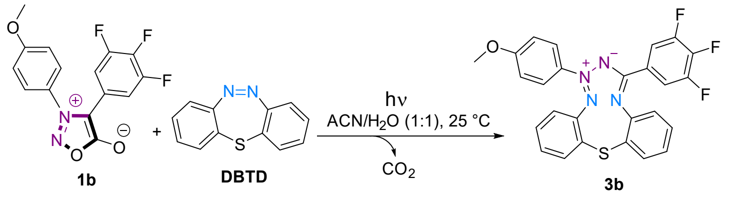

(**a**) For 30 s irradiation time

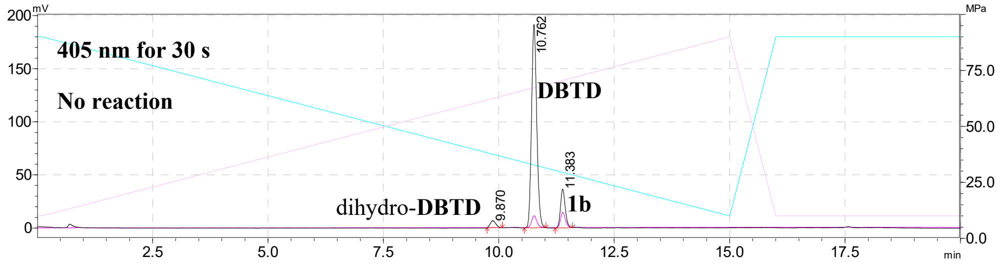

Peak No.	Time	Area	Height	%Area
1	9.870	56655	6689	3.12
2	10.762	1500953	190797	82.54
3	11.383	260815	36461	14.34

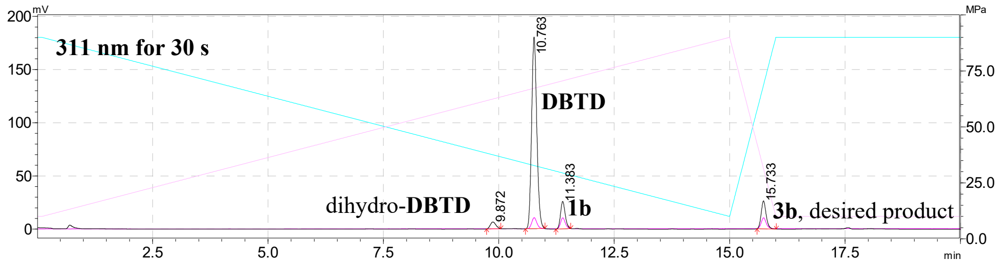

Peak No.	Time	Area	Height	%Area
1	9.872	53466	6416	2.91
2	10.763	1405230	179812	76.505
3	11.383	181313	25750	9.78
4	15.733	196769	26254	10.71

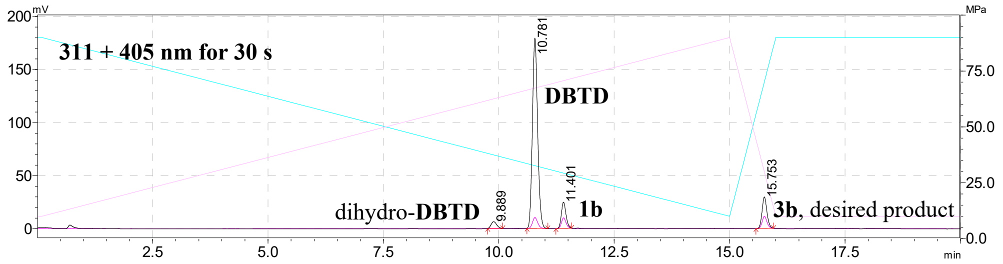

Peak No.	Time	Area	Height	%Area
1	9.889	54987	6490	2.96
2	10.781	1403013	179082	75.64
3	11.401	175543	24861	9.46
4	15.753	221437	29619	11.94

(**b**) For 5 min irradiation time

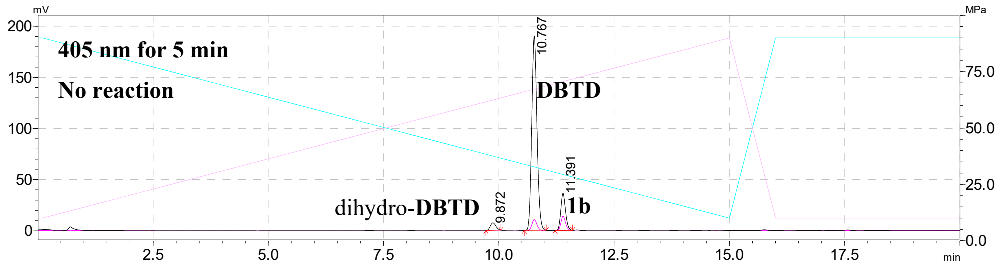

Peak No.	Time	Area	Height	%Area
1	9.872	62191	7341	3.41
2	10.767	1499399	189689	82.32
3	11.391	259852	36223	14.27

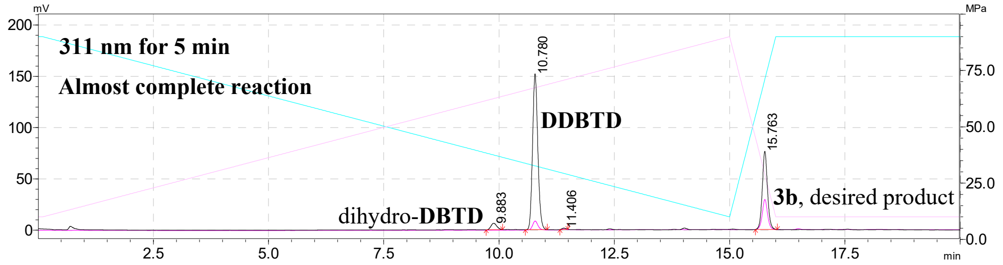

Peak No.	Time	Area	Height	%Area
1	9.883	55232	6296	2.98
2	10.780	1201782	151675	64.90
3	15.763	594727	76034	32.12

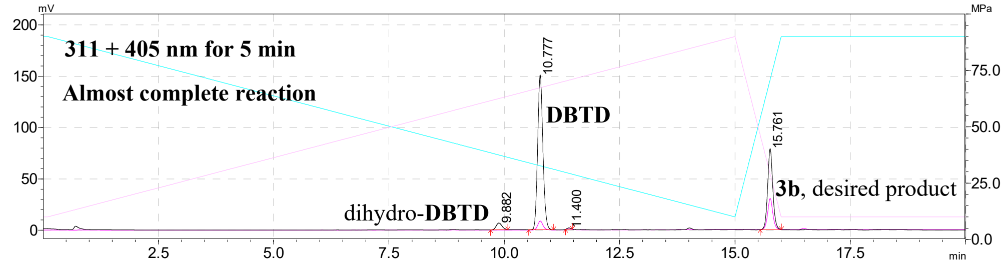

Peak No.	Time	Area	Height	%Area
1	9.882	57516	6572	3.09
2	10.777	1192425	150337	64.05
3	15.761	611765	78480	32.86

Supplementary Figure 8. HPLC analysis for the photo-induced intermolecular cycloaddition reaction between DASyd **1b** and **DBTD** in ACN/H_2O (1:1, v/v): (**a**) HPLC trace of reaction mixture after 30s photo-irradiation with 405 nm laser, 311 nm UV lamp and 311 + 405 nm combination, respectively; (**b**) HPLC trace of reaction mixture after 5 min photo-irradiation with 405 nm, 311 nm and 311 + 405 nm, respectively. The concentration of **1b** and **DBTD** used in the reaction were 10 µM and 50 µM, respectively. The conversion from **1b** to **3b** was calculated based on the absorbance at 254 nm, [NI = nitrile imine]. Dihydro-**DBTD** was the reduced product of **DBTD**. The mass identification of **3b** was confirmed by LC-MS: MS (ESI) calcd. for **3b** $C_{26}H_{18}F_3N_4OS^+$ 491.11 [M+H$^+$], found 490.80.

Sydnone **1c**:

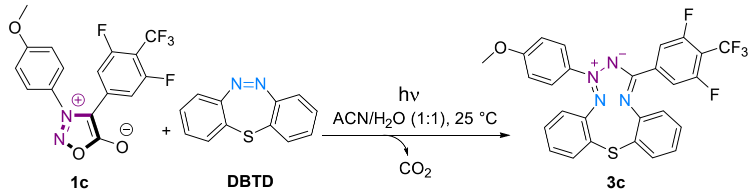

(**a**) For 30 s irradiation time

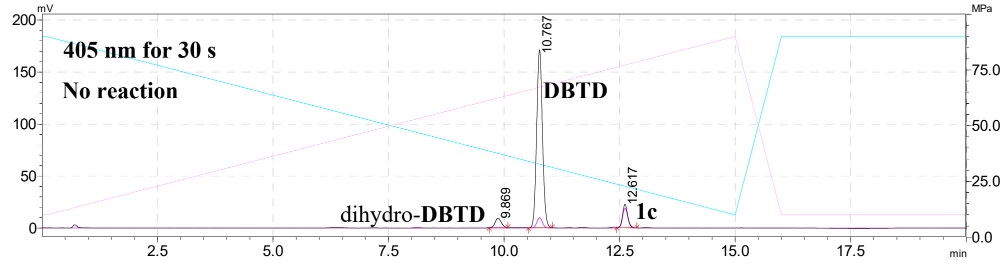

Peak No.	Time	Area	Height	%Area
1	9.869	83897	8879	4.82
2	10.767	1487341	170756	85.42
3	12.617	169987	22173	9.76

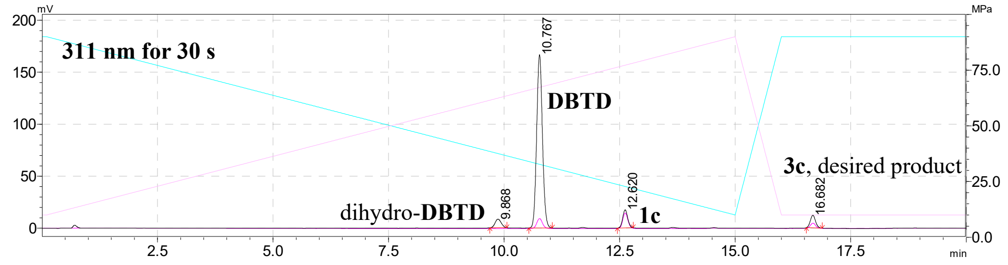

Peak No.	Time	Area	Height	%Area
1	9.868	76572	8263	4.38
2	10.767	1446354	166332	82.67
3	12.620	131631	17305	7.52
4	16.682	95011	12143	5.43

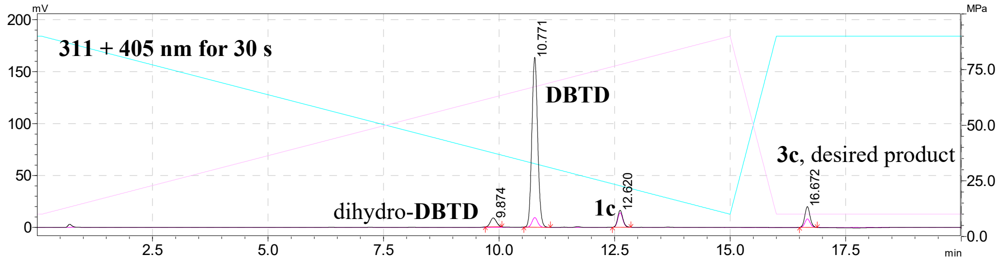

Peak No.	Time	Area	Height	%Area
1	9.874	77594	8452	4.33
2	10.771	1427918	163203	79.75
3	12.620	125185	16214	6.99
4	16.672	159719	20018	8.92

(**b**) For 5 min irradiation time

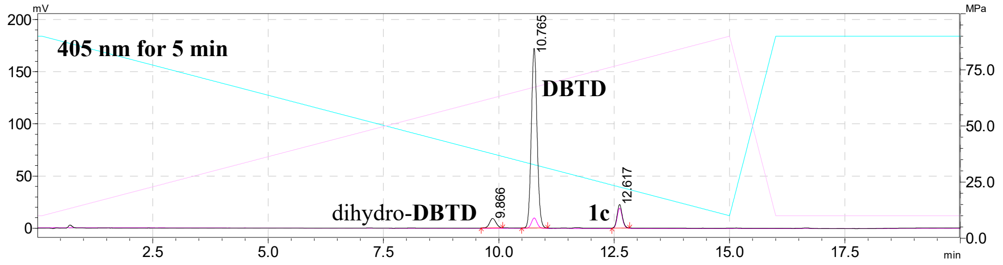

Peak No.	Time	Area	Height	%Area
1	9.866	87655	9001	4.98
2	10.765	1499369	172031	85.15
3	12.617	173796	22515	9.87

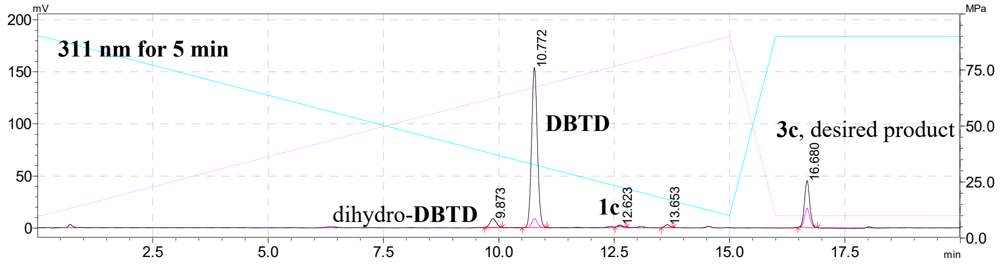

Peak No.	Time	Area	Height	%Area
1	9.873	77787	8329	4.29
2	10.772	1341001	153550	73.96
3	12.623	15280	1910	0.84
4	16.680	358235	44963	19.76

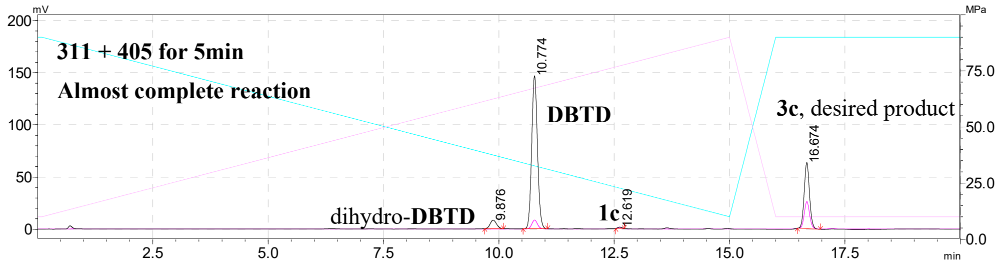

Peak No.	Time	Area	Height	%Area
1	9.876	8129	8129	4.14
2	10.774	1274450	146511	68.18
3	12.619	11426	1579	0.61
4	16.674	505922	63257	27.07

Supplementary Figure 9. HPLC analysis for the photo-induced intermolecular cycloaddition reaction between DASyd **1c** and **DBTD** in ACN/H_2O (1:1, v/v): (**a**) HPLC trace of reaction mixture after 30s photo-irradiation with 405 nm laser, 311 nm UV lamp and 311 + 405 nm combination, respectively; (**b**) HPLC trace of reaction mixture after 5 min photo-irradiation with 405 nm, 311 nm and 311 + 405 nm, respectively. The concentration of **1c** and **DBTD** used in the reaction were 10 µM and 50 µM, respectively. The conversion from **1c** to **3c** was calculated based on the absorbance at 254 nm, [NI = nitrile imine]. Dihydro-**DBTD** was the reduced product of **DBTD** for $C_{12}H_{11}N_2S^+$ 215.06 [M+H$^+$], found 215.03. The mass identification of **3c** was confirmed by LC-MS: MS (ESI) calcd. for **3c** $C_{27}H_{18}F_5N_4OS^+$ 541.11 [M+H$^+$], found 540.76.

Sydnone **1d**:

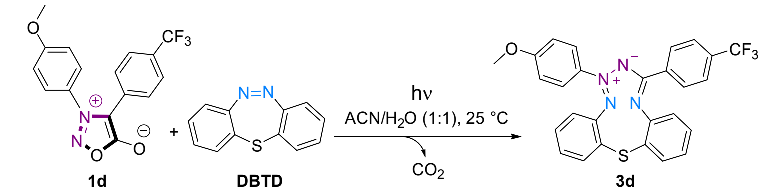

(**a**) For 30 s irradiation time

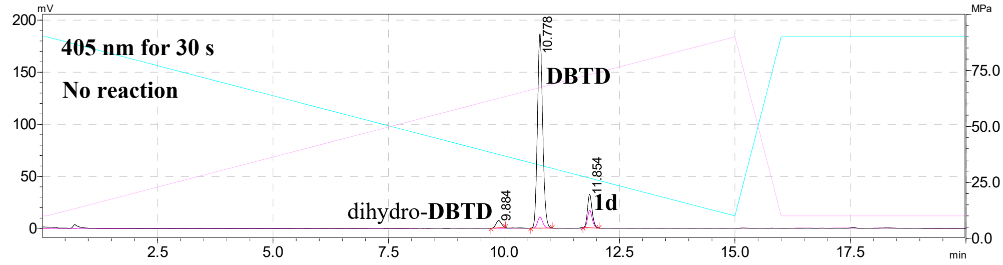

Peak No.	Time	Area	Height	%Area
1	9.884	57291	6992	3.28
2	10.778	1468898	185854	83.98
3	11.854	222988	31906	12.75

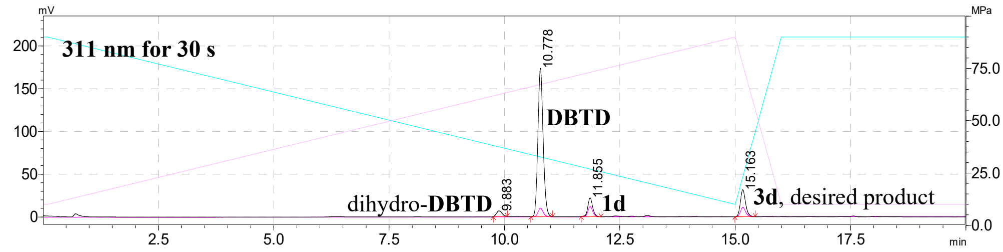

Peak No.	Time	Area	Height	%Area
1	9.883	53977	6546	2.96
2	10.778	1367398	172968	75.01
3	11.855	156524	21787	8.59
4	15.163	244984	31539	13.44

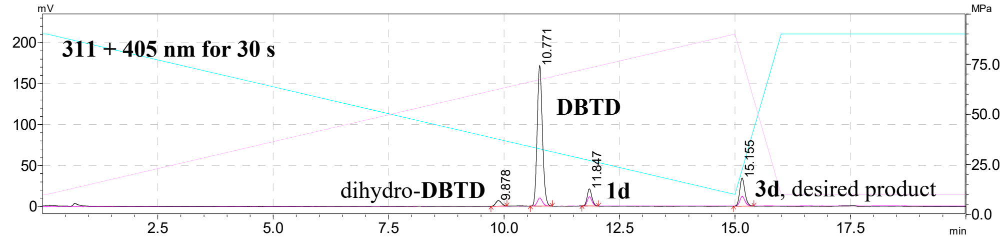

Peak No.	Time	Area	Height	%Area
1	9.878	58284	6818	3.18
2	10.771	1353047	171386	73.85
3	11.847	150213	21126	8.36
4	15.155	267636	34400	14.61

(**b**) For 5 min irradiation time

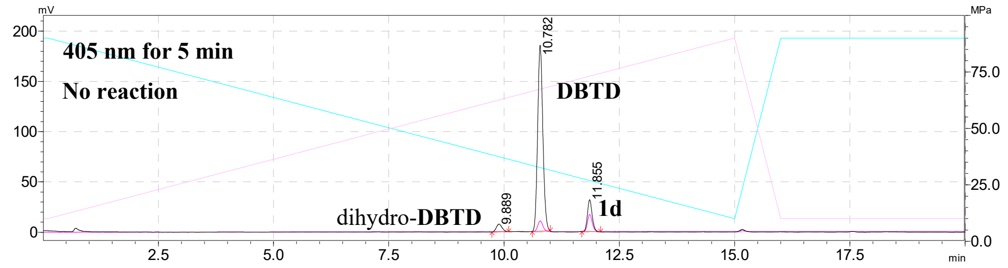

Peak No.	Time	Area	Height	%Area
1	9.889	66251	7614	3.80
2	10.782	1454161	184943	83.39
3	11.855	223453	31617	12.81

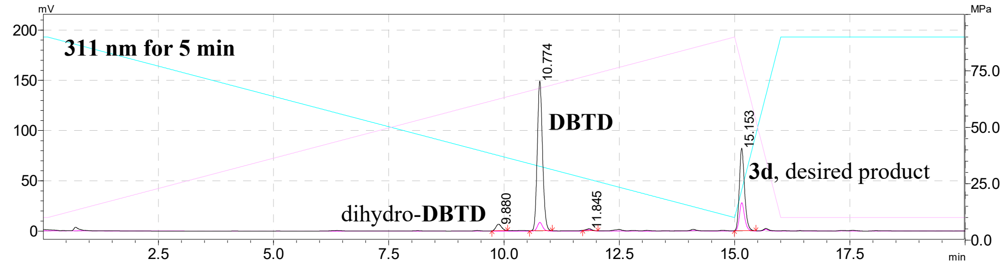

Peak No.	Time	Area	Height	%Area
1	9.880	55760	6514	2.98
2	10.774	1176770	148882	62.83
3	15.153	640543	81771	34.20

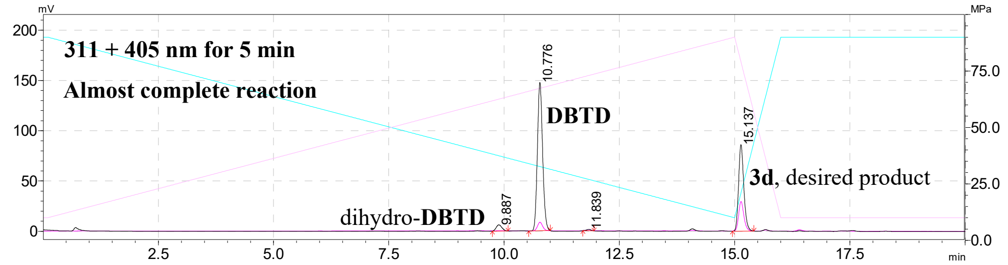

Peak No.	Time	Area	Height	%Area
1	9.887	53310	6169	2.83
2	10.776	1157398	147152	61.33
3	15.137	676592	85676	35.85

Supplementary Figure 10. HPLC analysis for the photo-induced intermolecular cycloaddition reaction between DASyd **1d** and **DBTD** in ACN/H_2O (1:1, v/v): (**a**) HPLC trace of reaction mixture after 30s photo-irradiation with 405 nm laser, 311 nm UV lamp and 311 + 405 nm combination, respectively; (**b**) HPLC trace of reaction mixture after 5 min photo-irradiation with 405 nm, 311 nm and 311 + 405 nm, respectively. The concentration of **1c** and **DBTD** used in the reaction were 10 µM and 50 µM, respectively. The conversion from **1e** to **3d** was calculated based on the absorbance at 254 nm, [NI = nitrile imine]. Dihydro-**DBTD** was the reduced product of **DBTD** for $C_{12}H_{11}N_2S^+$ 215.06 [M+H$^+$], found 215.03. The mass identification of **3d** was confirmed by LC-MS: MS (ESI) calcd. for **3d** $C_{27}H_{20}F_3N_4OS^+$ 505.13 [M+H$^+$], found 504.60.

Sydnone **1e**:

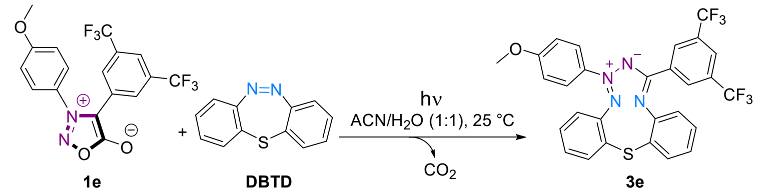

(**a**) For 30 s irradiation time

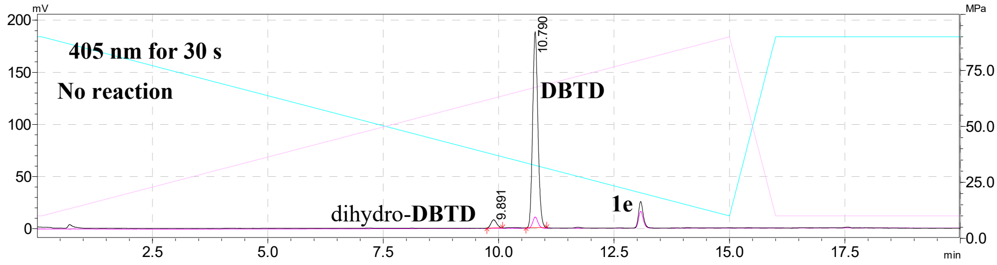

Peak No.	Time	Area	Height	%Area
1	9.883	45136	6023	2.58
2	10.780	1538334	212823	87.79
3	13.072	168820	26195	9.63

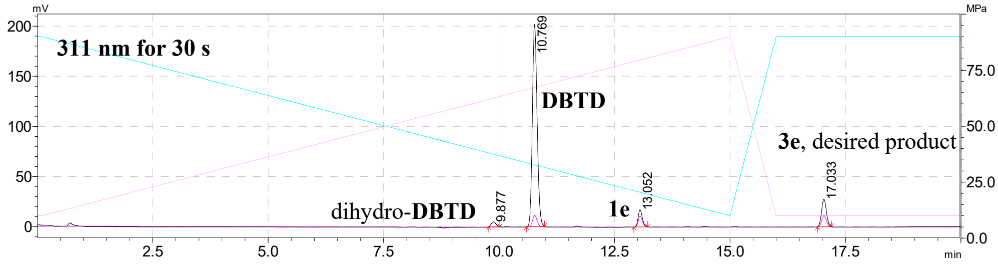

Peak No.	Time	Area	Height	%Area
1	9.877	34632	4878	1.94
2	10.769	1437293	172968	80.63
3	13.052	109841	16959	6.16
4	17.033	200724	27323	11.26

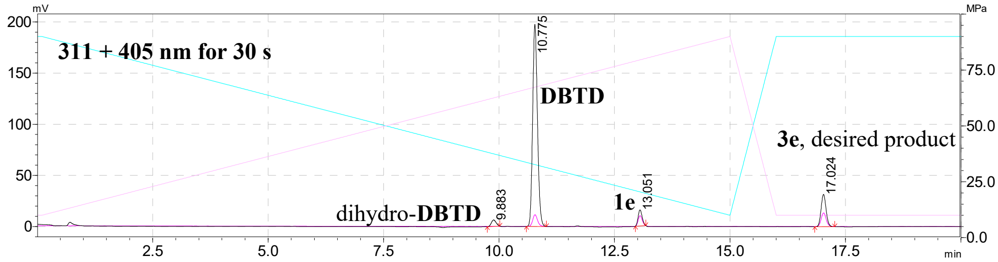

Peak No.	Time	Area	Height	%Area
1	9.883	45653	6309	2.54
2	10.775	1419385	197111	78.93
3	13.051	95896	15676	5.33
4	17.024	237272	31588	13.20

(**b**) For 5 min irradiation time

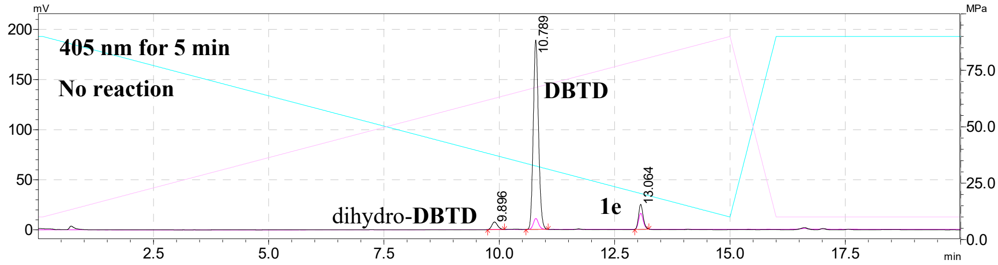

Peak No.	Time	Area	Height	%Area
1	9.896	64738	7507	3.75
2	10.789	1490022	188819	86.31
3	13.064	171599	24845	9.94

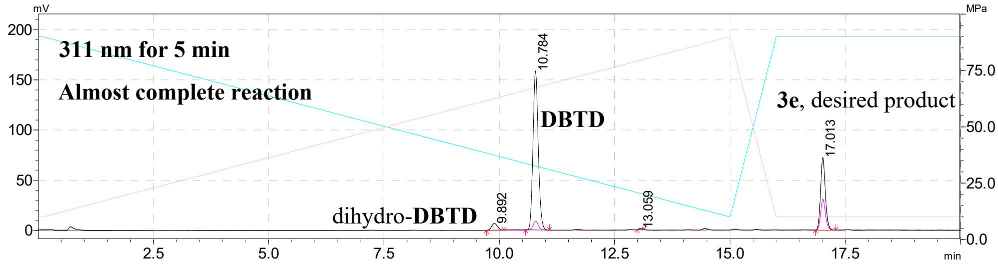

Peak No.	Time	Area	Height	%Area
1	9.892	58585	6748	3.20
2	10.784	1253121	158087	68.34
3	17.013	520946	72345	28.42

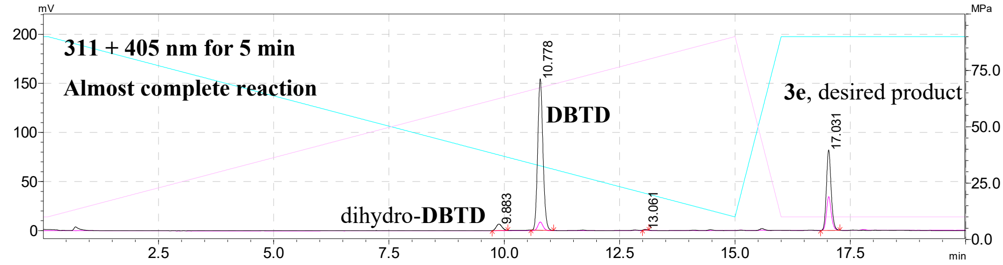

Peak No.	Time	Area	Height	%Area
1	9. 883	56233	6516	3.03
2	10. 778	1218792	153530	65.59
3	17.031	583168	81726	31.38

Supplementary Figure 11. HPLC analysis for the photo-induced intermolecular cycloaddition reaction between DASyd **1e** and **DBTD** in ACN/H_2O (1:1, v/v): (**a**) HPLC trace of reaction mixture after 30s photo-irradiation with 405 nm laser, 311 nm UV lamp and 311 + 405 nm combination, respectively; (**b**) HPLC trace of reaction mixture after 5 min photo-irradiation with 405 nm, 311 nm and 311 + 405 nm, respectively. The concentration of **1e** and **DBTD** used in the reaction were 10 µM and 50 µM, respectively. The conversion from **1e** to **3e** was calculated based on the absorbance at 254 nm, [NI = nitrile imine]. Dihydro-**DBTD** was the reduced product of **DBTD** for $C_{12}H_{11}N_2S^+$ 215.06 [M+H$^+$], found 215.03. The mass identification of **3e** was confirmed by LC-MS: MS (ESI) calcd. for **3e** $C_{28}H_{19}F_6N_4OS^+$ 573.12 [M+H$^+$], found 572.80.

Sydnone **1f**:

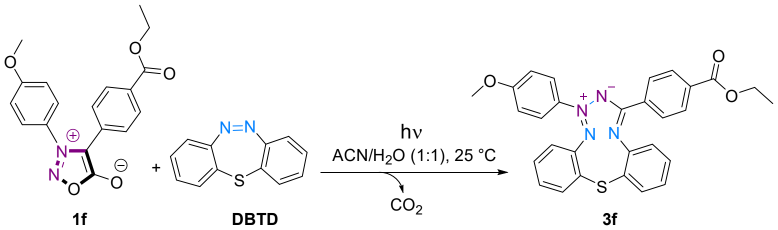

(**a**) For 30 s irradiation time

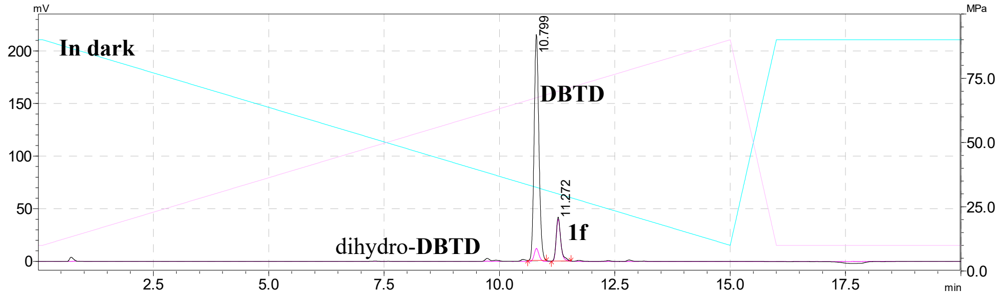

Peak No.	Time	Area	Height	%Area
1	10.799	1545162	214456	84.16
2	11.272	290912	41622	15.84

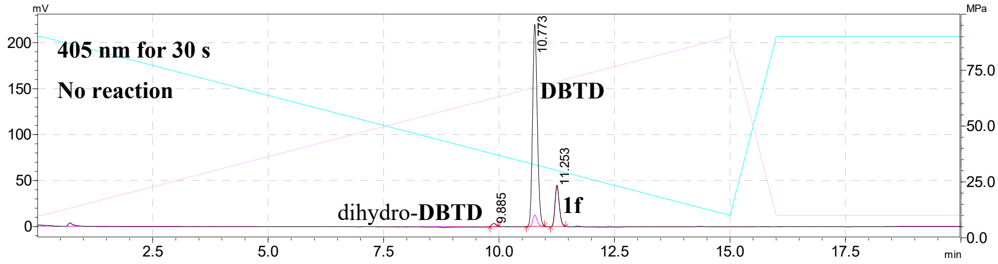

Peak No.	Time	Area	Height	%Area
1	9. 885	19131	3047	1.02
2	10. 773	1563214	219397	83.30
3	11.253	294250	44831	15.68

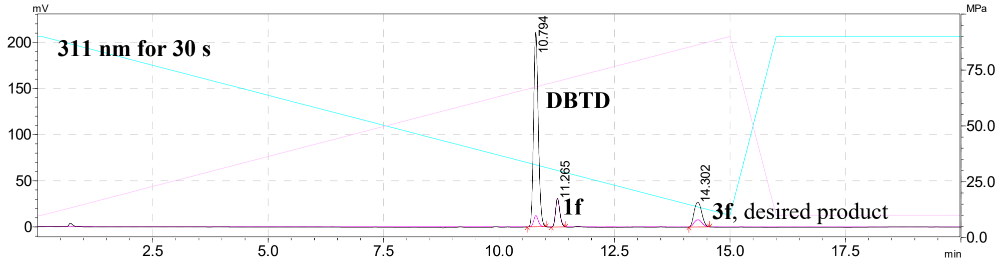

Peak No.	Time	Area	Height	%Area
1	10.794	1482369	209407	74.28
2	11.265	200732	31044	10.06
3	14.302	312550	26772	15.66

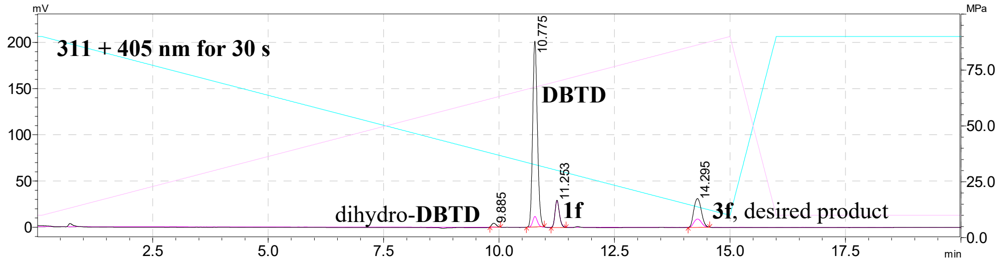

Peak No.	Time	Area	Height	%Area
1	9. 885	71592	9170	3.16
2	10. 783	1446906	201522	63.93
3	11.261	240825	36551	10.64
4	14.167	503974	62674	22.27

(**b**) For 5 min irradiation time

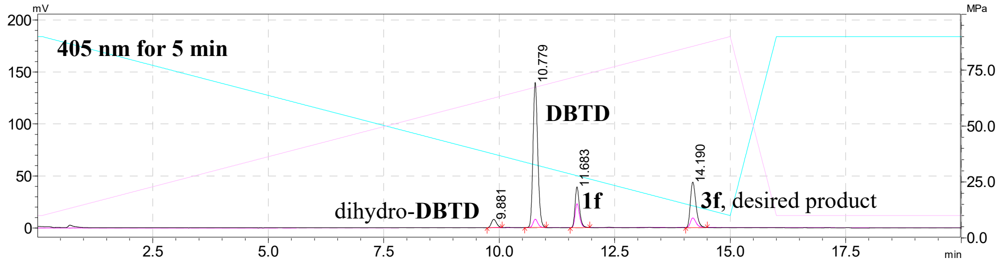

Peak No.	Time	Area	Height	%Area
1	9. 880	62441	8103	3.81
2	10. 776	1237286	173695	75.48
3	11.252	259482	39713	15.83

4	14.175	79939	10234	4.88

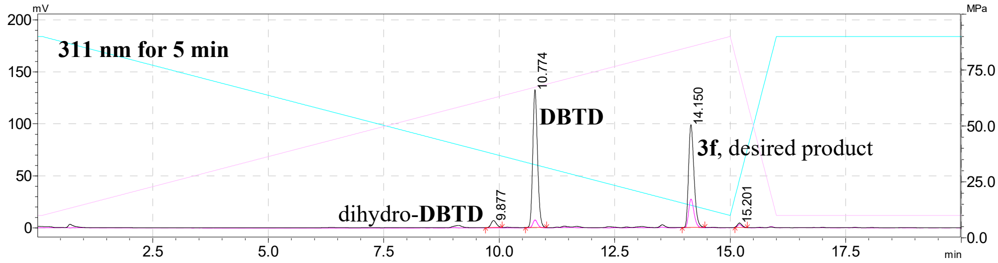

Peak No.	Time	Area	Height	%Area
1	9. 877	52083	6583	2.84
2	10. 774	946579	132045	51.66
3	14.150	805067	98539	43.94

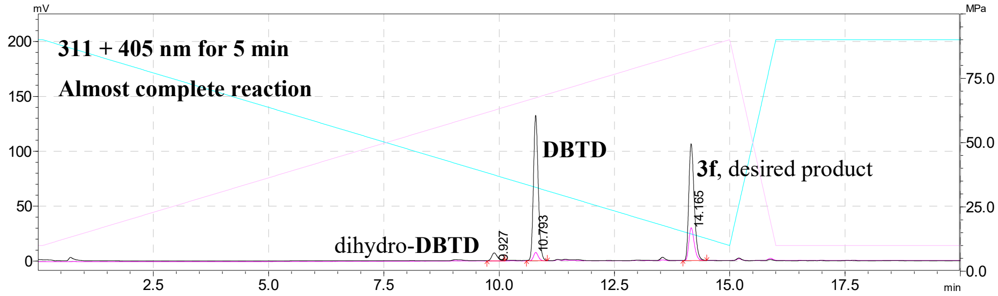

Peak No.	Time	Area	Height	%Area
1	9. 927	699	56	0.23
2	10. 793	54855	7610	18.39
3	14.165	242814	29788	81.38

Supplementary Figure 12. HPLC analysis for the photo-induced intermolecular cycloaddition reaction between DASyd **1f** and **DBTD** in ACN/H_2O (1:1, v/v): (**a**) HPLC trace of reaction mixture after 30s photo-irradiation with with 405 nm laser, 311 nm UV lamp and 311 + 405 nm combination, respectively; (**b**) HPLC trace of reaction mixture after 5 min photo-irradiation with 405 nm, 311 nm and 311 + 405 nm, respectively. The concentration of **1f** and **DBTD** used in the reaction were 10 µM and 50 µM, respectively. The conversion from **1f** to **3f** was calculated based on the absorbance at 254 nm, [NI = nitrile imine]. Dihydro-**DBTD** was the reduced product of **DBTD** for $C_{12}H_{11}N_2S^+$ 215.06 [M+H$^+$], found 215.03. The mass identification of **3f** was confirmed by LC-MS: MS (ESI) calcd. for **3f** $C_{29}H_{25}N_4O_3S^+$ 509.16 [M+H$^+$], found 508.82.

Sydnone **1g**:

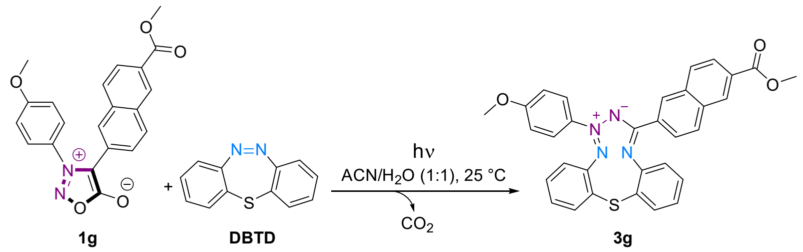

(**a**) For 30 s irradiation time

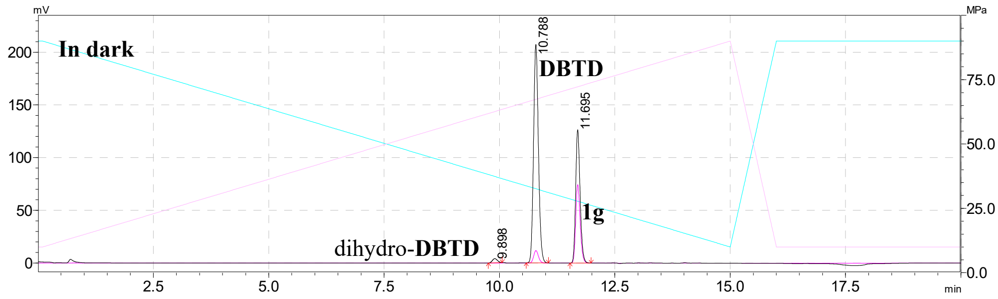

Peak No.	Time	Area	Height	%Area
1	9.898	29767	3933	1.25
2	10.788	1516971	206772	63.46
3	11.695	843523	125899	35.29

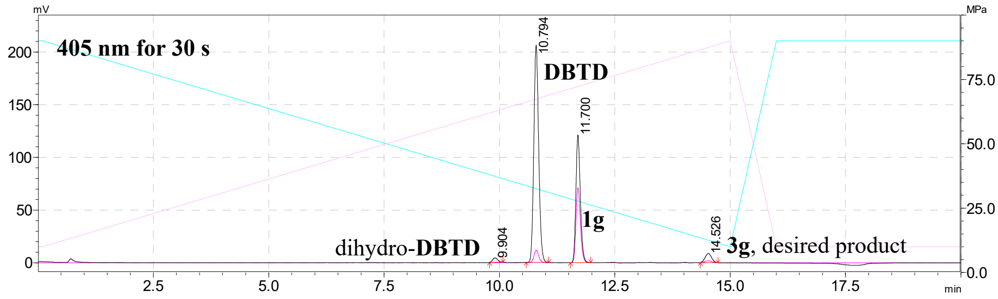

Peak No.	Time	Area	Height	%Area
1	9. 904	32245	4258	1.33
2	10. 794	1510150	205146	62.09
3	11. 700	806353	120858	33.15
4	14.526	83507	8637	3.43

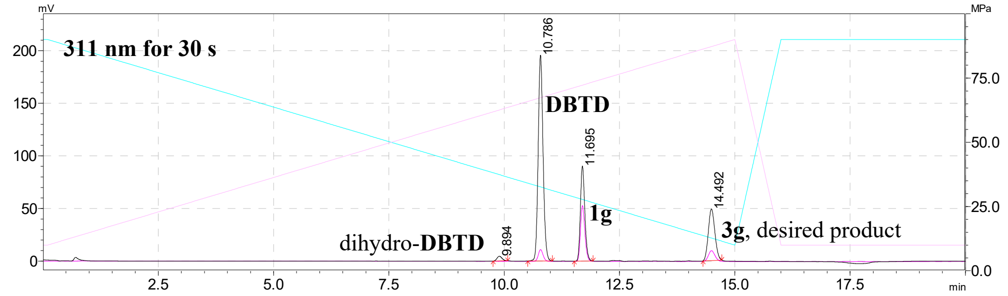

Peak No.	Time	Area	Height	%Area
1	9.894	34274	4494	1.34
2	10. 786	1436800	194807	56.25
3	11. 695	600641	89932	23.51
4	14.302	482841	48974	18.90

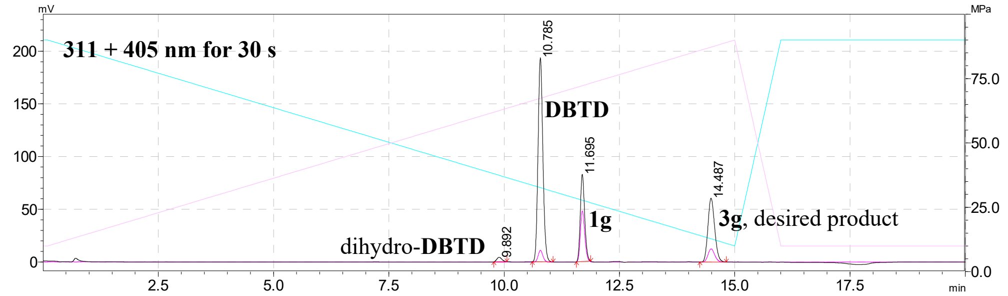

Peak No.	Time	Area	Height	%Area
1	9. 892	31670	4197	1.21
2	10. 785	1414792	192911	54.07
3	11. 695	554474	82755	21.19
4	14. 487	615886	60391	23.54

(**b**) For 5 min irradiation time

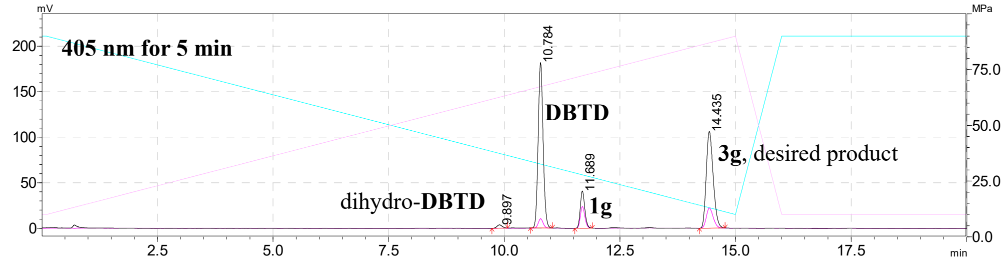

Peak No.	Time	Area	Height	%Area
1	9. 897	27944	3571	1.01
2	10. 784	1349208	181183	48.84
3	11. 689	275271	40769	9.96
4	14. 435	1110328	105911	40.19

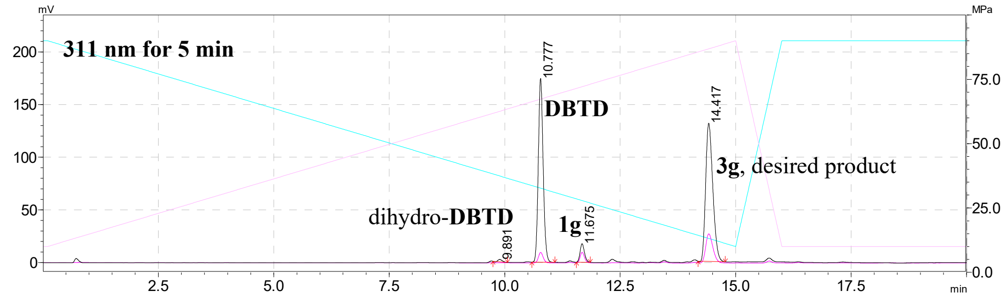

Peak No.	Time	Area	Height	%Area
1	9.891	12009	2222	0.43
2	10.777	1270696	173853	45.27
3	11.675	115802	17509	4.13
4	14.417	1438388	131159	50.18

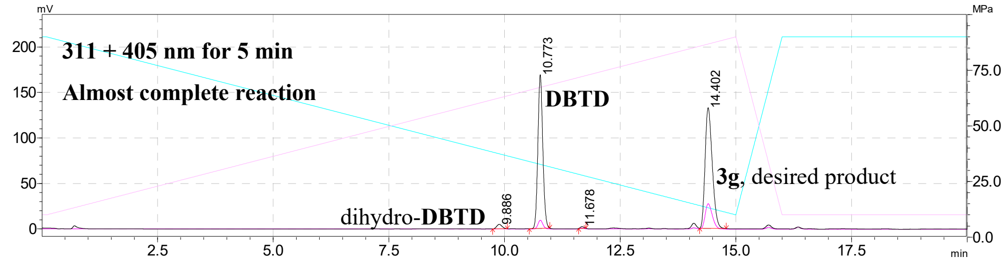

Peak No.	Time	Area	Height	%Area
1	9. 886	699	56	1.45
2	10. 773	54855	7610	45.79
3	14. 402	1488235	132351	52.38

Supplementary Figure 13. HPLC analysis for the photo-induced intermolecular cycloaddition reaction between DASyd **1g** and **DBTD** in ACN/H_2O (1:1, v/v): (**a**) HPLC trace of reaction mixture after 30s photo-irradiation with the 405 nm laser, the 311 nm UV lamp or the 311 + 405 nm combination, respectively; (**b**) HPLC trace of reaction mixture after 5min photo-irradiation with 405 nm, 311 nm and 311 + 405 nm, respectively. The concentration of **1g** and **DBTD** used in the reaction were 10 μM and 50 μM, respectively. The conversion from **1g** to **3g** was calculated based on the absorbance at 254 nm, [NI = nitrile imine]. Dihydro-**DBTD** was the reduced product of **DBTD** for $C_{12}H_{11}N_2S^+$ 215.06 [M+H$^+$], found 215.03. The mass identification of **3g** was confirmed by LC-MS: MS (ESI) calcd. for **3g** $C_{32}H_{25}N_4O_3S^+$ 545.16 [M+H+], found 544.72.

Sydnone **1h**:

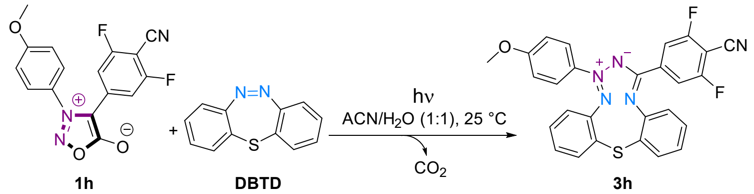

(**a**) For 30 s irradiation time

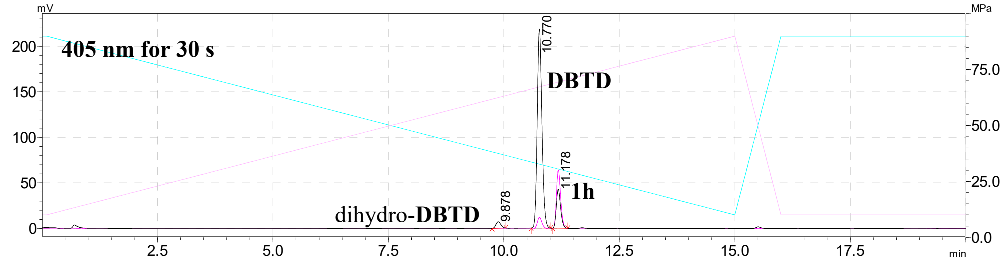

Peak No.	Time	Area	Height	%Area
1	9. 878	53311	7105	2.84
2	10. 770	1543750	217335	82.35
3	11.178	277685	42670	14.81

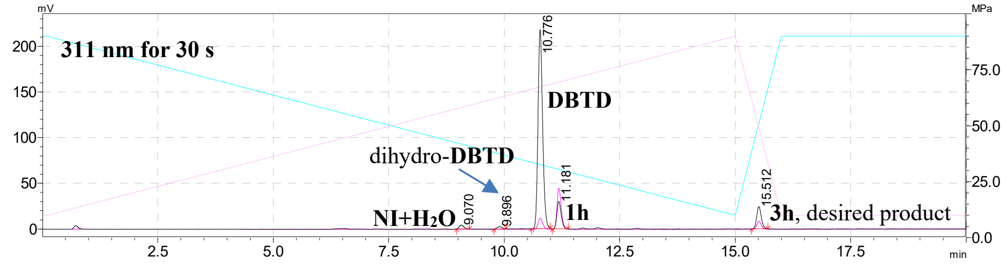

Peak No.	Time	Area	Height	%Area
1	9. 896	17769	2461	0.92
2	10. 776	1517670	216916	78.88
3	11.181	192774	29831	10.02
4	15.512	166849	24017	8.67

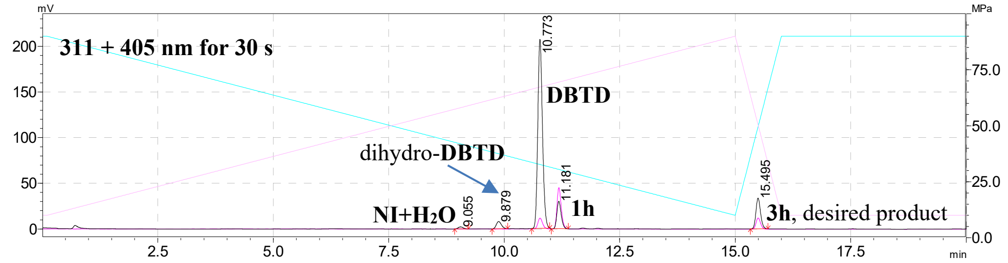

Peak No.	Time	Area	Height	%Area
1	9. 879	61967	7992	3.16
2	10. 773	1455280	206427	74.26
3	11.181	193352	29924	9.87
4	15.495	232650	33463	11.87

(**b**) For 5 min irradiation time

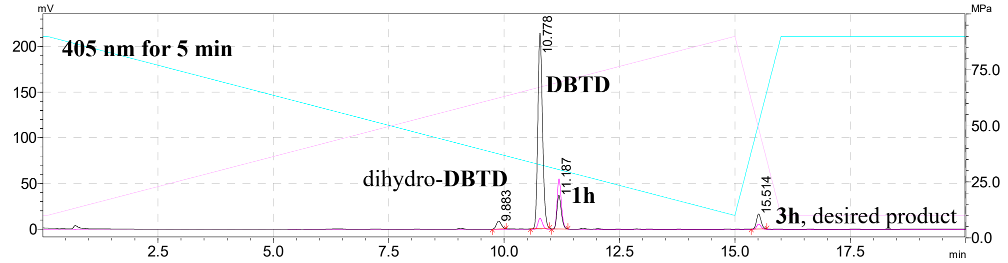

Peak No.	Time	Area	Height	%Area
1	9. 883	64106	8519	3.34
2	10. 778	1502238	213055	78.37
3	11.187	237083	36657	12.37
4	15.514	113406	16473	5.92

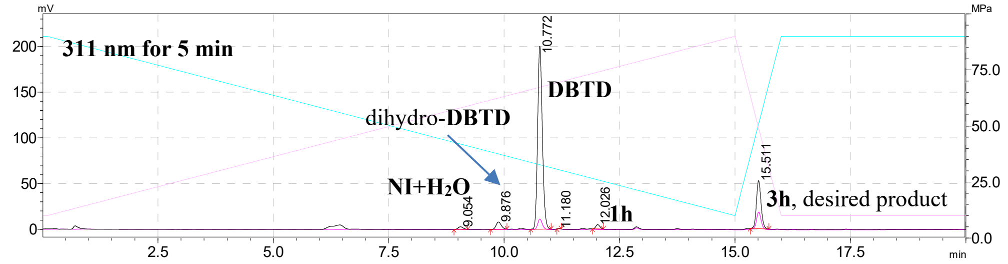

Peak No.	Time	Area	Height	%Area
1	9. 876	60837	7897	3.23
2	10. 772	1405045	199668	74.67
3	12.026	27831	4599	1.48
4	15.511	367250	52327	19.52

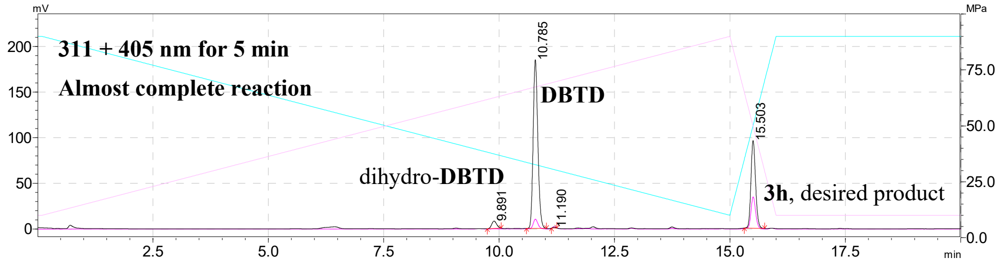

Peak No.	Time	Area	Height	%Area
1	9. 891	60192	8044	2.95
2	10. 785	1308311	184286	64.21
3	15.503	669213	95539	32.84

Supplementary Figure 14. HPLC analysis for the photo-induced intermolecular cycloaddition reaction between DASyd **1h** and **DBTD** in ACN/H_2O (1:1, v/v): (**a**) HPLC trace of reaction mixture after 30s photo-irradiation with 405 nm laser, 311 nm UV lamp and 311 + 405 nm combination, respectively; (**b**) HPLC trace of reaction mixture after 5 min photo-irradiation with 405 nm, 311 nm and 311 + 405 nm, respectively. The concentration of **1h** and **DBTD** used in the reaction were 10 µM and 50 µM, respectively. The conversion from **1h** to **3h** was calculated based on the absorbance at 254 nm, [NI = nitrile imine]. Dihydro-**DBTD** was the reduced product of **DBTD** for $C_{12}H_{11}N_2S^+$ 215.06 [M+H$^+$], found 215.03. The mass identification of **3h** was confirmed by LC-MS: MS (ESI) calcd. for **3h** $C_{27}H_{18}F_2N_5OS^+$ 498.12 [M+H$^+$], found 497.60.

Sydnone **1i**:

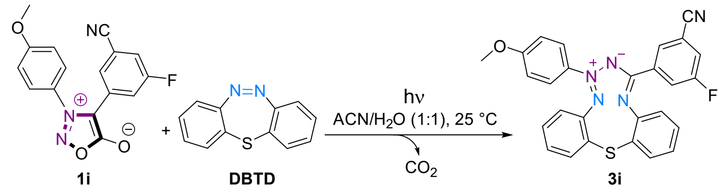

(**a**) For 30 s irradiation time

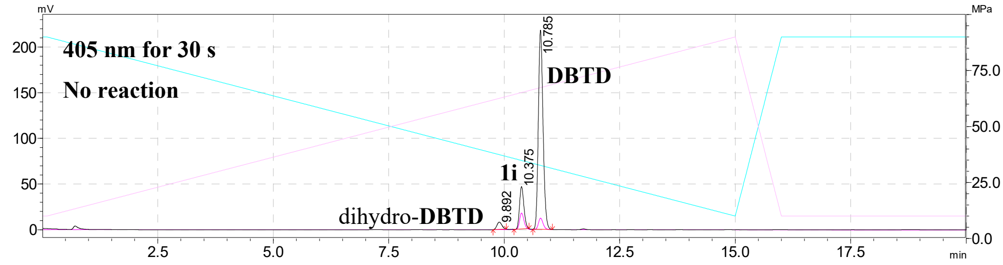

Peak No.	Time	Area	Height	%Area
1	9. 892	59558	7961	3.12
2	10. 375	303140	46279	15.90
3	10.785	1543635	217267	80.98

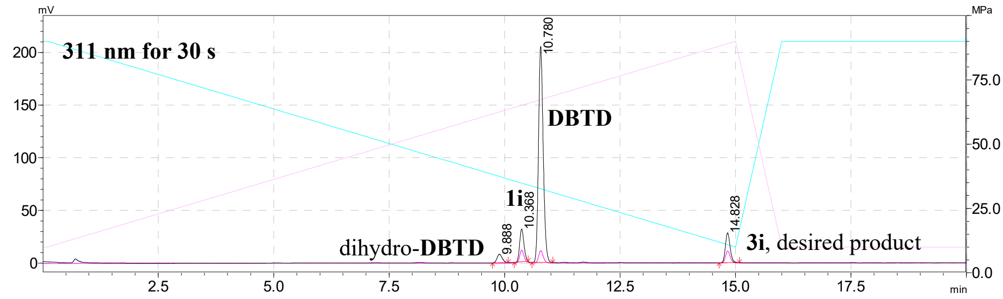

Peak No.	Time	Area	Height	%Area
1	9. 888	62578	8043	3.26
2	10. 368	199897	31000	10.42
3	10.780	1456308	204797	75.88
4	14.828	200376	28151	10.44

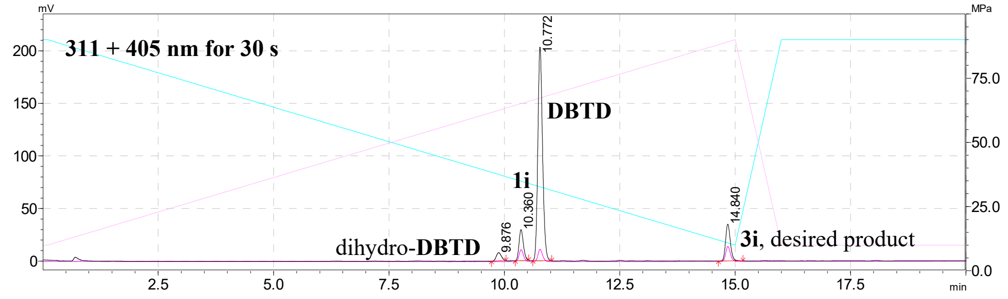

Peak No.	Time	Area	Height	%Area
1	9.876	59187	7782	3.05
2	10.360	193367	29276	9.97
3	10.772	1437018	202685	74.08
4	14.840	250329	34957	12.90

(**b**) For 5 min irradiation time

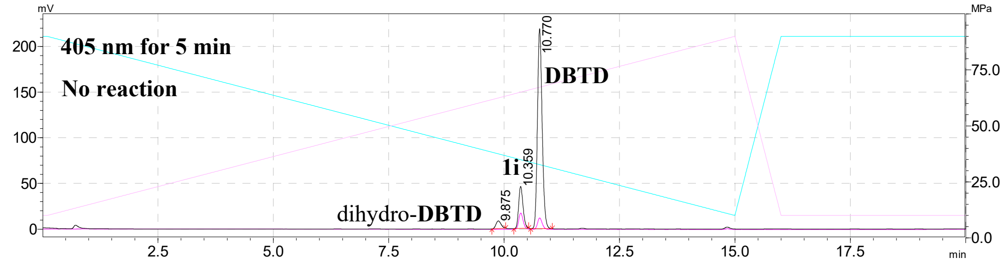

Peak No.	Time	Area	Height	%Area
1	9. 875	64318	8418	3.36
2	10. 359	303166	45948	15.84
3	10.770	1546370	217903	80.80

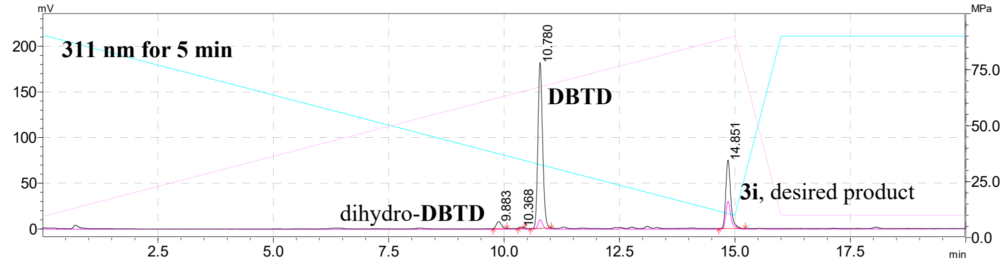

Peak No.	Time	Area	Height	%Area
1	9. 883	58960	7649	3.09
2	10. 780	1284165	181143	67.35
3	14.851	553577	74729	29.03

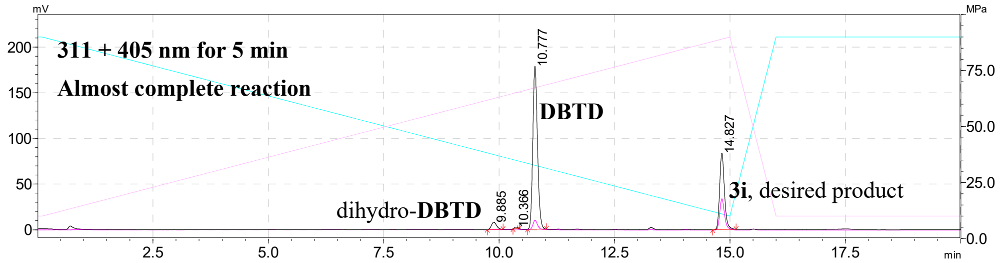

Peak No.	Time	Area	Height	%Area
1	9. 885	62221	7953	3.22
2	10. 777	1257523	177523	65.02
3	14.827	599309	83384	30.99

Supplementary Figure 15. HPLC analysis for the photo-induced intermolecular cycloaddition reaction between DASyd **1i** and **DBTD** in ACN/H_2O (1:1, v/v): (**a**) HPLC trace of reaction mixture after 30s photo-irradiation with 405 nm laser, 311 nm UV lamp and 311 + 405 nm combination, respectively; (**b**) HPLC trace of reaction mixture after 5min photo-irradiation with 405 nm, 311 nm and 311 + 405 nm, respectively. The concentration of **1i** and **DBTD** used in the reaction were 10 μM and 50 μM, respectively. The conversion from **1i** to **3i** was calculated based on the absorbance at 254 nm, [NI = nitrile imine]. Dihydro-**DBTD** was the reduced product of **DBTD** for $C_{12}H_{11}N_2S^+$ 215.06 [M+H$^+$], found 215.03. The mass identification of **3i** was confirmed by LC-MS: MS (ESI) calcd. for **3i** $C_{27}H_{19}FN_5OS^+$ 480.13 [M+H$^+$], found 479.63.

Sydnone **1j**:

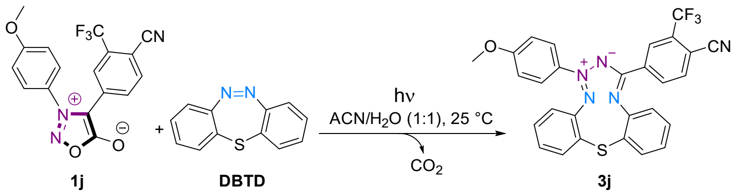

(**a**) For 30 s irradiation time

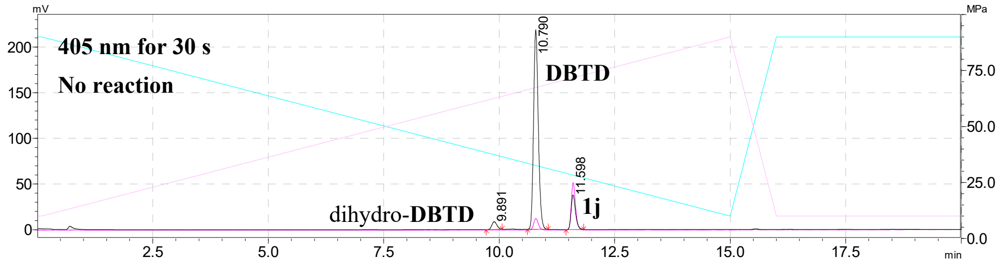

Peak No.	Time	Area	Height	%Area
1	9. 891	64520	8477	3.45
2	10. 790	1555500	218203	83.27
3	11. 598	247985	37788	13.28

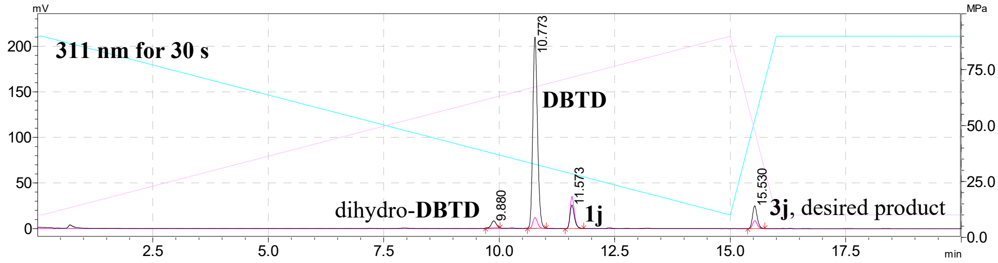

Peak No.	Time	Area	Height	%Area
1	9. 880	57529	7639	3.06
2	10. 773	1482770	209462	78.75
3	11. 573	169811	25699	90.2
4	15. 530	172666	24806	9.17

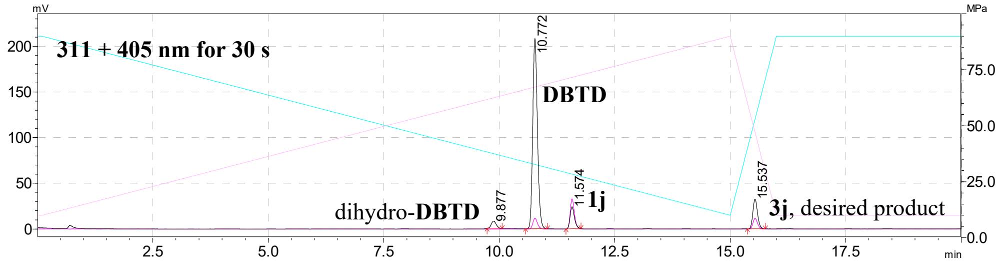

Peak No.	Time	Area	Height	%Area
1	9. 877	60232	7954	3.14
2	10. 772	1474270	207699	76.91
3	11. 574	155995	24042	8.14
4	15. 537	226379	32443	11.81

(**b**) For 5 min irradiation time

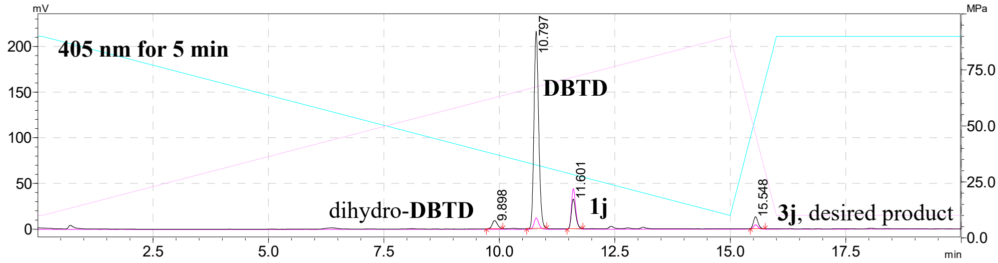

Peak No.	Time	Area	Height	%Area
1	9. 898	69137	8874	3.64
2	10. 797	1530194	215605	80.62
3	11. 601	210221	32327	11.08
4	15. 548	88546	13000	4.67

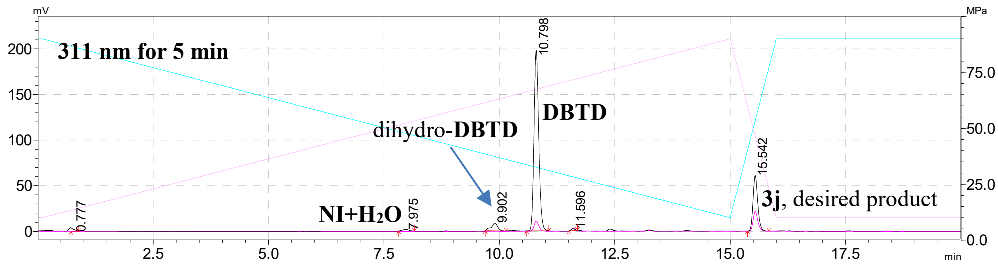

Peak No.	Time	Area	Height	%Area
1	7.975	16859	1703	0.87
2	9. 902	84455	8572	4.36
3	10. 798	1406643	197974	72.62
4	15. 542	429107	60623	22.15

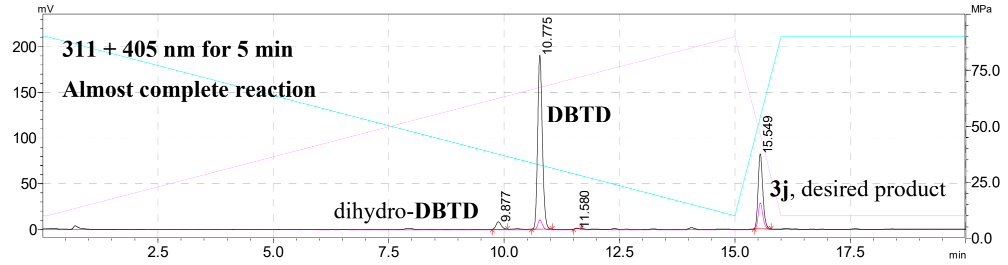

Peak No.	Time	Area	Height	%Area
1	9. 877	62494	8047	3.14
2	10. 775	1348808	190329	67.87
3	15. 549	576159	81916	28.99

Supplementary Figure 16. HPLC analysis for the photo-induced intermolecular cycloaddition reaction between DASyd **1j** and **DBTD** in ACN/H_2O (1:1, v/v): (**a**) HPLC trace of reaction mixture after 30s photo-irradiation with 405 nm laser, 311 nm UV lamp and 311 + 405 nm combination, respectively; (**b**) HPLC trace of reaction mixture after 5 min photo-irradiation with 405 nm, 311 nm and 311 + 405 nm, respectively. The concentration of **1j** and **DBTD** used in the reaction were 10 μM and 50 μM, respectively. The conversion from **1j** to **3j** was calculated based on the absorbance at 254 nm, [NI = nitrile imine]. Dihydro-**DBTD** was the reduced product of **DBTD** for $C_{12}H_{11}N_2S^+$ 215.06 [M+H$^+$], found 215.03. The mass identification of **3j** was confirmed by LC-MS: MS (ESI) calcd. for **3j** $C_{28}H_{19}F_3N_5OS^+$ 530.13 [M+H$^+$], found 529.84.

Photophysical properties and stability investigation of the MAI cycloadduct in water containing solution phase and its resistance test against nucleophile additions by several nucleophilic reagents and GSH.

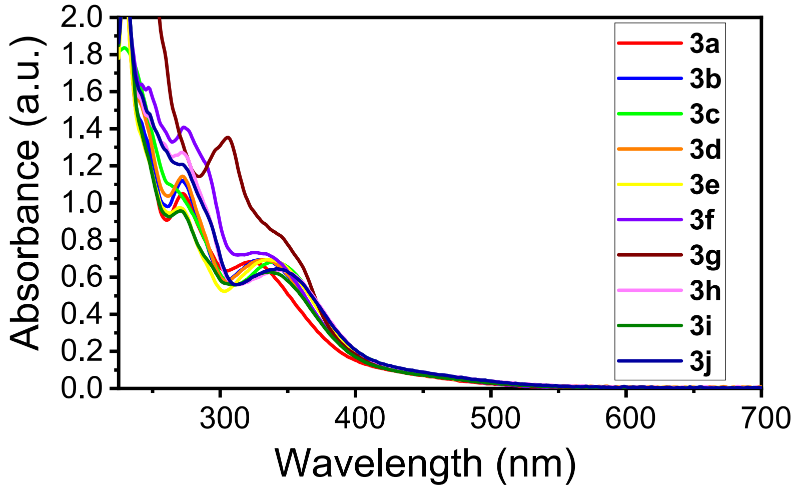

Supplementary Figure 17. UV-vis spectra of **3a-3j** at 50 µM in ACN/H_2O (1:1, v/v).

Monitoring the background thermo-cycloaddition reactions of 1d or 1g with (Z)-DBTD in dark at 25 ºC by HPLC-MS analysis.

After searching the value of the mass-to-charge ratio of potential adducts from the corresponding thermal cycloaddition reaction throughout the real-time mass spectra and the HPLC traces, it is discovered that there is no any adduct detected by LC-MS analysis after mixing the DASyd and (*Z*)-**DBTD** reactants within 116 hours at the concentration of 1000 μM. There is also no any new peak detected in the HPLC traces as well which are shown below. As a result, the DASyds (**1d** and **1g**) reacted extremely slow with 1 eq. ring-strained (*Z*)-**DBTD** at 25 ºC in ACN/H_2O.

(a)

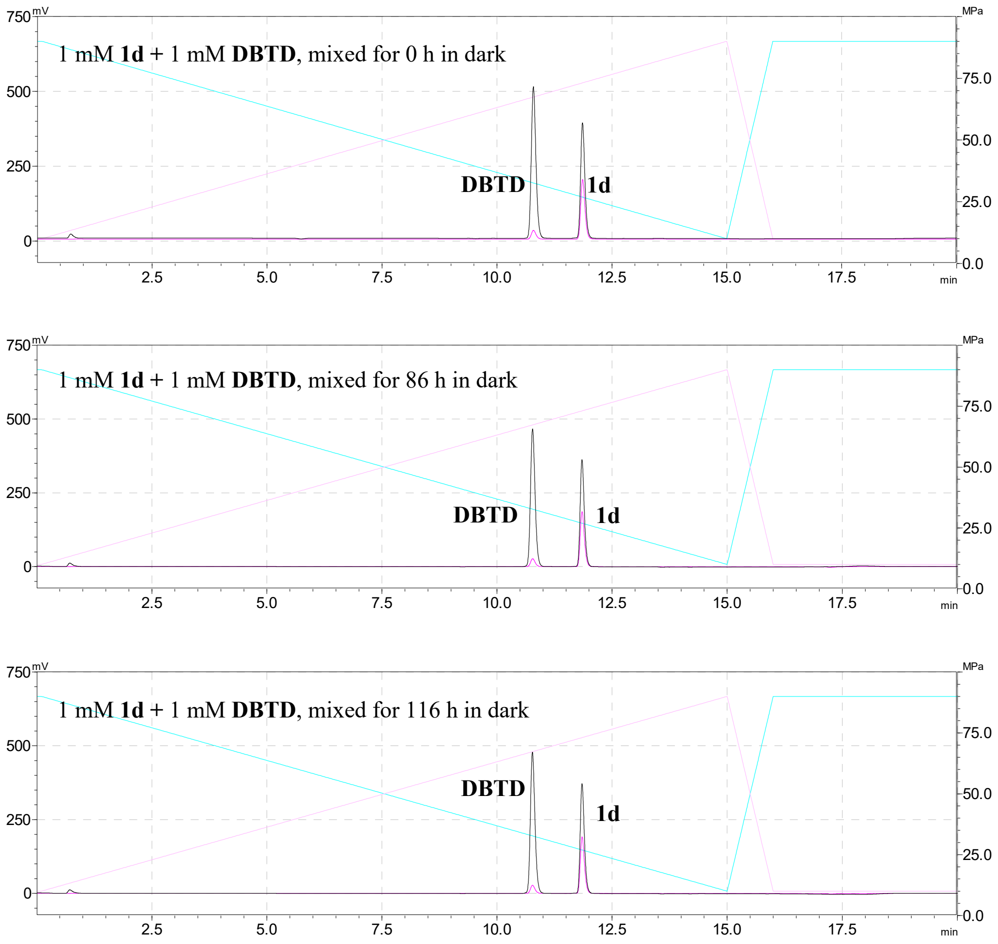

(**b**)

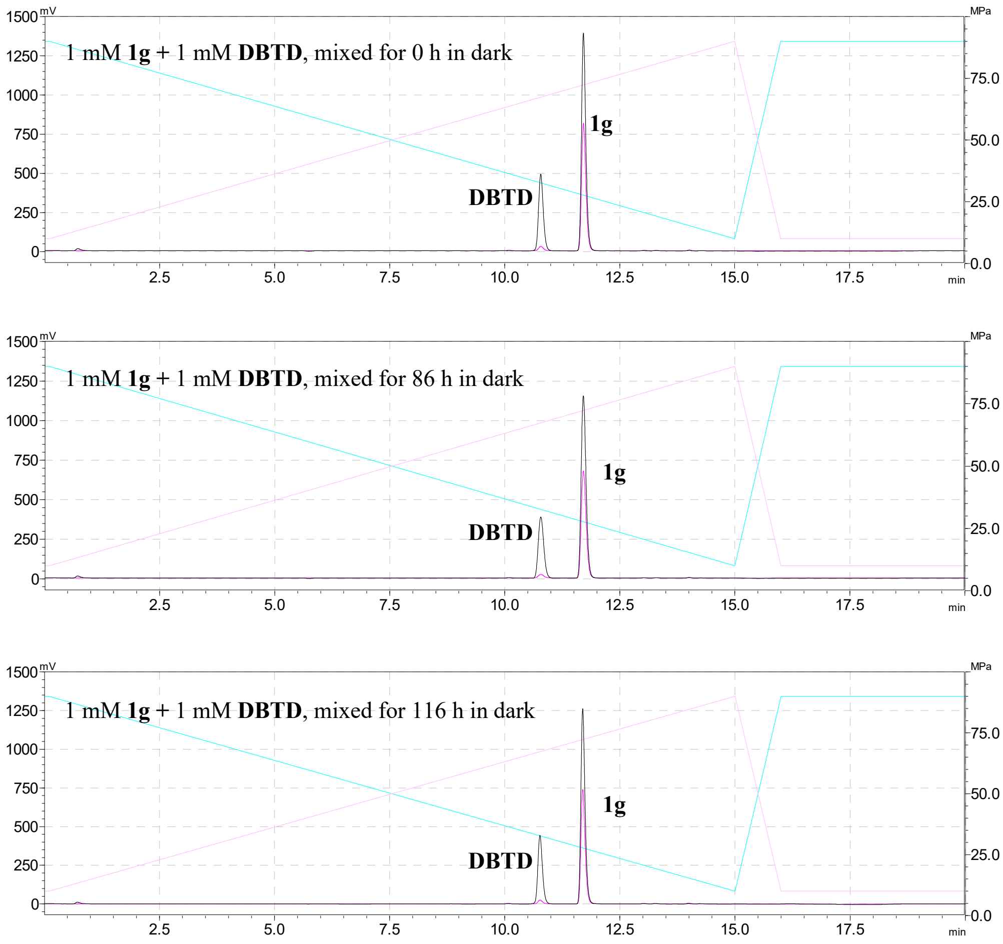

Supplementary Figure 18. LC-MS traces for exploration of potential cycloaddition reaction of the (*Z*)-**DBTD** with (**a**) **1d** for 0 h, 86 h and 116 h in the dark environment; (**b**) **1g** for 0 h, 86 h and 116 h in the dark environment.

Stability test of **MAI** against nucleophiles

(**a**) Stability test of **3c** in solution phase

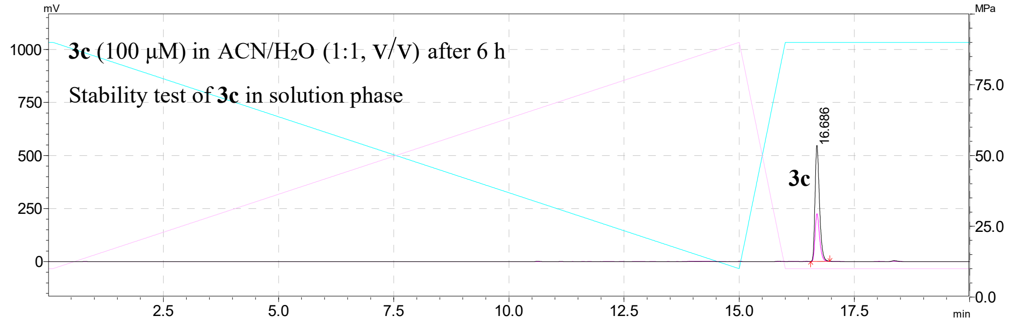

Peak No.	Time	Area	Height	%Area
1	16.686	3663141	544013	100

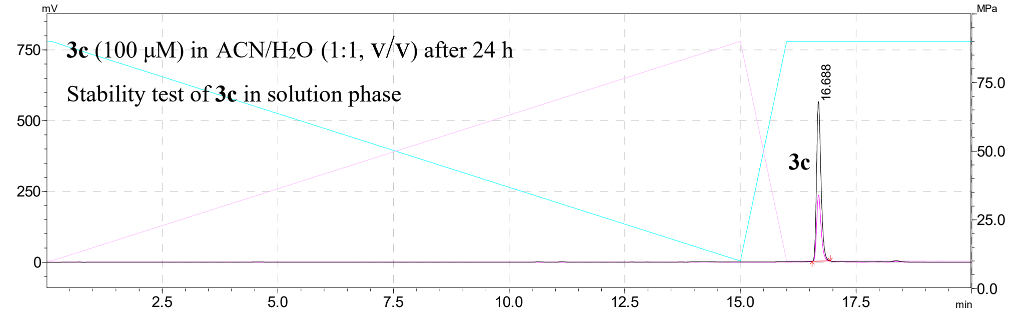

Peak No.	Time	Area	Height	%Area
1	16.688	3762152	562147	100

(**b**) Stability test of **3c** against primary amine addition

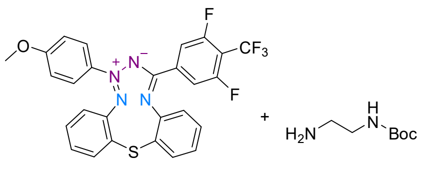

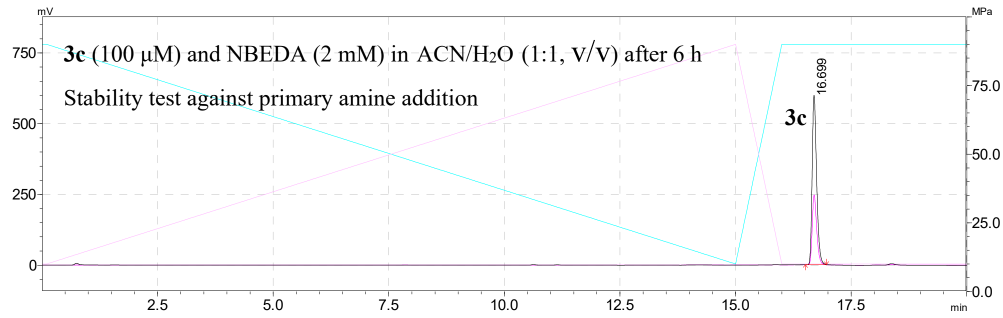

Peak No.	Time	Area	Height	%Area
1	16.699	4017918	597485	100

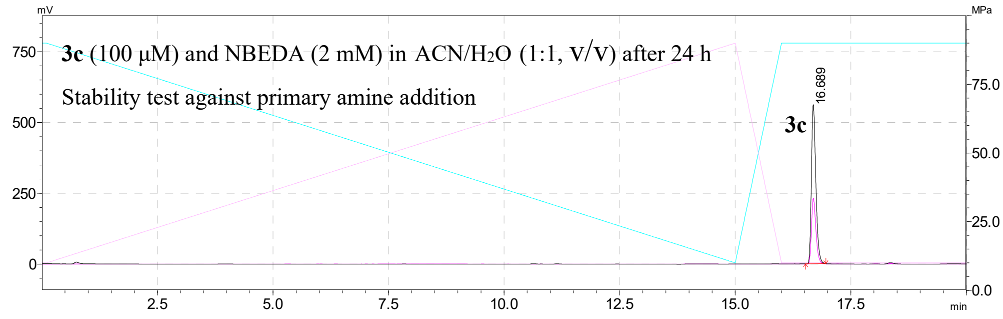

Peak No.	Time	Area	Height	%Area
1	16.689	3756693	560894	100

(**c**) Stability test of **3c** against hydroxy group addition

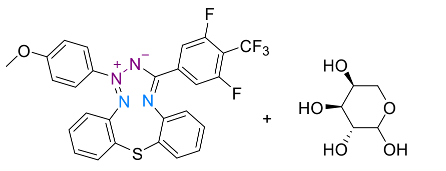

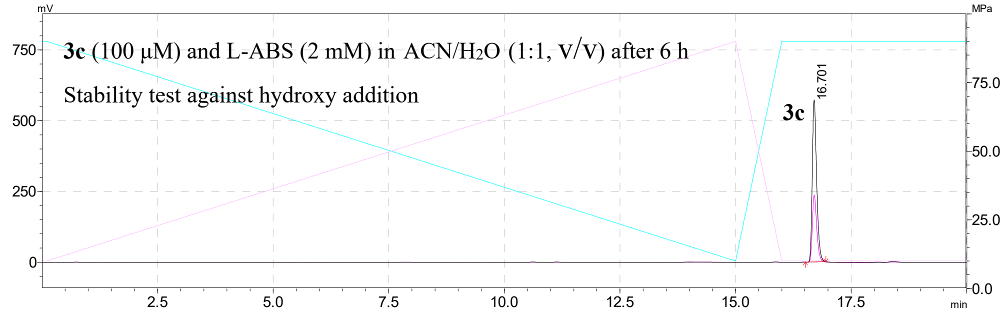

Peak No.	Time	Area	Height	%Area
1	16.701	3761188	570537	100

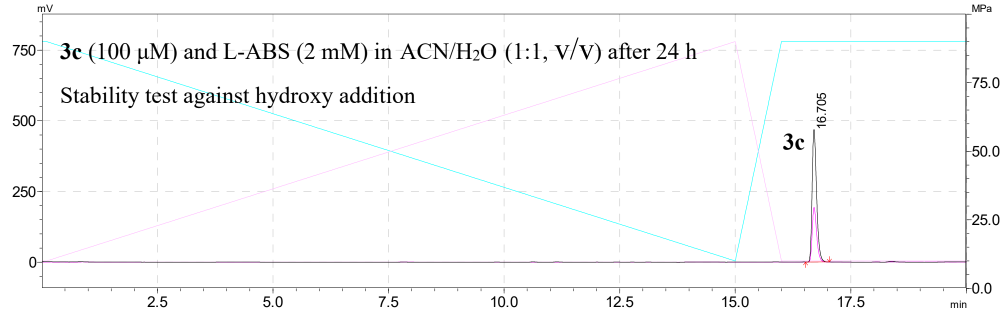

Peak No.	Time	Area	Height	%Area
1	16.705	3155575	467469	100

(**d**) Stability test of **3c** against thiol addition (GSH)

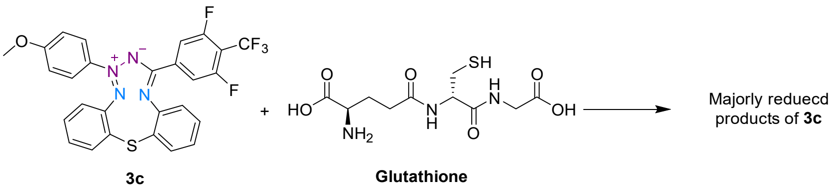

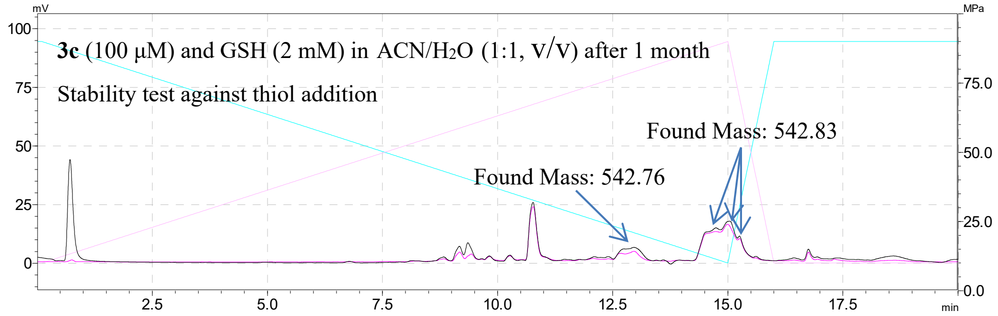

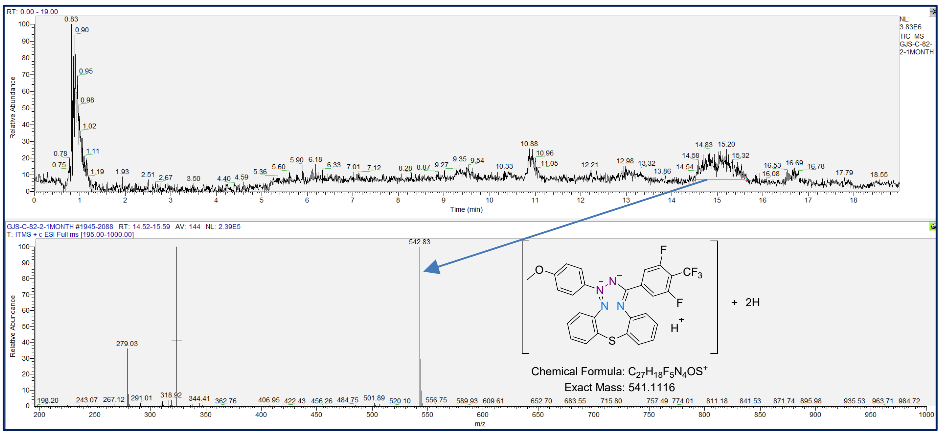

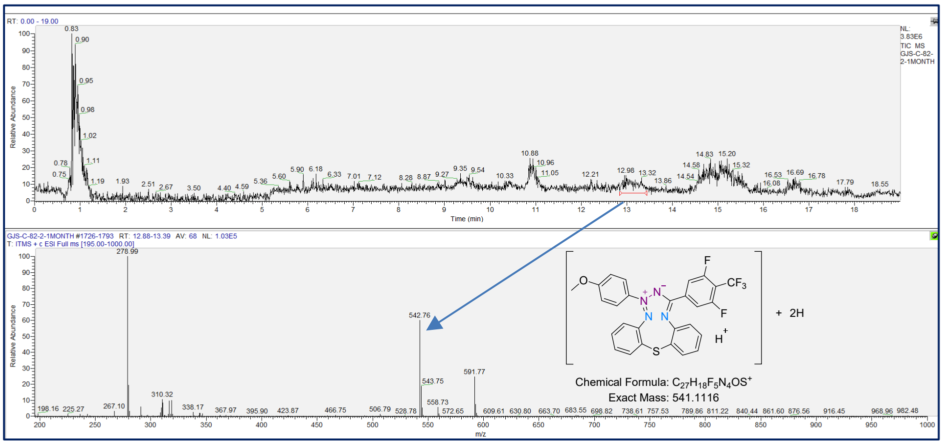

Supplementary Figure 19. HPLC analysis of the stability of **3c** (100 μM in ACN/H_2O = 1:1) toward various nucleophilic reagents at room temperature: (**a**) **3c** in ACN/H_2O (1:1, v/v) for 6 h or 24 h; (**b**) **3c** and *N*-Boc-ethylenediamine (NBEDA, 20 eq. 2 mM) in ACN/H_2O (1:1, v/v) for 6 h or 24 h; (**c**) **3c** and L-arabinose (L-ABS, 20 eq. 2 mM) in ACN/H_2O (1:1, v/v) for 6 h or 24 h. (**d**) **3c** and glutathione (GSH, 20 eq. 2 mM) in ACN/H_2O (1:1, v/v) reacted for 1 month. The products of the reduced **3c** were found in the HPLC trace with the mass/charge ratios, 542.76 and 542.83 found.

Stability test of **3c** against dipolarophiles

(**a**) Stability test of **3c** in solution phase against TCO

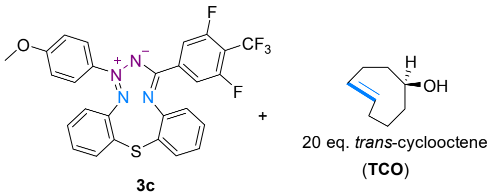

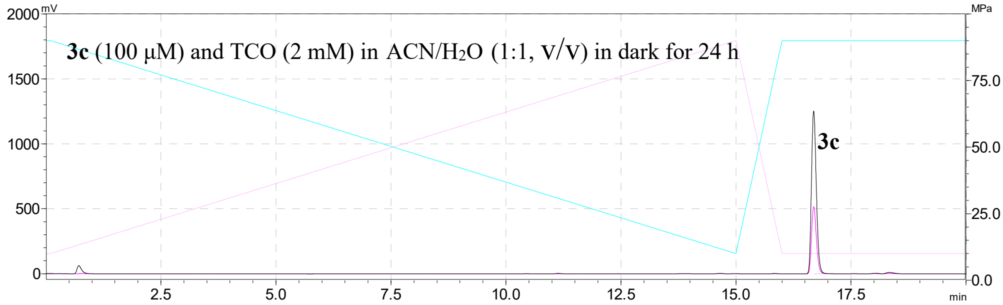

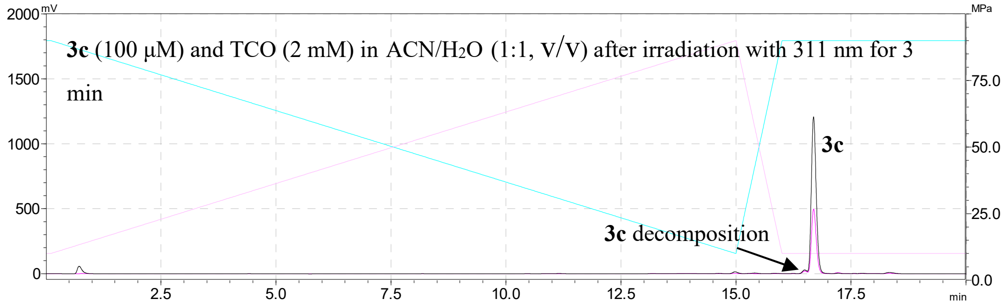

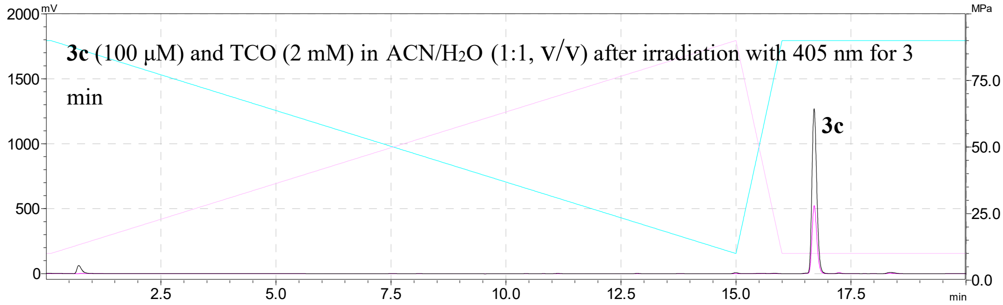

(**b**) Stability test of **3c** in solution phase against electron deficient alkene

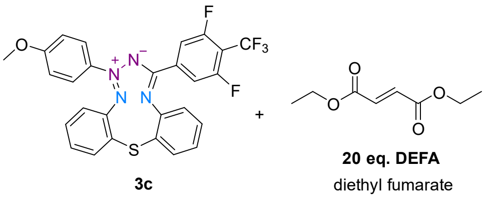

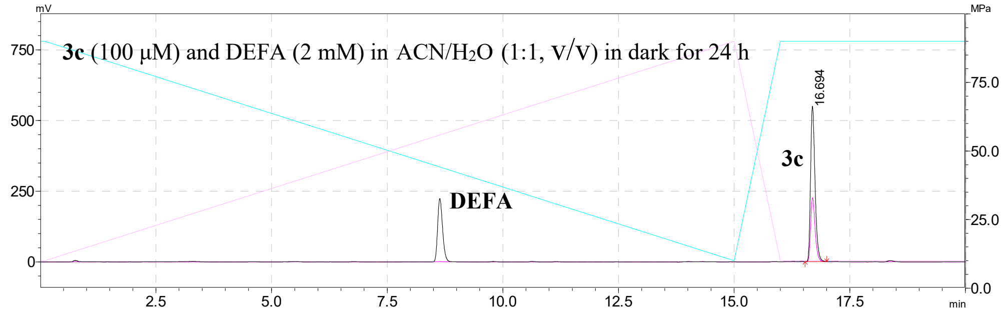

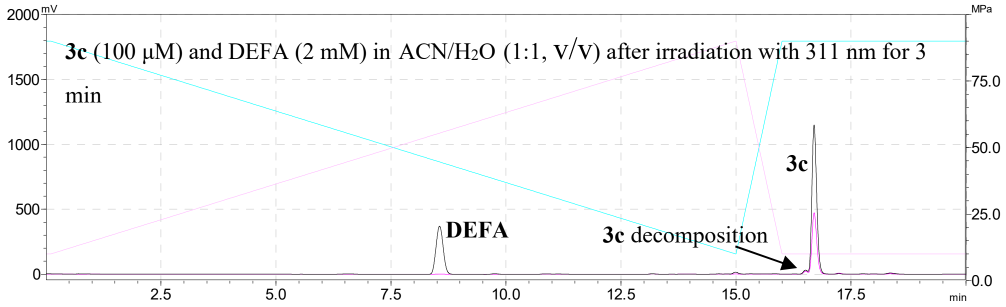

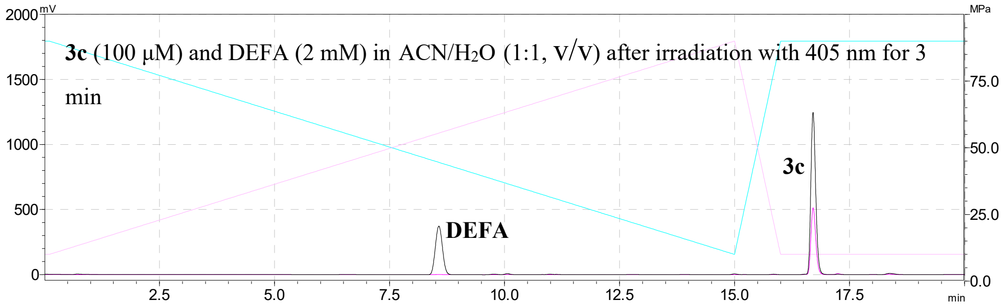

(**c**) Stability test of **3i** in solution phase against BCN

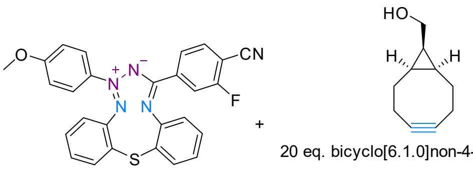

3i

20 eq. bicyclo[6.1.0]non-4-yn-9-ylmethanol (**BCN**)

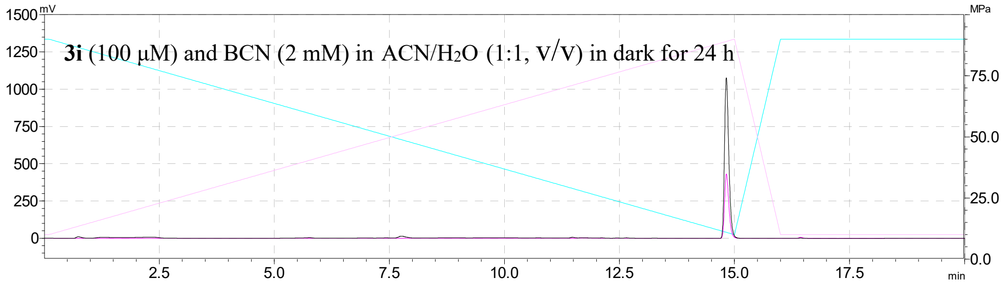

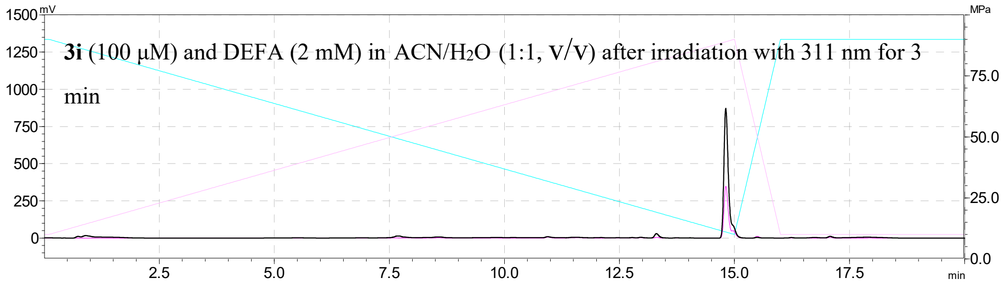

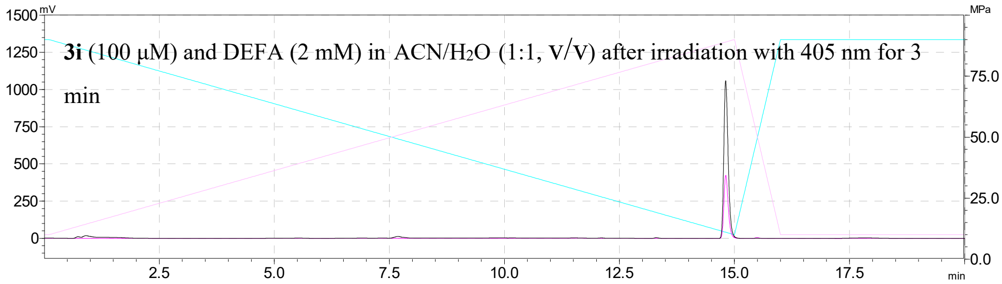

Supplementary Figure 20. HPLC analysis of the stability of the **3c** (100 µM in ACN/H_2O = 1:1) toward alkenes at room temperature: (**a**) **3c** and TCO (20 eq. 2 mM) in ACN/H_2O (1:1, v/v) for 24h and under irradiation with 311 or 405 nm for 3 min; (**b**) **3c** and diethyl fumarate (DEFA, 20 eq. 2 mM) in ACN/H_2O (1:1, v/v) for 24 h and under irradiation with 311 or 405 nm for 3 min; (**c**) **3i** and BCN (20 eq. 2 mM) in ACN/H_2O (1:1, v/v) for 24 h and under irradiation with 311 or 405 nm for 3 min.

Stability test of **3i** under the irradiation of 311 + 405 nm or under ambient light

(a)

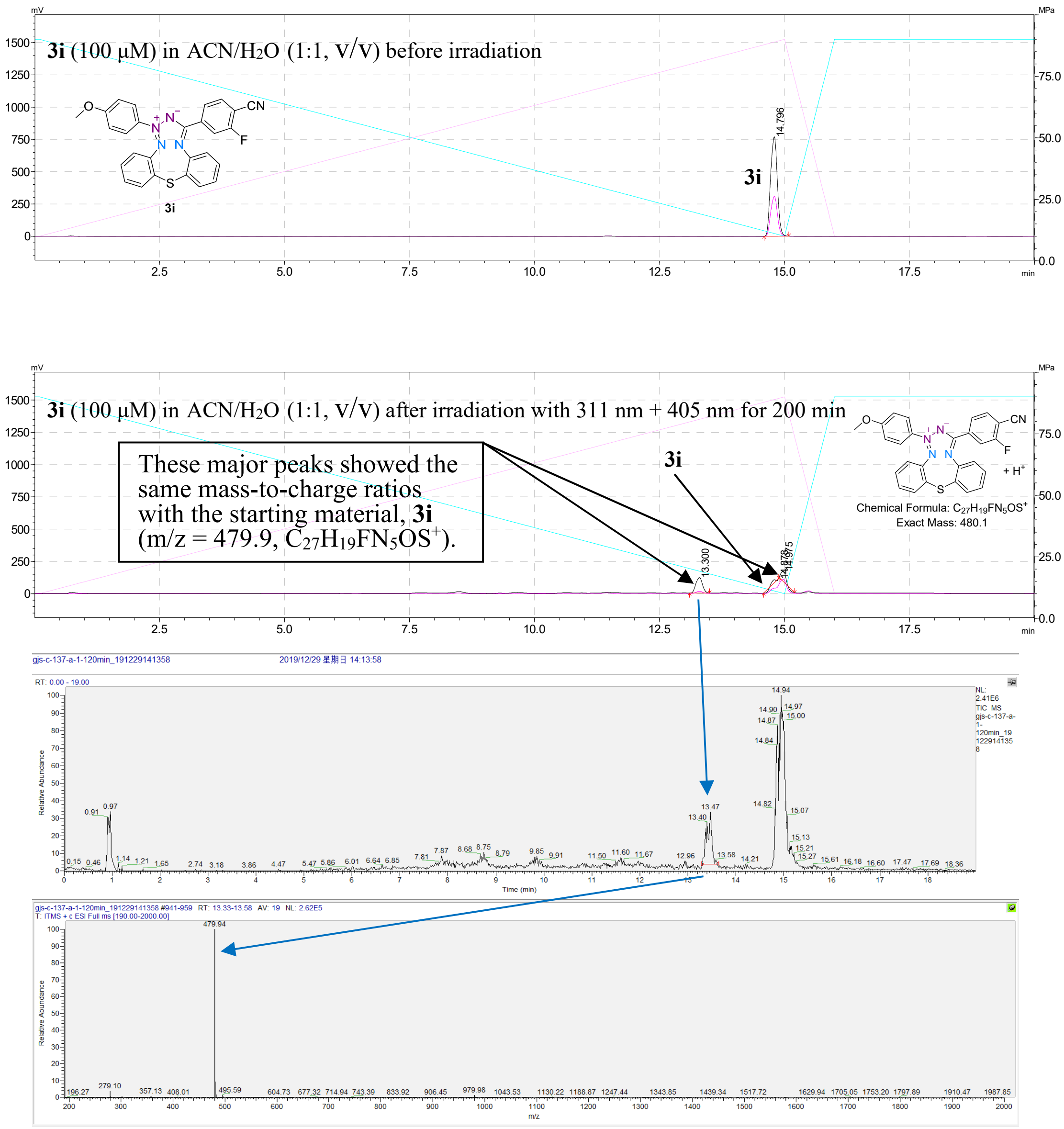

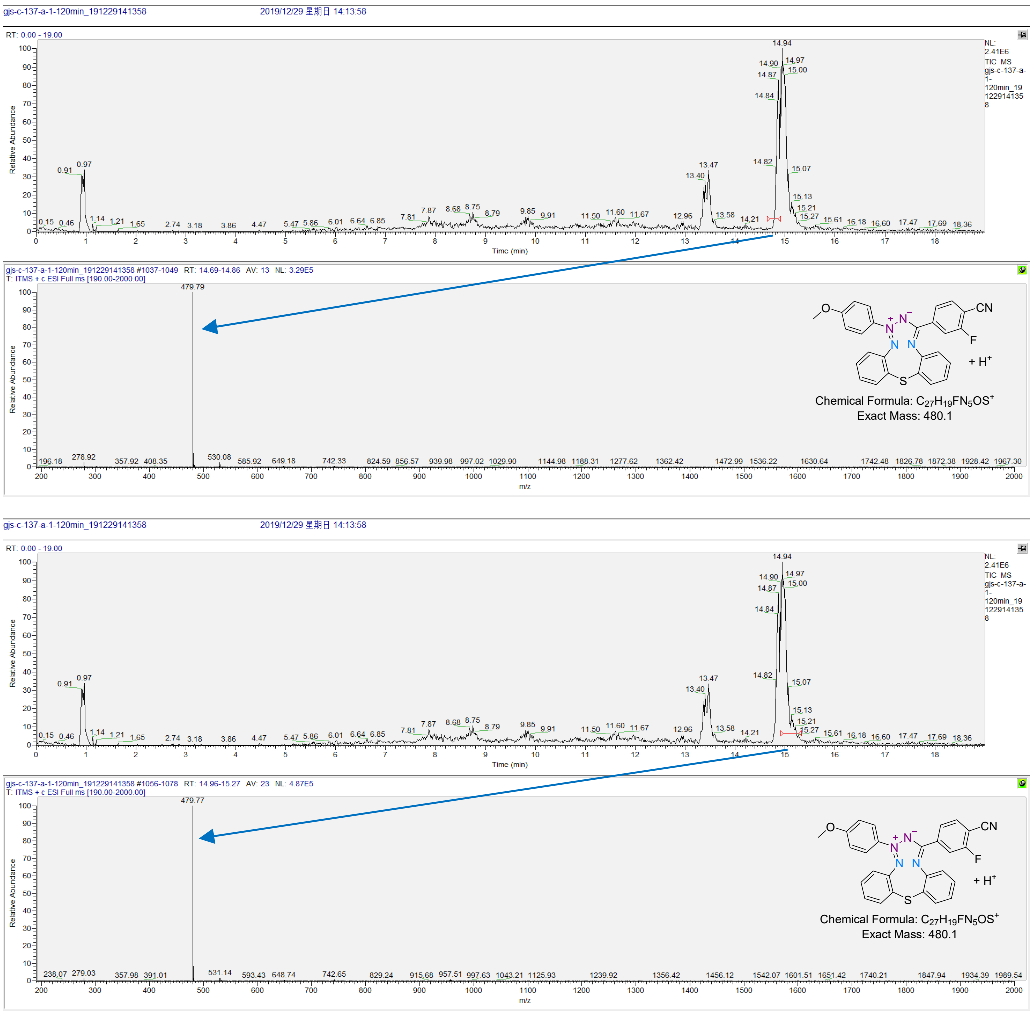

gjs-c-137-a-1-120min_191229141358
2019/12/29 星期日 14:13:58
Relative Abundance
Time (min)
m/z
Chemical Formula: C27H19FN5OS+
Exact Mass: 480.1
+ H+
CN
F
S
gjs-c-137-a-1-120min_191229141358
2019/12/29 星期日 14:13:58
Relative Abundance
Time (min)
m/z
Chemical Formula: C27H19FN5OS+
Exact Mass: 480.1
+ H+
CN
F
S

(b)

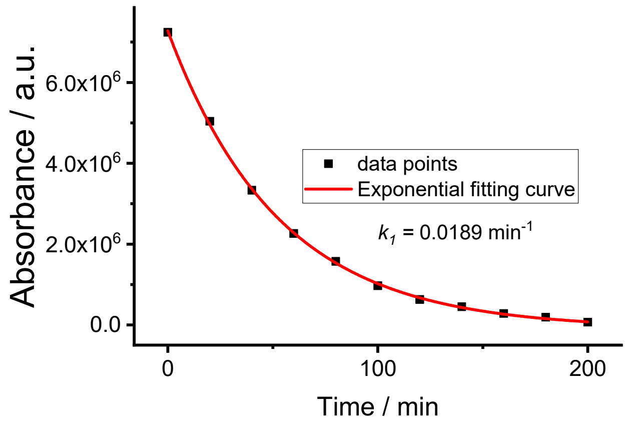

Absorbance / a.u.
Time / min
data points
Exponential fitting curve
k1 = 0.0189 min-1
6.0x10^6
4.0x10^6
2.0x10^6
0.0
0
100
200

(c)

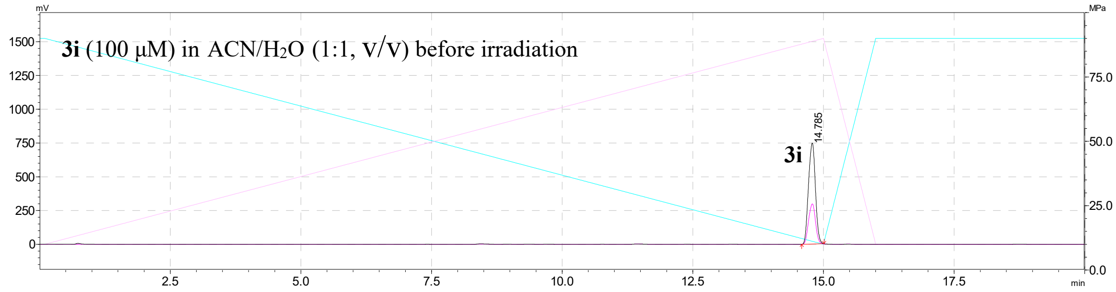

Peak No.	Time	Area	Height	%Area
1	14.785	6906386	765600	100

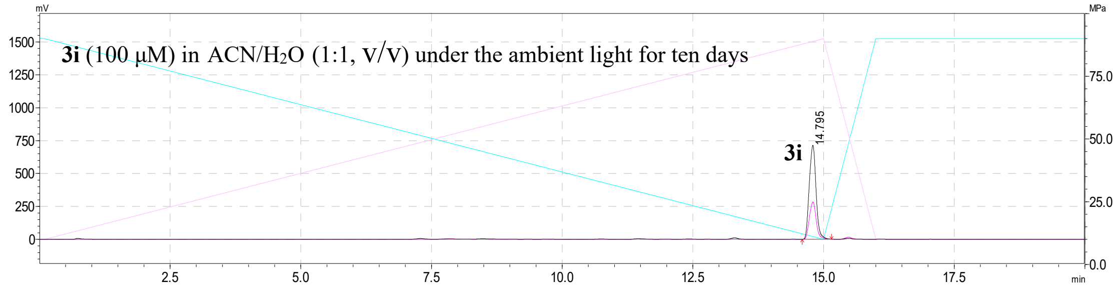

Peak No.	Time	Area	Height	%Area
1	14.795	6432646	712520	100

(d)

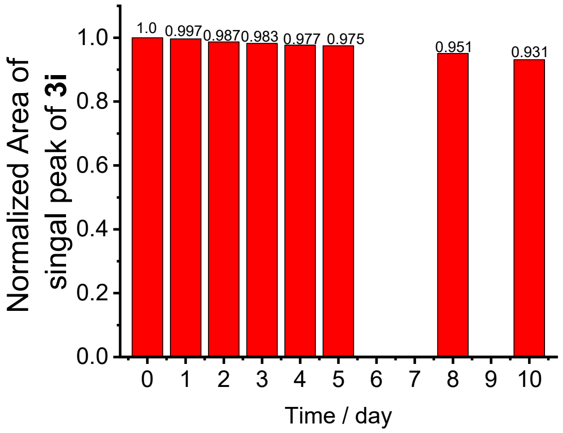

Supplementary Figure 21. HPLC-MS analysis for studying the photo-stability of **3i** (100 μM in ACN/H_2O = 1:1) at room temperature: (**a**) HPLC traces for **3i** in ACN/H_2O (1:1, v/v) before and after irradiation with 311 + 405 nm for 200 min with the mass spectra of denoted peaks; (**b**) Plots for the photo-decay rate of **3i** under irradiation with 311 + 405 nm in time-dependent manner monitored by HPLC; (**c**) HPLC traces for **3i** in ACN/H_2O (1:1, v/v) under ambient light. (**d**) Histogram for the photo-decay of **3i** under ambient light in time-dependent manner monitored by HPLC-MS.

(**a**) Stability test of **3e**, in solid phase

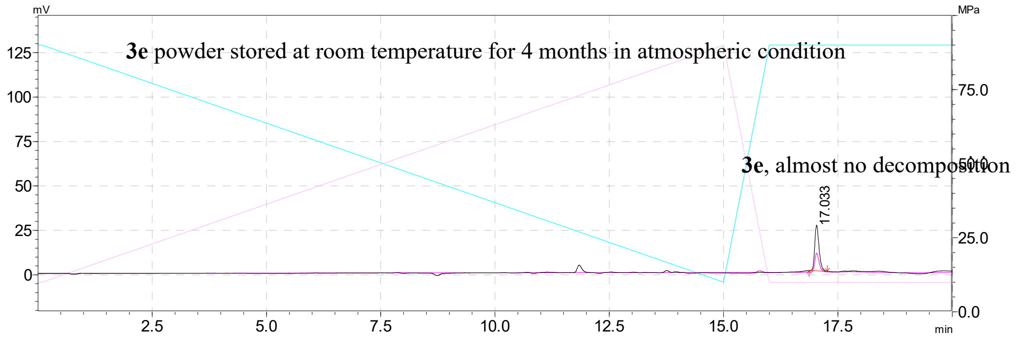

(**b**) Stability test of **3e** against tris(2-carboxyethyl)phosphine (TCEP)

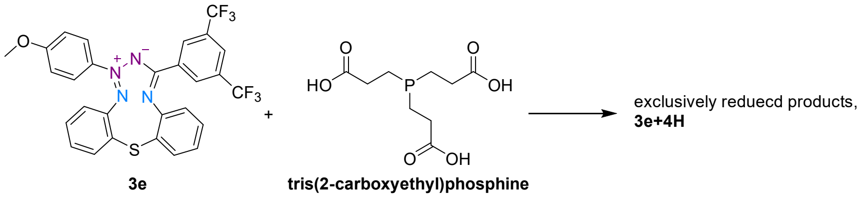

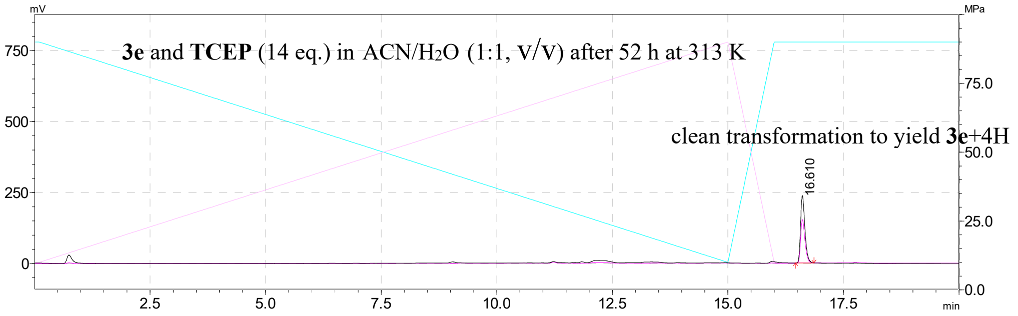

Peak No.	Time	Area	Height	%Area
1	16.610	1500056	233532	100

(**c**) Stability test of **3e** against TCEP and GSH

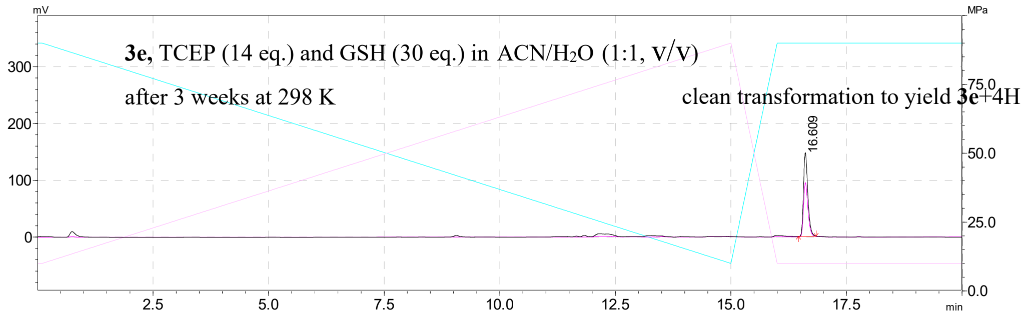

Peak No.	Time	Area	Height	%Area
1	16.609	971875	146881	100

Supplementary Figure 22. (**a**) HPLC-MS analysis of stability of **3e**. **3e** powder was stored at room temperature for 4 months in air to test its stability in solid phase. After dissolved in ACN/H_2O, there was almost no by-product monitored by HPLC, suggesting **3e** was stable in solid phase. (**b**) HPLC analysis for **3e** (87 μM) reduced by TCEP (14 eq. 12.4 mM) in ACN/H_2O (1:1, v/v) for 52 h at 313 K; (**c**) HPLC analysis for the resulting mixture added with glutathione (30 eq.) and stored for 3 weeks at 298 K. The identification was confirmed by LC-MS: MS (ESI) calcd. for **3e**+4H+H^+ $C_{28}H_{23}F_6N_4OS^+$ 577.15 [**3e**+4H+H^+], found 576.94. (**3e** was totally reduced by TCEP).

(a)

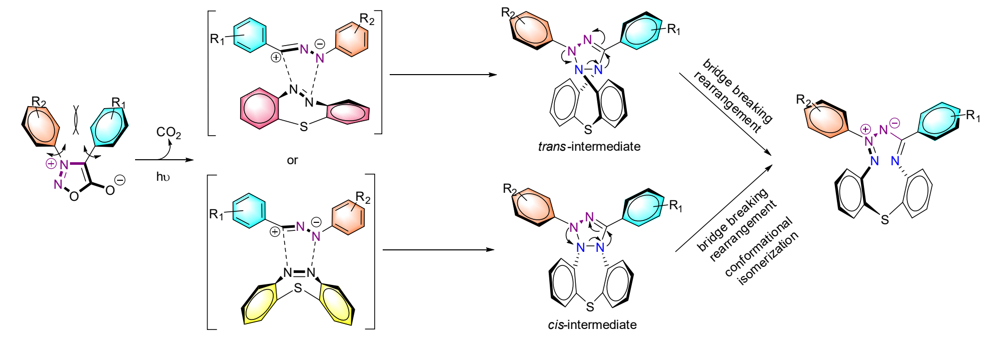

Nitrile imine generation via *in-situ* elimination to cycloaddition with **DBTD**

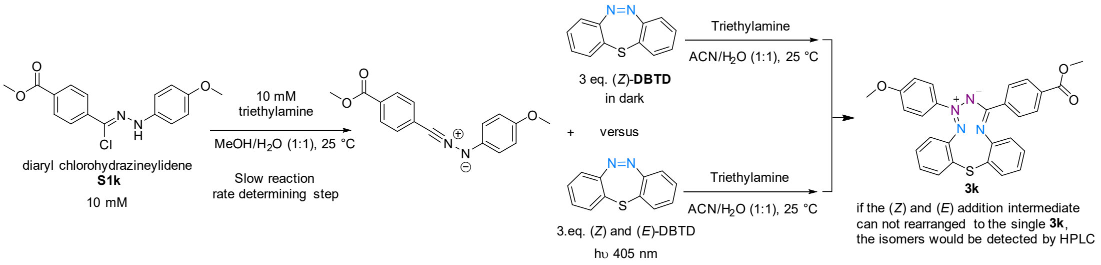

(b) Nitrile imine generation via *in-situ* elimination to cycloaddition with **DBTD** in dark for 3 h

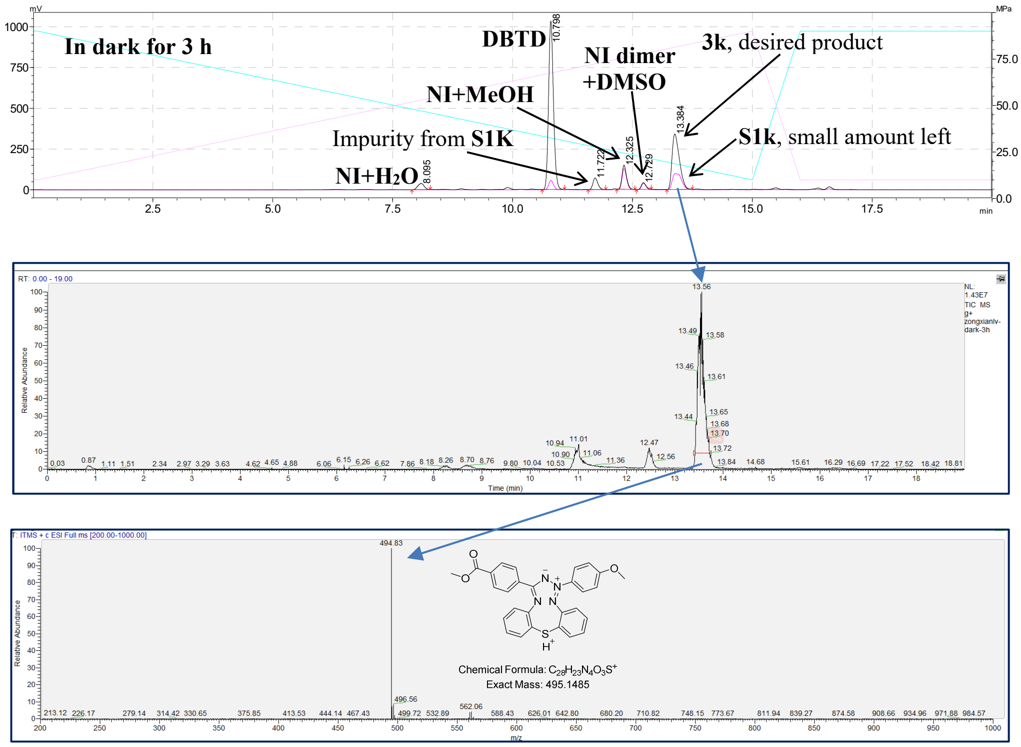

(**c**) Nitrile imine generation via elimination to cycloaddition with **DBTD** under 405 nm irradiation for 3 h

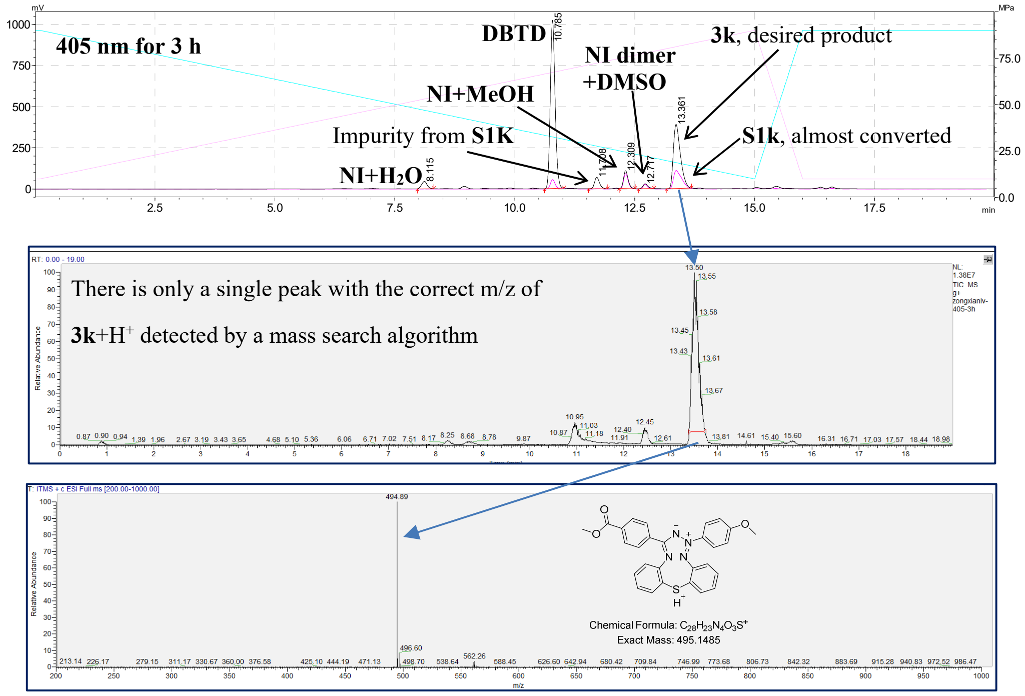

Supplementary Figure 23. Study of the photo-induced cycloaddition mechanism between NI and **DBTD**. (**a**) The proposed stepwise annulation and ring-expansion mechanism for the DASyd-DBTD photo-click reaction with (*Z*)- or (*E*)-**DBTD**. HPLC analysis for the cycloaddition reaction between chlorohydrazineylidene and **DBTD** in ACN/H_2O (1:1, v/v): HPLC trace of reaction mixture after (**b**) 3 h in dark or (**c**) with the 405 nm laser, respectively. The conversion from **S1k** to **3k** was calculated based on the absorbance at 254 nm, [NI = nitrile imine]. The mass identification of **3k** was confirmed by LC-MS: MS (ESI) calcd. for **3k** $C_{28}H_{23}N_4O_3S^+$ 495.15 [M+H$^+$], found 494.86.

Supplementary Note 2. Comparing the resultant HPLC traces of the reaction in dark *vs*. the *PSS* conditions with the 405 nm laser-stimulated **DBTD**, it can be seen that, except for the improved conversion of the starting material **S1k** to the desired product **3k**, there was no significant change in the composition of the rest peaks. At the same, there were no isomers of **3k** found via mass/charge searching process.

The experiment apparatus and procedure for the *PSS* measurement with 405 nm laser continuous illumination recorded by ^{1}H NMR *in-situ*.

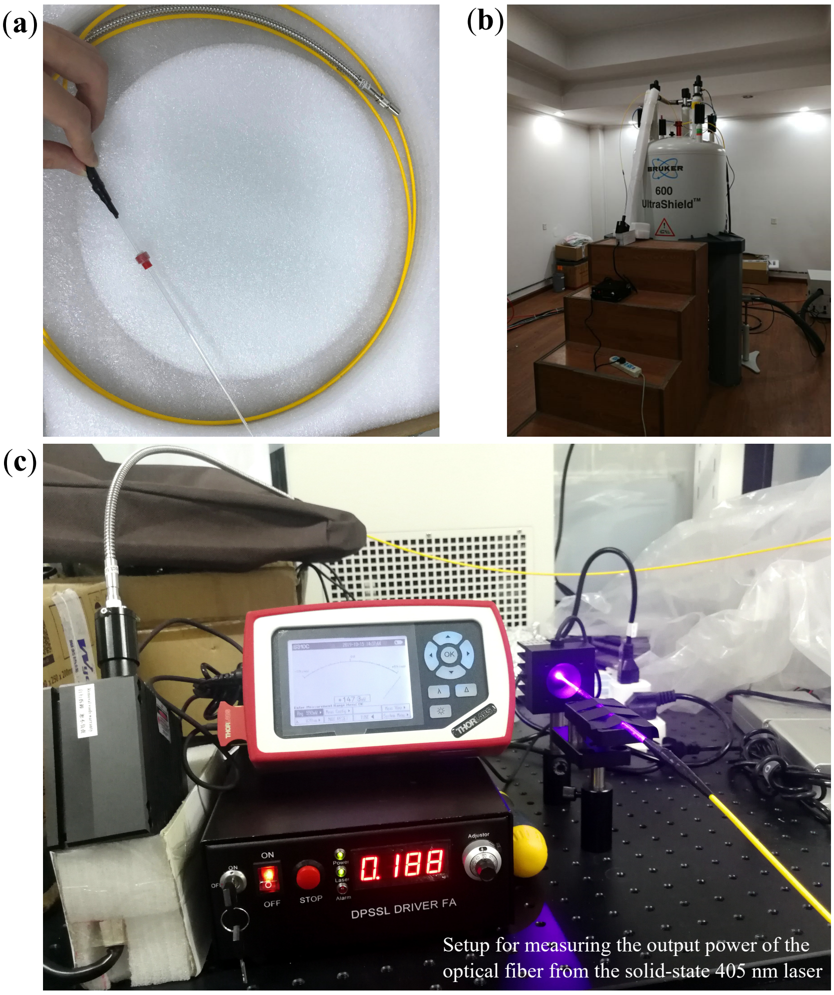

Supplementary Figure 24. (**a**) The inner tube of a coaxial quartz NMR tube was embedded with a quartz optical-fiber (core diameter: 1000 μm), aligning at the axial direction. The other end of the optical-fiber is combined with an adjustable solid-state 405 nm laser emitter (1 W) through the SMA905 interface. (**b**) The light irradiation was introduced into the interior of the NMR spectrometer by the optical fiber, and the interlayer of the NMR tube containing the sample to be tested can be placed in the sampling chamber of the NMR spectrometer. This setup allows the irradiance and temperature of the sample to be adjusted at any time while acquiring the NMR signals. As the illustration shown, the NMR spectra was obtained through an optical-fiber-guided *in-situ* excitation of 405 nm laser. The concentration of the substrate in the NMR tube should be

as low as possible for even irradiation of light to reach the *PSS*, while maintaining the resonance signal strong enough for better signal/noise ratio within a limited number of repetitions. (**c**) Instrument setup for measuring the output power of the optical fiber from the solid-state 405 nm laser.

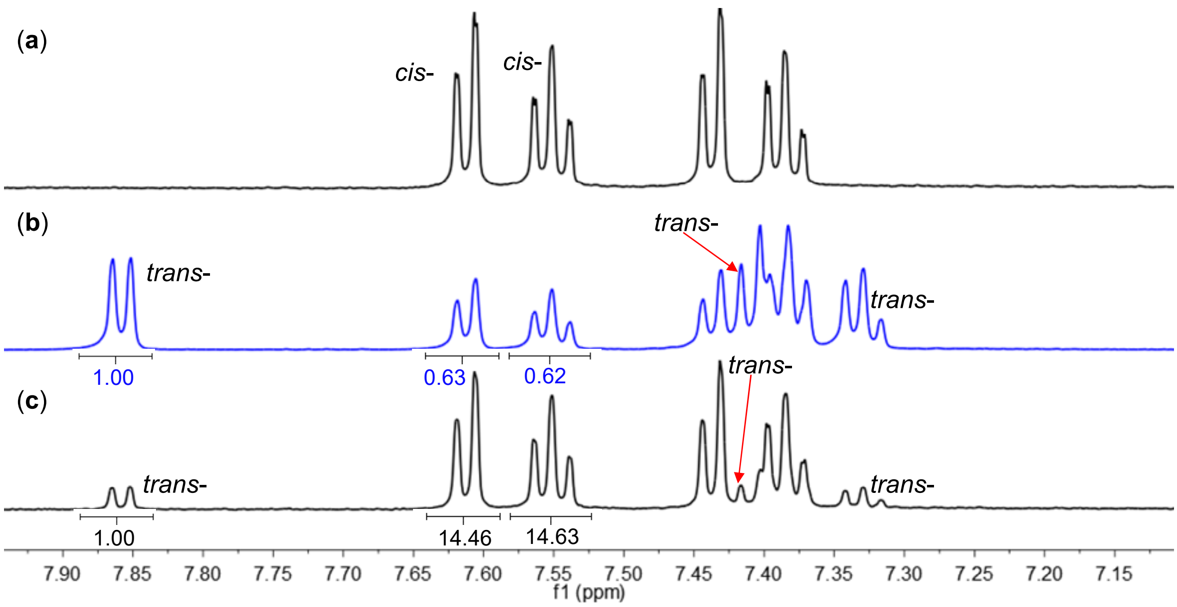

Supplementary Figure 25. ^{1}H NMR spectra of the photostationary state (*PSS*) study of **DBTD** under exposure of 405 nm light continuously in ACN-d_3 solvent at 233 K. (**a**) The initial spectra of (*Z*)-**DBTD**. (**b**) The spectra recorded under 405 nm laser irradiation continuously, (*Z*)/(*E*) = 5/8. (**c**) The spectra recorded after irradiation of the 405 nm laser withdrawn, recorded for 5 min. The decay rate of (*E*)-**DBTD** to (*Z*)-**DBTD** was significantly slower at 233 K than that at 298 K (3.0 mM in deuterated solvents, 600 MHz).

Photo-switching kinetic and photo-antifatigue studies of the DBTD under irradiation of 405 nm laser.

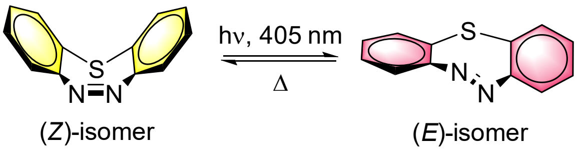

The unimolecular rate constants k_1 of the photo-switching process of the **DBTD** was measured via dynamic spectrum tracing upon a 405 nm laser irradiation intermittently (laser on for 2s and off for 3s per cycle) at 50 μM in ACN/H_2O = 1:1 (v/v; 0.1% DMSO). The laser irradiation was sat in front of a quartz lens to adjust the spot size of the laser beam from the laser emitting surface of the solid-state laser to the cuvette, while the cuvette was exposed to the laser irradiation spot as evenly as possible. Mixing appropriate volume of the prepared stock solutions to derive the desired final concentration in sample vials, and the mixture was transferred into 1 cm optical path quartz optical cuvette (405 nm laser irradiation optical path was 0.2 cm). Signals were read out by monitoring the characteristic absorbance signal, indicating the presence of the *trans* state. Kinetic runs were recorded using the following instrumental parameters: monitoring wavelength, $\lambda_{moni.}$= 285 nm; Interval 17 millisecond per data point over the recorded time range. The data sets were recorded and analyzed with the commercial software, Lightscan. All data processing was performed using Origin pro software. The thermostat, stirred cell and apparatus for studying the photo-switching performance of the **DBTD** is shown below.

(a)

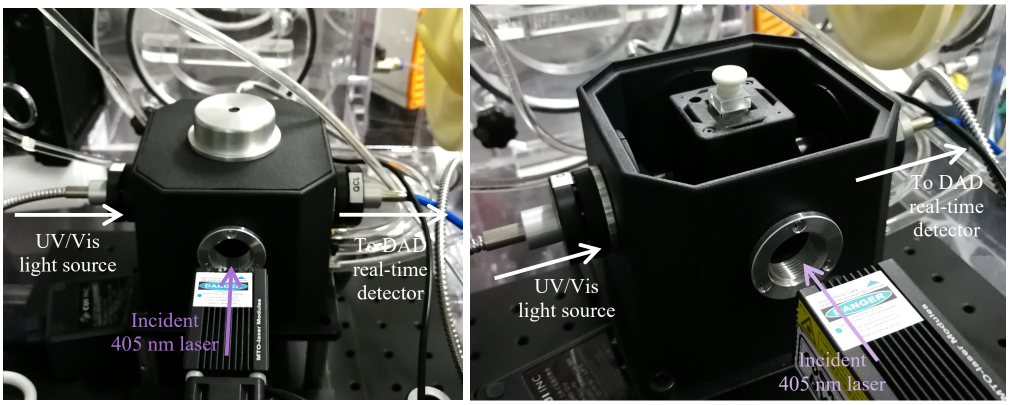

(b)

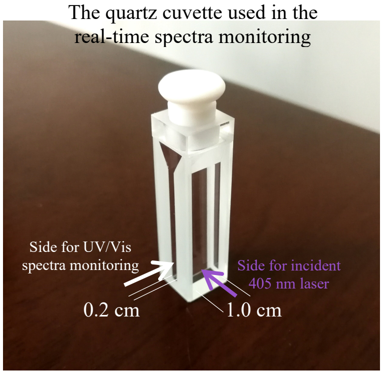

(c)

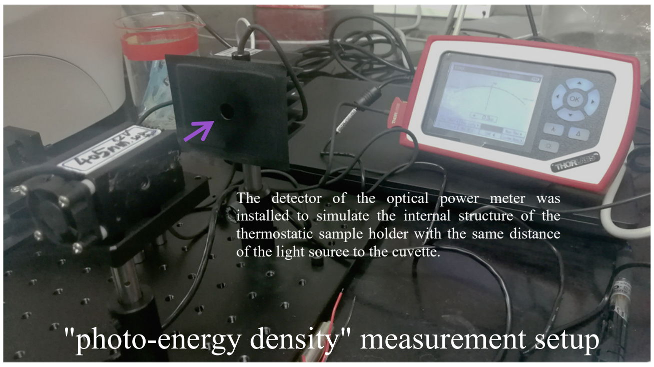

Supplementary Figure 26. The instrument setup for measuring photo-switching performance of the **DBTD**. (**a**) The apparatus for thermostated photo-switching of **DBTD** via 405 nm laser stimulation. The high-speed optical fiber spectrometer consciously records changes in absorbance in UV/Vis wavelengths. (**b**) The 1.0 × 0.2 cm optical path quartz optical cuvette used in the photo-chemical reactor. (**c**) The instrument setup for measuring the output power density of the 405 nm diode laser.

(a)

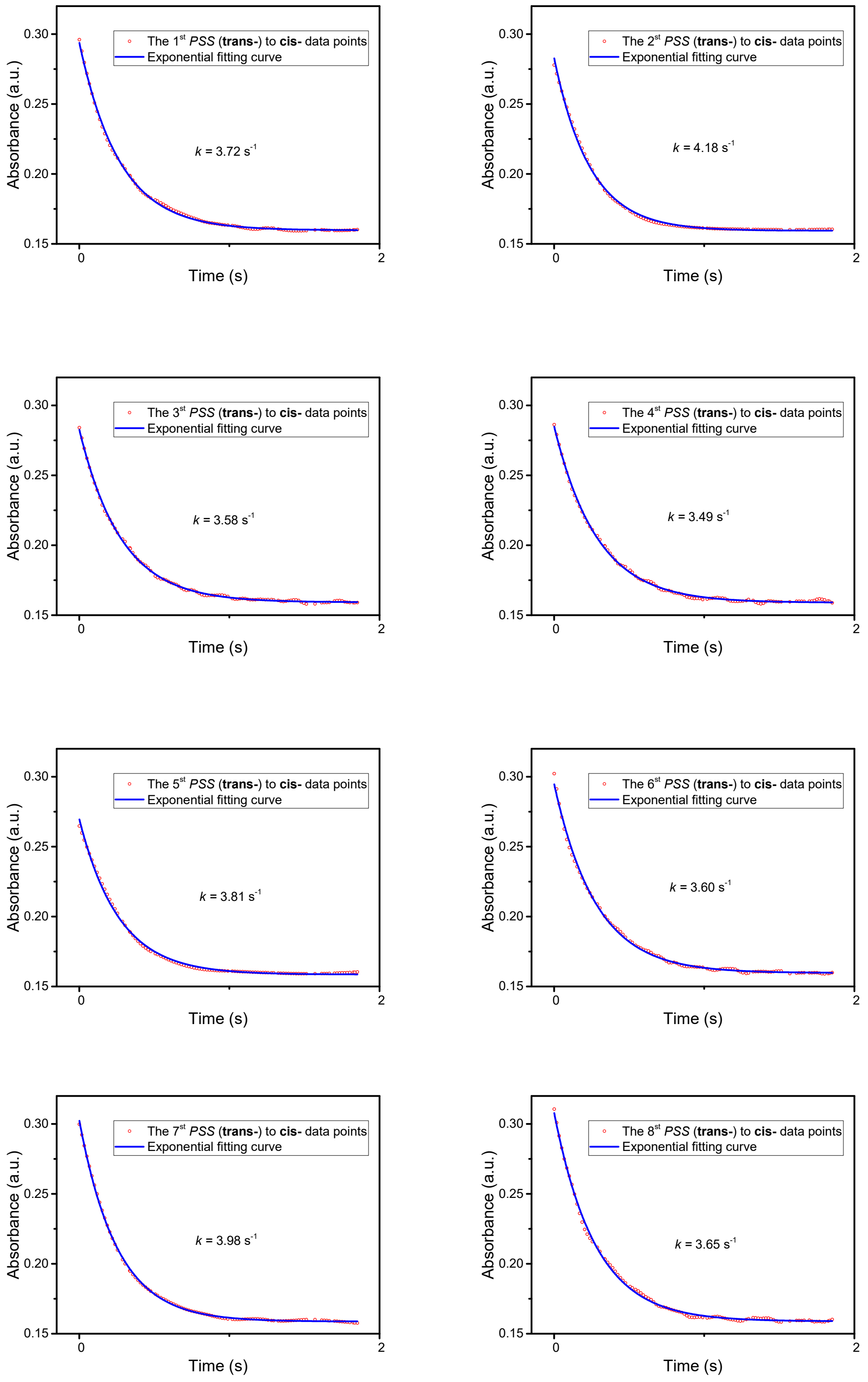
The 1st PSS (trans-) to cis- data points
Exponential fitting curve
k = 3.72 s-1
The 2st PSS (trans-) to cis- data points
Exponential fitting curve
k = 4.18 s-1
The 3st PSS (trans-) to cis- data points
Exponential fitting curve
k = 3.58 s-1
The 4st PSS (trans-) to cis- data points
Exponential fitting curve
k = 3.49 s-1
The 5st PSS (trans-) to cis- data points
Exponential fitting curve
k = 3.81 s-1
The 6st PSS (trans-) to cis- data points
Exponential fitting curve
k = 3.60 s-1
The 7st PSS (trans-) to cis- data points
Exponential fitting curve
k = 3.98 s-1
The 8st PSS (trans-) to cis- data points
Exponential fitting curve
k = 3.65 s-1
Absorbance (a.u.)
Time (s)
0.15
0.20
0.25
0.30
0
2

(b)

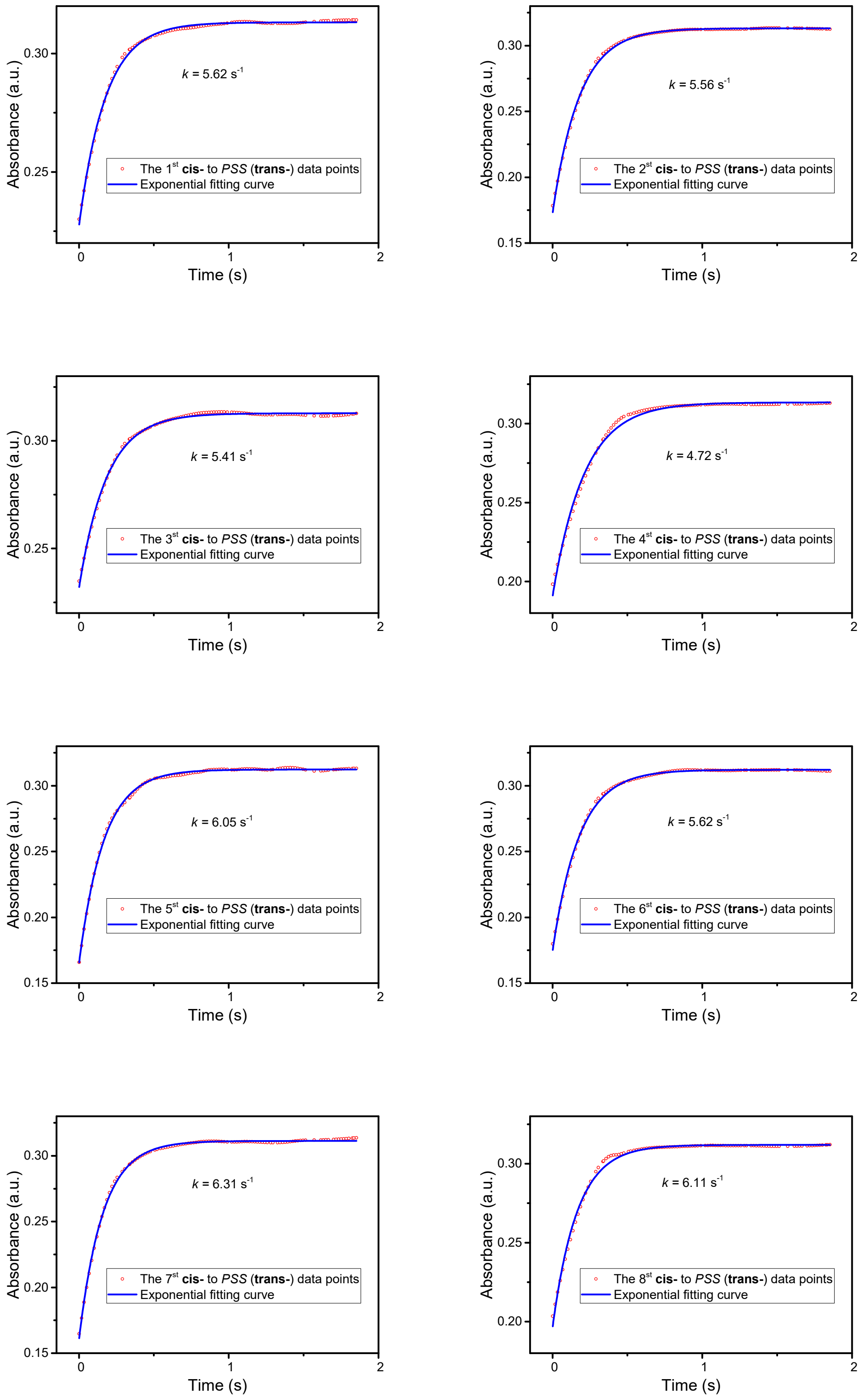
Absorbance (a.u.)
Time (s)
k = 5.62 s-1
The 1st cis- to PSS (trans-) data points
Exponential fitting curve
k = 5.56 s-1
The 2st cis- to PSS (trans-) data points
Exponential fitting curve
k = 5.41 s-1
The 3st cis- to PSS (trans-) data points
Exponential fitting curve
k = 4.72 s-1
The 4st cis- to PSS (trans-) data points
Exponential fitting curve
k = 6.05 s-1
The 5st cis- to PSS (trans-) data points
Exponential fitting curve
k = 5.62 s-1
The 6st cis- to PSS (trans-) data points
Exponential fitting curve
k = 6.31 s-1
The 7st cis- to PSS (trans-) data points
Exponential fitting curve
k = 6.11 s-1
The 8st cis- to PSS (trans-) data points
Exponential fitting curve

(**c**)

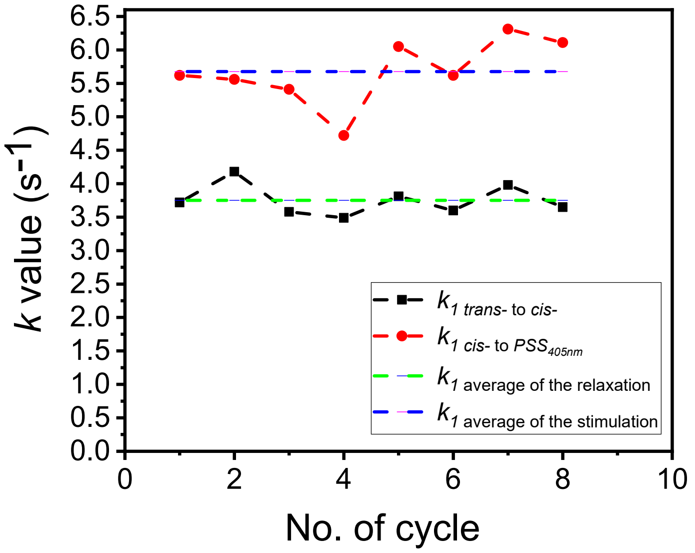

Supplementary Figure 27. Kinetic study of the conversion process of the **DBTD** between its *cis*-state and the *PSS*405nm by monitoring the absorbance intensity evolution at 285 nm with the 405 nm laser on/off in ACN/H_2O (1:1). (**a**) Detailed data points with exponential fitting curves of the absorbance intensity evolution at 285 nm and the averaged first order kinetic (k_{1relax}, *trans*- to *cis*-) was determined to be 3.75 ± 0.23 s^{-1}, 298 K. (**b**) Detailed data points with exponential fitting curves of the absorbance intensity evolution at 285 nm and the averaged kinetic (k_{405PSS}, the *cis*- to the *PSS*) value was determined to be 5.68 ± 0.50 s^{-1}, 298 K. (**c**) Reversible switching *k* values comparison of the *cis*-state to the PSS_{405nm} and the *trans*-state to the *cis*-state with the averaged values plotted, fitted and shown.

The anti-fatigue study of the photo-chemical conversion process between *cis*-state and PSS_{405nm}

(a)

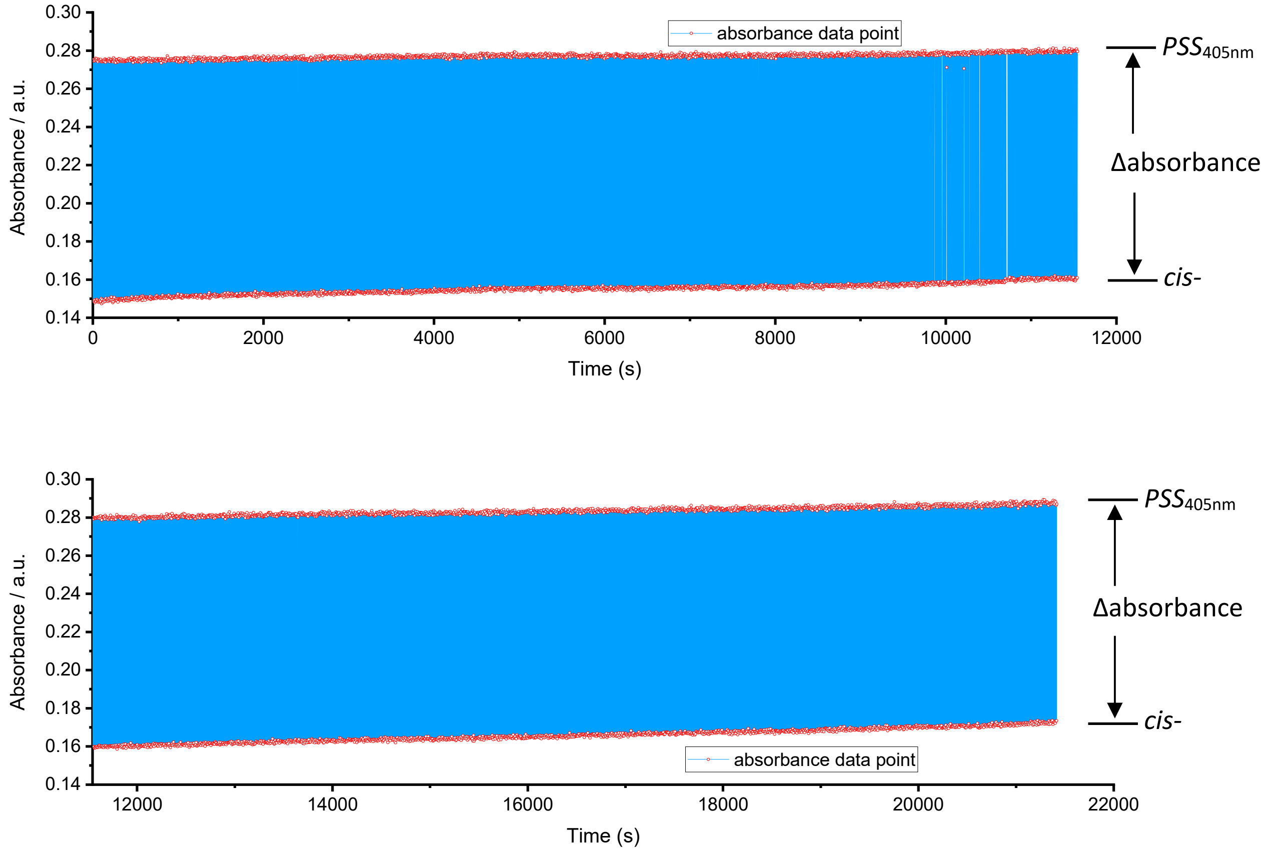

(b)

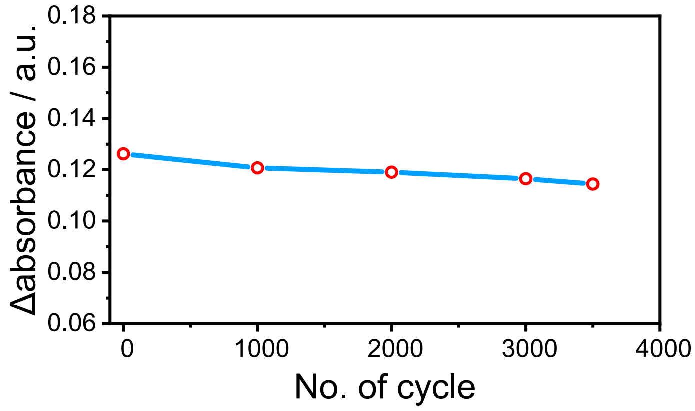

Supplementary Figure 28. The anti-fatigue study of the process of the photo-conversion between *cis*-state and PSS_{405nm} by monitoring the absorbance intensity evolution at 285 nm under the 405 nm laser on/off in ACN/ H_2O (1:1). (**a**) Full scale tracing the absorbance evolution @285 nm via 405 nm laser stimulation periodically (3s on and 3s off per cycle) over a total of 21417 s (3569 times of photo-switching cycles). (**b**) Plotting the decay of Δabsorbance versus the switching cycles. The "x axis" is cycle index, the "y axis" is the difference between the absorbance of PSS_{405} and *cis*-state in individual cycle**s**. The decay of the Δabsorbance was determined to be 9.43% versus that of the initial **DBTD** reagent which was also verified by HPLC-MS analysis, indicating a small amount of reduced dihydro-**DBTD** was generated as a cyclic diphenylhydrazine.

Supplementary Note 3. Determination of photo-switching quantum yield of the DBTD under irradiation of the 405 nm laser.[8,9]

For the photochromic compound, **DBTD**, there is a trade-off between the decoloration (from *trans*- to *cis*-, the color returned to yellow from red) speed and the decrease of the absorbance of a photo-generated isomer (PGI, the *trans*-**DBTD** isomer generated at 405 nm stimulation, a photo-chemical process). That is, when the thermal reversion reaction of the decoloration process becomes faster, the counter generation of the PGI (the *trans*-**DBTD**) at the *PSS* increase faster [the total absorbance is decreasing faster, due to the lower ε_E (< ε_z) at 405 nm]. The relationship of this "trade-off" can be described by solving the rate equations of the photochromic reaction of the **DBTD**.

The thermal steady state convert from *cis*-isomer to *trans*-isomer, approaching to the *PSS*, by irradiation of 405 nm light with an intrinsic quantum yield of $\emptyset_{ZE405}$, and the conversion of thermally switch-back has an unimolecular rate constant of k_{1relax} (*trans*- to *cis*-). We hypothesized the concentrate of *cis*-state did not change (d[Z] $\approx$ 0) at the *PSS* and regarded this photo-transformation as a two-state *trans*/*cis* system for simplicity.

There are two processes occurred simultaneously, one is the *cis*-state convert to PSS_{405nm} via the photo-stimulation, and the other one is the turn-back reaction under the control of thermodynamics. Therefore, the rate equations of the photochromic reaction of the **DBTD** can be written as follow (**Supplementary equation 4**, shown below). When this photo-process reaches the PSS_{405nm}, we can assume d[Z]/dt $\approx$ 0, the ε_z and ε_E are the absorption coefficients of (*Z*)- and (*E*)-isomer at the stimulation wavelength (405 nm), respectively.

F is the photokinetic factor for the photo-induced absorbance at the stimulation wavelength (405 nm, **Supplementary equation 5**), $[Z]_0$ is initial concentration of the solution, *l* is the thickness of the optical path which was 0.2 cm, I_0 is the incident monochromatic photon flux (405 nm laser) by the reaction medium at PSS_{405} in the unit: moles of photons liter^{-1} s^{-1} or einstein liter^{-1} s^{-1}. k_{1relax} is the thermal relaxation rate constant. At the *PSS*, $[E]_{PSS} = [Z]_0 - [Z]_{PSS}$, thus the $\emptyset_{ZE405}$ can be obtained from **Supplementary equation 7-8**. Via experimental acquisition, we obtained the k_{1relax} = 3.75 s^{-1}, ε_Z = 754 M^{-1} cm^{-1}, ε_E = 156 M^{-1} cm^{-1}, *l* = 0.2 cm, I_0 = 250 × 10^{-3} W cm^{-2} = 4.22 × 10^{-3} moles of photons liter^{-1} s^{-1} or einstein liter^{-1} s^{-1} (The photonic fluxes are volumic. The volume of the irradiated solution was 0.157 cm^3 in a cuvette with 0.2 cm optical path for photo-stimulation, with an exposed area of 0.785 cm^2), $[Z]_0$ = 50 μM, $[E]_{PSS}/[Z]_{PSS}$ = 0.2 (determined by ^{1}H NMR via *in-situ* acquisition under irradiation of 405 nm laser at 298K), therefore, $[Z]_{PSS}$ = 41.7 μM and the $\emptyset_{ZE}$ was derived to be 0.516.

$-\frac{d[Z]}{dt} = (k_{EZ} + \phi_{ZE}\varepsilon_{Z} l I_0 F)\,[Z] - k_{EZ}\,[Z]_0 \approx 0$ (if reached PSS_{405nm}) **Supplementary Equation 4**

Abs = $(\varepsilon_{Z}\,[Z] + \varepsilon_{E}\,[E])l = 0.006539$ **Supplementary Equation 5**

$F = (1 - 10^{-Abs})/\,Abs = 2.285$ (photokinetic factor) **Supplementary Equation 6**

The **Supplementary Equation 4** could be derived as followed if the PSS_{405nm} has been approached, where the $[Z] = [Z]_{PSS}$:

$[Z]_{PSS}\varepsilon_{Z} l I_0 F \phi_{ZE} = k_{EZ}\,([Z]_0 - [Z]_{PSS})$ **Supplementary Equation 7**

$\phi_{ZE405} = k_{EZ}\,([Z]_0 - [Z]_{PSS})\,/\,([Z]_{PSS}\varepsilon_{Z} l I_0 F)$ **Supplementary Equation 8**

The equations for determination of photo-quantum yield of the photo-switching process of the **DBTD**.

Stability test of the **DBTD** against 10 mM GSH in dark or under 405 nm irradiation

(a)

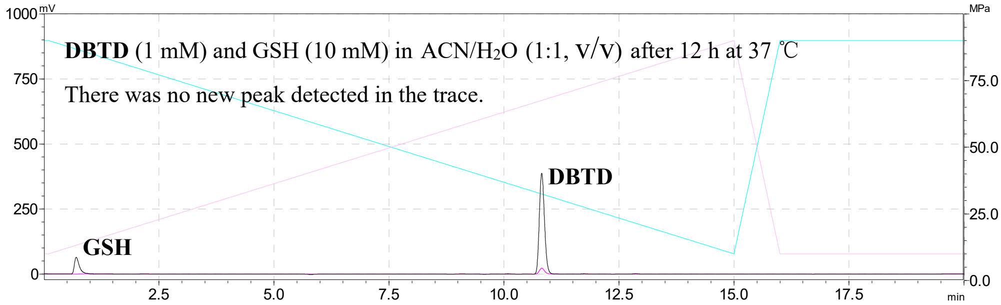

(b)

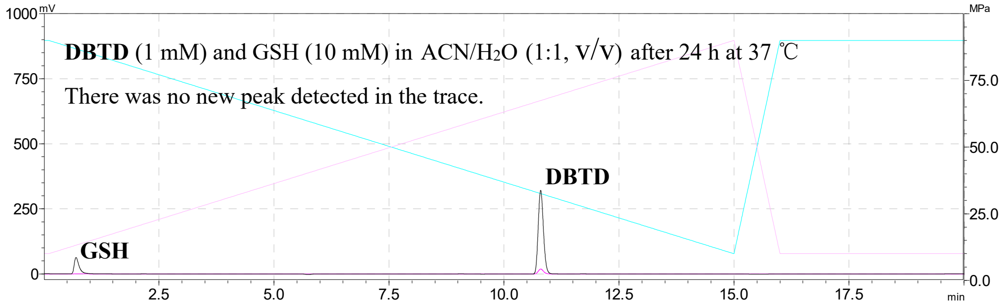

(c)

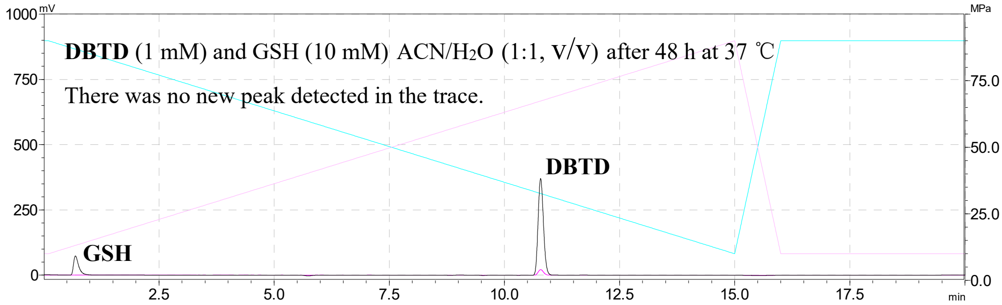

(**d**)

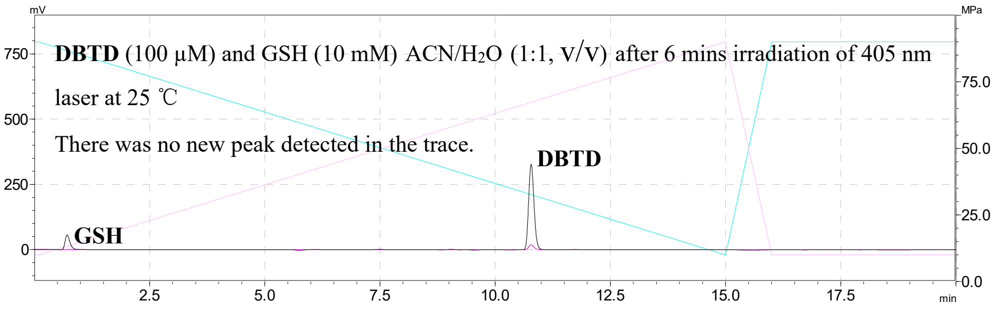

Supplementary Figure 29. HPLC analysis for the stability test of the **DBTD** toward GSH (10 mM in ACN/H_2O = 1:1). (**a**) **DBTD** (1 mM in ACN/H_2O = 1:1) and GSH (10 eq. 10 mM) in ACN/H_2O (1:1, v/v) for 12 h; (**b**) for 24 h; (**c**) for 48 h; at 37℃. (**d**) **DBTD** (100 μM in ACN/H_2O = 1:1) and GSH (100 eq. 10 mM) in ACN/H_2O (1:1, v/v) under irradiation with 405 nm laser for 6 min. The dihydro-**DBTD** was detected in trace amount (no peak detected in HPLC trace) as the reduced product of **DBTD** when 405 nm laser stimulation was applied, calculated for $C_{12}H_{11}N_2S^+$ 215.06 [M+H$^+$], found the mass/charge ratio, 215.03, in the HPLC trace.

Demonstration of the spatiotemporally controlled isomerization of the DBTD at low temperature.

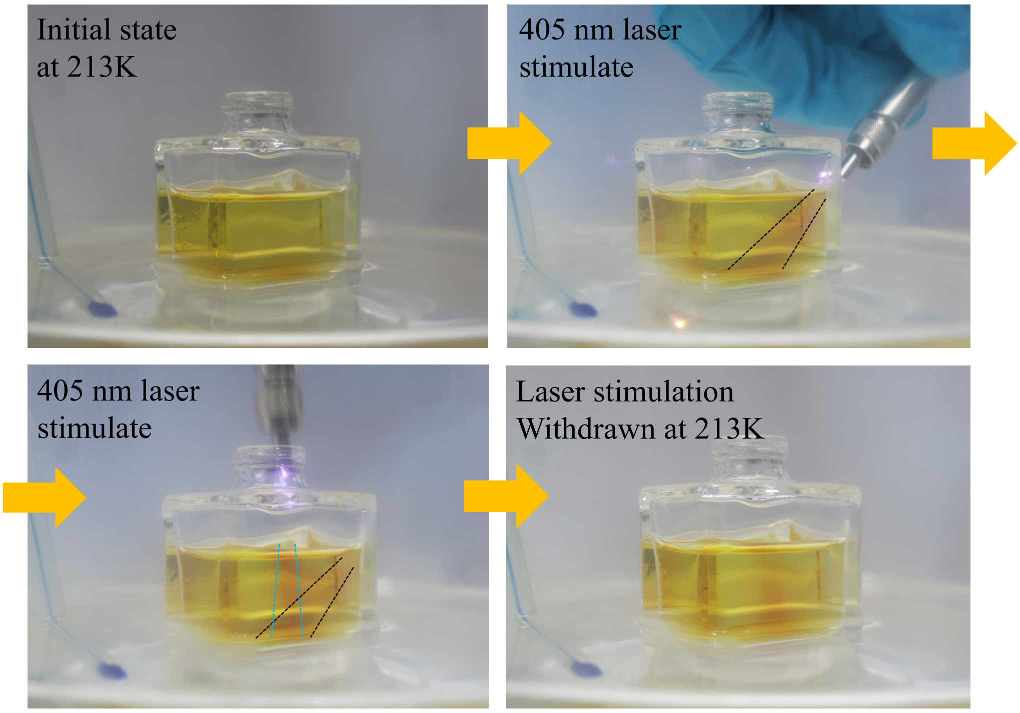

Supplementary Figure 30. Spatial control of the photo-switching of the **DBTD** (0.1 mg mL^{-1}) in ethanol at 213 K via *in-situ* stimulation through an optical fiber guided 405 nm laser. The switched *trans*-**DBTD** contents could be seen in the illumination path (crimson color, marked in the dash line region sequentially). The spatial controlled switching of the **DBTD** for twice at 213K via an optical fiber guided 405 nm laser could also been observed.

Supplementary Note 4. **Kinetic study of photo-induced 1,3-dipolar cycloaddition between the NI intermediate from tetrazole-1d (TAZ-1d) or DASyd 1d versus methacrylamide (MAA).**

Based on previous theory and study, the DASyd-DBTD photo-click chemistry is likely initialized with a photo-conversion of DASyd to NI which is followed by a cycloaddition between NI and **DBTD** and subsequent intramolecular rearrangement to generated the MAI product. We have attempted to directly measure the k_2 of the DASyd-DBTD cycloaddition step, but only to find the photo-conversion first-order rate of DASyd to NI which is irrelevant with the concentration of **DBTD**, because the k_2 of the DASyd-DBTD cycloaddition step (**DBTD** react with NI) is much faster than the first-order rate of DASyd to NI. To obtain the bimolecular rate constant between NI and **DBTD**, we thus have to utilize the diaryltetrazole (**TAZ-1d**) for producing the NI intermediate under continuous 311 nm LED array with power density as high as possible (for fast photolysis of **TAZ-1d**) and MAA as the dipolarophile (for relatively slow k_2), trying to make the cycloaddition step to be the rate-determining step.

According to our previous work, **TAZ-1d** and DASyd **1d** possess the same reaction intermediate, NI. However, the *pseudo*-first-order rate of converting of the DASyd **1d** to NI (releasing a CO_2, the rate-determining step) is much slower than that of the **TAZ-1d** (high photo-conversion quantum yield), converting to the identical NI (releasing a N_2). Therefore, we were unable to directly obtain the bimolecular rate constant (not the rate determining step) via tracing the product generation versus the cycloaddition reaction time. For the subsequent [3+2] cycloaddition reactions, the second-order rate constant k_2 between NI (generated from DASyd **1d**) and **DBTD** cannot be determined immediately. Therefore, we took an indirect approach, where the 1,3-dipolar cycloaddition happened between the less reactive olefin MAA, and **TAZ-1d** which possessed faster photo-induced first-order rate (generate the NI with releasing a N_2) was chosen to be the NI source, so that the second-order rate constant k_2 between NI and MAA was determined. Therefore, the second-order rate constant k_2 between NI and **DBTD** could be obtained via a competition strategy that the mixture of the **DBTD** vs. MAA toward DASyd is exposed to photo-triggering. By integrating the corresponding proton signal area to derive the

ratio of the MAI (**3d**) vs. pyrazoline **4** in ^{1}H and ^{19}F NMR spectra after the photo-irradiation, we were able to obtain the k_2 value of the target DASyd-DBTD reaction.

The second-order rate constants k_2 for the **TAZ-1d** (2 μM) or DASyd (**1d**) were measured under *pseudo*-first-order conditions with 250 μM, 500 μM, 750 μM and 1000 μM (125- to 500-fold excess) of MAA in ACN/H_2O = 1:1 upon the 311 nm LED array irradiation monitored by real-time tracing of fluorescence evolution. Signals were read out by monitoring the fluorescence signal appearance of the cycloaddition product, pyrazoline (Pyr). Kinetic runs were recorded by using the following instrumental parameters: monitoring wavelength, λ_{em}= 520 nm; 10 data points per second over the recorded time range. Stock solutions of the reactants were prepared for each DASyd (1 to 10 μM in ACN: H_2O = 1:1, v/v; 0.1% DMSO) and for MAA (1 mM in ACN:H_2O = 1:1, v/v). The data sets were averaged out of at least two replicates, which were recorded and analyzed with the commercial software Graphpad prism 6. All data processing was performed by using Origin pro software.

(a)

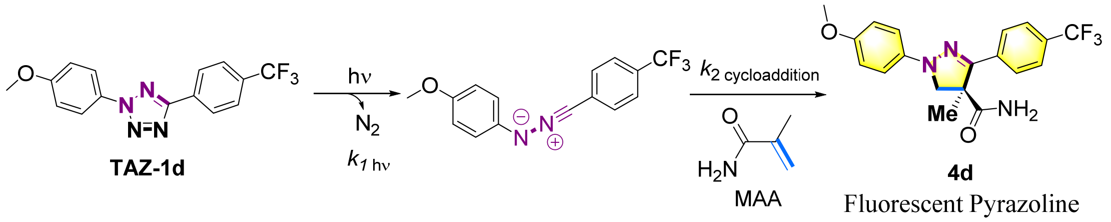

(b)

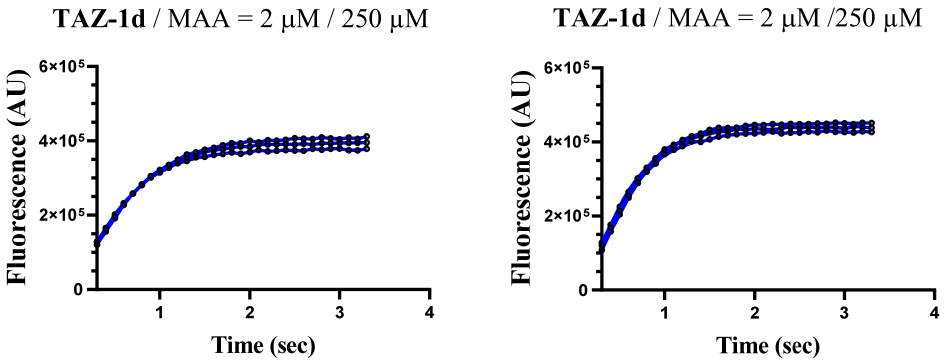

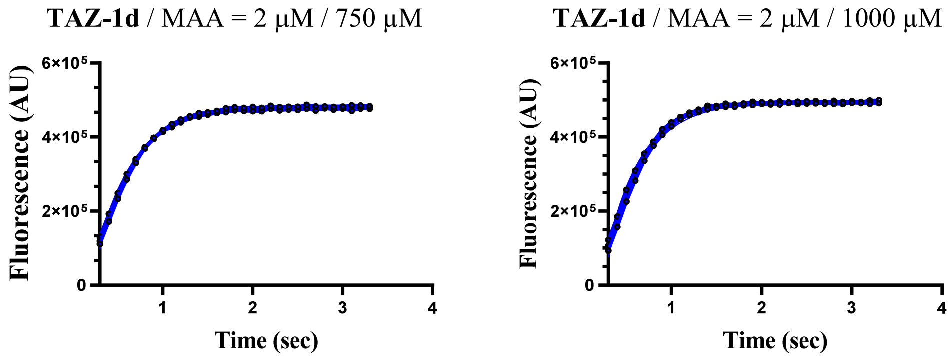

(c)

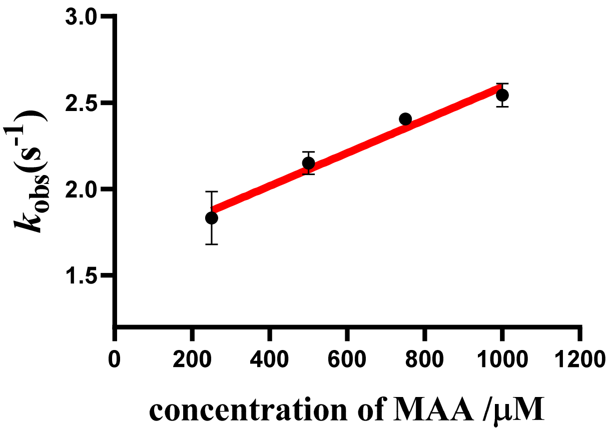

(d)

Test	k_2 ($M^{-1}s^{-1}$)	Linear Fitting equation	R^2
**TAZ-1d-MAA**	956 ± 98.3	y = 0.000956x + 1.635	0.9044

Supplementary Figure 31. Kinetic study for the photo-induced 1,3-dipolar cycloaddition step of **TAZ-1d** with MAA under irradiation of 311 nm LED array. (**a**) Reaction scheme of **TAZ-1d** toward MAA under light irradiation of 311 nm via NI intermediate generated via the photo-ring rupture of the tetrazole ($k_{1\,hv}$), releasing nitrogen gas, followed by cycloaddition between NI and MAA (k_2, biomolecular reaction rate constant). (**b**) Plots for the fluorogenic reaction of **TAZ-1d** with MAA in time-dependent manner. (**c**) Plots of the apparent rate constants, k_{obs}, versus MAA concentration with the data fitted into a linear equation. (**d**) Derived second-order rate constants, k_2, for the reaction of **NI-1d** with MAA at 298K. Values were determined from three independent measurements. Error bars denote standard deviation from three experimental replicates (n = 3).

Supplementary Note 5. **The competition reaction between the DBTD vs. MAA toward 1d via NMR *in-situ* recording to demonstrate the acceleration through 405 nm illumination**

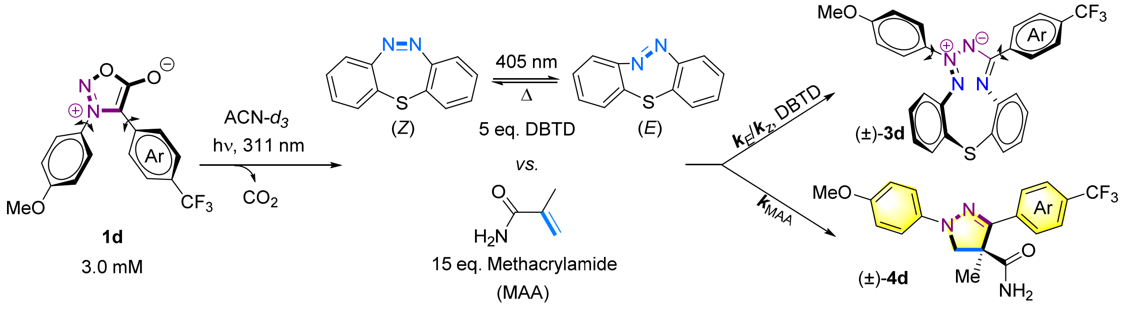

Because the DASyd **1d** could be converted to NI under 311 nm light but not under 405 nm light, we took a competition strategy in which the mixture of **DBTD** vs. MAA reacted with **1d** under irradiation of either 311 nm or 311 + 405 nm, respectively, in ACN-d_3 to study the cycloaddition kinetic and the acceleration of this reaction via 405 nm light assisted photo-isomerization of **DBTD**. Since the isomerization of **DBTD** would reach its PSS at 298K under continuous irradiation of 405 nm, there is only a small portion of (*Z*)-**DBTD** converted into "metastable" (*E*)-**DBTD**. It is because of the existence of a small amount of "metastable" state **DBTD** that react with NI (311 nm induced formation) in a faster rate, yielding two-fold of **3d** under the same concentration of competitive MAA (both the stable state and "metastable" state were transformed into the same product, **3d**). Therefore, when introduce the additional 405 nm, the ratio of **3d**/**4d** was increased about one-fold. The sum of **3d** can be divided into two parts, one of them is from the (*Z*)-**DBTD** and the second part from the (*E*)-**DBTD** (**Supplementary equation 12**). **Supplementary equation 11** and **Supplementary equation 14** were obtained to display the ratio of **3d**/**4d** at 311 nm or 311 + 405 nm, respectively. **Supplementary equation 18** is obtained after transformation of the equation (**Supplementary equation 11**)/(**Supplementary equation 14**) to extract the k_E/k_Z. As a result, the ratio of the bimolecular reaction constant (k_E/k_Z) of the "metastable" state versus the stable state is only related to the ratios of **3d**/**4d** acquired from the NMR signal at 311 (value *a*) or 311 + 405 (value *b*) nm and the ratio of [*Z*]/[*E*] (value *n*) at the *PSS*. $[B]_{311\ nm}$ is the concentration of **3d**, $[D]_{311\ nm}$ is the concentration of **4d** under 311 nm light irradiation. Similarly, $[B]_{311+405\ nm}$ is concentration of **3d**, $[D]_{311+405\ nm}$ is concentration of **4d** under 311 + 405 nm light irradiation. [A] is concentration of **DBTD**, [C] is concentration of MAA, k_Z, k_E, k_C, are the rate constants of the cycloaddition between (*Z*)-**DBTD**, (*E*)-**DBTD** and

MAA toward **1d**, respectively. $[A]_1$ is concentration of **DBTD** under 311 nm light irradiation. $[A]_Z$, $[A]_E$ are the concentration of (*Z*)-**DBTD** and (*E*)-**DBTD** under 311 + 405 nm light irradiation, $[A]_2 = [A]_Z + [A]_E$, $[C]_1 = [C]_2$ is the concentration of MAA under 311 nm or 311 + 405 nm light irradiation, respectively. We prepared the mixture of **1d**, **DBTD** and MAA (in the ratio = 1:5:15) in ACN-d_3, and $[A]_1 = [A]_2$ (two identical NMR samples under different irradiation condition), *PSS* = n, so the equation of ratio between k_Z and k_E could be simplified as followed.

$[B]_{311nm} = k_Z [A]_1 \cdot [NI]$ **Supplementary Equation 9**

$[D]_{311nm} = k_C [C]_1 \cdot [NI]$ **Supplementary Equation 10**

a = (**Supplementary equation 9**)/(**Supplementary equation 10**) = $(k_Z [A]_1)/(k_C [C]_1)$

Supplementary Equation 11

$[B]_{311+405nm} = (k_Z [A]_Z + k_E [A]_E) \cdot [NI]$ **Supplementary Equation 12**

$[D]_{311+405nm} = k_C [C]_2 \cdot [NI]$ **Supplementary Equation 13**

b = (**Supplementary equation 12**)/(**Supplementary equation 13**) = $(k_Z [A]_Z + k_E [A]_{E}$-$)/(k_C [C]_2)$

Supplementary Equation 14

The values of **Supplementary equation 11** and **14** could be obtained from NMR experiments.

$[A]_2 = [A]_Z + [A]_E$ **Supplementary Equation 15**

$PSS = [A]_Z/[A]_E = n$ **Supplementary Equation 16**

The values of n could also be obtained from *in-situ* NMR experiments.

(**Supplementary equation 11**)/(**Supplementary equation 14**) → $a\,(nk_Z + k_E) = b\,k_Z (n + 1)$ **Supplementary Equation 17**

(**Supplementary equation 17**) → $k_E/k_Z = [b\,(n + 1) - an]/a$

Supplementary Equation 18

The deducing formula of the relative rate constant ratio of k_E/k_Z.

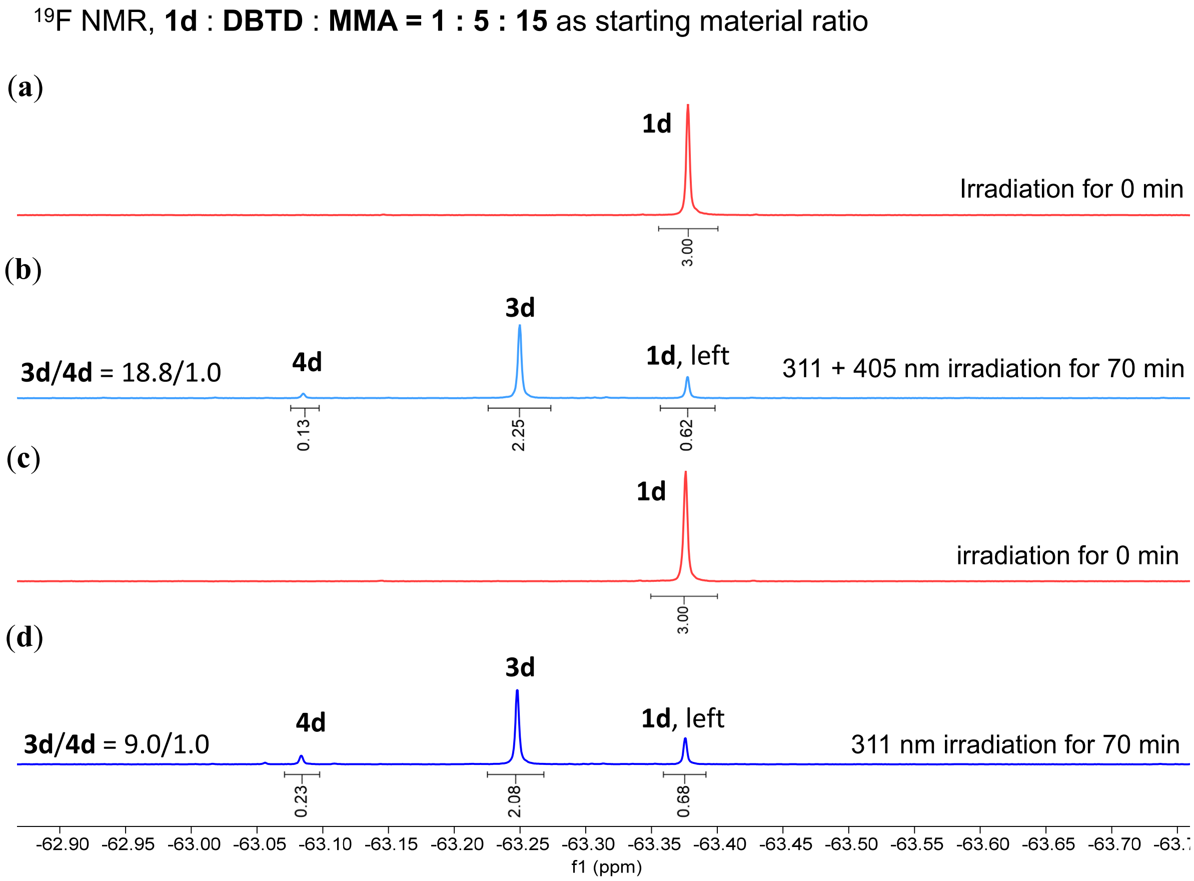

Supplementary Figure 32. ^{19}F NMR spectra of competition reactions of **DBTD** vs. MAA toward **1d** (3 mM) in ACN-*d*3 solvent at 25 °C. The initiate ratio of **1d** : **DBTD** : MAA = 1 : 5 : 15. (**a**) The ^{19}F NMR spectrum before photo-irradiation. (**b**) The ^{19}F NMR spectrum after photo-irradiation of 311 + 405 nm light for 70 min. (**c**) The ^{19}F NMR spectrum before photo-irradiation. (**d**) The ^{19}F NMR spectrum after photo-irradiation of 311 nm light for 70 min.

The competition reaction between the DBTD vs. TCO toward 1d via NMR *in-situ* recording

(a)

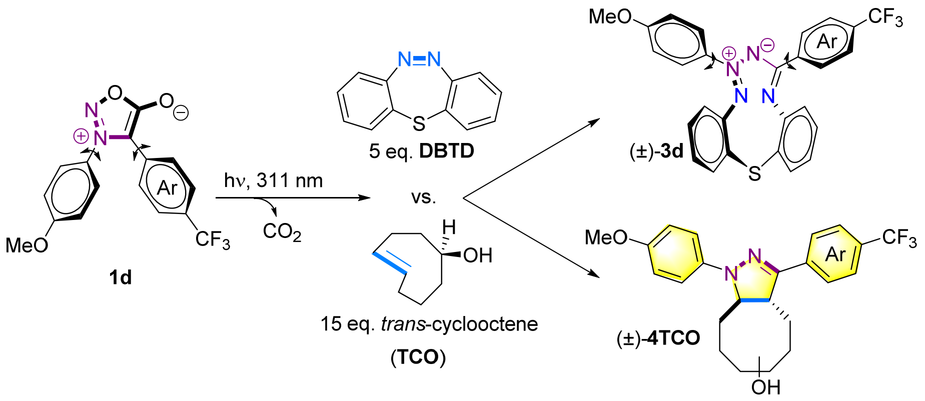

(b)

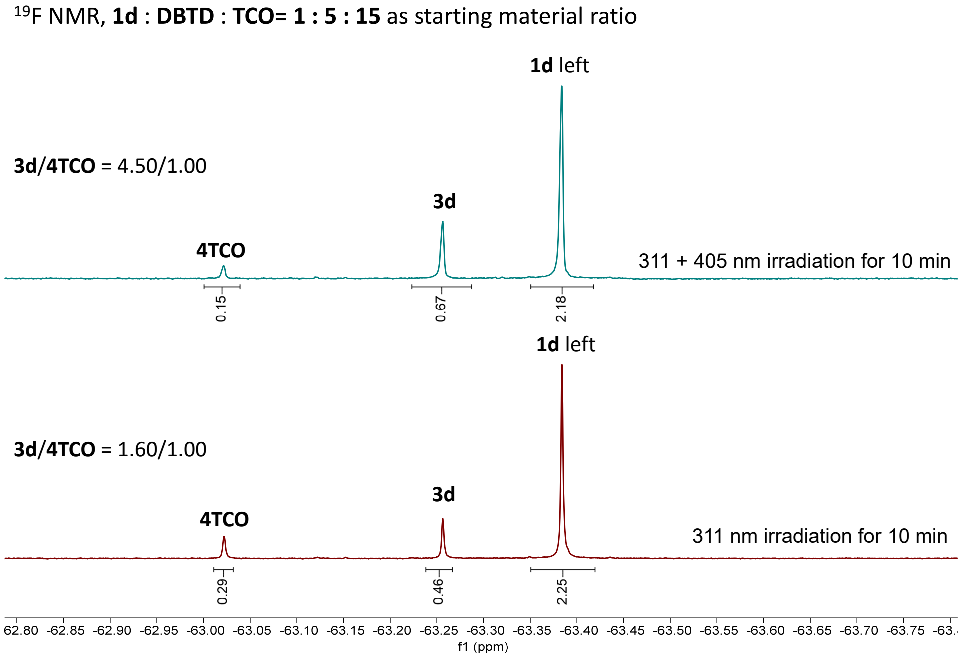

(**c**)

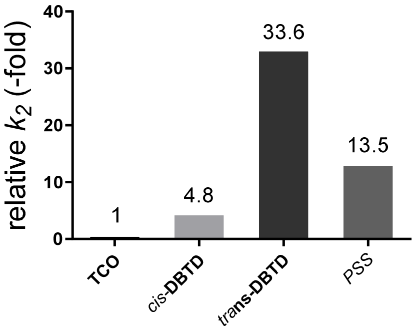

Supplementary Figure 33. (**a**) Competitive cycloaddition of the **DBTD** vs. **TCO** under various irradiation conditions via ^{19}F NMR monitoring. (**b**) ^{19}F NMR spectra of competition reaction of **DBTD** vs. **TCO** toward **1d** (3 mM) in ACN-d_3 solvent at 25 °C. The initiate ratio of **1d** : **DBTD** : TCO = 1 : 5 : 15. (**c**) The relative reactivity of **TCO** or *cis*-**DBTD** or *trans*-**DBTD** or the *PSS* toward NI displayed by histogram of the relative reaction rates of the bimolecular cycloaddition step.

(a)

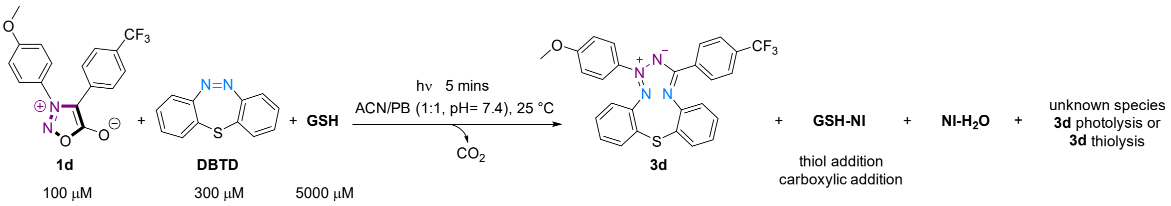

Product distribution of **1d** photo-click reaction at 311 nm

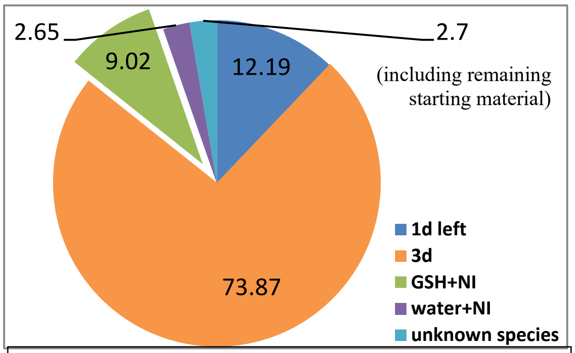

3d yield (excluding SM left): 84%
Selectivity **3d** vs. **GSH-NI** = ([**3d**]/[**DBTD**])/([**GSH+NI**]/[**GSH**]) = 135

Product distribution of **1d** photo-click reaction at 311 + 405 nm

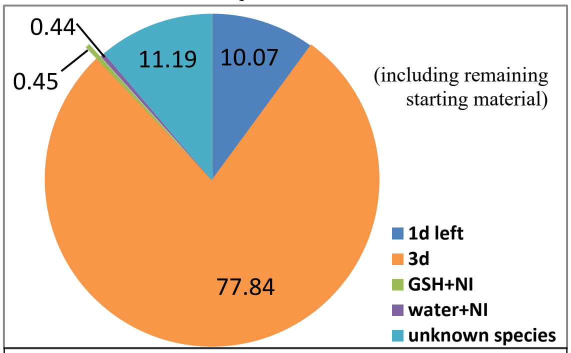

3d yield (excluding SM left): 87%
Selectivity **3d** vs. **GSH-NI** = ([**3d**]/[**DBTD**])/([**GSH+NI**]/[**GSH**]) = 2883

Under add-on 405 nm irradiation, the ratio of **3d**/GSH-NI was increased by 21-fold, indicating a higher selectivity if the (*E*)-**DBTD** is generated.

(b)

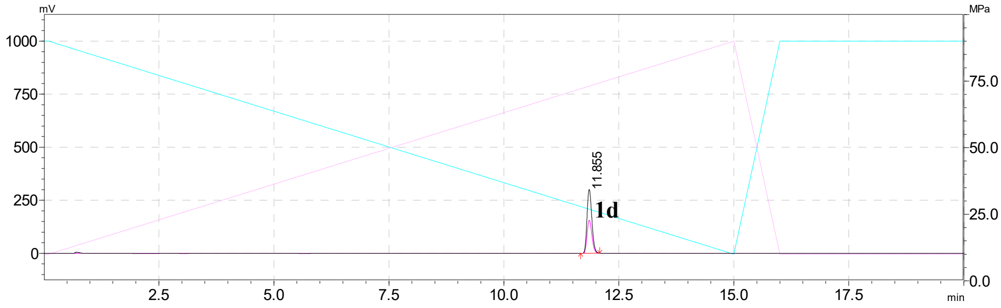

Peak No.	Time	Area	Height	%Area
1	11.855	2200644	300579	100

(c)

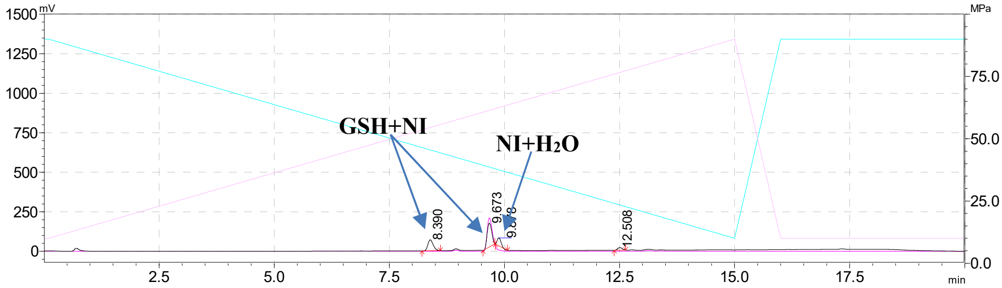

Peak No.	Time	Area	Height	%Area
1	8.390	561129	69260	28.235
2	9.673	1032868	149911	51.972
3	9.878	278436	50394	14.010
4	12.508	114919	16307	5.783

(d)

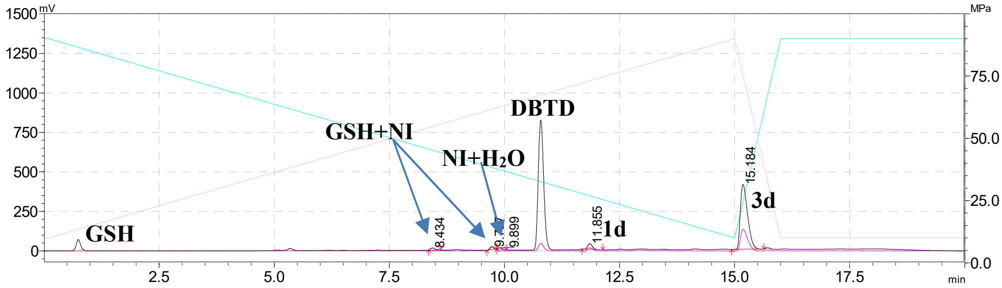

Peak No.	Time	Area	Height	%Area
1	8.434	93532	12908	1.879
2	9.727	98806	17557	1.985
3	9.899	56597	9973	1.137
4	11.855	248654	35543	4.995
5	15.184	4480056	409640	90.004

(e)

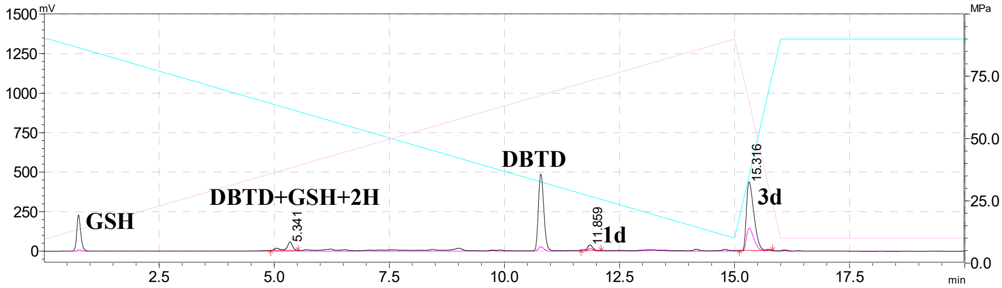

Peak No.	Time	Area	Height	%Area
1	5.341	514352	54051	8.624
2	11.859	246552	33806	4.134
3	15.316	5203156	437254	87.242

Supplementary Figure 34. HPLC analysis for photo-click reaction of **1d** toward **DBTD** in the presence of GSH in PB/H_2O (1:1, v/v), pH = 7.4, under bio-mimic conditions: (**a**) The cycloaddition scheme and the distribution of the products. (**b**) HPLC trace of 100 µM **1d**; (**c**) HPLC trace of 100 µM **1d** and 5 mM GSH after 5 min photo-irradiation with 311 nm. (**d**) HPLC trace of 100 µM **1d**, 300 µM **DBTD** and 5 mM GSH after 5 min photo-irradiation with 311 nm. (**e**) HPLC trace of 100 µM **1d**, 300 µM **DBTD** and 5 mM GSH after 5 min photo-irradiation with 311 + 405 nm. The product distribution was analyzed by reverse-phase HPLC with absorbance setting at 254 nm for integration of the peak area.

(**a**)

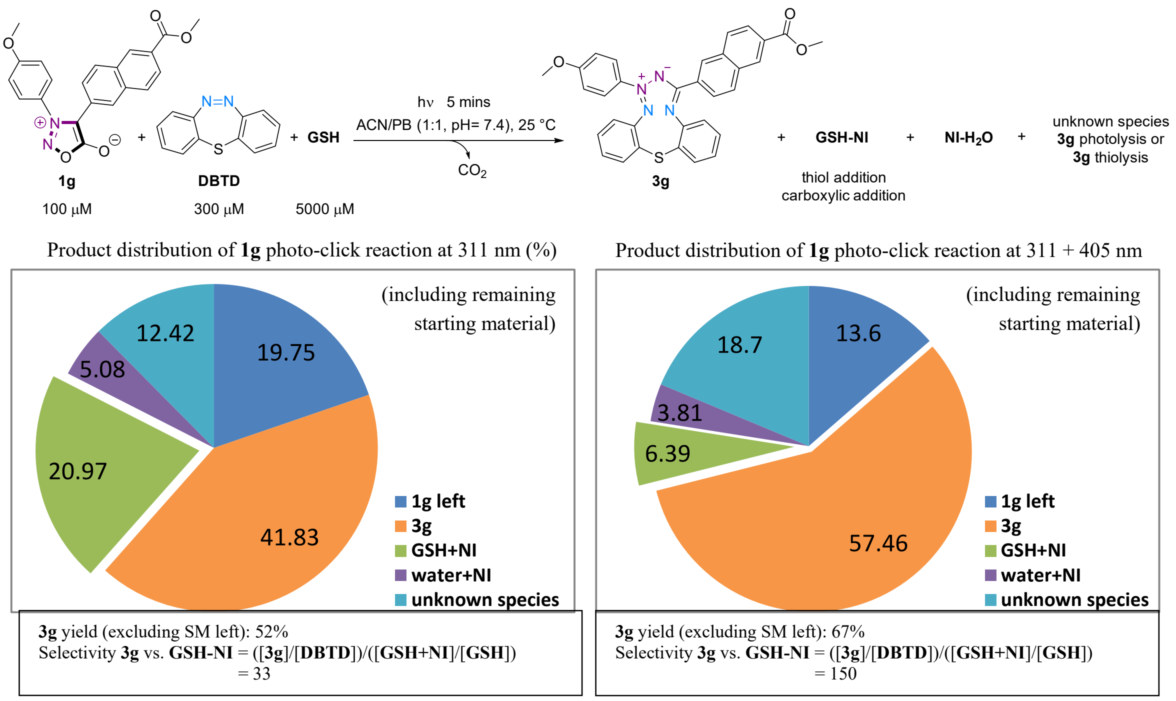

Under add-on 405 nm irradiation, the ratio of **3g**/GSH-NI was increased by 4.5-fold, indicating a higher selectivity if the (*E*)-**DBTD** is generated.

(b)

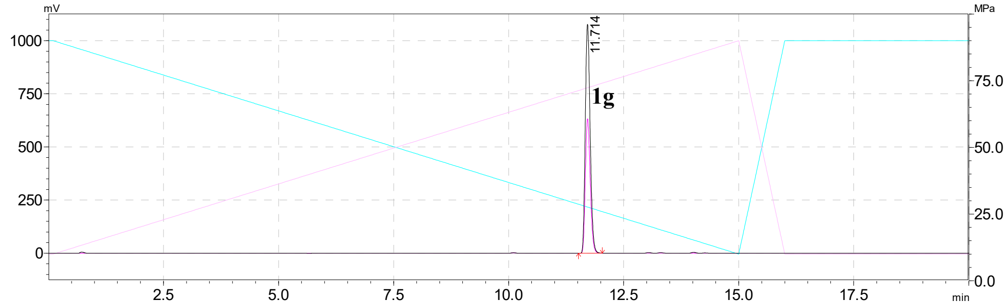

Peak No.	Time	Area	Height	%Area
1	11.714	7908507	1075474	100

(c)

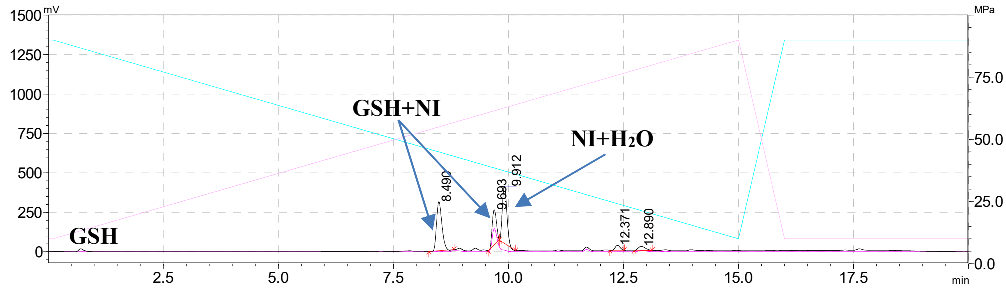

Peak No.	Time	Area	Height	%Area
1	8.490	2480266	309879	36.167
2	9.693	1405900	220228	20.501
3	9.912	2441093	361184	35.596
4	12.371	229523	31406	3.347
5	12.890	301061	26544	4.390

(d)

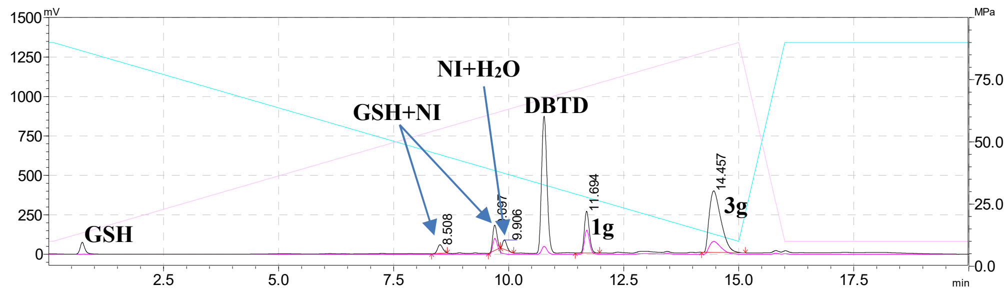

Peak No.	Time	Area	Height	%Area
1	8.508	423062	54564	3.981
2	9.697	1026214	159834	9.656
3	9.906	351887	59351	3.311
4	11.694	1946008	262616	18.311
5	14.457	6880566	390093	64.742

(e)

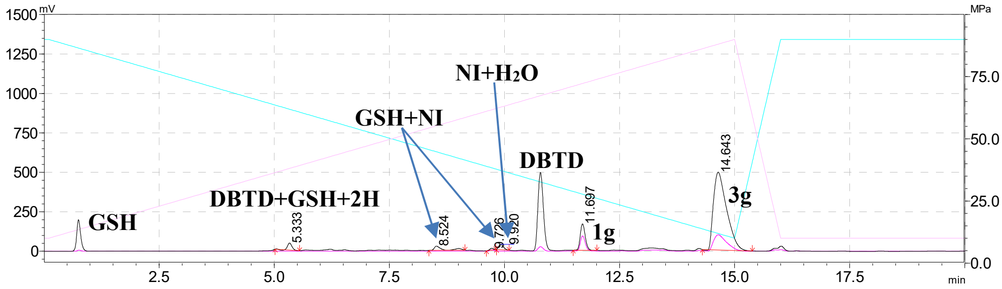

Peak No.	Time	Area	Height	%Area
1	5.333	291070	44827	2.426
2	8.524	347809	26918	2.898
3	9.726	63278	11188	0.527
4	9.920	245194	34189	2.043
5	11.697	1244652	166417	10.372
6	14.643	9807775	493053	81.733

Supplementary Figure 35. HPLC analysis for photo-click reaction of **1g** toward **DBTD** in the presence of GSH in PB/H_2O (1:1, v/v), pH = 7.4, under bio-mimic conditions: (**a**) The cycloaddition scheme and the distribution of the products. (**b**) HPLC trace of 100 μM **1g**; (**c**) HPLC trace of 100 μM **1g** and 5 mM GSH after 5 min photo-irradiation with 311 nm. (**d**) HPLC trace of 100 μM **1g**, 300 μM **DBTD** and 5 mM GSH after 5 min photo-irradiation with 311 nm. (**e**) HPLC trace of 100 μM **1g**, 300 μM **DBTD** and 5 mM GSH after 5 min photo-irradiation with 311 + 405 nm. The product distribution was analyzed by reverse-phase HPLC with absorbance setting at 254 nm for integration of the peak area.

Chemical modification of proteins by DBTD-NHS

To 0.485 mL solution of protein (lysozyme or BSA, 100 μM in 100 mM NaH_2PO_4, 25 mM NaOAc, pH 8.5) was added **DBTD-NHS** (7.5 μL, 10 mM in DMSO; final concentration = 300 μM). The resulting solution was incubated on a rotating shaker at room temperature for 3 h. Excess amount of small molecules was removed from the protein by protein spin columns (10 KDa cutoff) using 0.25 M $NH_3{\cdot}H_2O$ solution as eluent. And the **BSA-DBTD** was further modified with FITC (15 μL, 60 eq. 10 mM in DMSO) for 3h. The modified lysozyme was characterized by LC-ESI/MS: Lyso, calcd. 14304 Da, found 14300 ± 0.3 Da, Lyso-**DBTD**, calcd. 14637 Da, found 14637.5 ± 0.2 Da.

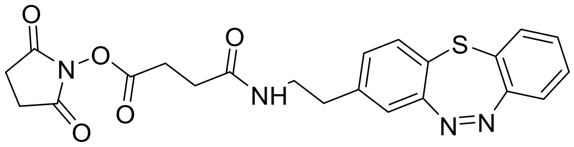

DBTD-NHS

(a)

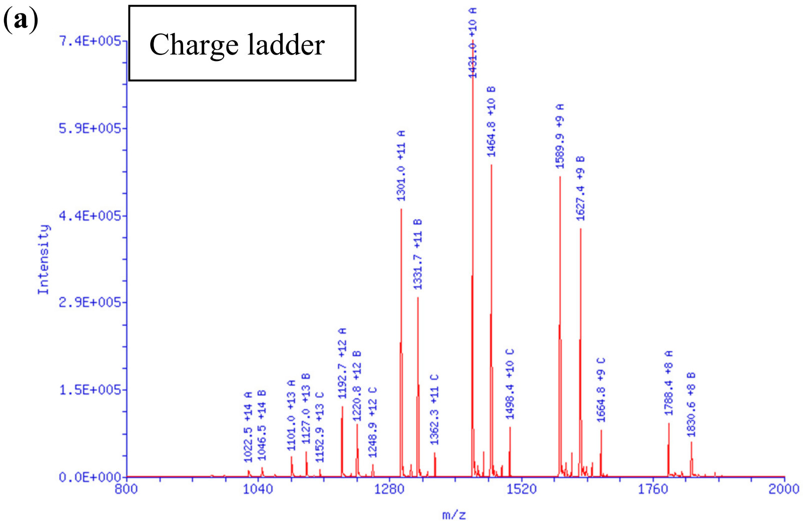

(**b**)

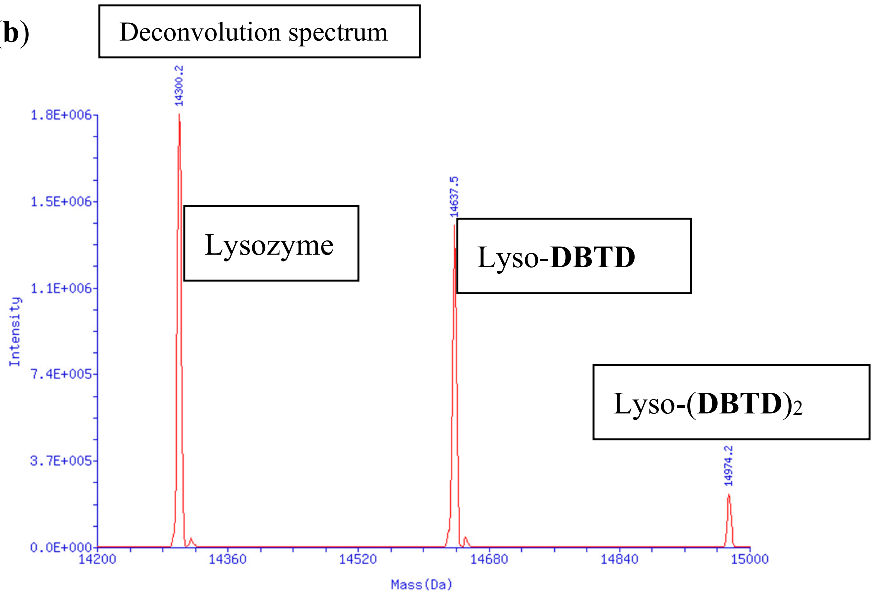

Mass (Da)	Intensity	Score	Delta Mass	%Total
14300.2 (Lyso)	1.85E+006	10.7	0	53.6
14637.5 (Lyso-**DBTD**)	1.37E+006	9.17	337.3	39.85
14974.2 (Lyso-**DBTD**2)	2.26E+005	4.30	674	6.55

Supplementary Figure 36. HPLC-MS spectra of the chicken lysozyme (Lyso) and **DBTD**-modified Lyso. (**a**) The charge ladders of the HPLC-MS spectrum of the modified Lyso-**DBTD**. (**b**) The deconvolution MS spectrum.

Photo-activated cycloaddition reaction of Lyso-DBTD with DASyd 1d resolved by HPLC-MS.

To 8 μL samples of 50 μM lysozyme-**DBTD** in PBS (phosphate buffer saline, pH = 7.4) were added 1 μL **1d** (10 mM in DMSO; final concentration = 100 μM) and 91 μL PBS. After irradiation with a hand-held 311 nm UV lamp or 311 nm + 405 nm (LED array) combination for 30s, respectively. The resulting protein mixture was characterized by LC-MS.

(**a**) 311 nm

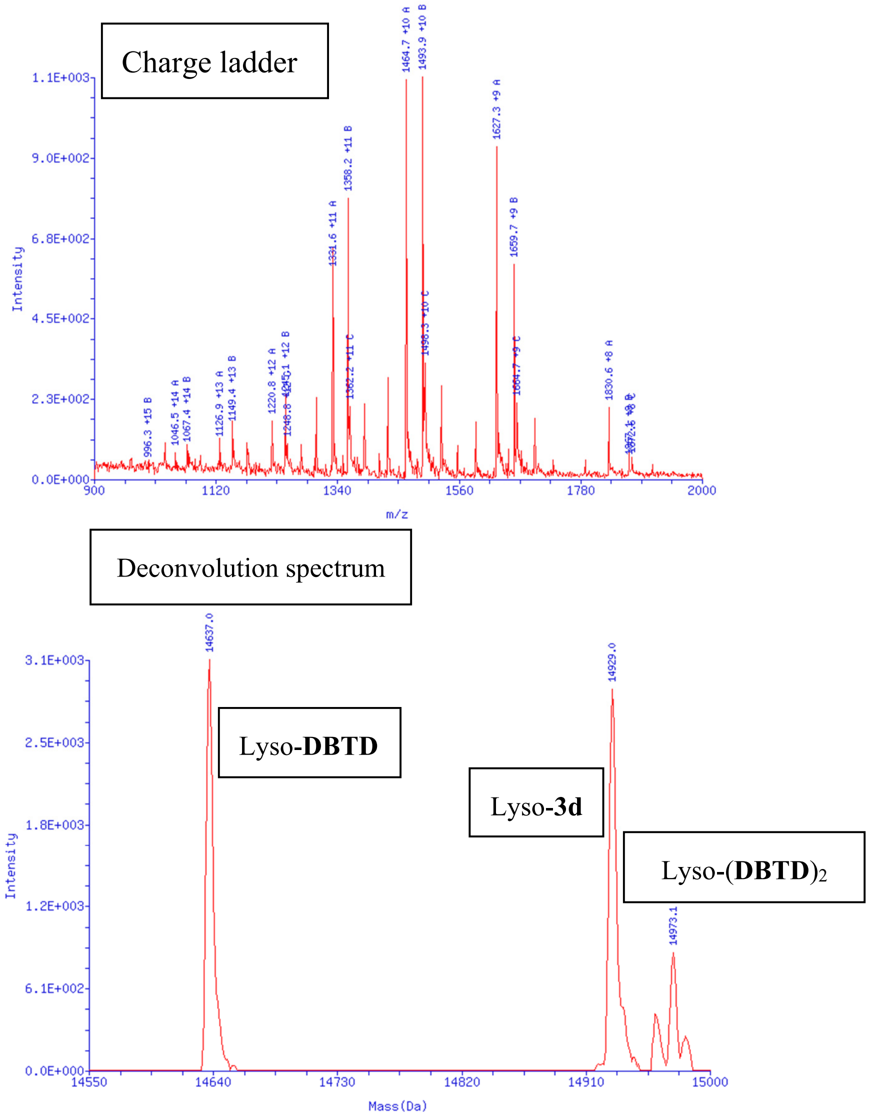

Mass (Da)	Intensity	Score	Delta Mass	%Total
14637.0 (Lyso-**DBTD**)	3.07E+003	4.70	0.0	45.16
14929.0 (Lyso-**3d**)	2.85E+003	4.52	292	41.88
14973.1 (Lyso-**DBTD**$_2$)	8.81E+002	2.24	336.1	12.96

(**b**) 311 + 405 nm

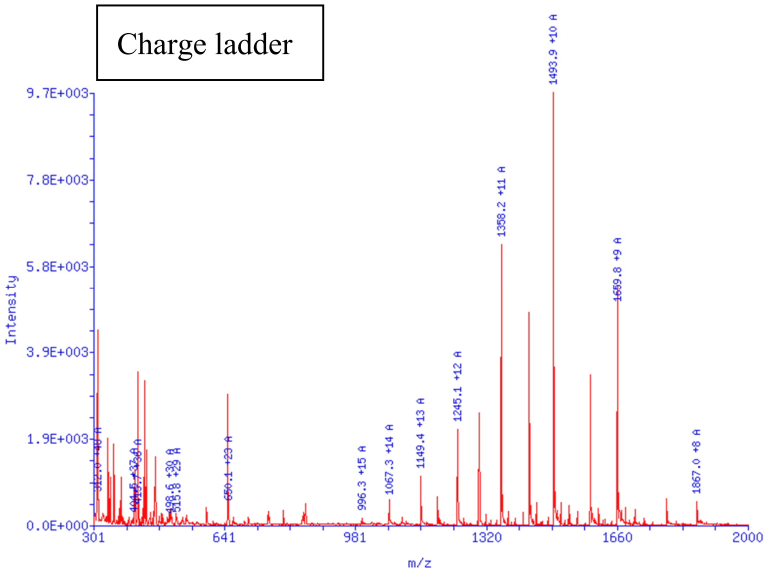

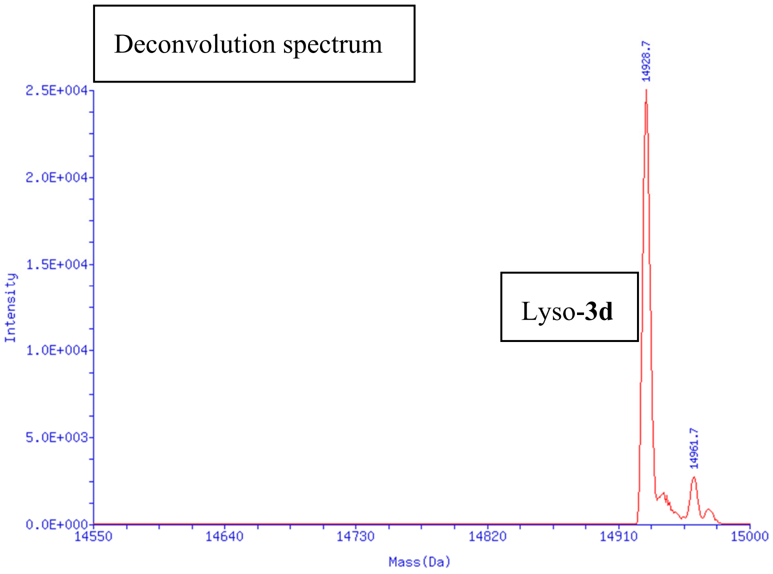

Mass (Da)	Intensity	Score	Delta Mass	%Total
14637.0 (Lyso-**DBTD**)	- not found	-	-	0

14928.7 (Lyso-**3d**)	2.50E+004	10.02	0	90.29
14961.7(Lyso-**DBTD**$_2$)	2.69E+003	3.38	33	9.71

Supplementary Figure 37. LC-MS analysis of Lyso-**DBTD** (4 μM) after the photo-click ligation with DASyd **1d** (100 μM) under irradiation of (**a**) 311 nm or (**b**) 311 + 405 nm for 30 s. Calcd. mass 14929.17 Da (starting material Lyso-**DBTD** adding the nitrile imine derived from DASyd **1d**), found 14928.9 ± 0.15 Da. The yield of Lyso-**DBTD** was 41.9% at 311 nm and 90.3% at 311 + 405 nm (calculated by comparing the ion count of the product to that of the starting material plus product).

Specificity study of photo-activated DASyd 1d toward Lysozyme (Lyso) resolved by LC-MS.

(**a**)

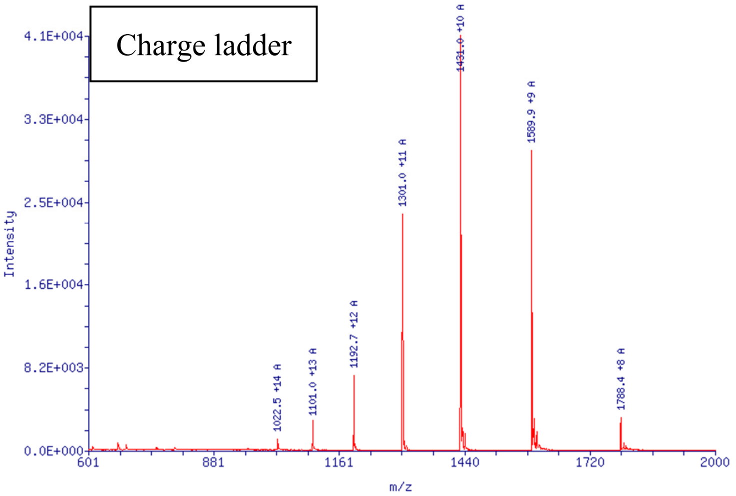

(**b**)

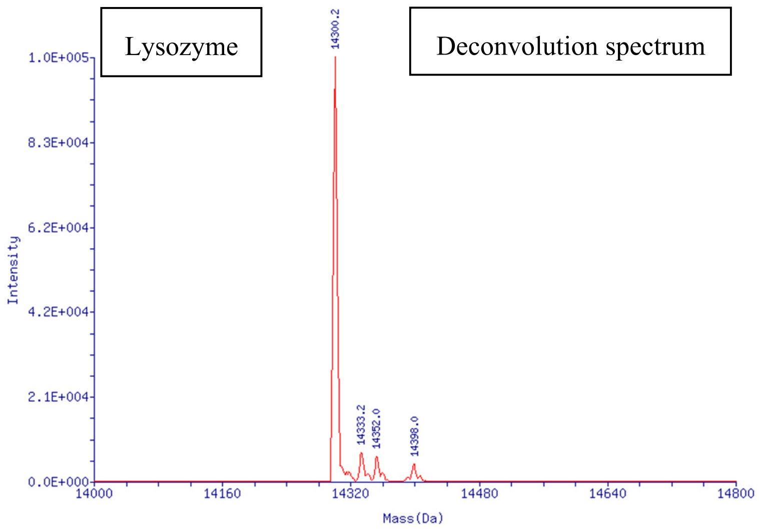

Mass (Da)	Intensity	Score	Delta Mass	%Total
14300.2 (Lyso)	1.04E+005	11.47	0.0	85.76
14333.2	6.96E+003	6.05	33.0	5.74
14352.0	6.07E+003	3.95	51.8	5.01
14398.0	4.22E+003	3.14	97.8	3.48
14592.0 (Lyso+NI)	not found			0

Supplementary Figure 38. LC-MS analysis of Lyso (4 μM) after the photo-click ligation with DASyd **1d** (100 μM) under irradiation of 311 nm for 30 s (calculated by comparing the ion count of the product to that of the starting material plus product). (**a**) The charge ladders of the HPLC-MS spectrum of the reaction mixture. (**b**) The deconvolution MS spectrum. There was no non-specific labeling observed under this irradiation condition.

(**a**)

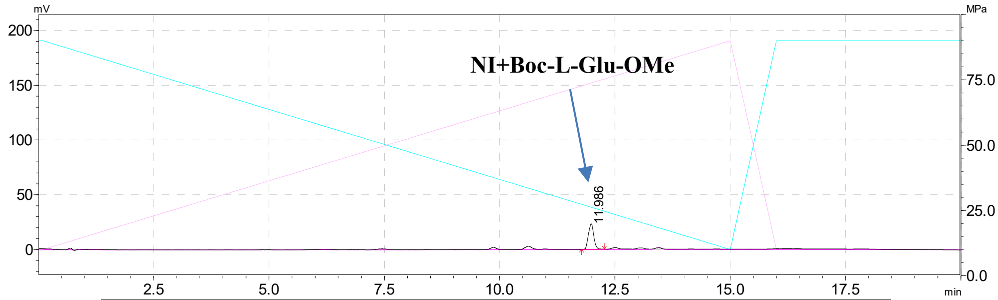

Peak No.	Time	Area	Height	%Area
1	11.986	185162	23128	100

(**b**)

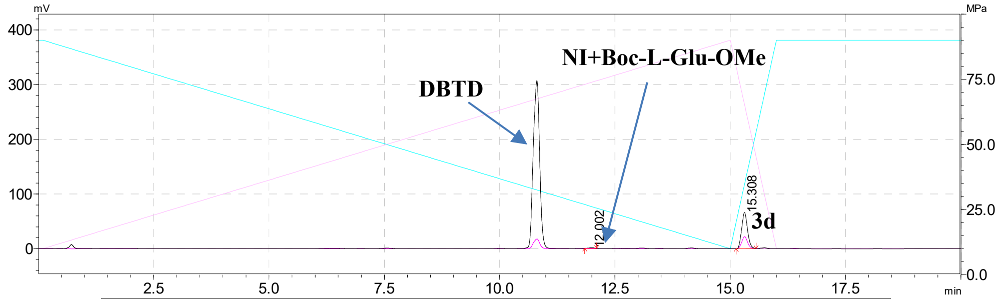

Peak No.	Time	Area	Height	%Area
1	12.002	11267	1562	1.91
2	15.285	579396	65981	98.09

Supplementary Figure 39. HPLC analysis for photo-click reaction of **1d** toward **DBTD** in the presence of carboxylic acid in PB/ACN = 1/1 (PB = phosphate buffer, pH = 7.4). (**a**) HPLC trace of 10 μM **1d**, 5 mM Boc-L-Glu-OMe after the irradiation of 311 + 405 nm for 2 min; (**b**) HPLC trace of 10 μM **1d**, 100 μM **DBTD** and 5 mM Boc-L-Glu-OMe after the irradiation of 311 + 405 nm for 2 min. The product distribution was analyzed by reverse-phase HPLC with absorbance setting at 254 nm for integral area.

Supplementary Note 6. The LC-MS/MS analysis for DASyd 1d photo-ligation toward DBTD-K residue on lysozyme.

After the photo-ligation reaction, the DASyd **1d** labeled protein samples were subjected to in-gel digestion followed by LC−MS/MS analysis (Q Exactive plus, Thermo). The mass spectrometry raw data were analyzed by searching the desired residue fragments using Thermo proteome discoverer. The resulting sequence coverage of lysozyme is 80.27% (near 91.47%, because the first 18 amino acid sequences were derived from the complete translation of the lysozyme gene prior to the post-translational modification. In the actual sample, the lysozyme contains only the polypeptide sequence from residue 19 to 147) that covers all 6 lysine residues except **K19** (5 potentially reactive lysine residues that could be chemically tagged with **DBTD**. The results show that there is a specific lysine residue modified by **DBTD**-NHS. These data indicated that **DBTD**-lysine33 residue (**DBTD**-K51 in the full polypeptide sequence) is in fact the prominently modified site in lysozyme by the photo-click chemistry of DASyd **1d** (see the attached MS/MS spectrum confirming the modified site.

The LC-MS/MS spectra and analysis of the modified peptide covering the key residue-K51

(**a**) Sequence coverage of the MS/MS analysis:

MRSLLILVLC FLPLAALGKV FGRCELAAAM KRHGLDNYRG YSLGNWVCAA KFESNFNTQA TNRNTDGSTD YGILQINSRW WCNDGRTPGS RNLCNIPCSA LLSSDITASV NCAKKIVSDG NGMNAWVAWR NRCKGTDVQA WIRGCRL

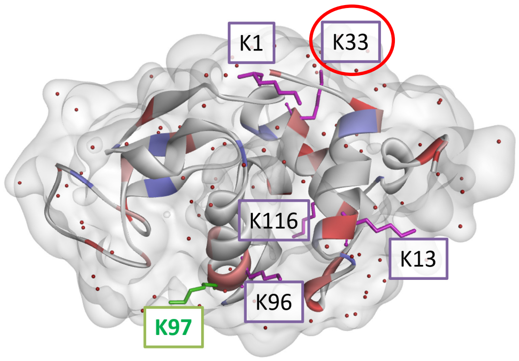

Lysozyme (6 Lys., no free Cys.)
modified residue by **DBTD** and **MAI** observed via photo-click reaction:
the K33 (51–18) residue in red circle

(b)

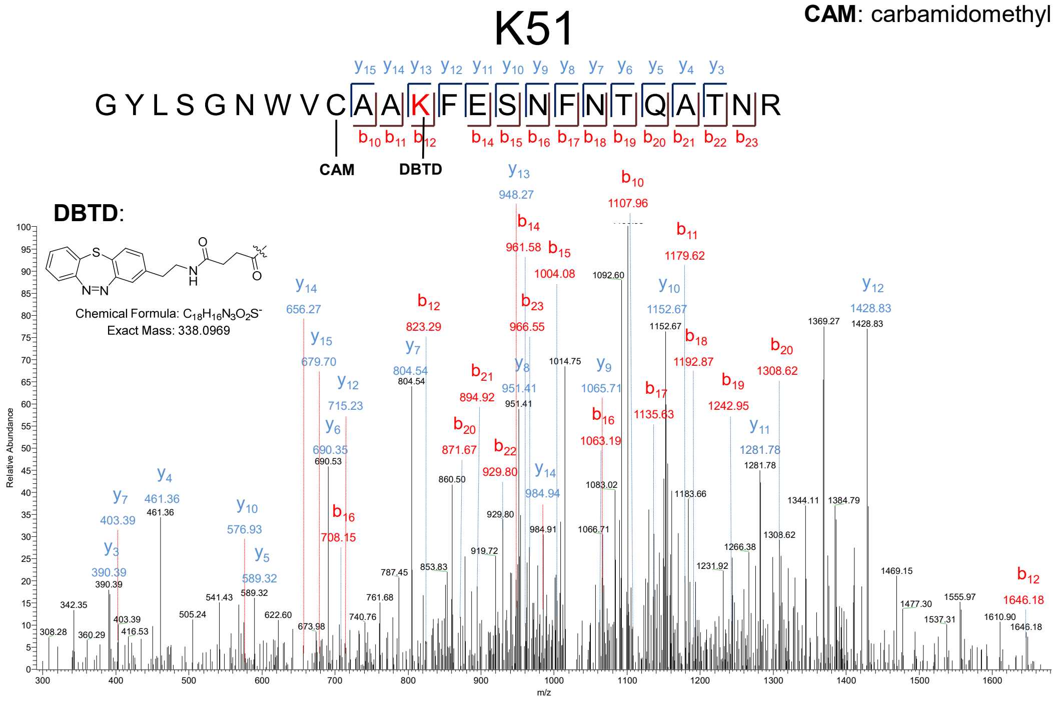

K51
CAM: carbamidomethyl
G Y L S G N W V C A A K F E S N F N T Q A T N R
CAM
DBTD
DBTD:
Chemical Formula: C18H16N3O2S-
Exact Mass: 338.0969
Relative Abundance
m/z

(c)

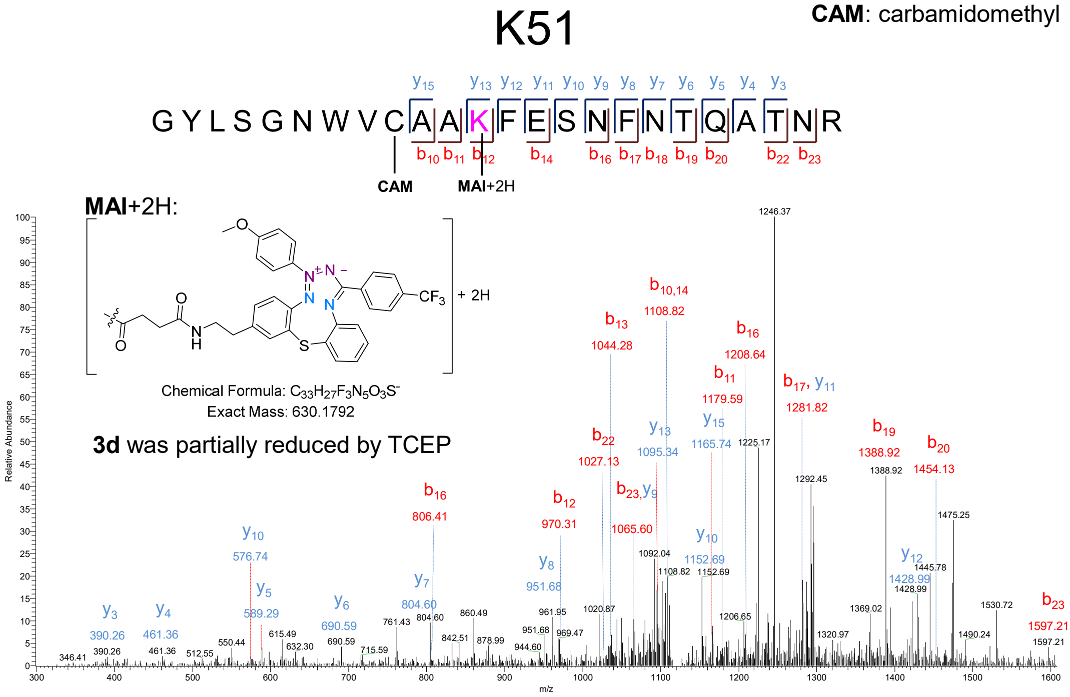

K51
CAM: carbamidomethyl
G Y L S G N W V C A A K F E S N F N T Q A T N R
CAM
MAI+2H
MAI+2H:
+ 2H
Chemical Formula: C33H27F3N5O3S-
Exact Mass: 630.1792
3d was partially reduced by TCEP
Relative Abundance
m/z

Supplementary Figure 40. The labeling of **DBTD**-K residue via the photo-click reaction of DASyd **1d** on lysozyme. (**a**) The illustration of ligation site on the lysozyme residue, K33 (K51 in full sequence), among the 6 potentially reactive Lys. residues displayed via lysozyme crystal structure (PDB ID: 3A3R). The amino acid sequences of lysozyme and the portion of sequence detected in LC-MS/MS analysis was marked in green showing the coverage. (**b**) The LC−MS/MS spectrum for identification of the labeled **DBTD**-K moiety in its peptide fragments with sequence displayed. (**c**) The LC−MS/MS spectrum for identification of the labeled MAI-K moiety through the photo-click chemistry of DASyd **1d** with **DBTD** in its peptide fragments with sequence displayed. (modified residues underlined and labeled with coresponding attachment with proposed structure and the calculated molecular weight).

The stability of **3s** in *E. coli* cell lysate

(**a**)

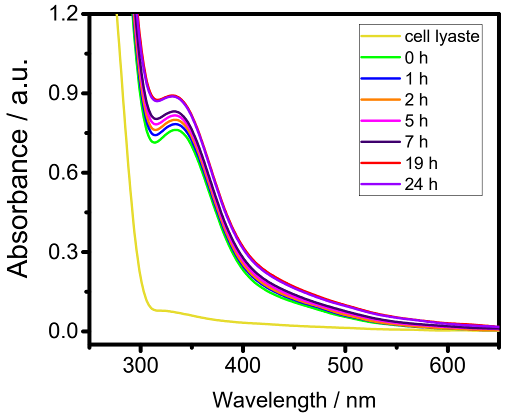

(**b**)

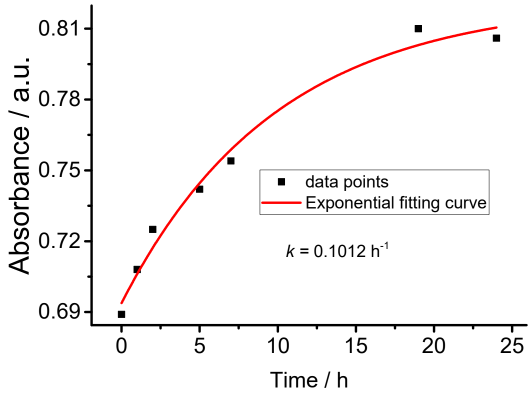

Supplementary Figure 41. The stability study of **3s** in *E. coli* cell lysate monitored by UV-Vis spectra. (**a**) Time course of UV-Vis spectra evolution for the decay of **3s** in cell lysate/ACN =1/2. (**b**) Detailed data points with exponential fitting curve of the absorbance intensity evolution at 340 nm and the first order kinetic ($k_{deacy\text{-}3c}$) was determined to be 0.1012 ± 0.02374 h^{-1}, decaying half-life was 6.9 hours, 298K.

Fluorescence changes after photo-click conjugation of 1g-Cy3 to the DBTD, and subsequent reduction with either GSH or TCEP

(a)

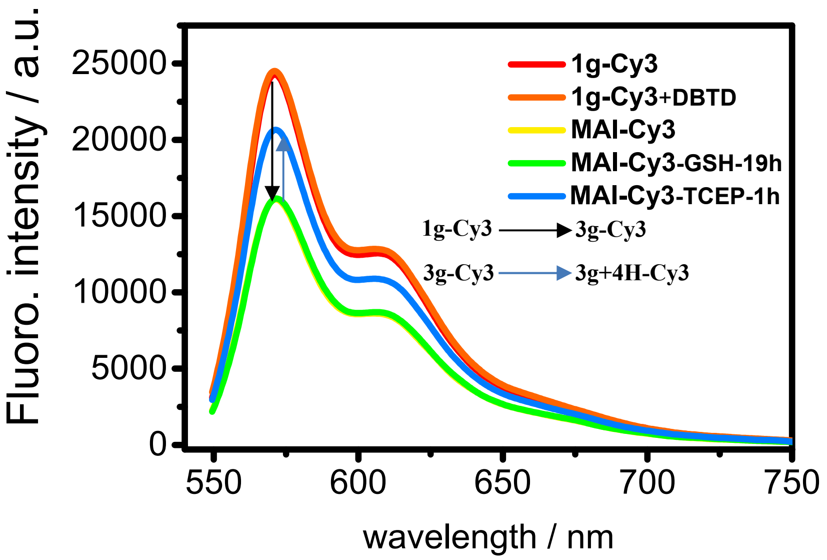

(b)

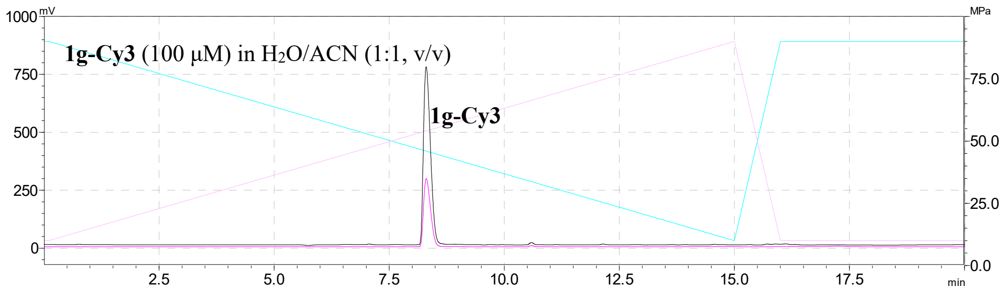

(c)

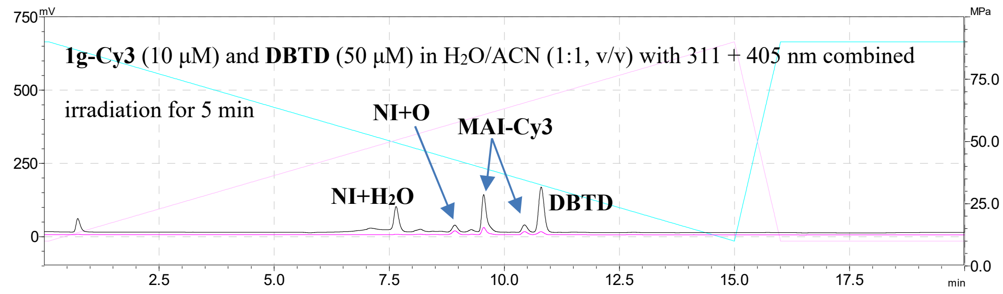

(**d**)

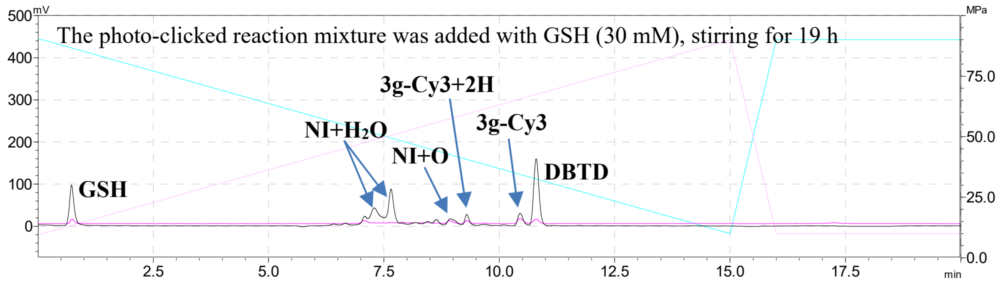

(**e**)

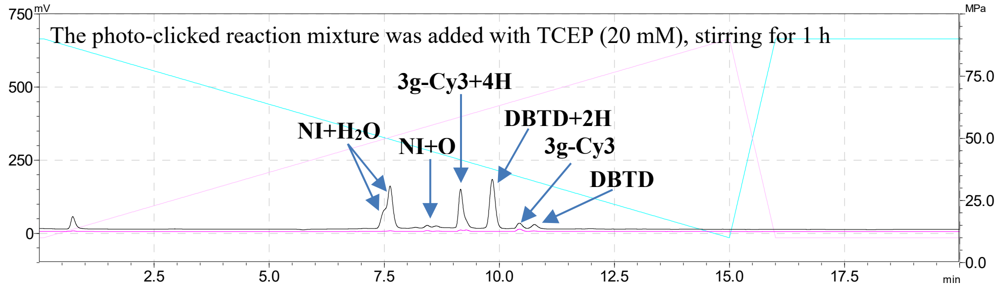

Supplementary Figure 42. The study of fluorescence intensity changes after the photo-ligation. (**a**) The comparison of fluorescence spectra of **1g-Cy3** (10 μM) before, after the photo-click, and addition of GSH or TCEP after photo-irradiation and control **1g-Cy3** without adding the **DBTD**. (**b**) HPLC analysis of **1g-Cy3** (100 μM); (**c**) HPLC analysis of **1g-Cy3** (10 μM) and **DBTD** (50 μM) in H_2O/ACN (1:1, v/v) after 5 min photo-irradiation with 311 + 405 nm combination; (**d**) HPLC analysis of the reaction mixture with GSH (30 mM) for 19 h; (**e**) HPLC analysis of the reaction mixture with TCEP (20 mM) for 1 h.

Chemical modification of Cetuximab (chimeric monoclonal antibody) and Panitumumab (fully human mAb) by DBTD-NHS and FITC.

A solution of Cetuximab or Panitumumab (1 mg/mL) in PBS (pH = 7.4) was mixed with 60 equivalents of **DBTD-NHS** at room temperature and incubated for 3 h. Excess amount of small molecules was removed from the antibody via a protein spin columns (molecular weight cutoff 10 kDa, 5 times exchange with PBS). Then, 5 equivalents FITC was added into the solution of the Cetuximab modified with **DBTD** and incubated for 2 h, then them mixture was fileted again by protein spin columns (molecular weight cutoff 10kD, 5 times exchange with PBS). Finally, the resultant antibody was stored at 4 ºC. The final concentration of the Cetuximab-**DBTD**/FITC was quantified with BCA protein assay kit. For Panitumumab, only **DBTD-NHS** modification was used to form the Panitumumab-**DBTD** conjugate.

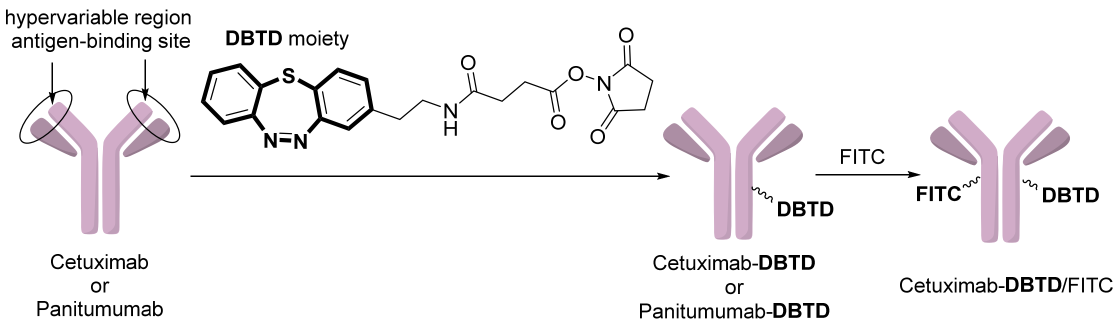

Supplementary Figure 43. Modification of the chimeric Cetuximab (monoclonal antibody, mAb) with 60 eq. of **DBTD-NHS** (20 mM) and 5 eq. FITC (10 mM) to form the bi-conjugated Cetuximab(Cex)-**DBTD**/FITC. Modification of the Panitumumab (mAb) with 60 eq. of **DBTD-NHS** (20 mM) to form the conjugated Panitumumab(Pantu)-**DBTD**.

(a)

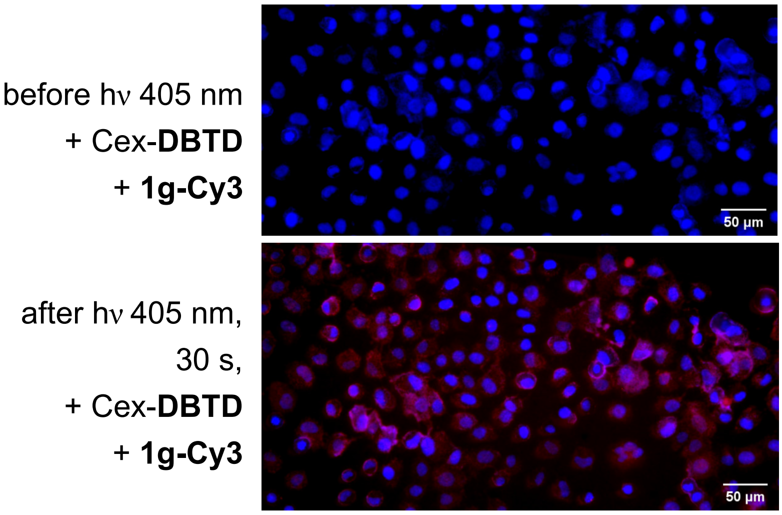
before hν 405 nm
+ Cex-DBTD
+ 1g-Cy3
50 µm
after hν 405 nm,
30 s,
+ Cex-DBTD
+ 1g-Cy3
50 µm

(b)

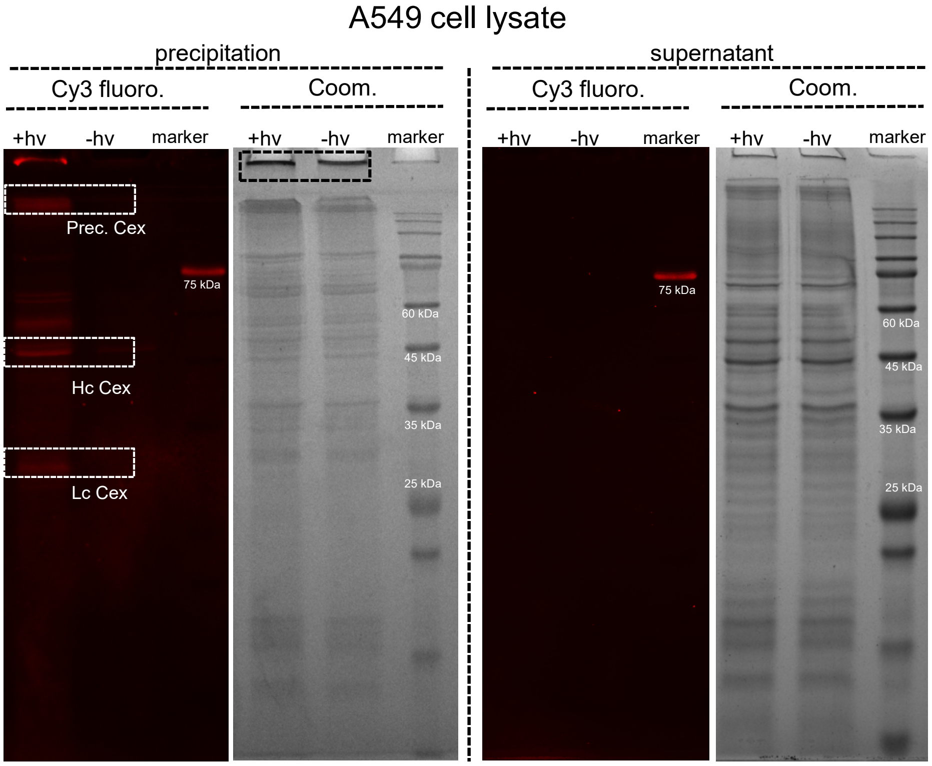
A549 cell lysate
precipitation
supernatant
Cy3 fluoro.
Coom.
Cy3 fluoro.
Coom.
+hv
-hv
marker
Prec. Cex
Hc Cex
Lc Cex
75 kDa
60 kDa
45 kDa
35 kDa
25 kDa

Supplementary Figure 44. (**a**) Fluorescence turn-on before and after irradiation of the single 405 nm LED array on live cells surface. (**b**) The SDS-PAGE images of the supernatant and precipitation of the A549 cell lysate. The fluorescence of either the heave or the light chains of the labeled Cetuximab-MAI were found in the left gel showing in-gel fluorescence (as marked in the dash lined rectangular for different composition, the Cetuximab was bound to EGFR embedded through the cytomembrane of the cells. After lysis, the majority of the Cetuximab were still bound to form the precipitation, making it difficult to migrate in SDS-PAGE. However, small fraction of the antibody fragment could be detected in the in-gel assay). On the other hand, the supernatant of A549 cell lysate contains few free antibodies, and there is almost no fluorescence signal of Cetuximab detected.

Spatiotemporally resolved fluorescence labeling of the live A549 or A431 cells.

(a)

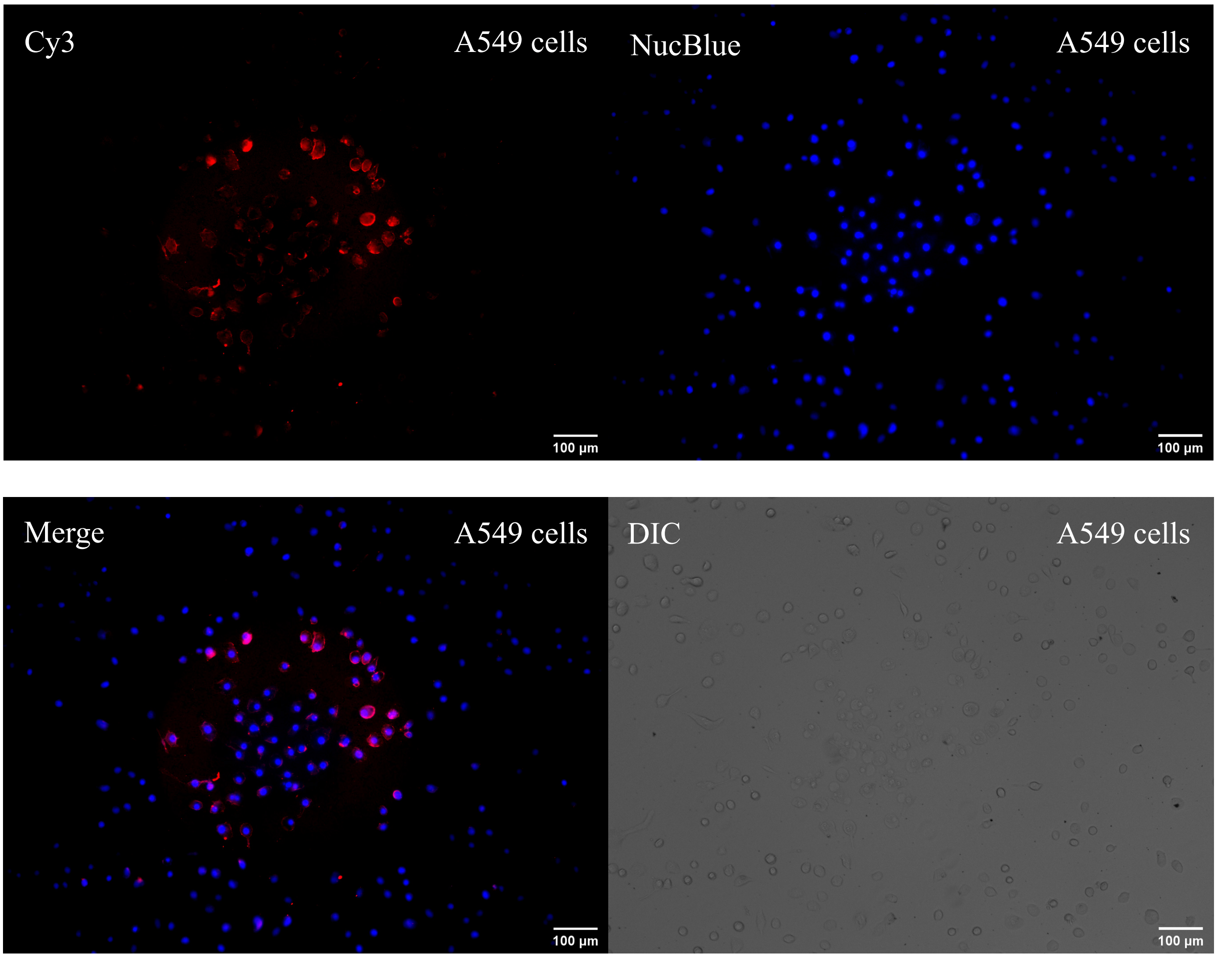

(b)

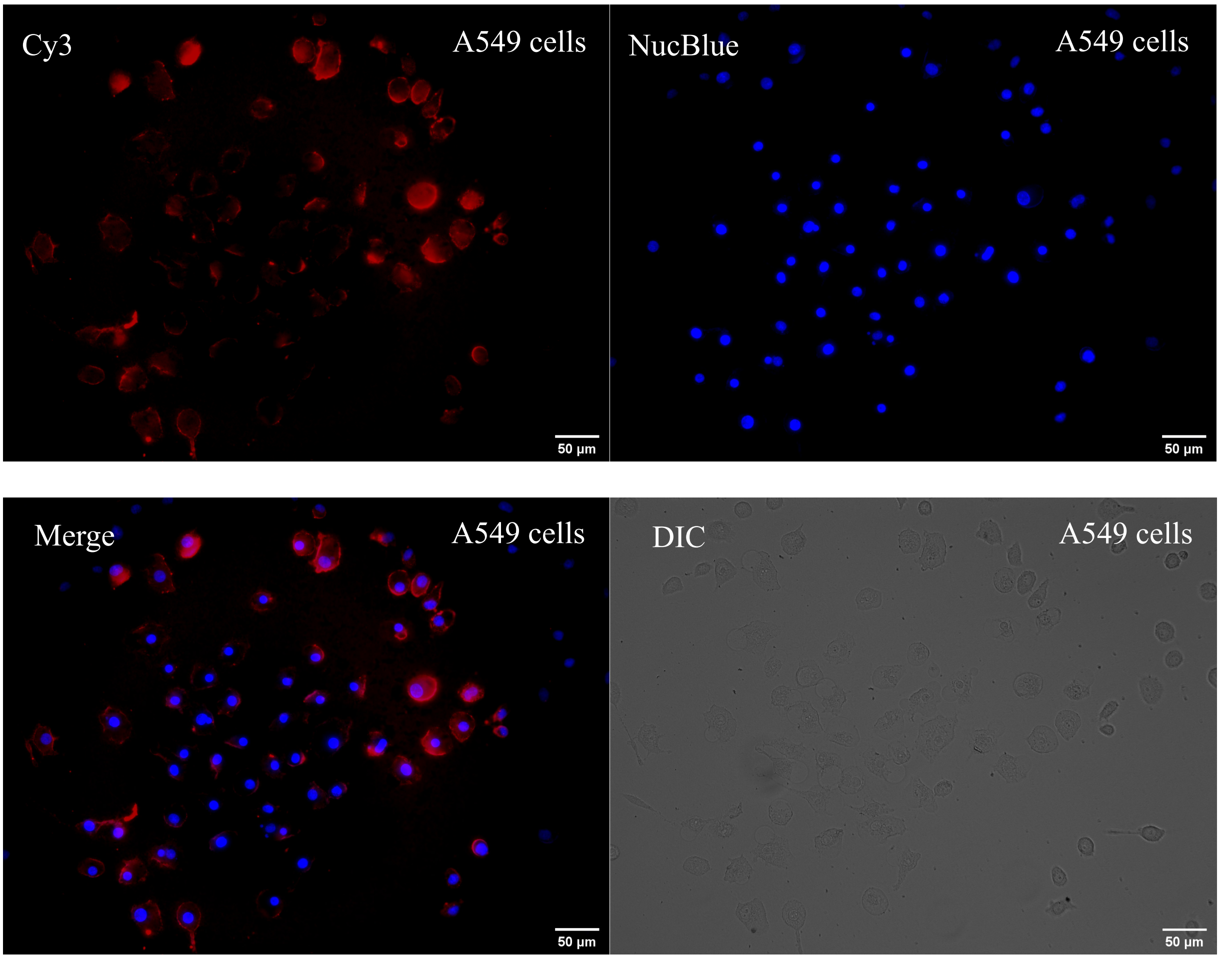
Cy3
A549 cells
50 μm
NucBlue
A549 cells
50 μm
Merge
A549 cells
50 μm
DIC
A549 cells
50 μm

(**c**)

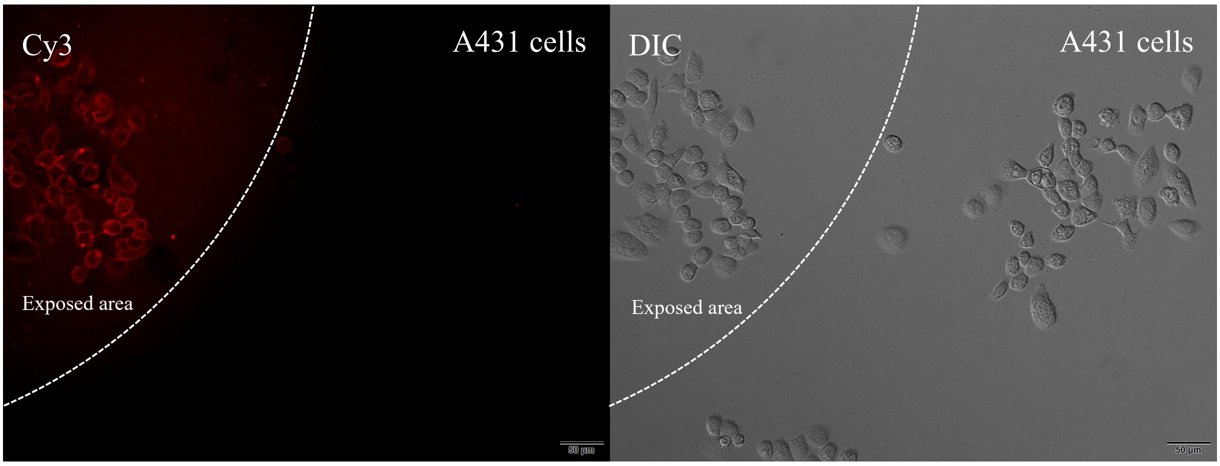

Supplementary Figure 45. Spatially resolved fluorescence labeling of the live A549 cells via 390 nm light induced DASyd-DBTD ligation for 3 min. The pretreated (Cetuximab-**DBTD**) live cells in culture medium supplied with **1g-Cy3** were exposed to embedded excitation light source (390 nm) via the 40× objective. The live cell imaging with objective lens in various magnification at the same location of interest: (**a**) imaged with a 10× objective, (**b**) imaged with a 20× objective. (**c**) Spatially resolved fluorescence labeling of the live A431 cells via 390 nm light induced DASyd-DBTD ligation for 3 min (cells were pretreated with Panitumumab-**DBTD**), imaged with a 20× objective.

Cell viability of A549 and A431 cells after incubation with the **DBTD**.

(**a**)

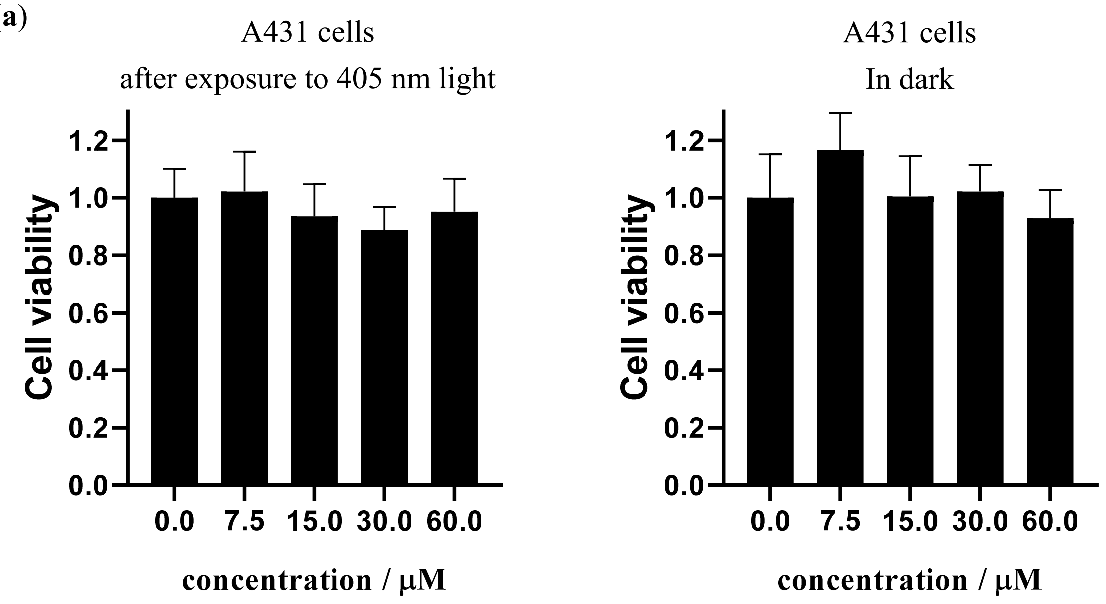

(**b**)

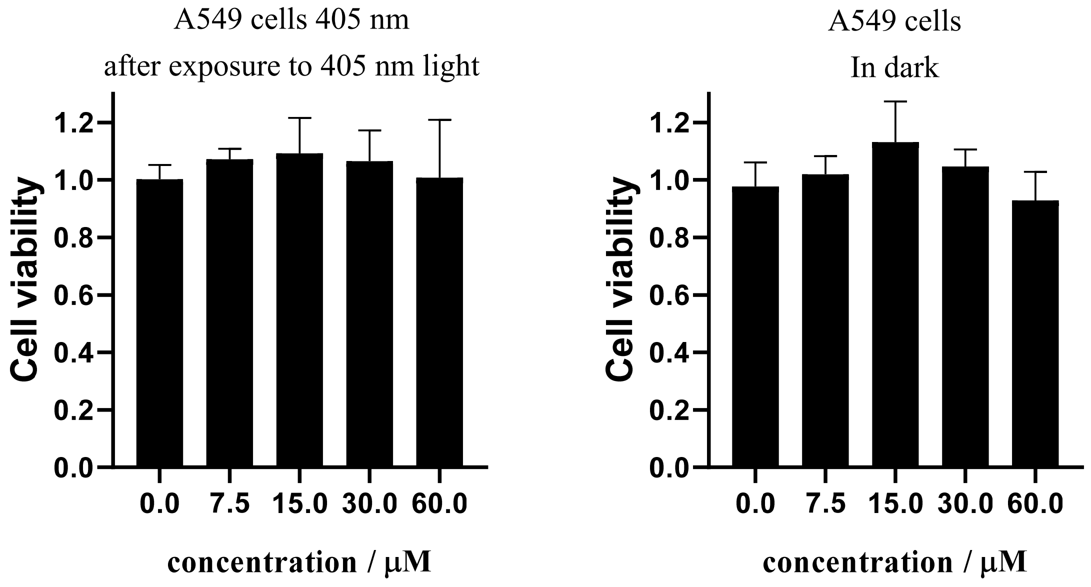

Supplementary Figure 46. Cell viability of A549 and A431 cells after incubation with the **DBTD** in dark or stimulated with the 405 nm LED array for 30s. (**a**) The A431 cells were incubated with 7.5-60 μM **DBTD** for 24 hours. The CCK-8 assay was used to assess the cell

viability. Data were shown as mean ± SEM (n = 5). (**b**) The A431 cells were incubated with 7.5-60 μM **DBTD** for 24 hours. The CCK-8 assay was used to assess the cell viability. Data were shown as mean ± SEM (n = 5). Error bars denote standard deviation from five experimental replicates.

Supplementary Table 2. Crystal data and structure refinement for **3e**.

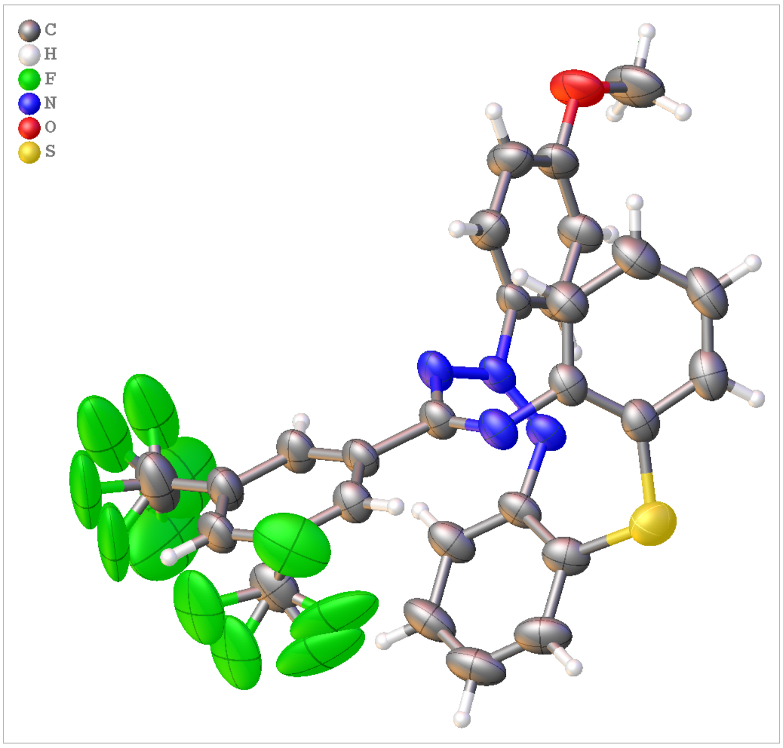

Identification code	180511_s2_gjs	
Empirical formula	$C_{28}H_{18}F_6N_4OS$	
Formula weight	572.52	
Temperature	293.15 K	
Crystal system	monoclinic	
Space group	C2/c	
Unit cell dimensions	*a* = 25.630(2) Å	α = 90 °
	b = 7.8333(5) Å	β = 111.329(10) °
	c = 27.839(2) Å	γ = 90 °
Volume	5206.2(8) $Å^3$	
Z	8	
Density (calculated)	1.461 g cm^{-3}	
Absorption coefficient	0.197 mm^{-1}	
F (000)	2336.0	
Crystal size	0.35 x 0.3 x 0.2 mm^3	
Radiation	MoKα (λ = 0.71073)	

Theta range for data collection	5.864 to 52.744°
Index ranges	$-22 \leq h \leq 32$, $-9 \leq k \leq 9$, $-32 \leq l \leq 34$
Reflections collected	11181
Independent reflections	5317 [Rint = 0.0304, Rsigma = 0.0531]
Data / restraints / parameters	5317 / 6 / 380
Goodness-of-fit on F^2	1.018
Final R indices [I>2 sigma (I)]	R_1 = 0.0696, wR_2 = 0.1679
Final R indices (all data)	R_1 = 0.0865, wR_2 = 0.1976
Largest diff. peak and hole	0.37 and -0.36 e. Å^{-3}

Single crystal of **3e** [$C_{28}H_{18}F_6N_4OS$] was obtained by recrystallization in hexane/EtOAc. CCDC-1905280 (CIF) contains the supplementary crystallographic data which can be obtained free of charge from Cambrige Crystallographic Data Centere *via* www.ccdc.cam.ac.uk/data_request/cif.

Supplementary Table 3. Crystal data and structure refinement for (*Z*)-**DBTD**.

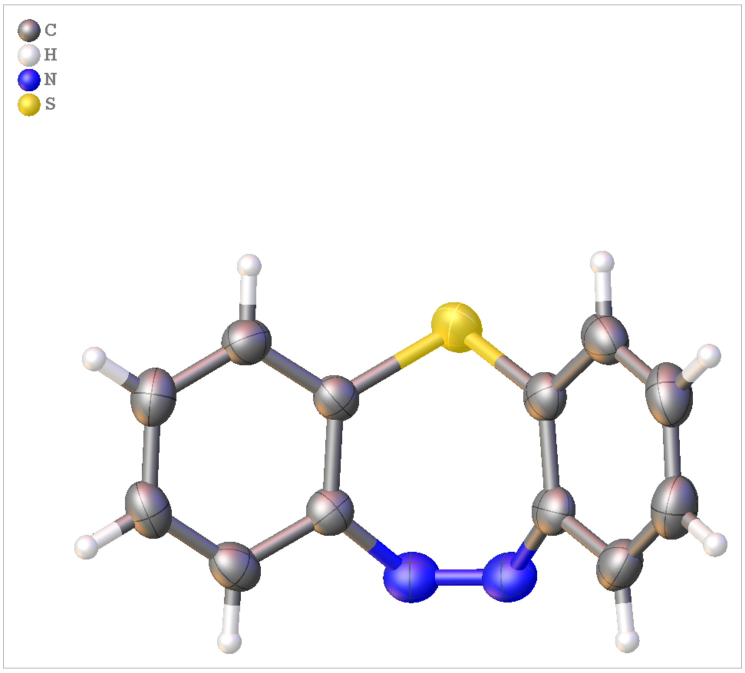

Identification code	180917_s3_gjs	
Empirical formula	$C_{12}H_8N_2S$	
Formula weight	212.26	
Temperature	293.15 K	
Crystal system	monoclinic	
Space group	$P2_1/n$	
Unit cell dimensions	a = 5.8600(5) Å	α = 90 °
	b = 12.4535(12) Å	β = 91.561(7) °
	c = 13.6689(10) Å	γ = 90 °
Volume	997.16(14) Å^3	
Z	4	
Density (calculated)	1.414 g cm^{-3}	
Absorption coefficient	0.286 mm^{-1}	
F (000)	440.0	
Crystal size	0.4 x 0.4 x 0.35 mm^3	
Radiation	MoKα (λ = 0.71073)	
2Θ range for data collection	5.964 to 58.738°	

Index ranges	$-8 \le h \le 5$, $-15 \le k \le 10$, $-11 \le l \le 17$
Reflections collected	4596
Independent reflections	2293 [Rint = 0.0178, Rsigma = 0.0343]
Data / restraints / parameters	2293/0/136
Goodness-of-fit on F^2	1.033
Final R indices [I>2 sigma (I)]	$R_1 = 0.0409$, $wR_2 = 0.0898$
Final R indices (all data)	$R_1 = 0.0611$, $wR_2 = 0.1028$
Largest diff. peak and hole	0.17 and -0.29 e. Å^{-3}

Single crystal of (*Z*)-**DBTD** [$C_{12}H_8N_2S$] was obtained by recrystallization in hexane/EtOAc. CCDC-1905279 (CIF) contains the supplementary crystallographic data which can be obtained free of charge from Cambrige Crystallographic Data Centere *via* www.ccdc.cam.ac.uk/data_request/cif.

Supplementary Reference

1. Zhang, L.; Zhang, X.; Yao, Z.; Jiang, S.; Deng, J.; Li, B.; Yu, Z. Discovery of Fluorogenic Diarylsydnone-Alkene Photoligation: Conversion of *ortho*-Dual-Twisted Diarylsydnones into Planar Pyrazolines. *J. Am. Chem. Soc.* **2018**, *140*, 7390-7394.
2. Allinger, N. L.; Youngdale, G. A. Aromatic and Pseudoaromatic Non-benzenoid Systems. III. The Synthesis of Some Ten π-Electron Systems[1-3]. *J. Am. Chem. Soc.* **1962**, *84*, 1020-1026.
3. Chen, J.; Zhao, K.; Ge, B.; Xu, C.; Wang, D.; Ding, Y. Iridium-Catalyzed Synthesis of Diaryl Ethers by Means of Chemoselective C-F Bond Activation and the Formation of B-F Bonds. *Chem. Asian J.* **2015**, 10, 468-473.
4. Kuhn, H. J.; Braslavsky, S. E.; Schmidt, R. Chemical Actinometry. *Pure & Appl. Chem.* **1989**, *61*, 187−210.
5. Calvert, J. G.; Pitts, J. N. Jr. Photochemistry, John Wiley, New York, **1967**, pp 781.
6. Stadler, E.; Eibel, A.; Fast, D.; Freißmuth, H.; Holly, C.; Wiech, M.; Moszner, N.; Gescheidt, G. A versatile method for the determination of photochemical quantum yields via online UV-Vis spectroscopy. *Photochem. Photobiol. Sci.* **2018**, *17*, 660-669.
7. Ginn, B. T.; Steinbock, B.; Kahveci, M.; Seinbock O. Microfluidic Systems for the Belousov-Zhabotinsky Reaction. *J. Phys. Chem. A* **2004**, *108*, 1325-1332.
8. Deniel, M. H.; Lavabre, D. and Micheau J. C. in *Organic Photochromic and Thermochromic Compounds*, Vol. 2 (Eds.: J. C. Crano, R. J. Guglielmetti) Kluwer Academic / Plenum Publishers, New York, **1999**, pp. 167-177.
9. Inagaki, Y.; Kobayashi, Y.; Mutoh, K.; Abe, J. A Simple and Versatile Strategy for Rapid Color Fading and Intense Coloration of Photochromic Naphthopyran Families. *J. Am. Chem. Soc.* **2017**, *139*, 13429−13441.

^{1}H, ^{19}F and ^{13}C NMR Spectra

^{1}H NMR

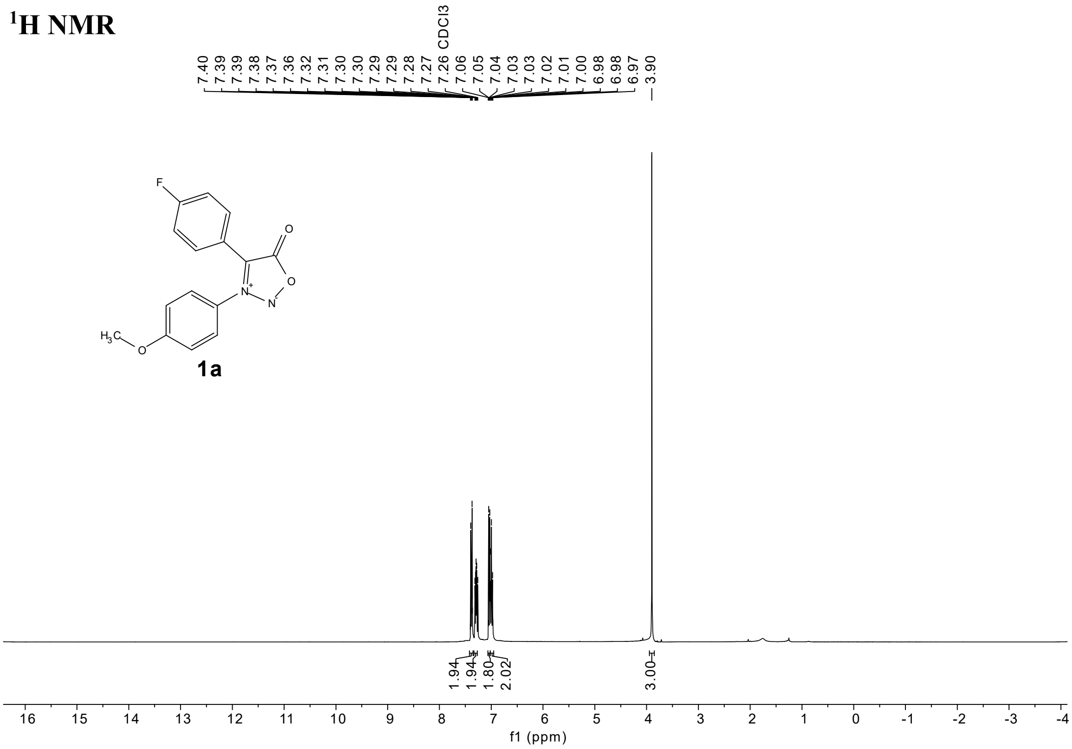

^{13}C NMR

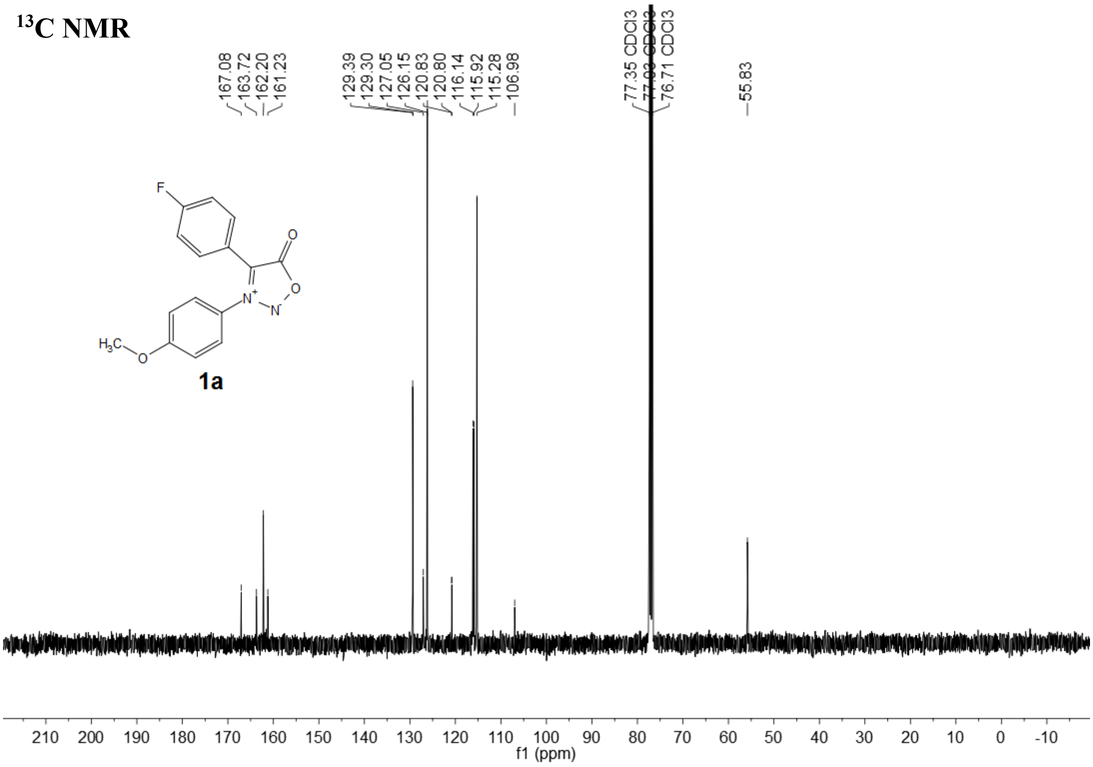

Supplementary Figure 47 ^{1}H and ^{13}C NMR spectra of Compound **1a**.

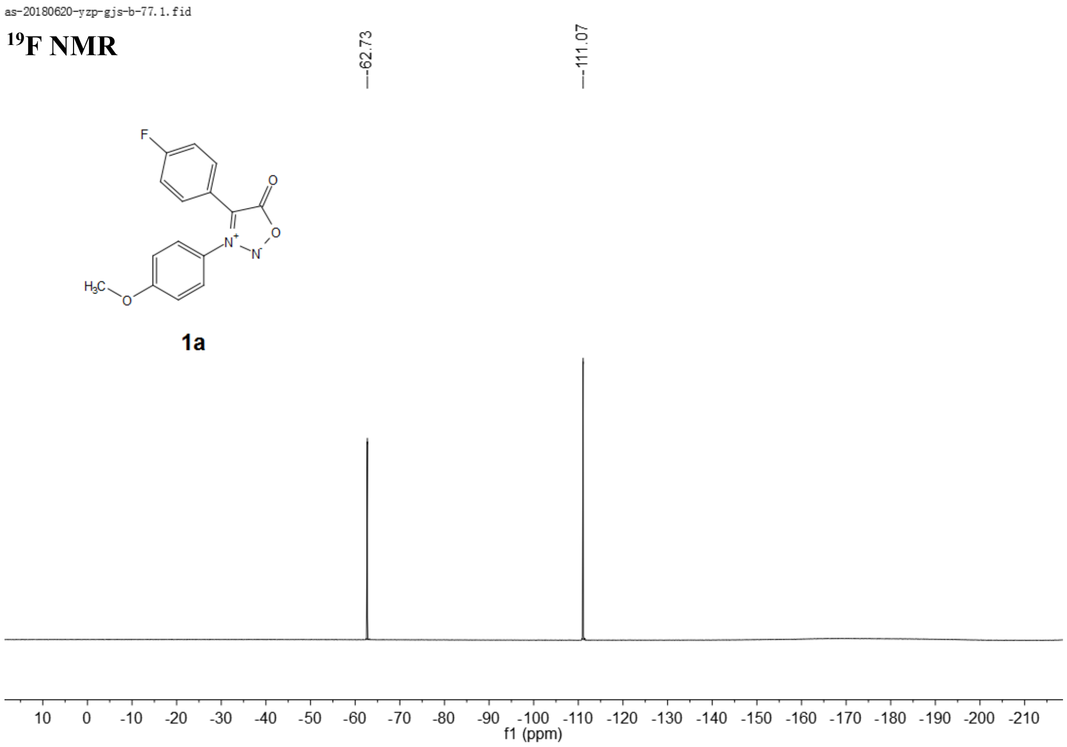

Supplementary Figure 48 ^{19}F NMR spectra of Compound **1a**.

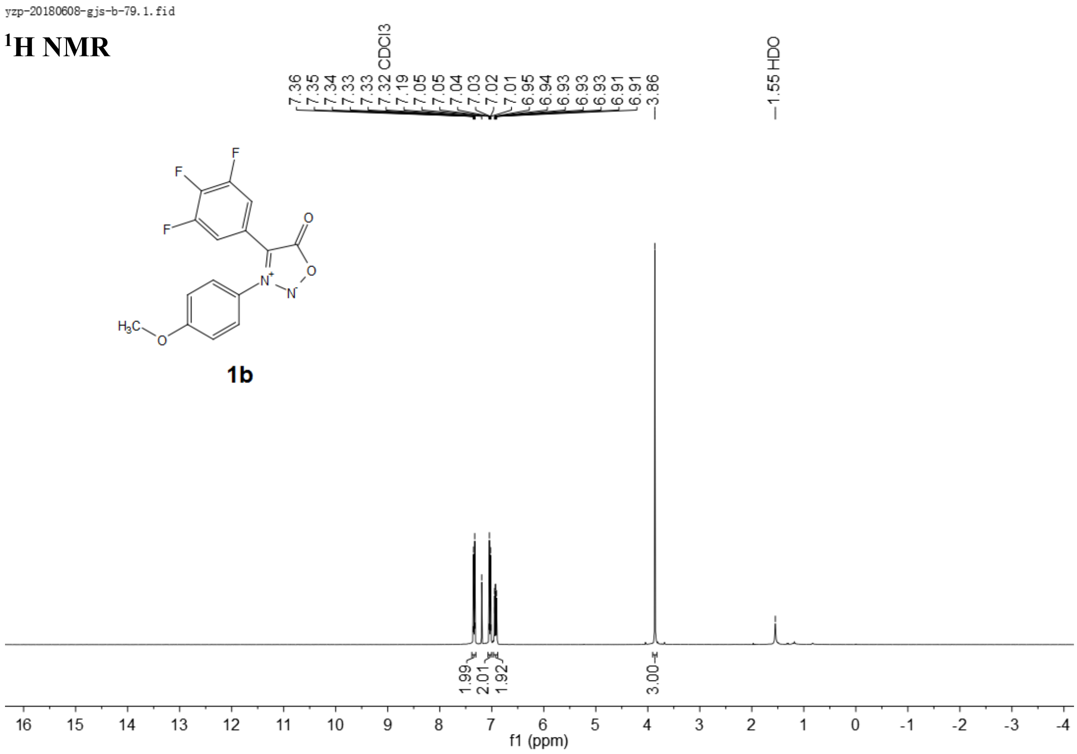

Supplementary Figure 49 ^{1}H NMR spectra of Compound **1b**.

^{13}C NMR

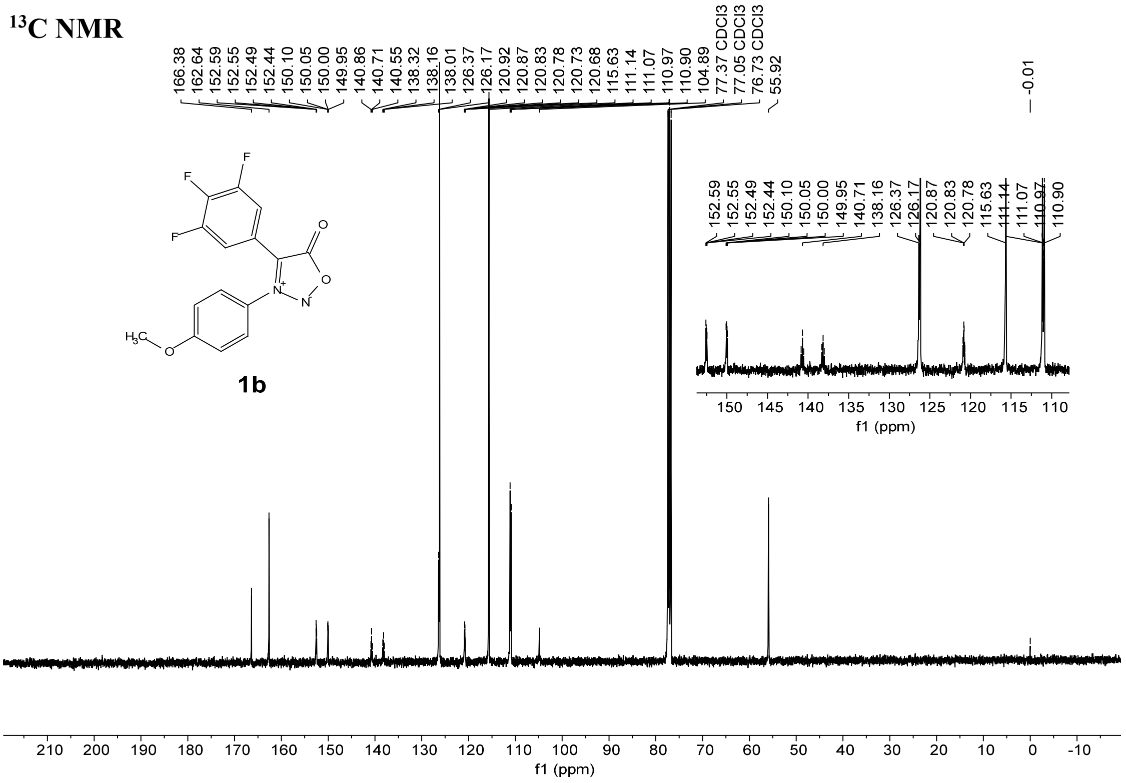

^{19}F NMR

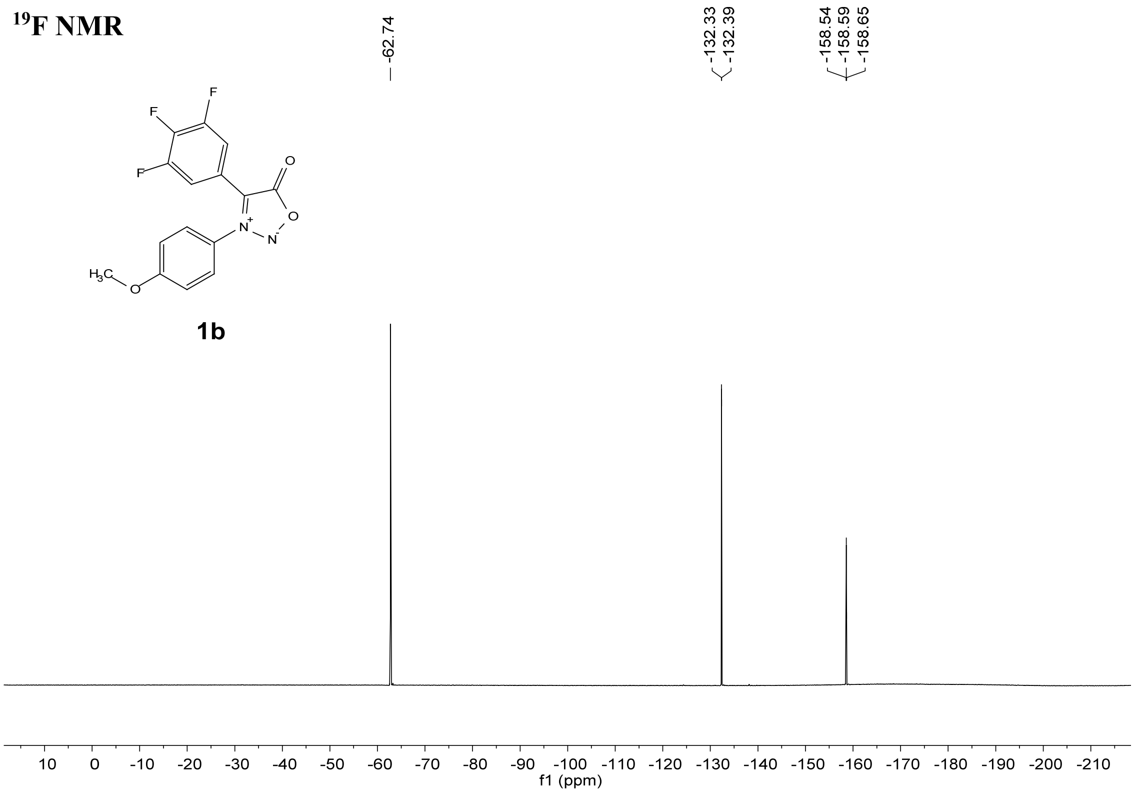

Supplementary Figure 50 ^{13}C NMR and ^{19}F NMR spectra of Compound **1b**.

^{1}H NMR

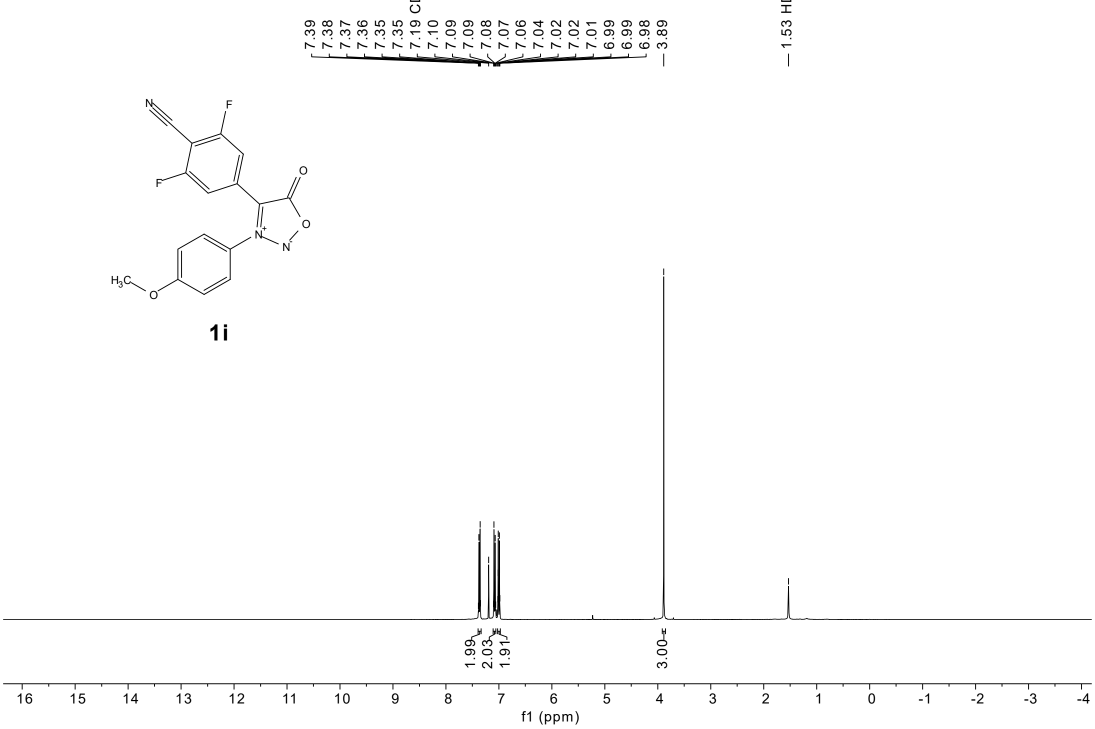

^{13}C NMR

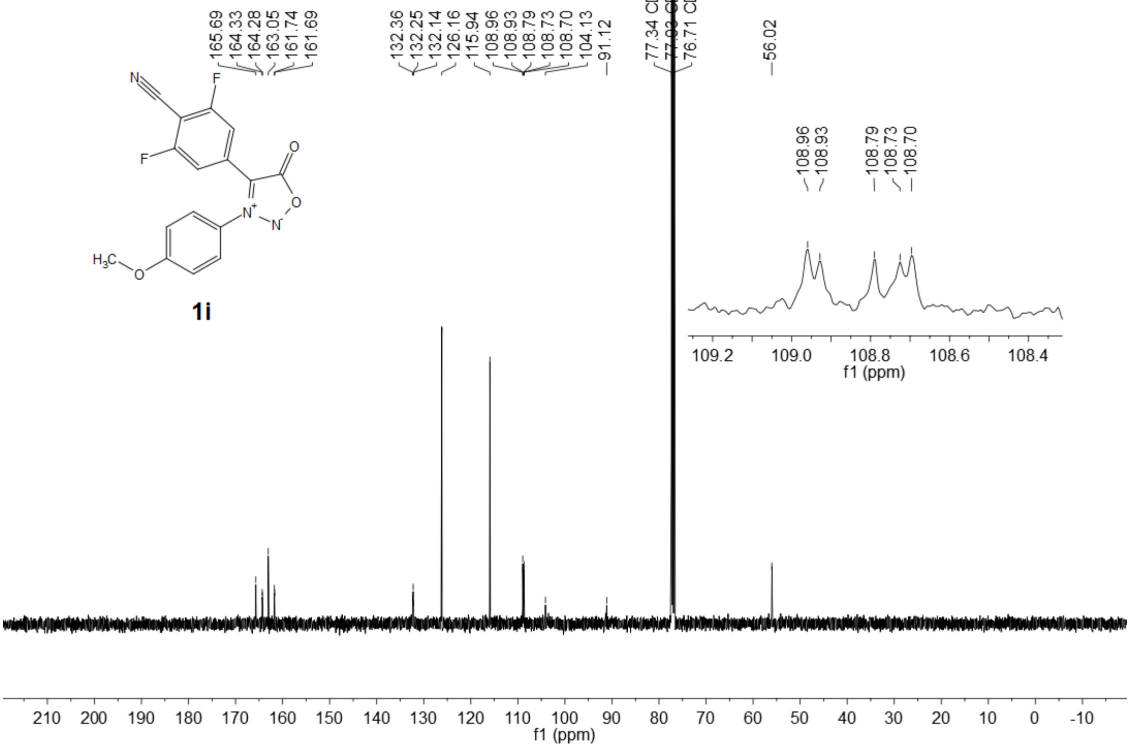

Supplementary Figure 51 ^{1}H NMR and ^{13}C NMR spectra of Compound **1i**.

^{19}F NMR

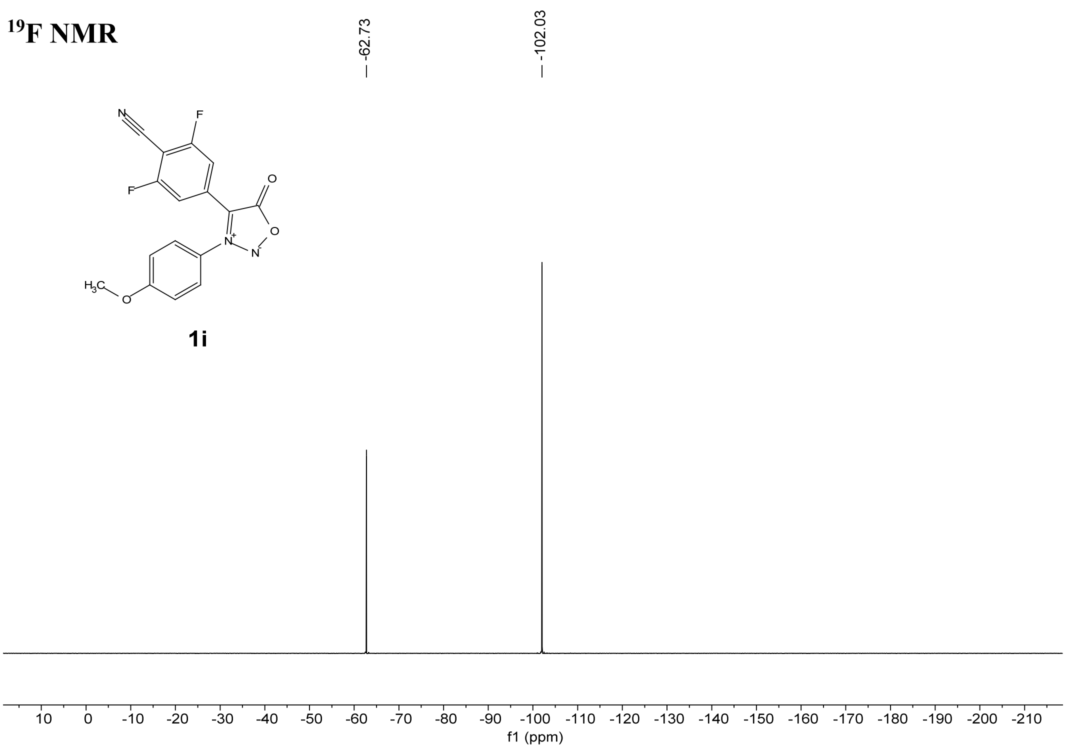

Supplementary Figure 52 ^{19}F NMR spectra of Compound **1i**.

^{1}H NMR

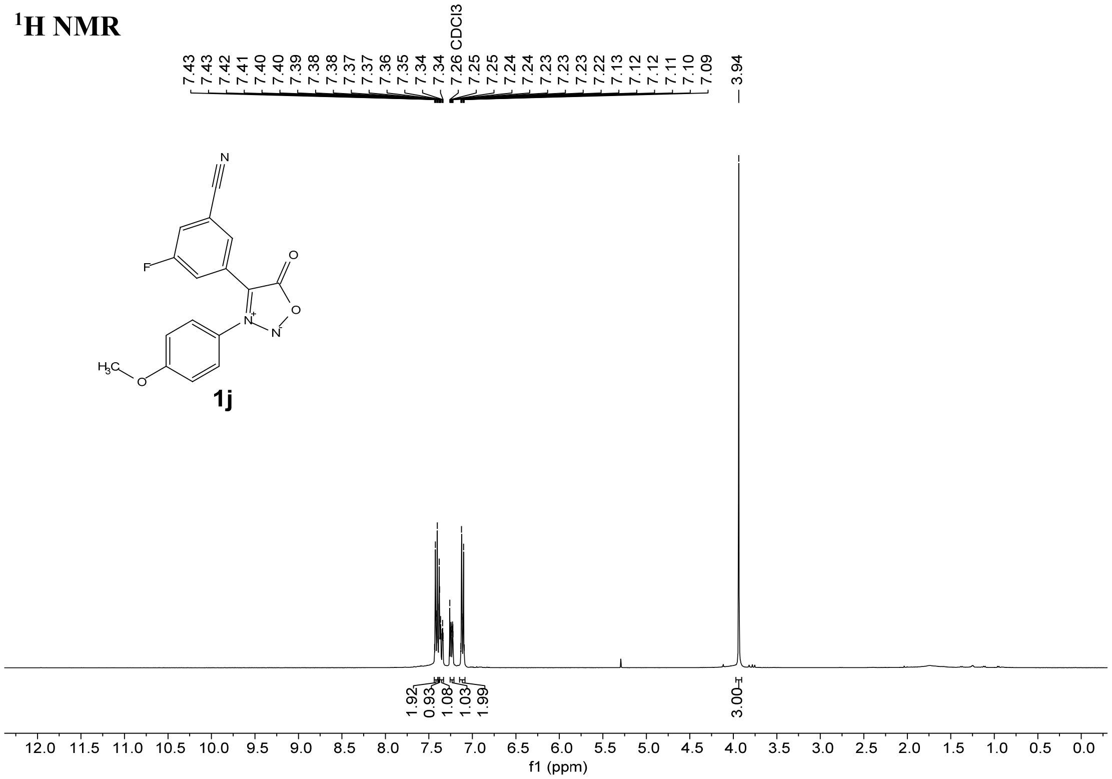

Supplementary Figure 53 ^{1}H NMR spectra of Compound **1j**.

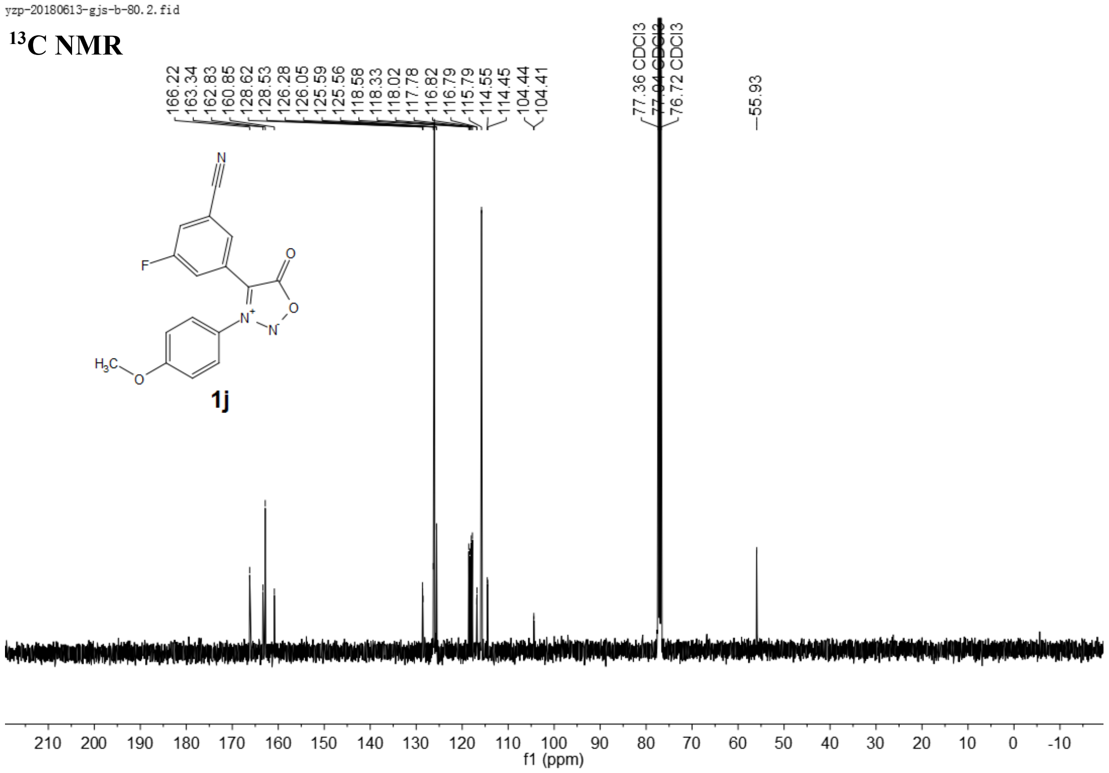

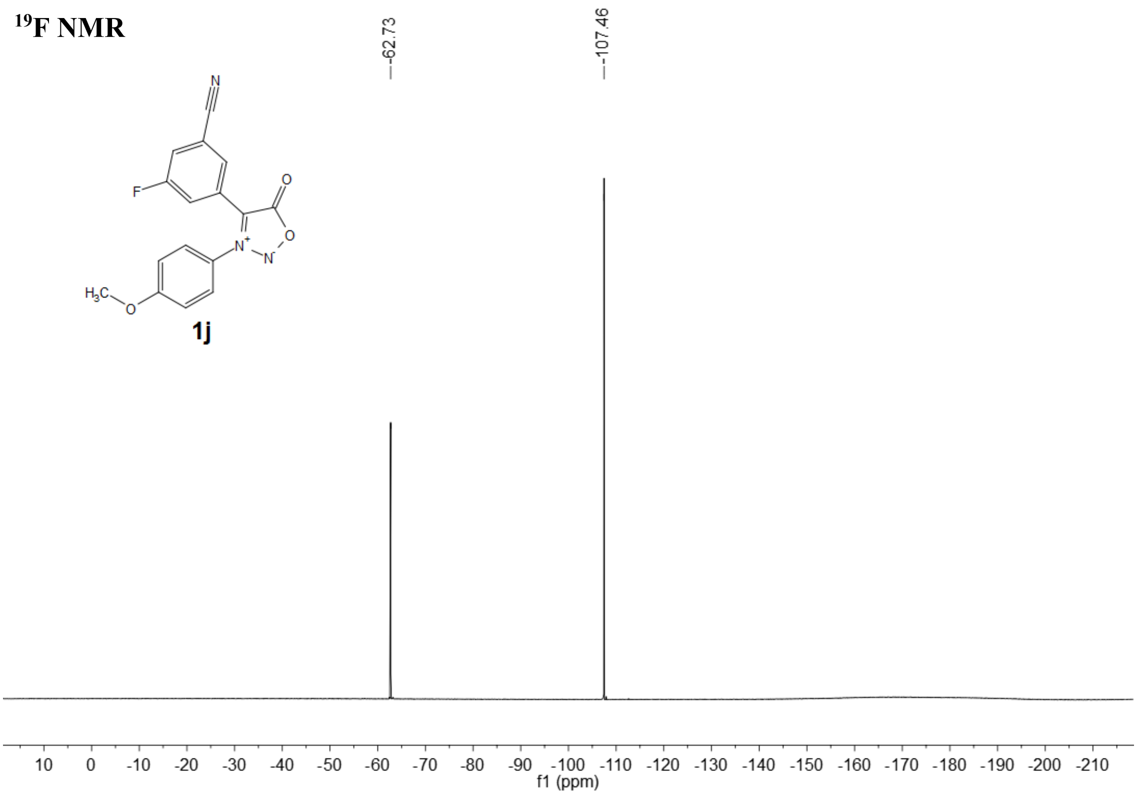

Supplementary Figure 54 ^{13}C NMR and ^{19}F NMR spectra of Compound **1j**.

yzp-20190219-gjs-b-66.2.fid

^{1}H NMR

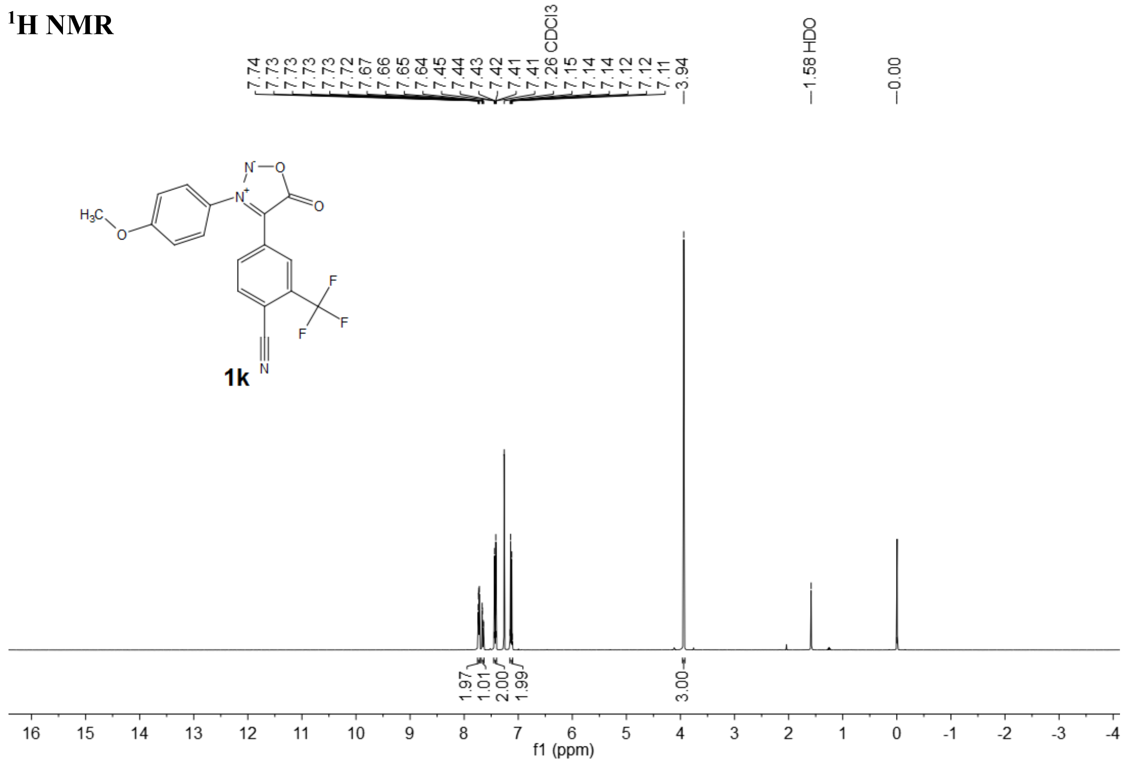

^{13}C NMR

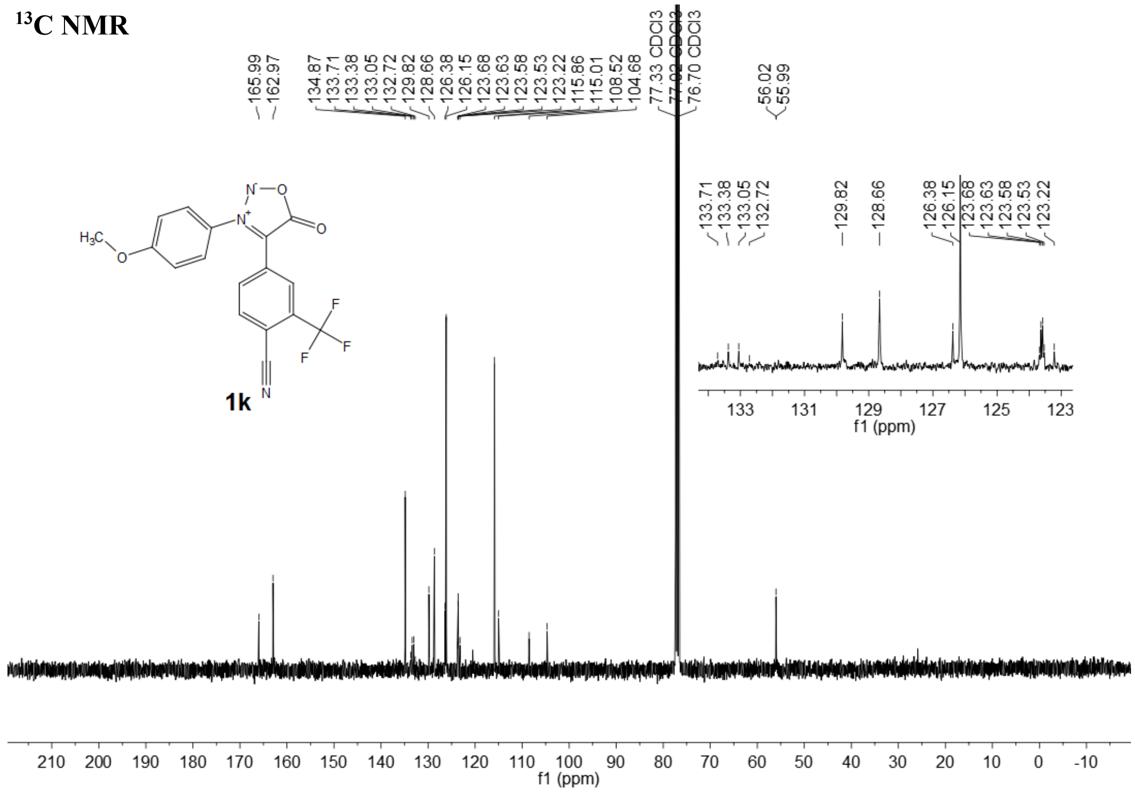

Supplementary Figure 55 ^{1}H NMR and ^{13}C NMR spectra of Compound **1k**.

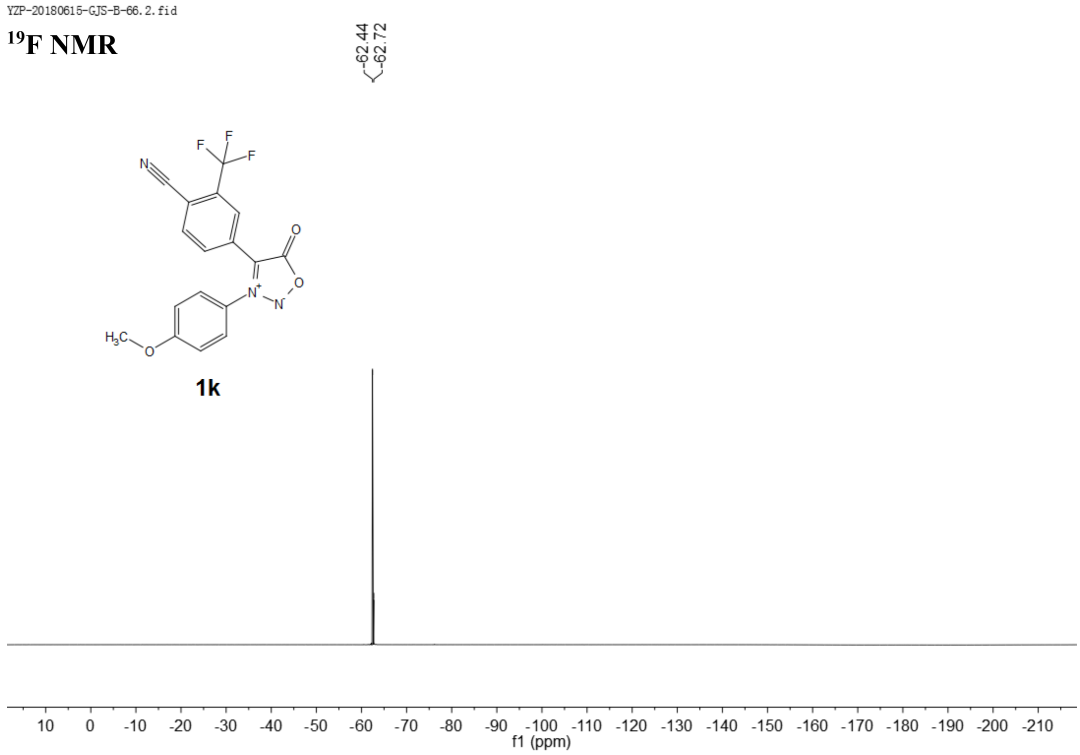

Supplementary Figure 56 ^{19}F NMR spectra of Compound **1k**.

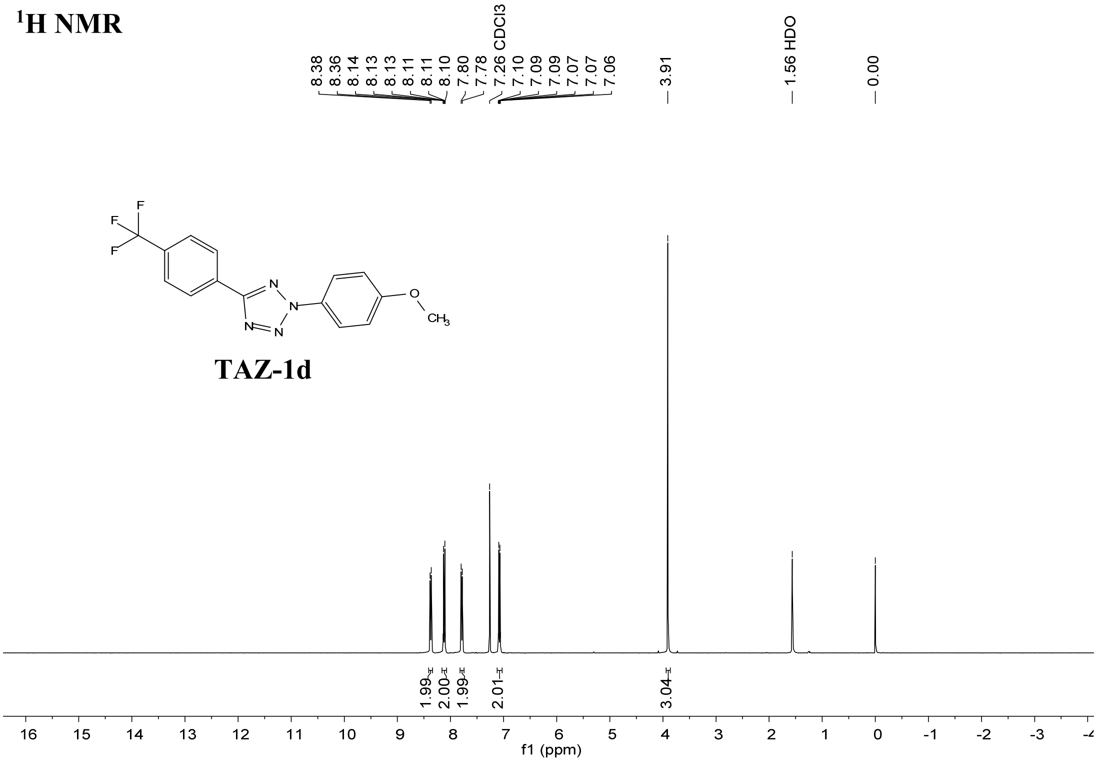

Supplementary Figure 57 ^{1}H NMR spectra of Compound **TAZ-1d**.

^{13}C NMR

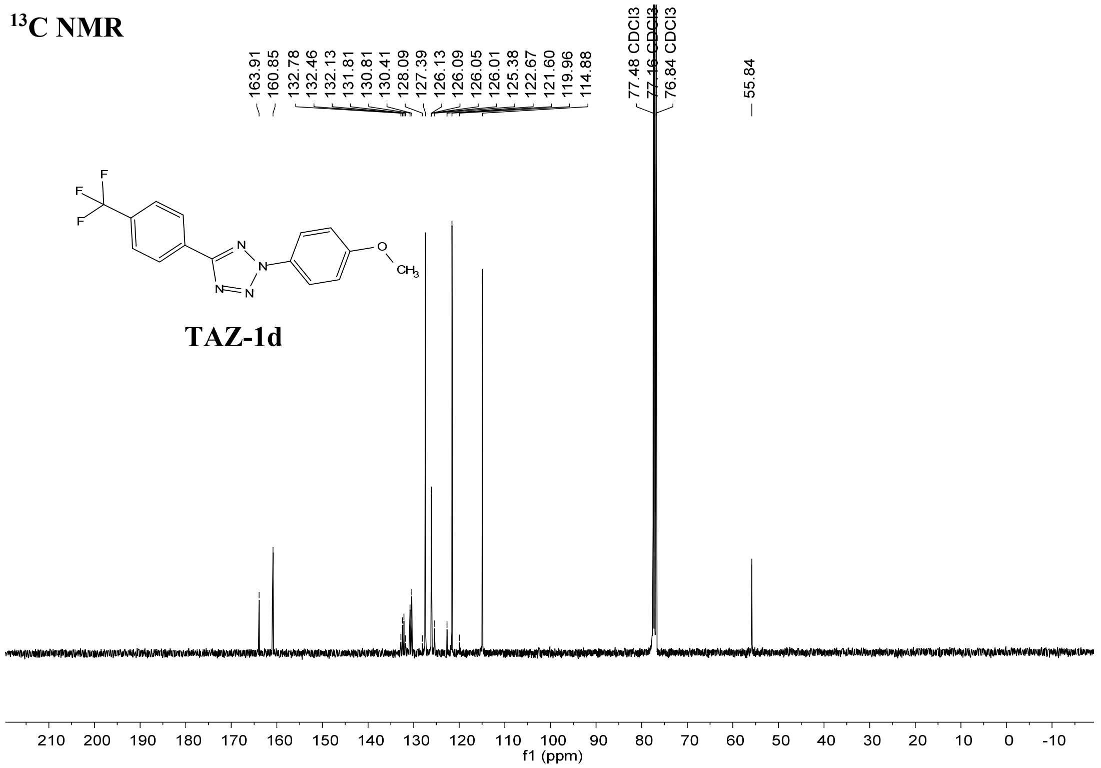

^{19}F NMR

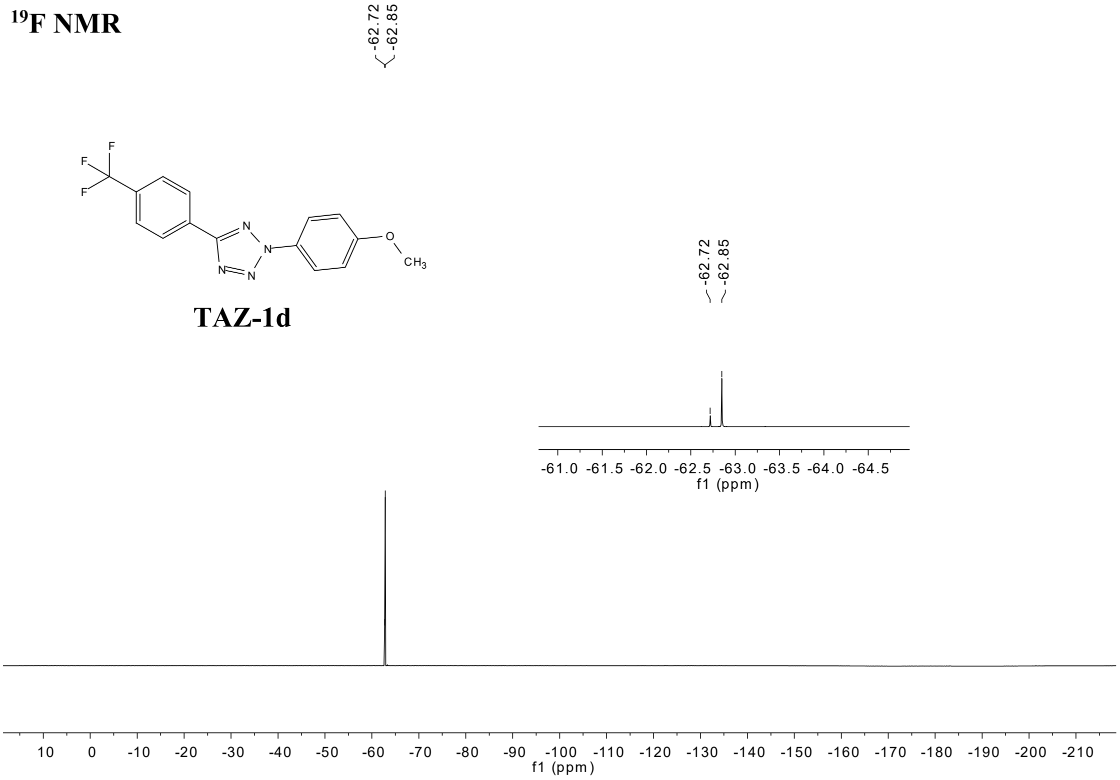

Supplementary Figure 58 ^{13}C NMR and ^{19}F NMR spectra of Compound **TAZ-1d**.

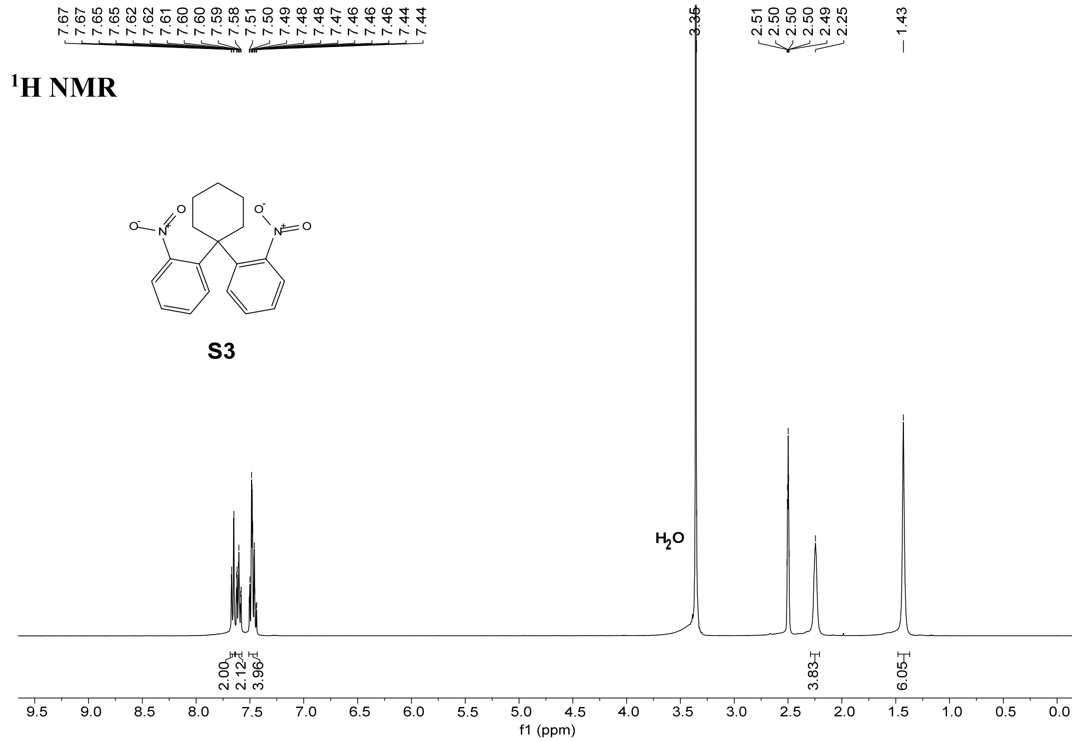

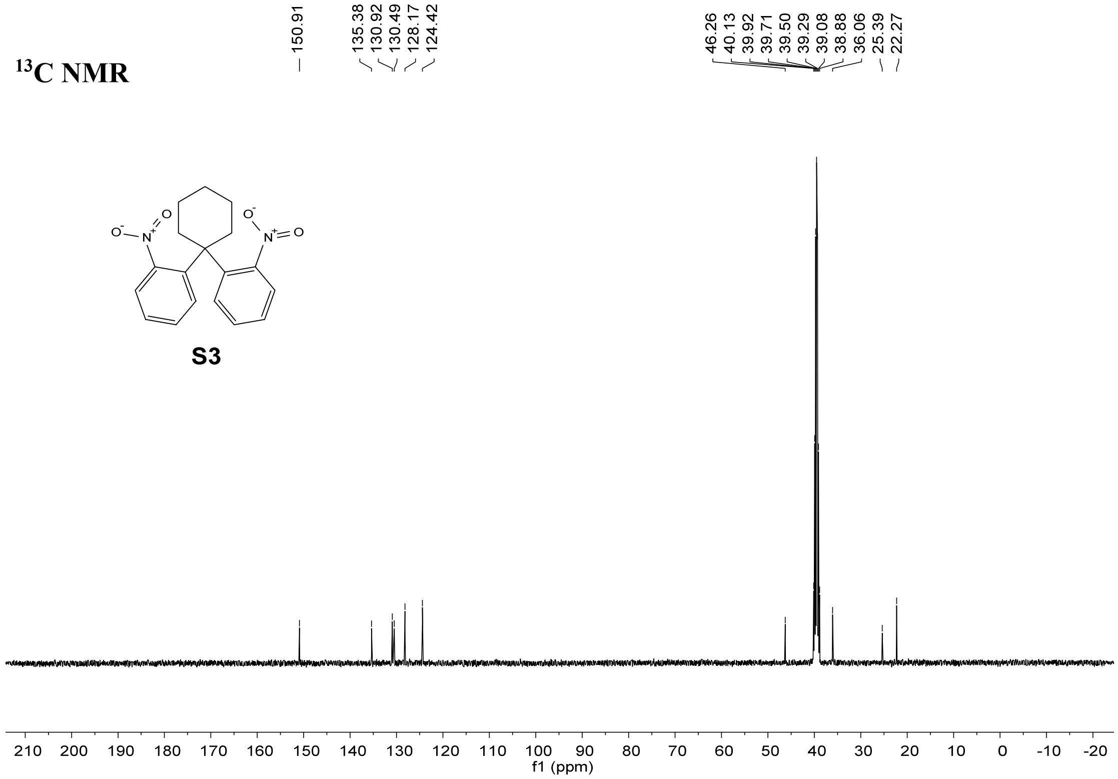

Supplementary Figure 59 ^{1}H NMR and ^{13}C NMR spectra of Compound **S3**.

^{1}H NMR

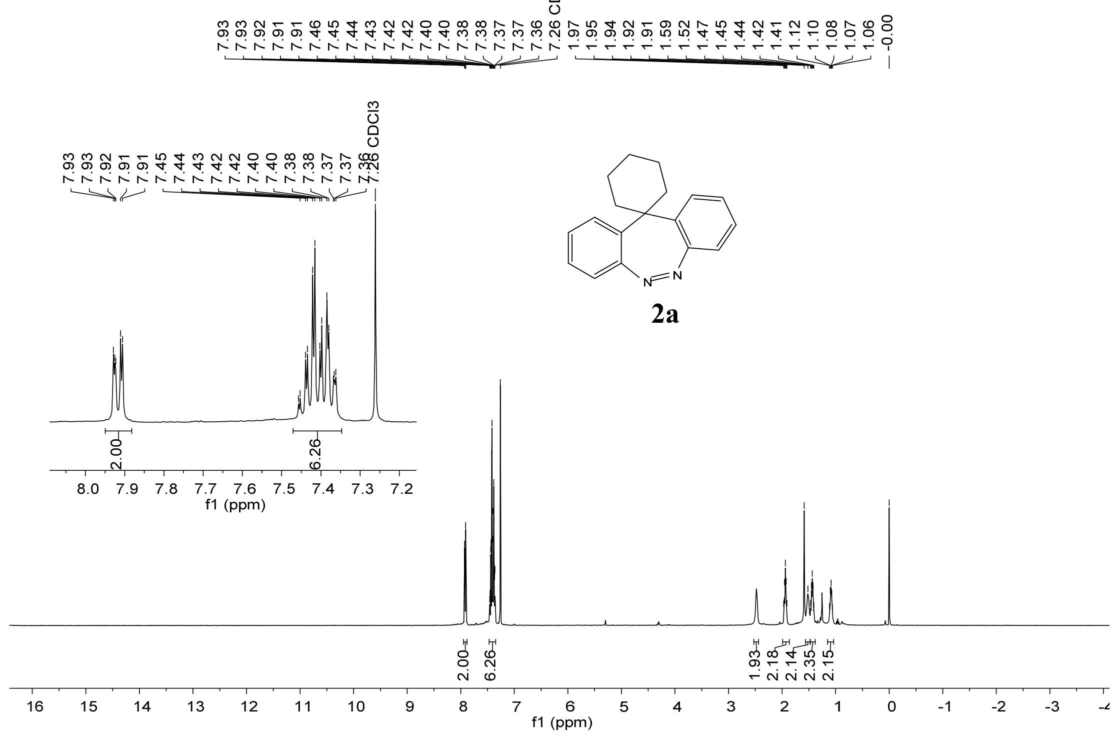

^{13}C NMR

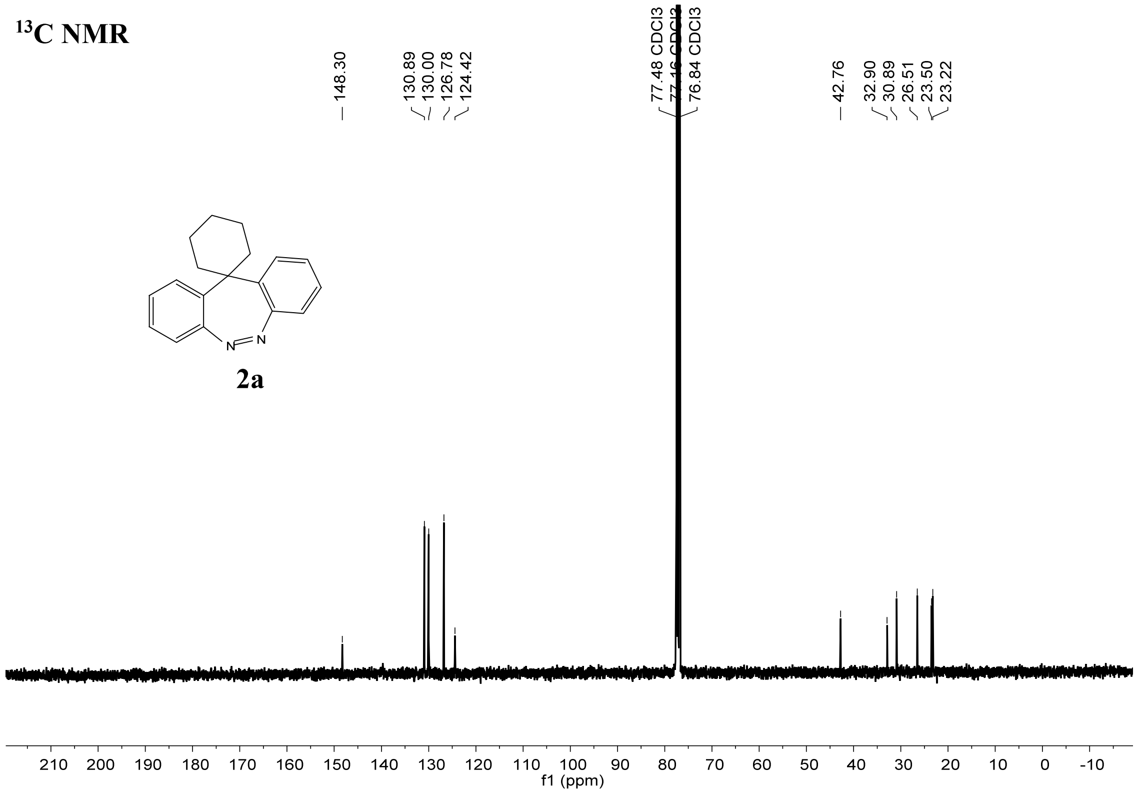

Supplementary Figure 60 ^{1}H NMR and ^{13}C NMR spectra of Compound **2a**.

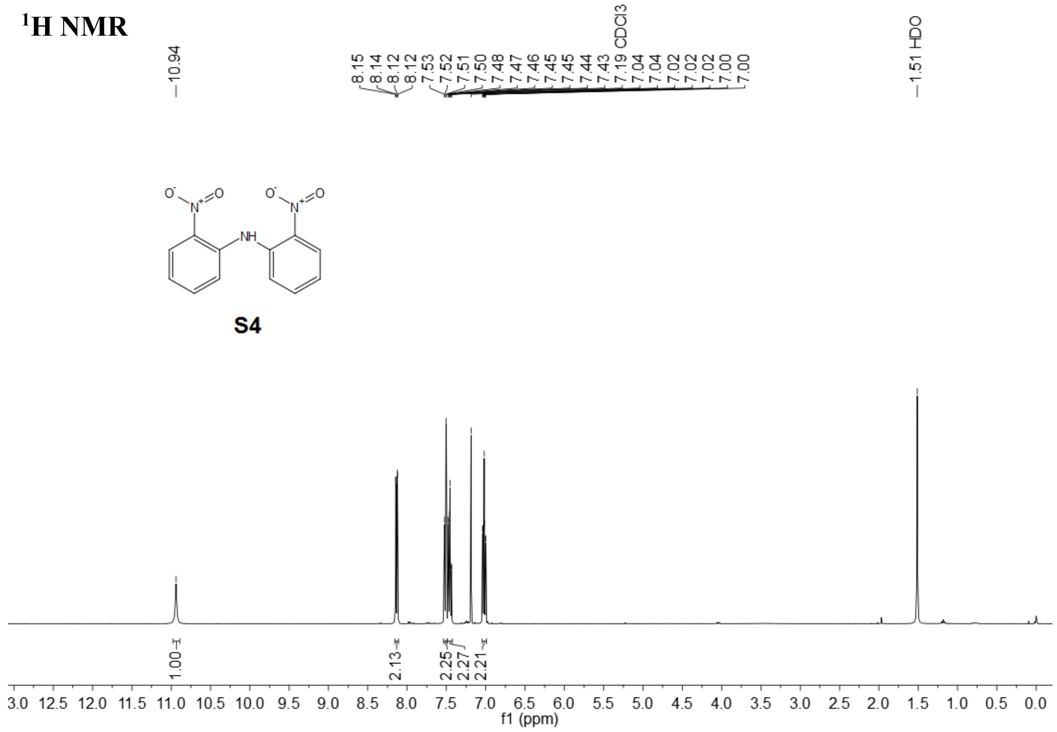

Supplementary Figure 61 ¹H NMR spectra of Compound **S4**.

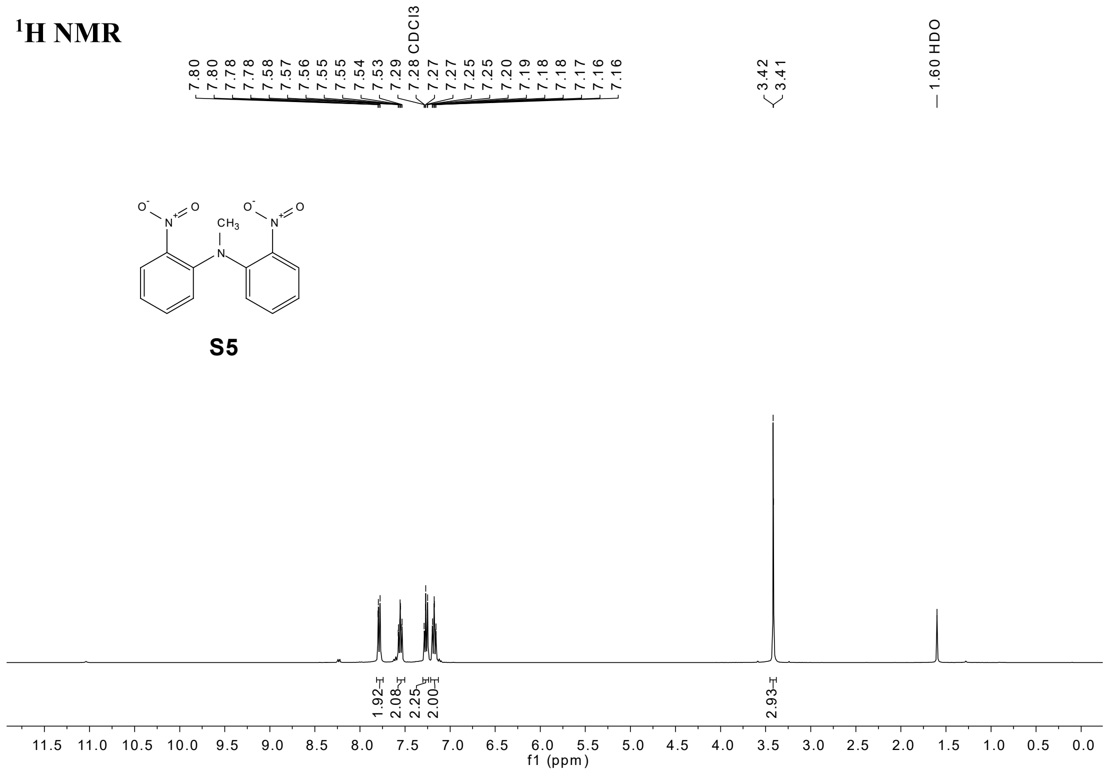

Supplementary Figure 62 ¹H NMR spectra of Compound **S5**.

^{1}H NMR

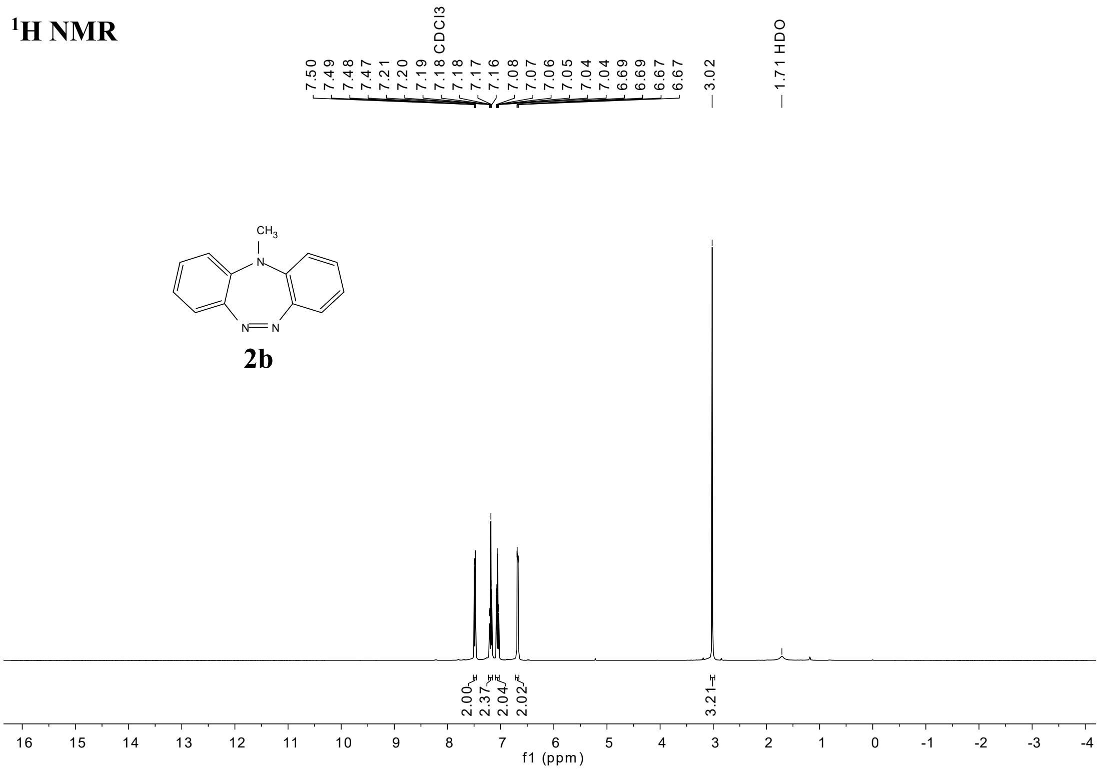

^{13}C NMR

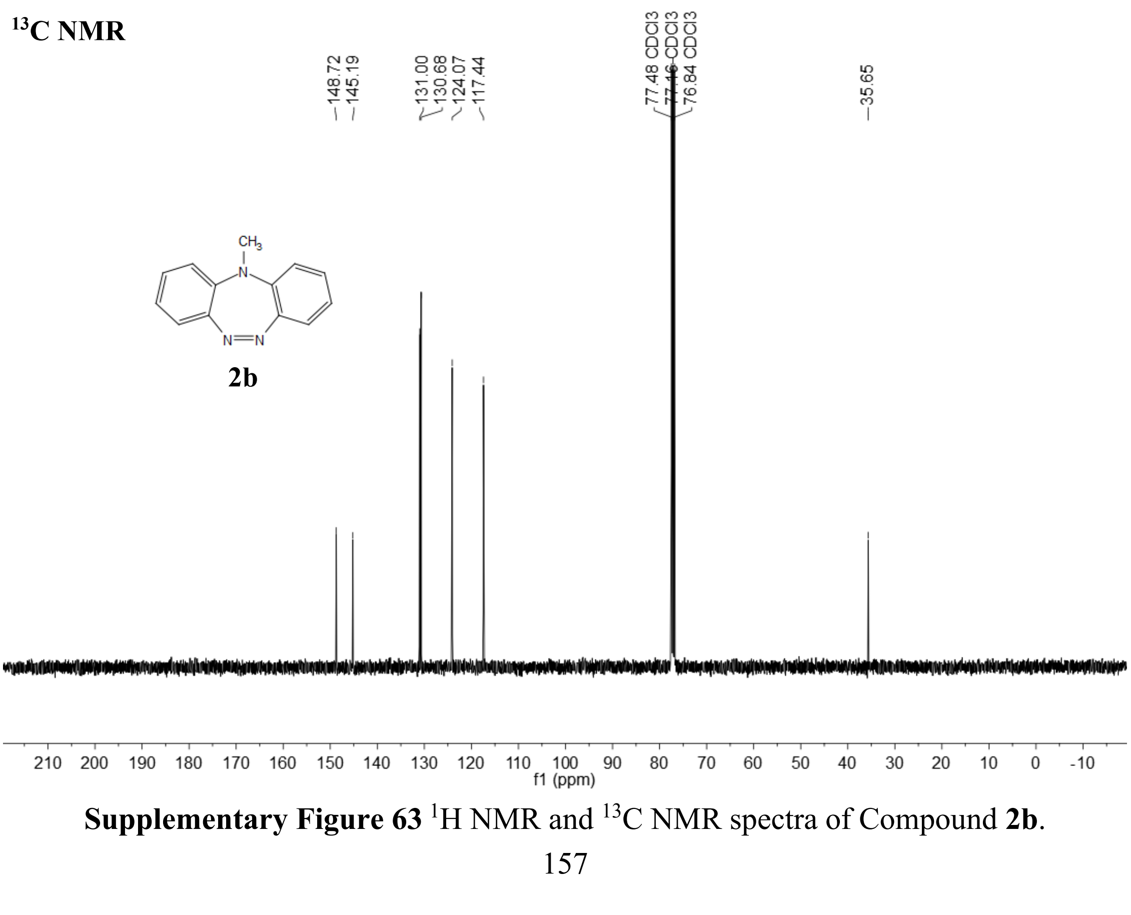

Supplementary Figure 63 ^{1}H NMR and ^{13}C NMR spectra of Compound **2b**.

^{1}H NMR

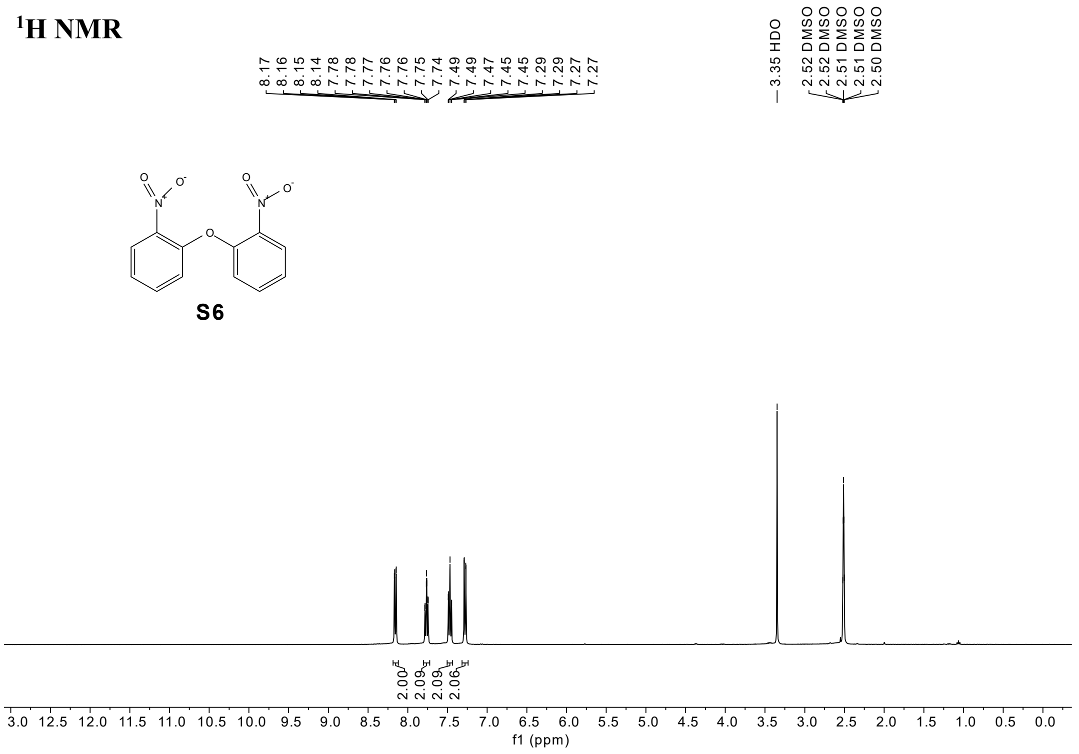

Supplementary Figure 64 ^{1}H NMR spectra of Compound **S6**.

yzp-20190219-gjs-a-122.1.fid

^{1}H NMR

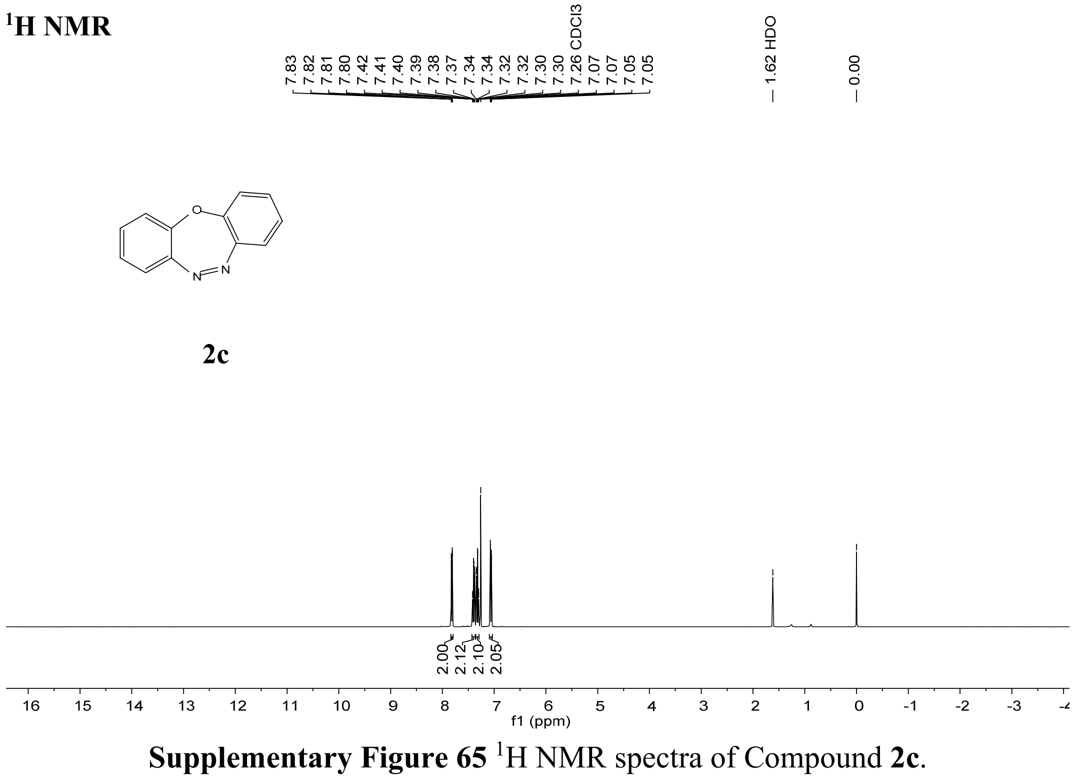

Supplementary Figure 65 ^{1}H NMR spectra of Compound **2c**.

^{13}C NMR

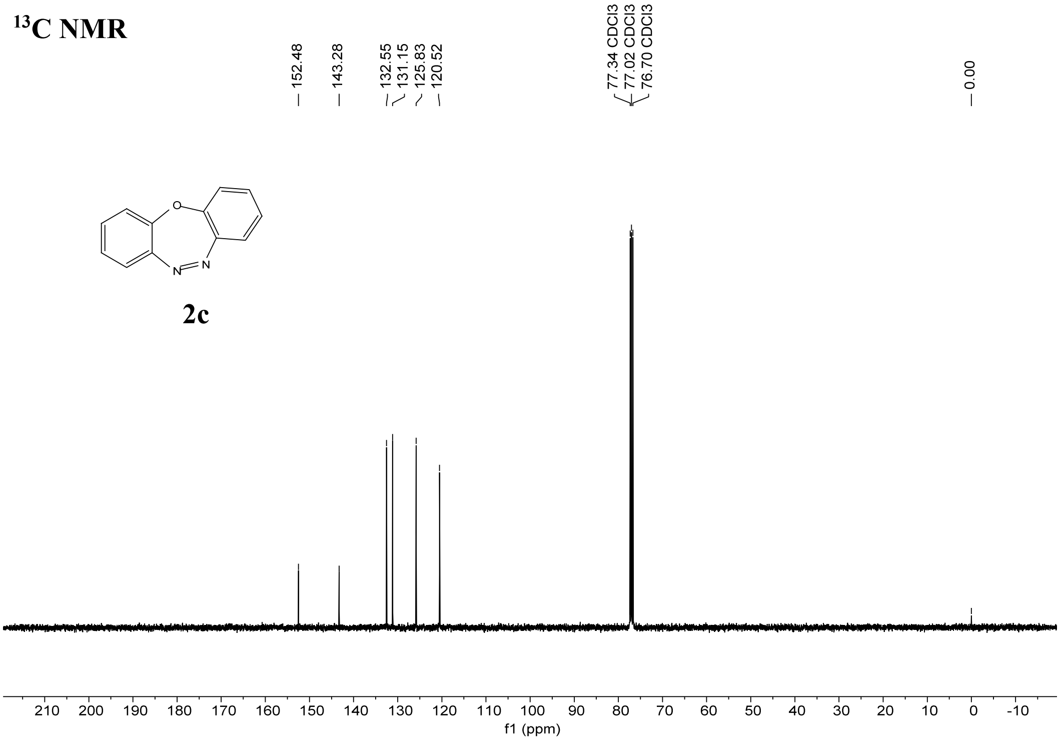

Supplementary Figure 66 ^{13}C NMR spectra of Compound **2c**.

^{1}H NMR

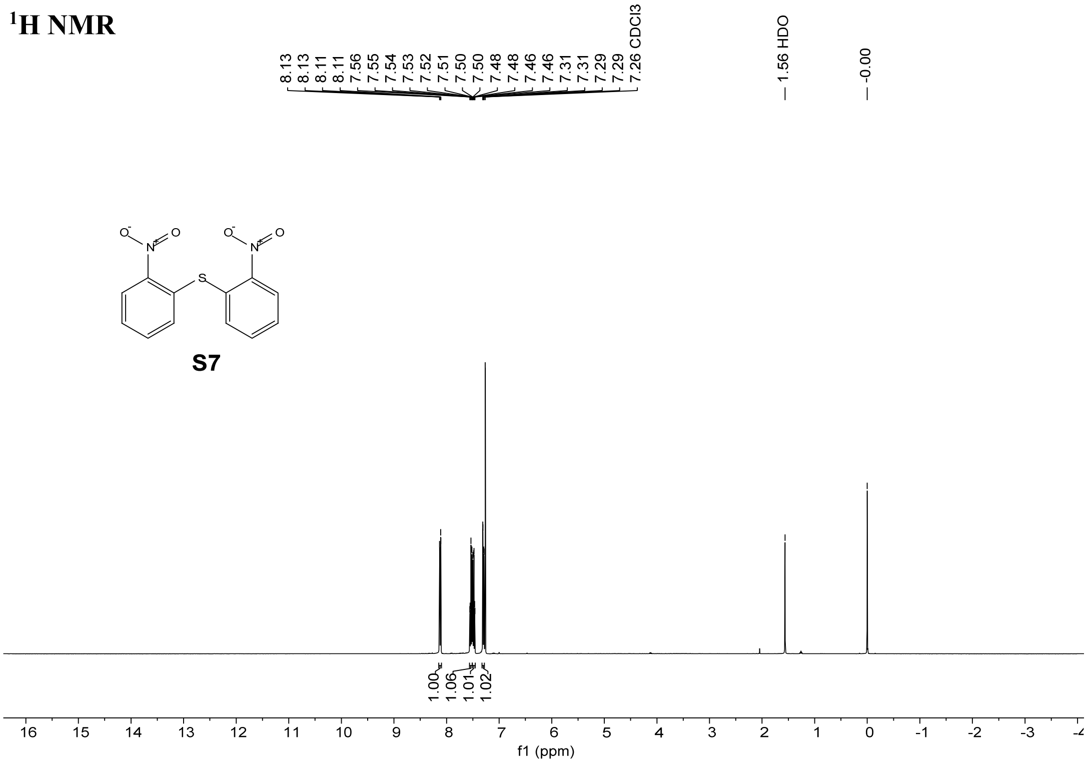

Supplementary Figure 67 ^{1}H NMR spectra of Compound **S7**.

^{1}H NMR

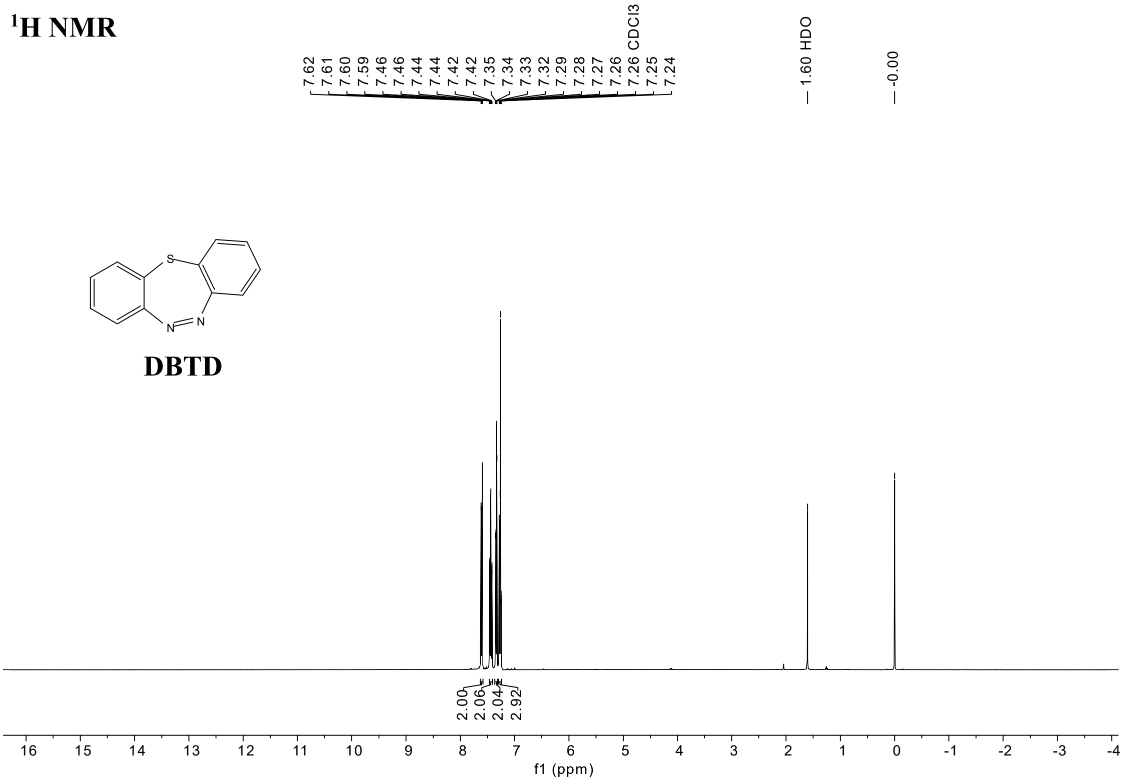

yzp-20190219-gjs-g.2.fid

^{13}C NMR

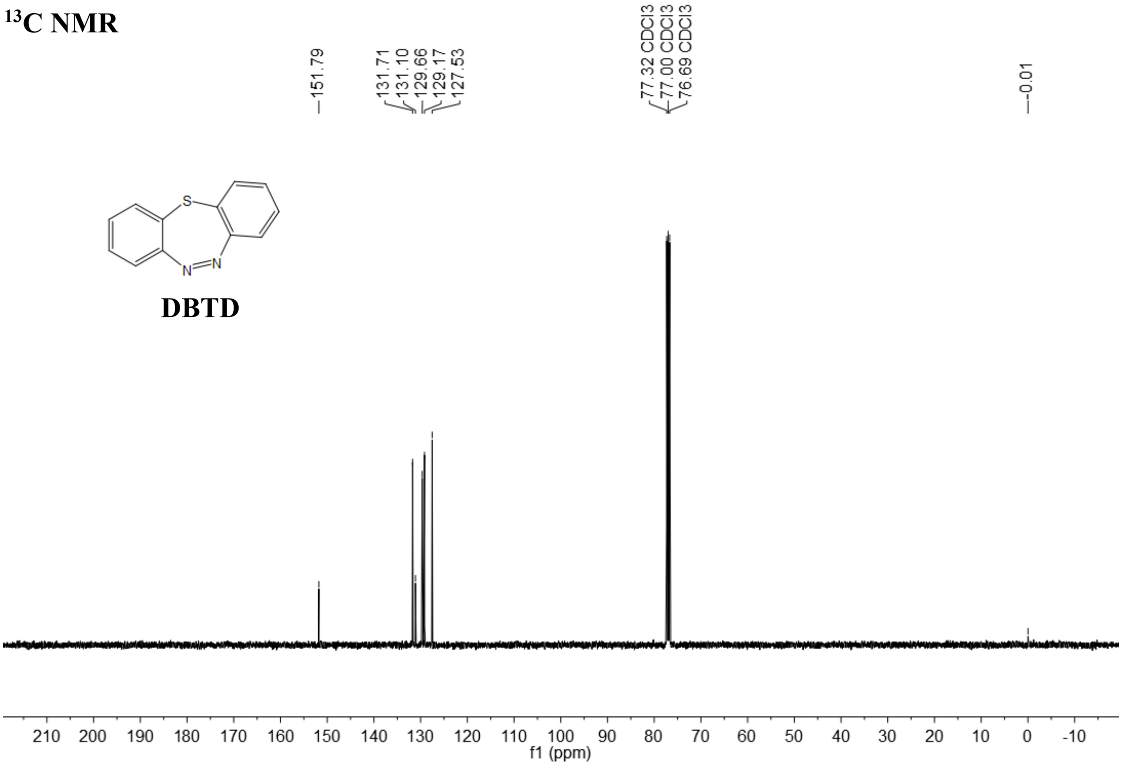

Supplementary Figure 68 ^{1}H NMR and ^{13}C NMR spectra of Compound **DBTD**.

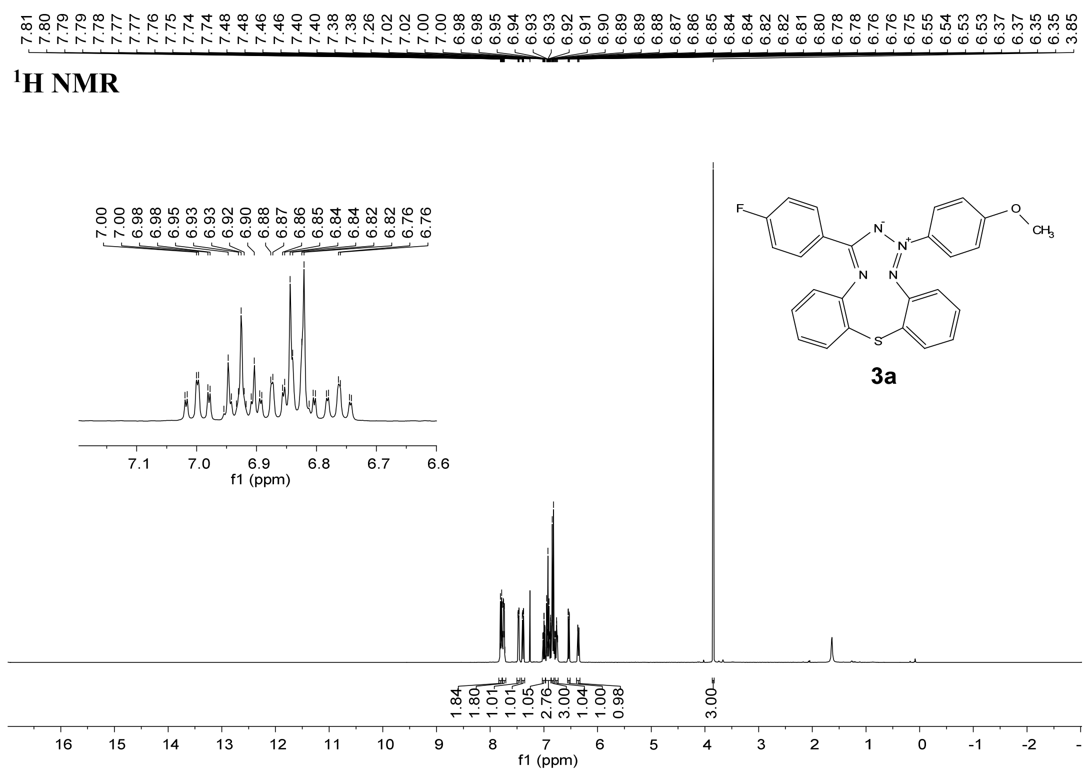

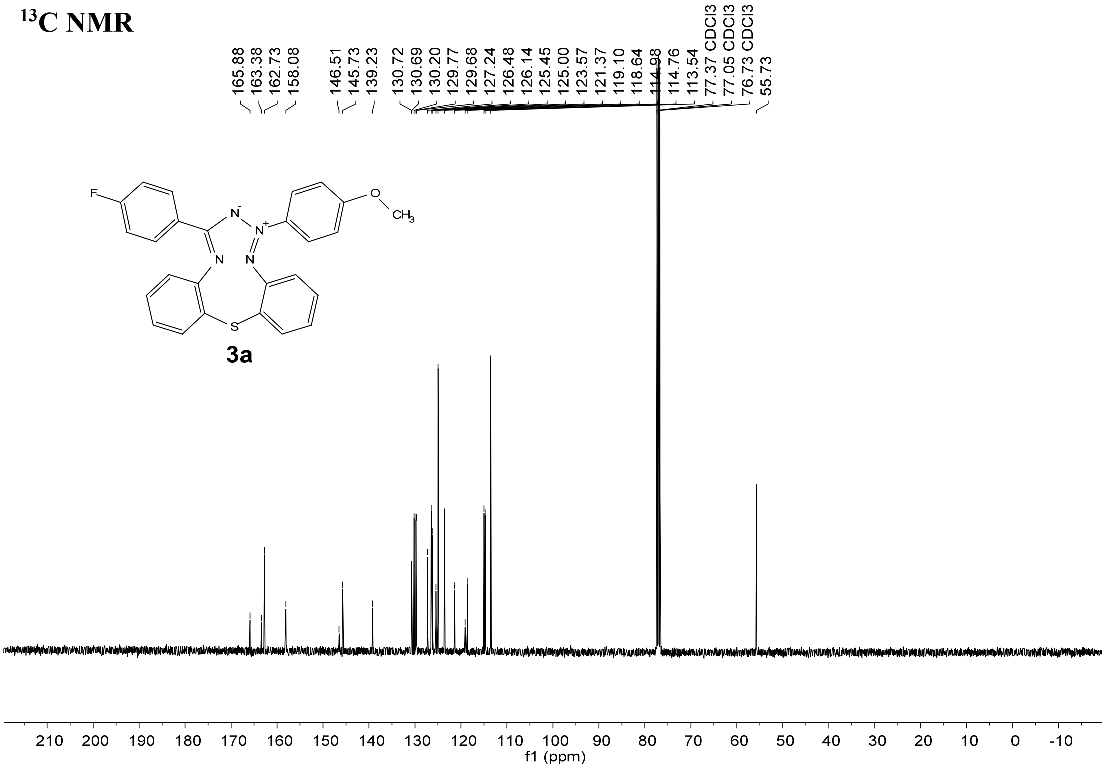

Supplementary Figure 69 ^{1}H NMR and ^{13}C NMR spectra of Compound **3a**.

^{19}F NMR

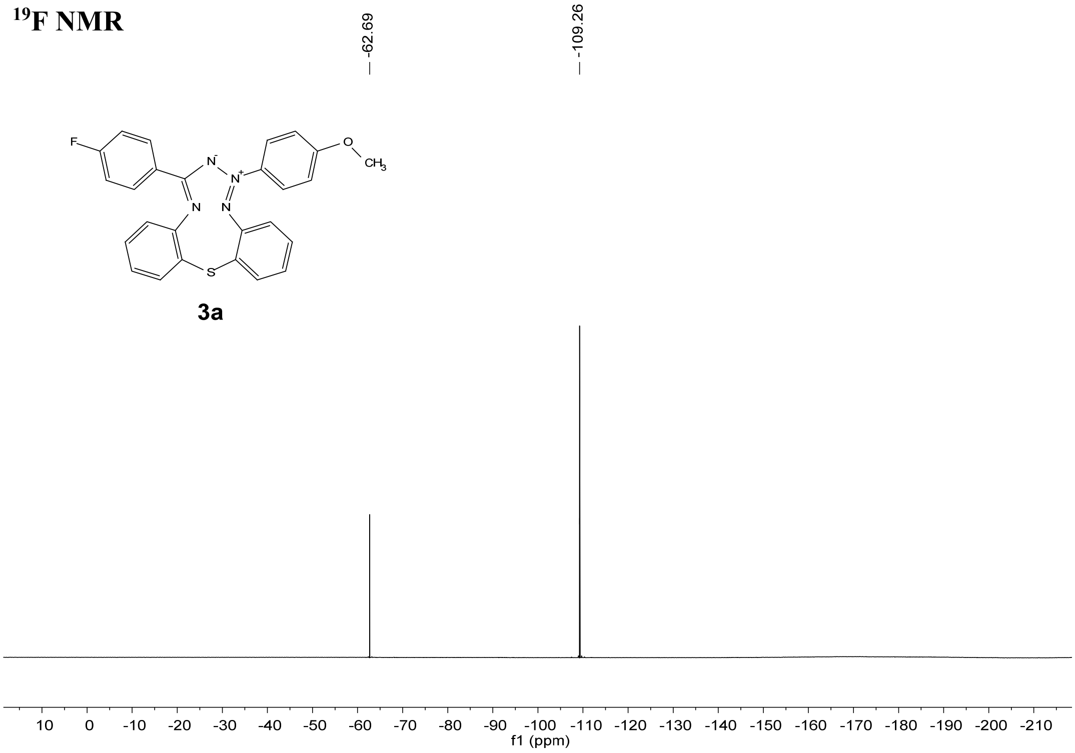

Supplementary Figure 70 ^{19}F NMR spectra of Compound **3a**.

^{1}H NMR

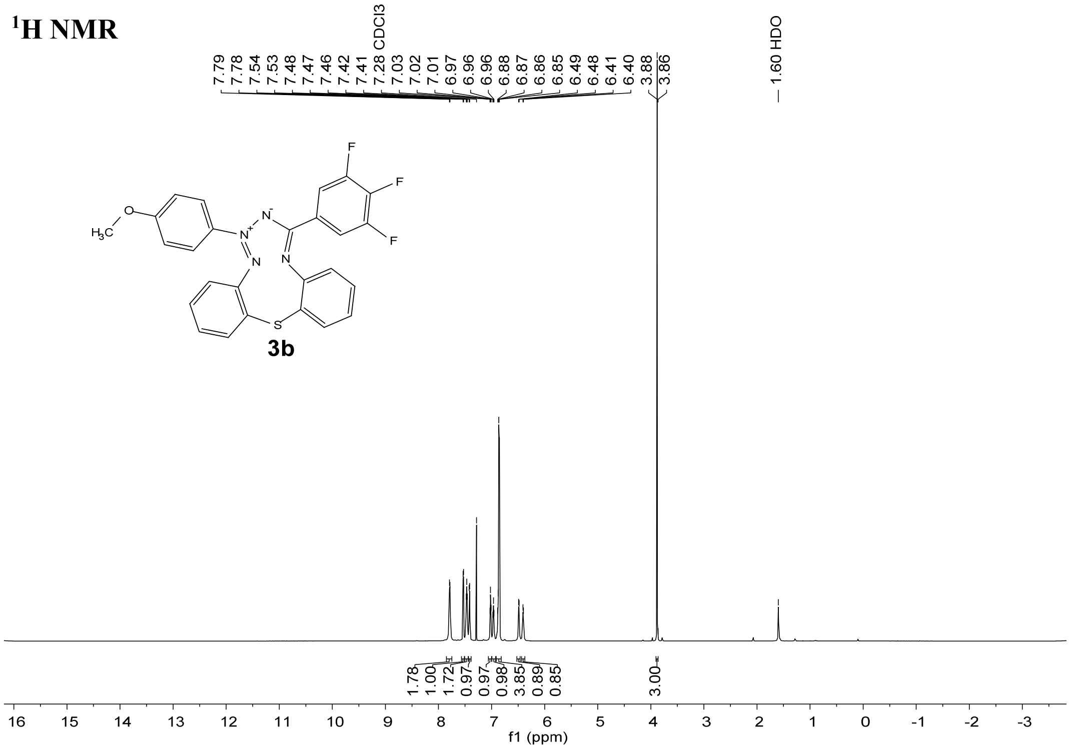

Supplementary Figure 71 ^{1}H NMR spectra of Compound **3b**.

^{13}C NMR

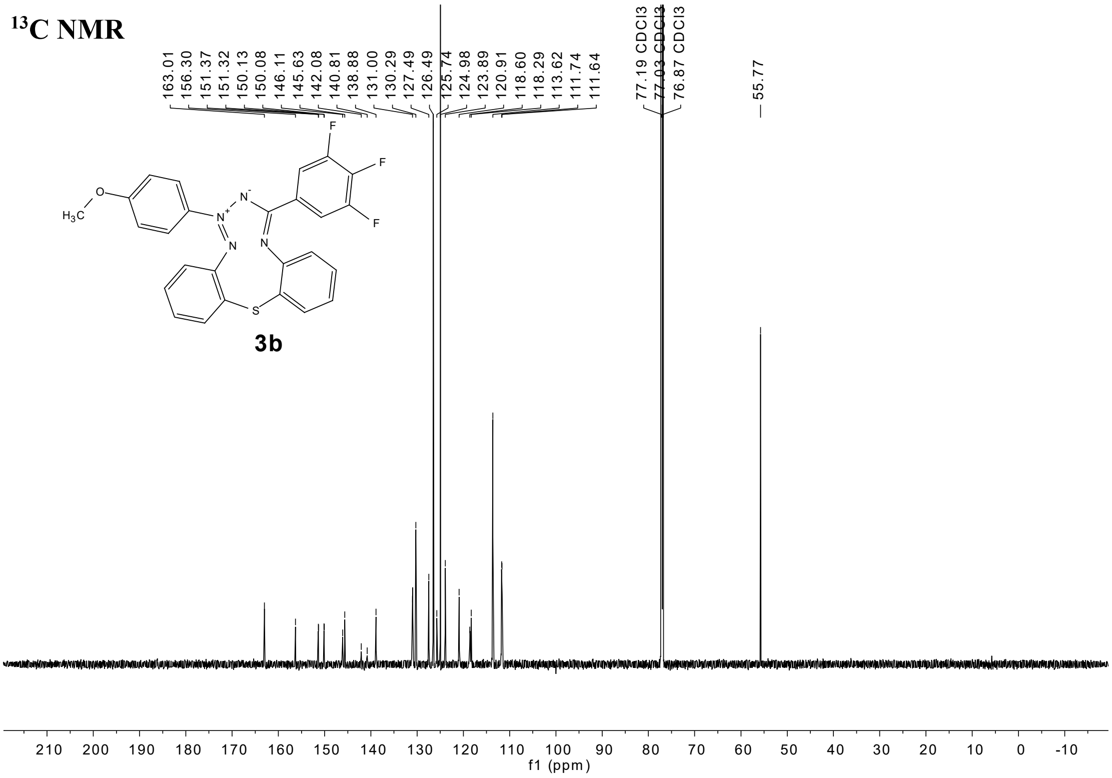

^{19}F NMR

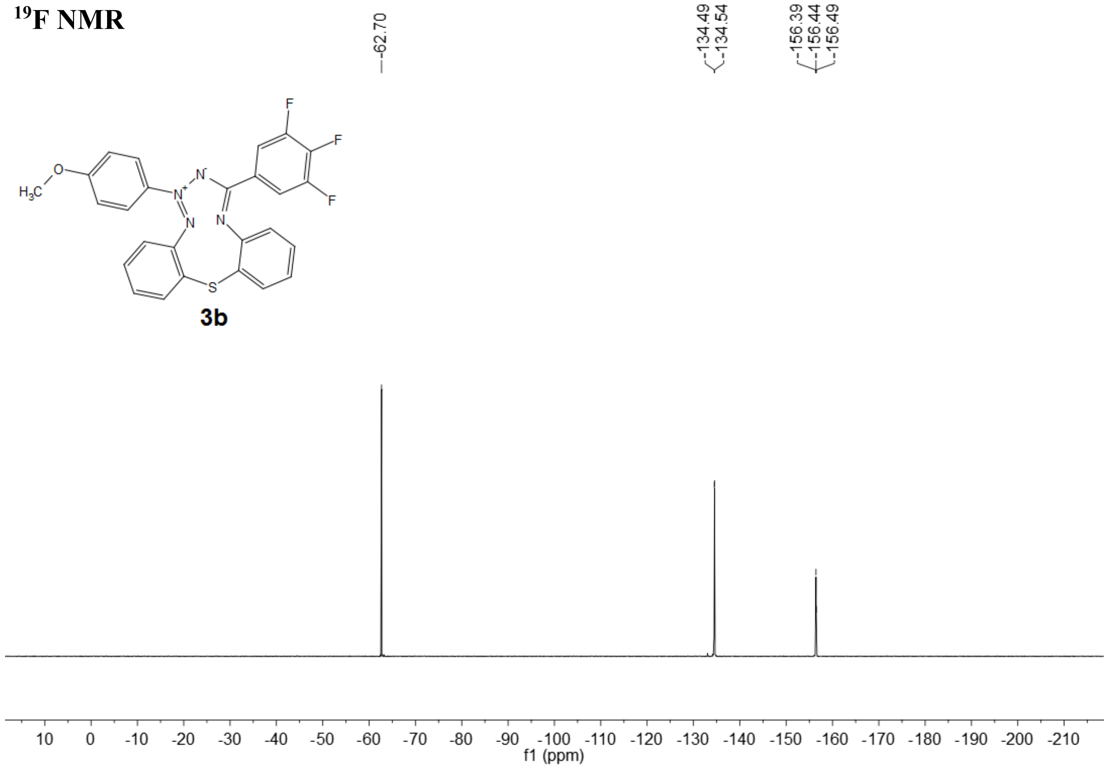

Supplementary Figure 72 ^{13}C NMR and ^{19}F NMR spectra of Compound **3b**.

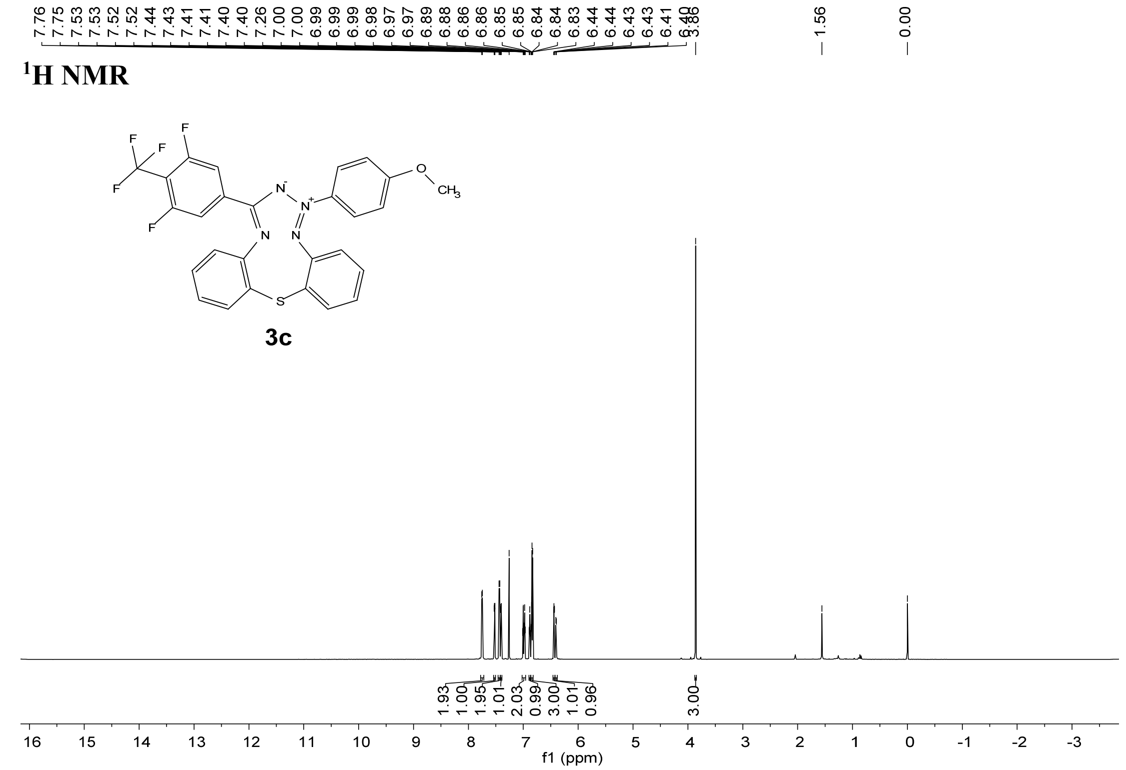

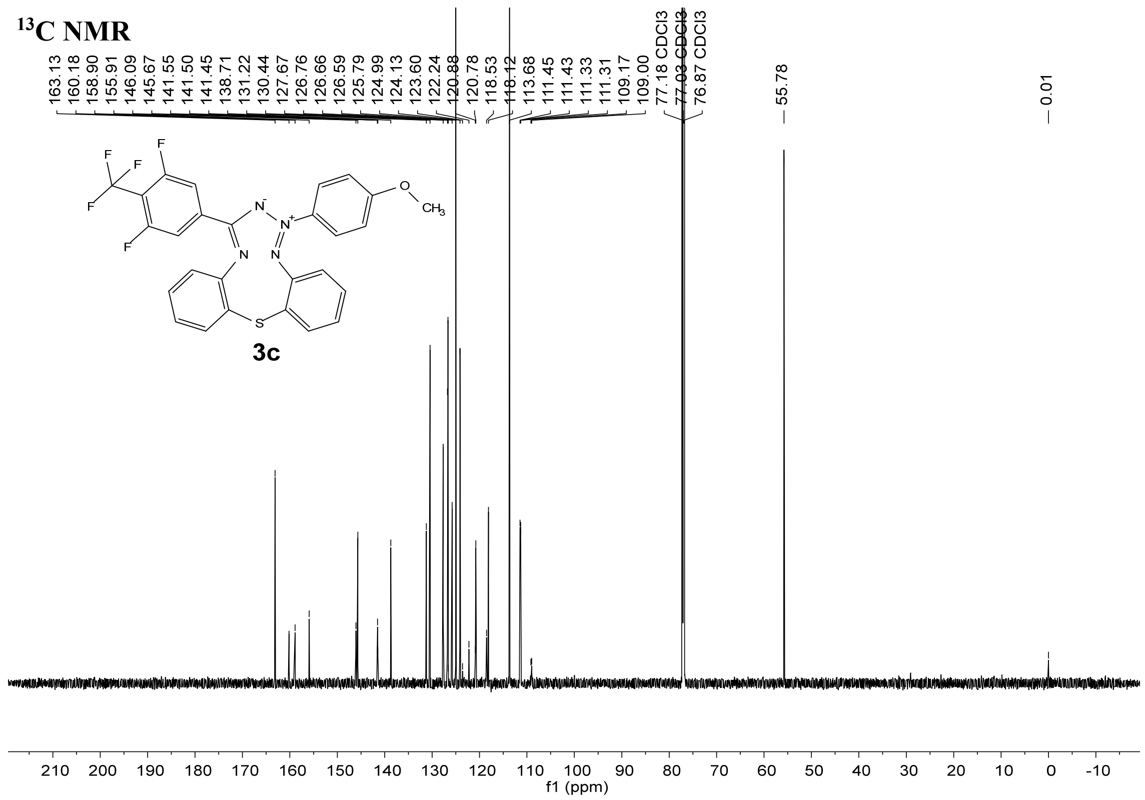

Supplementary Figure 73 ^{1}H NMR and ^{13}C NMR spectra of Compound **3c**.

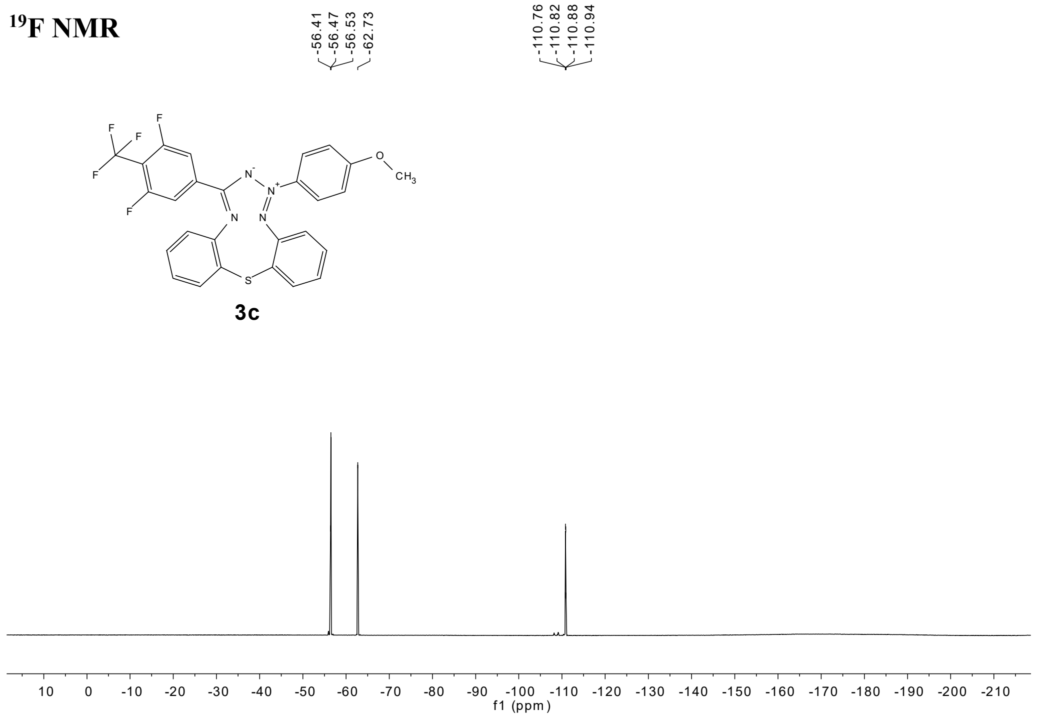

Supplementary Figure 74 ^{19}F NMR spectra of Compound **3c**.

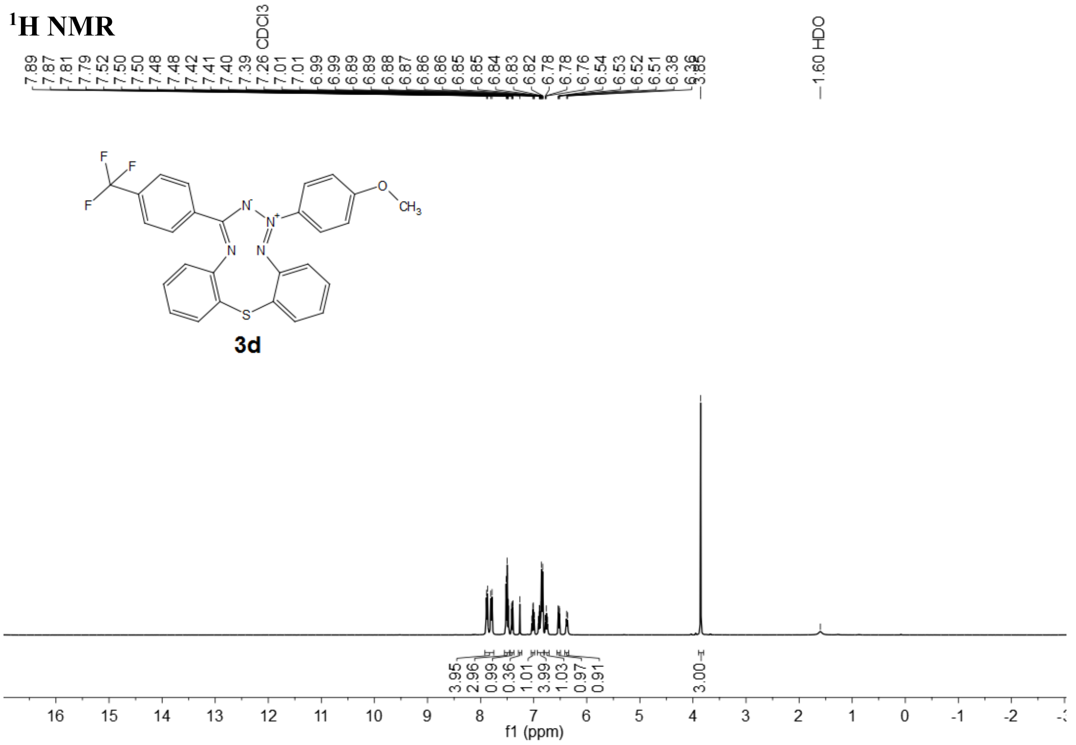

Supplementary Figure 75 ^{1}H NMR spectra of Compound **3d**.

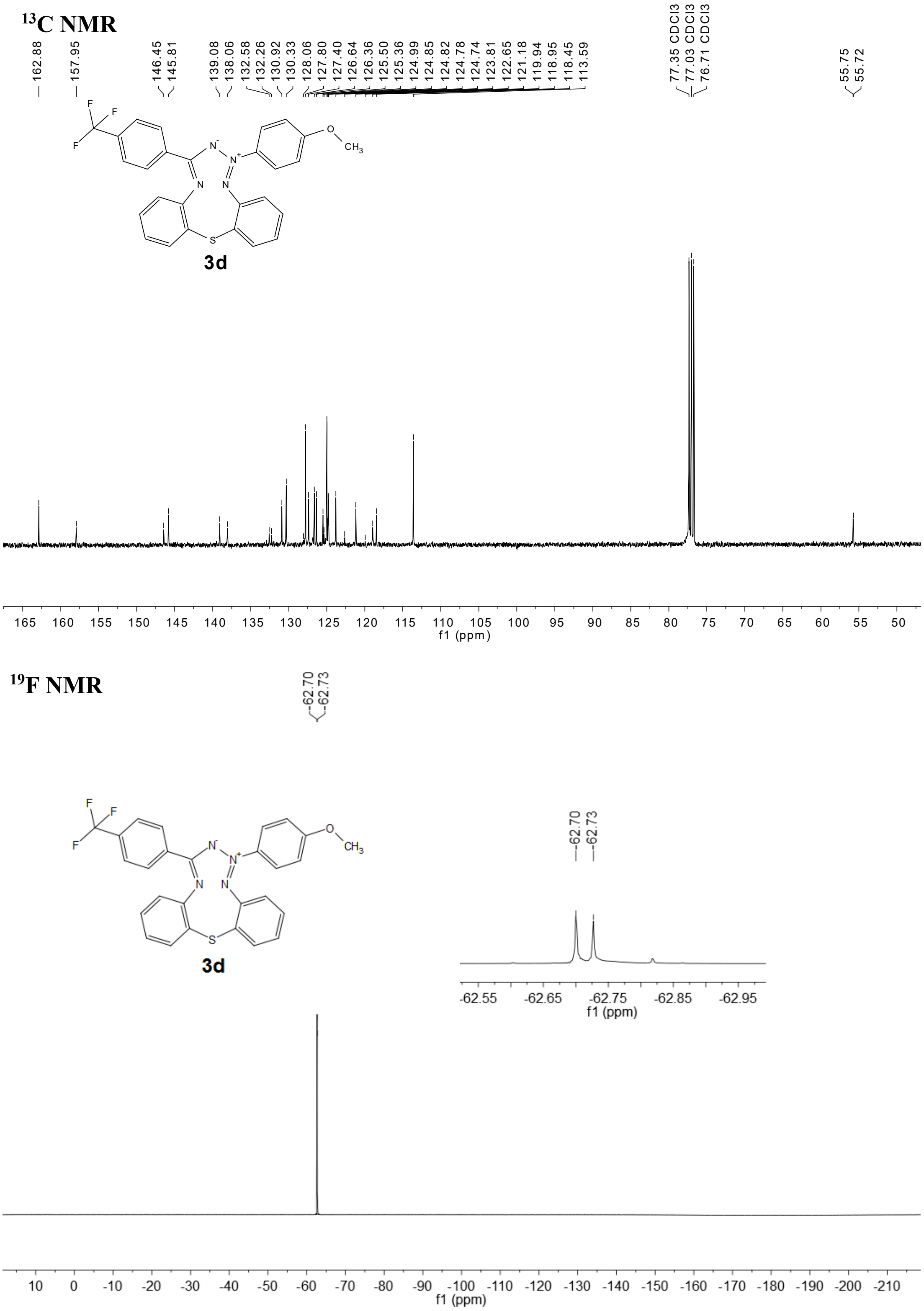

Supplementary Figure 76 ^{13}C NMR and ^{19}F NMR spectra of Compound **3d**.

^{1}H NMR

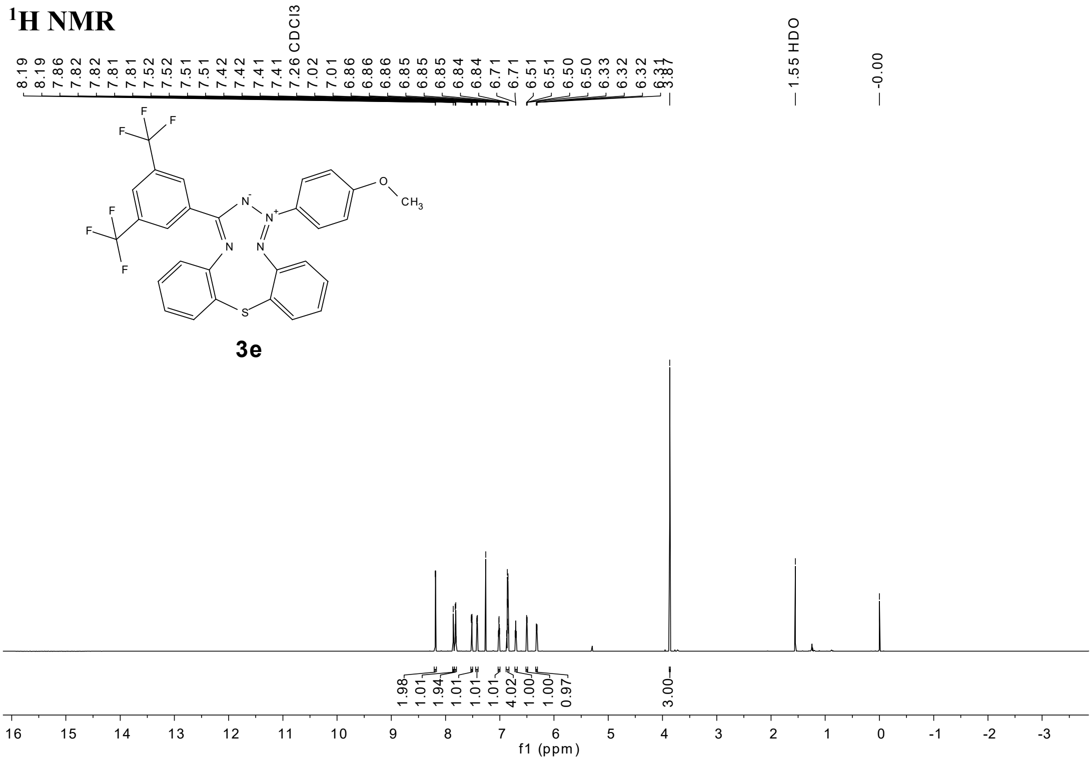

^{13}C NMR

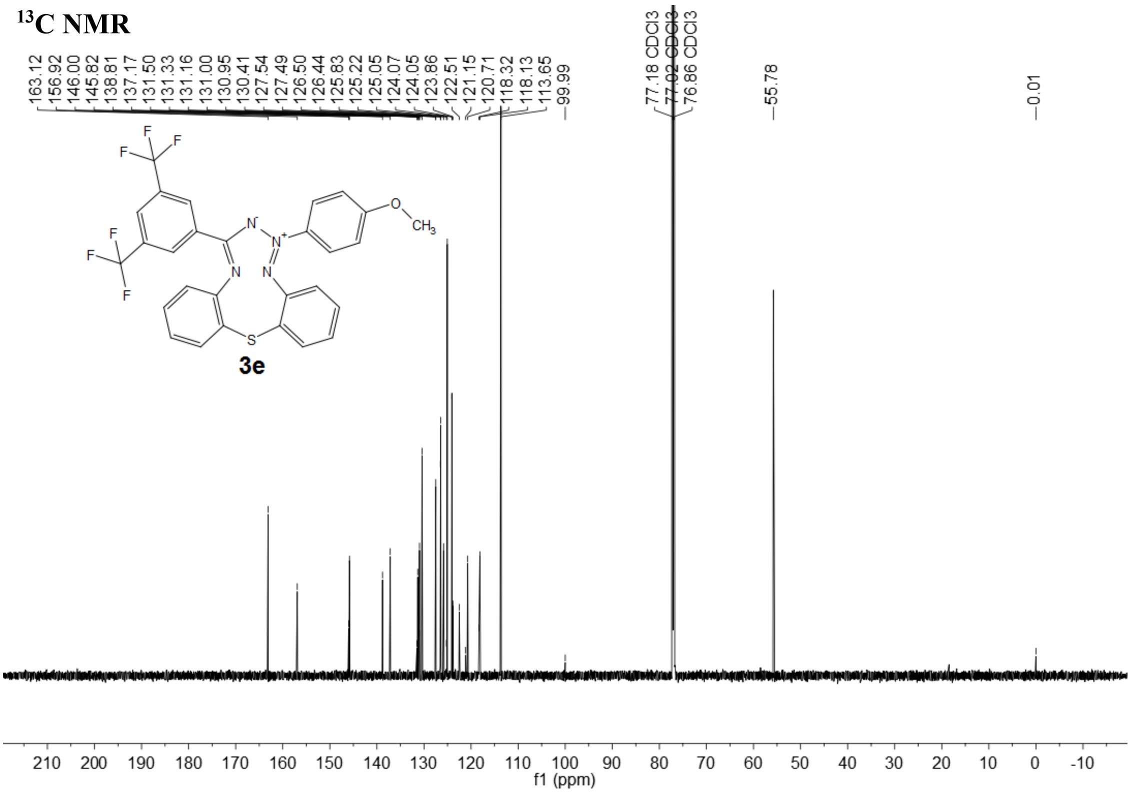

Supplementary Figure 77 ^{1}H NMR and ^{13}C NMR spectra of Compound **3e**.

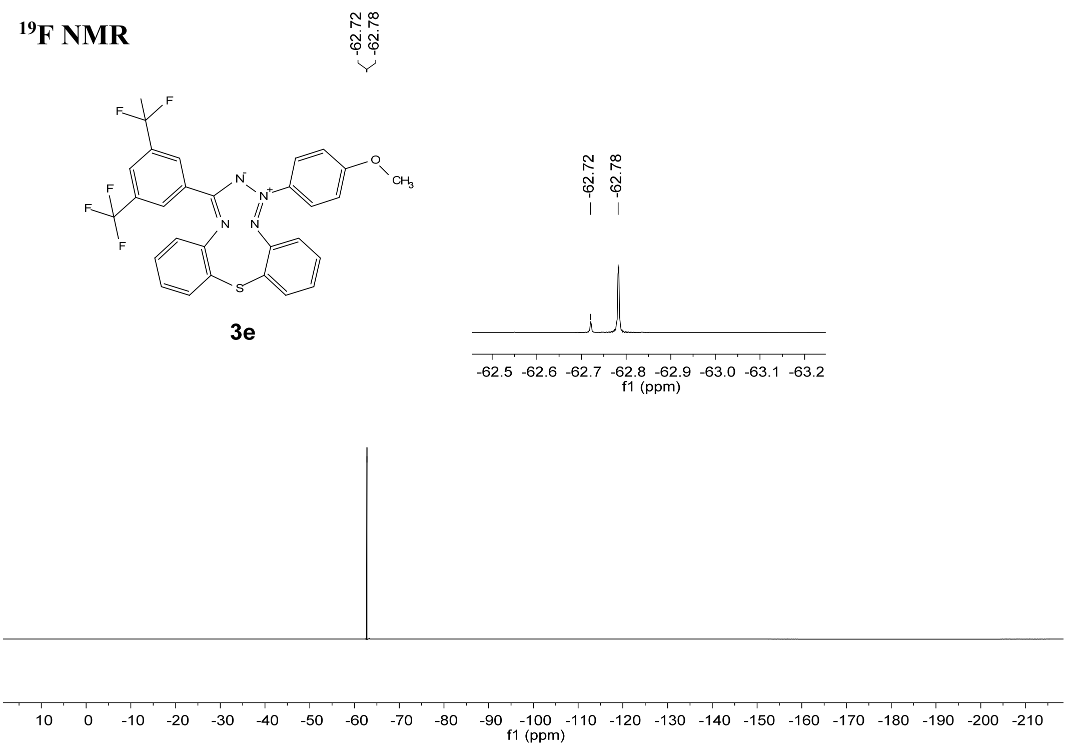

Supplementary Figure 78 ^{19}F NMR spectra of Compound **3e**.

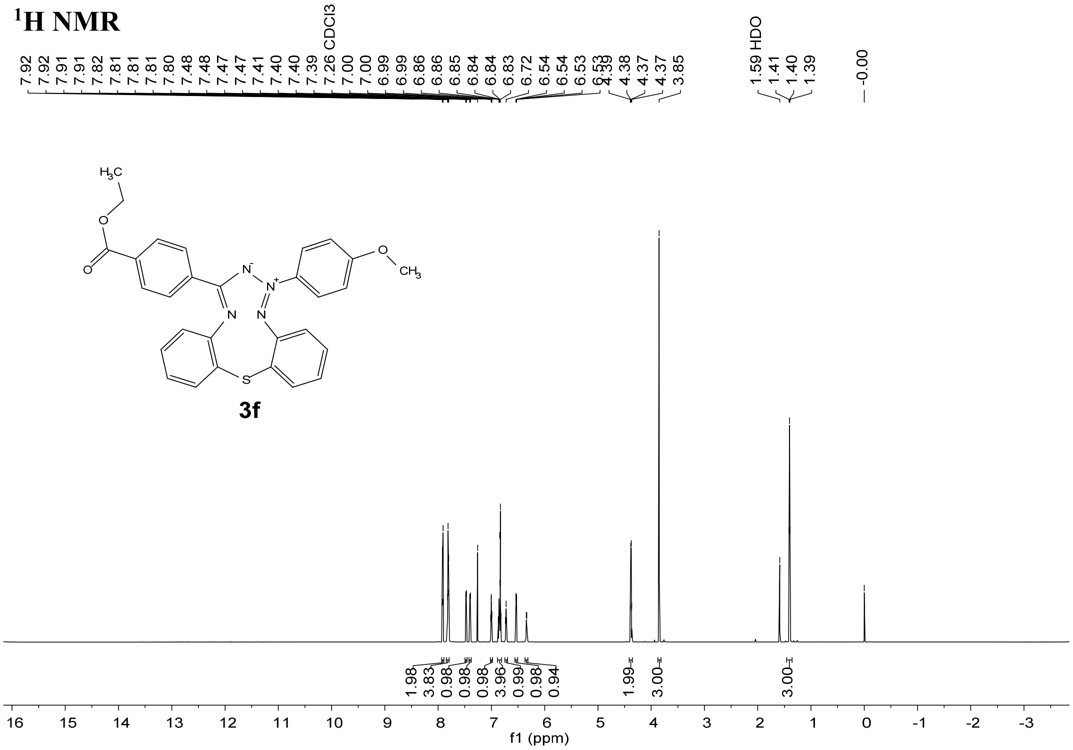

Supplementary Figure 79 ^{1}H NMR spectra of Compound **3f**.

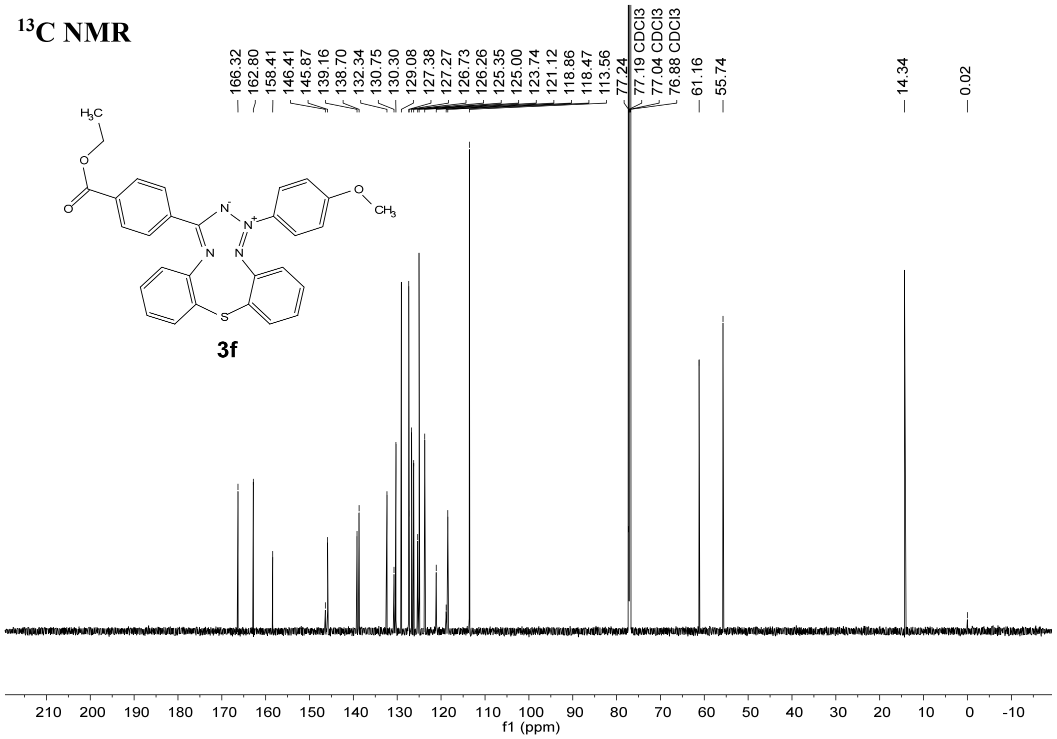

Supplementary Figure 80 ^{13}C NMR spectra of Compound **3f**.

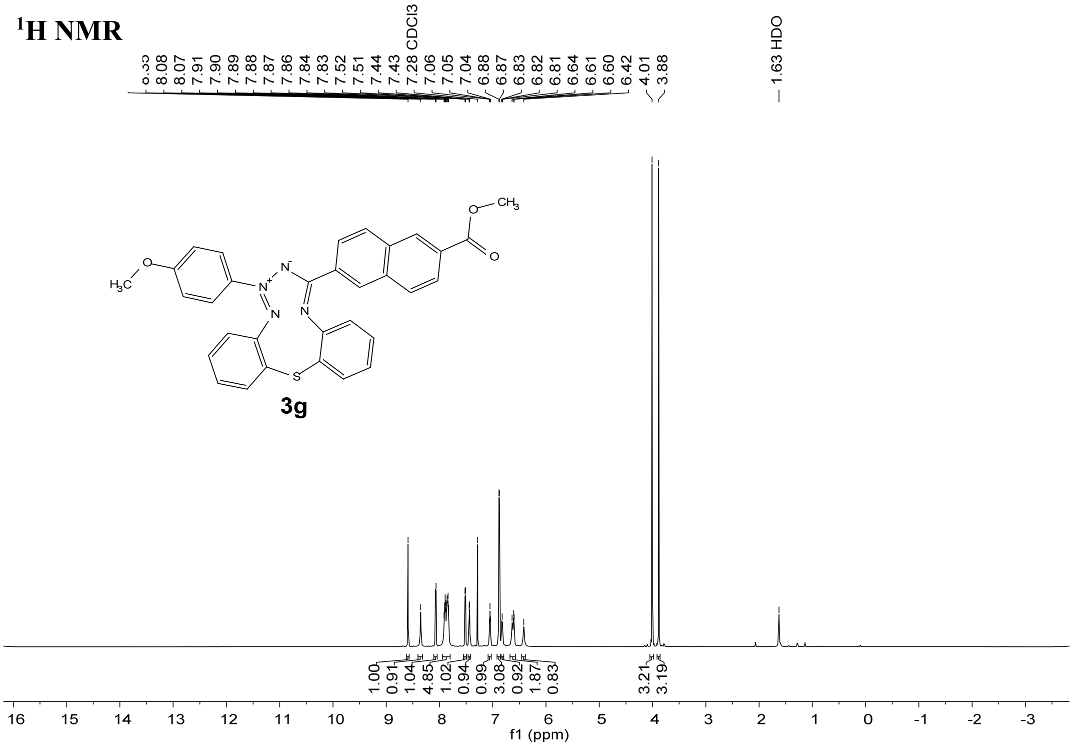

Supplementary Figure 81 ^{1}H NMR spectra of Compound **3g**.

^{13}C NMR

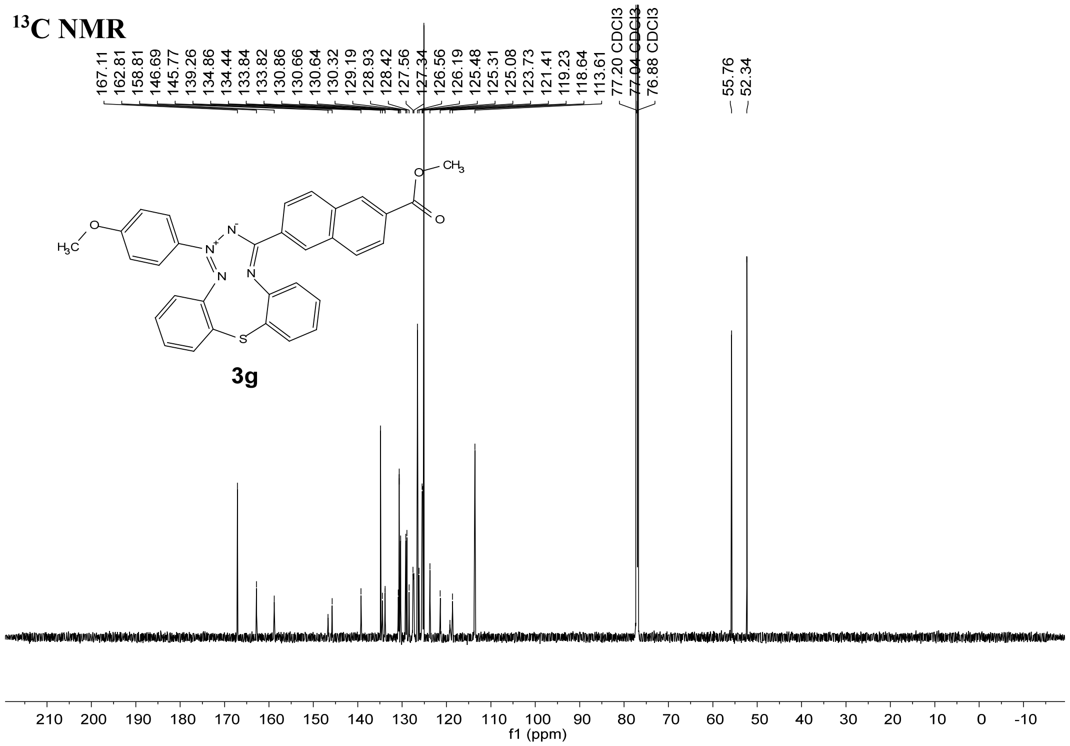

Supplementary Figure 82 ^{13}C NMR spectra of Compound **3g**.

^{1}H NMR

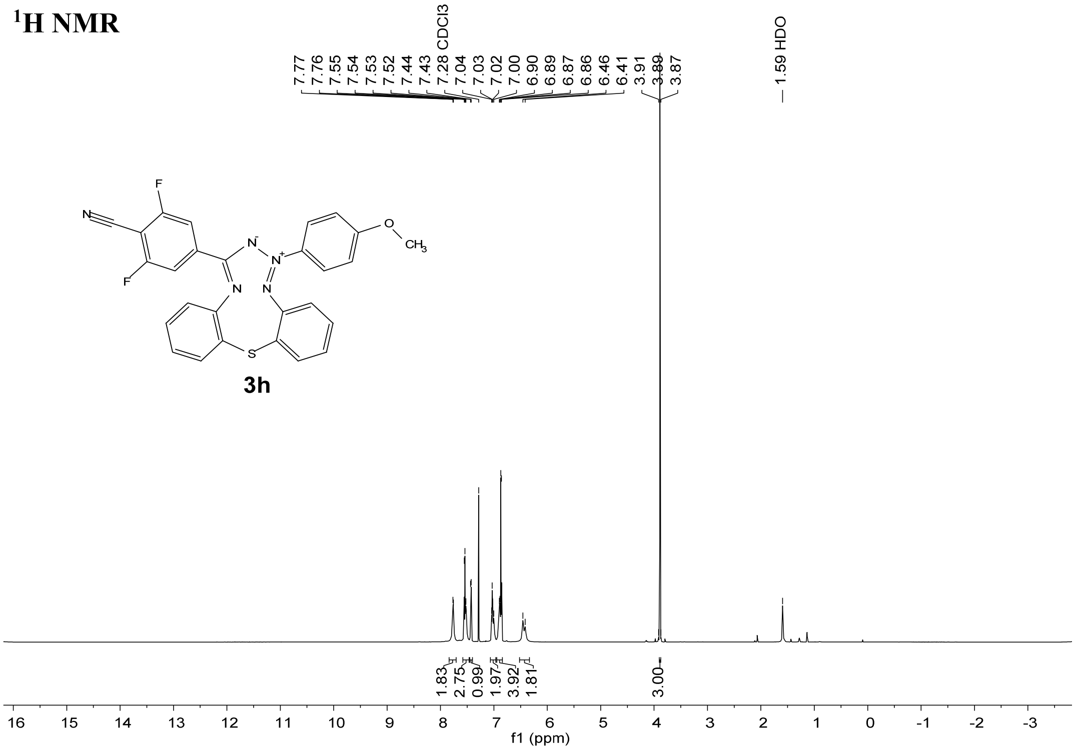

Supplementary Figure 83 ^{1}H NMR spectra of Compound **3h**.

^{13}C NMR

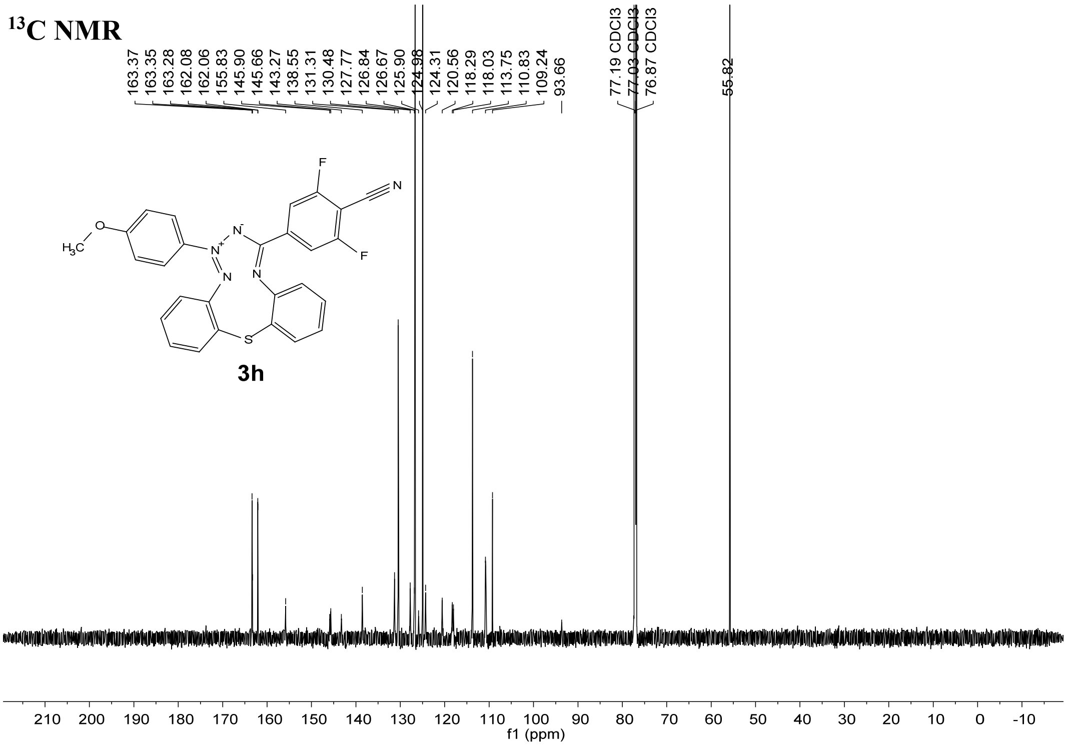

^{19}F NMR

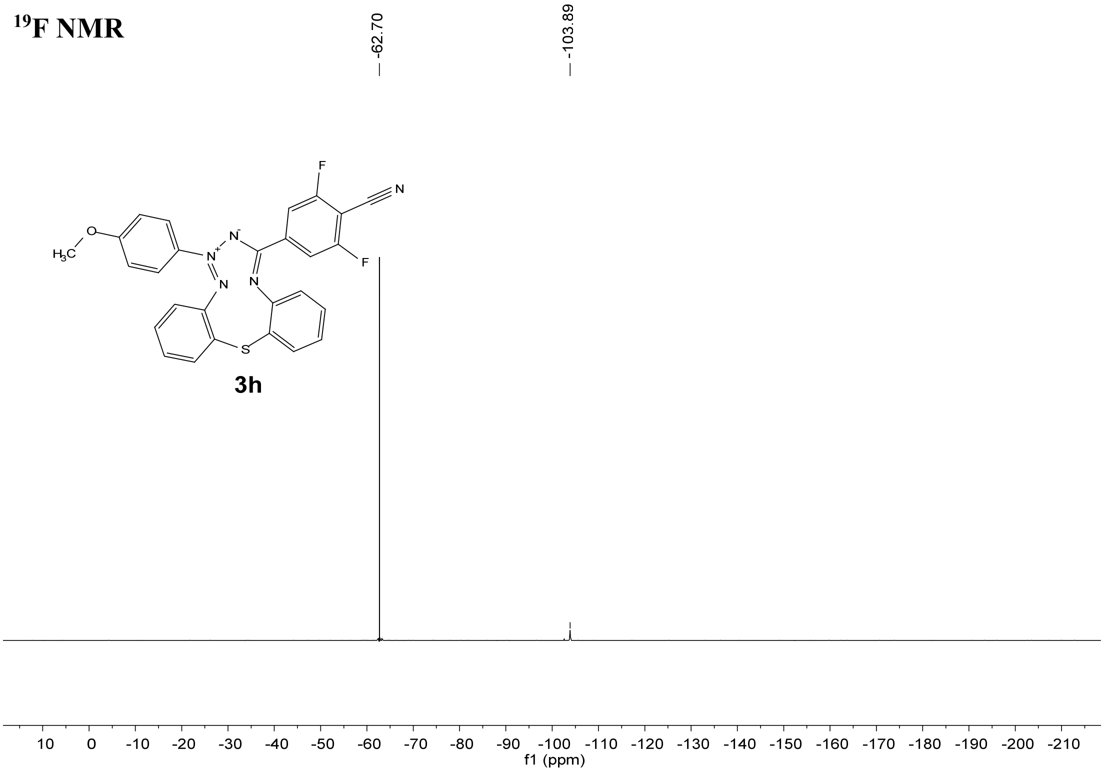

Supplementary Figure 84 ^{13}C NMR ^{19}F NMR spectra of Compound **3h**.

^{1}H NMR

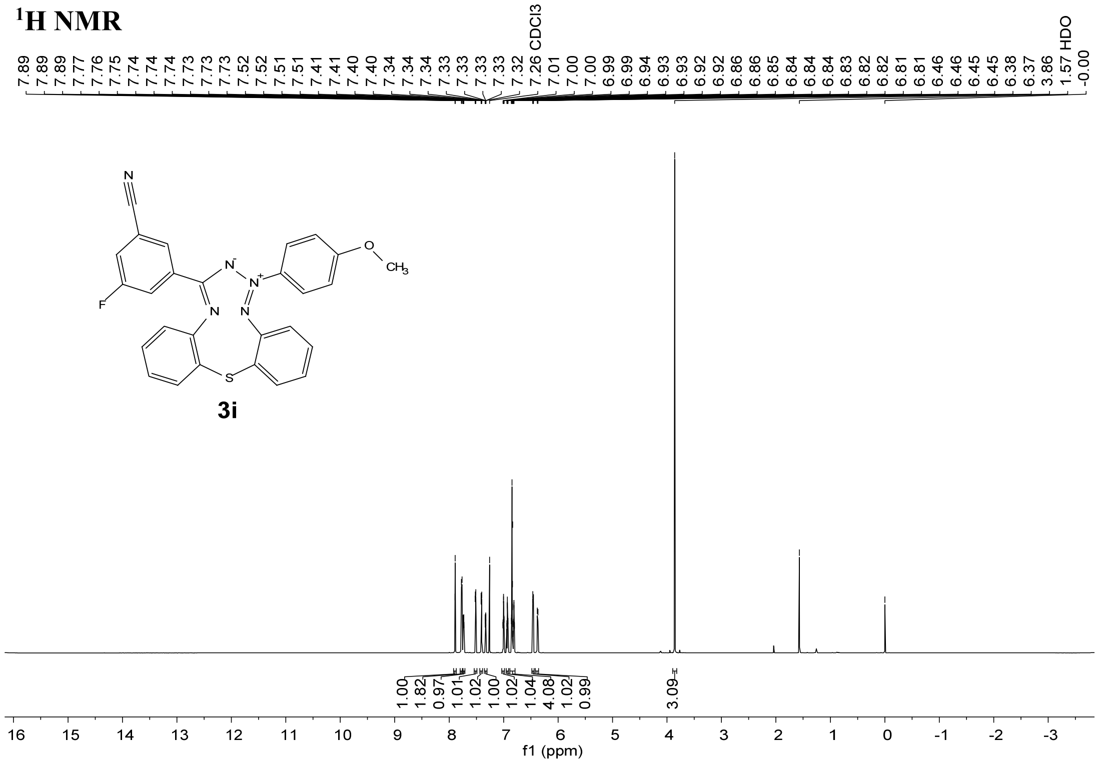

^{13}C NMR

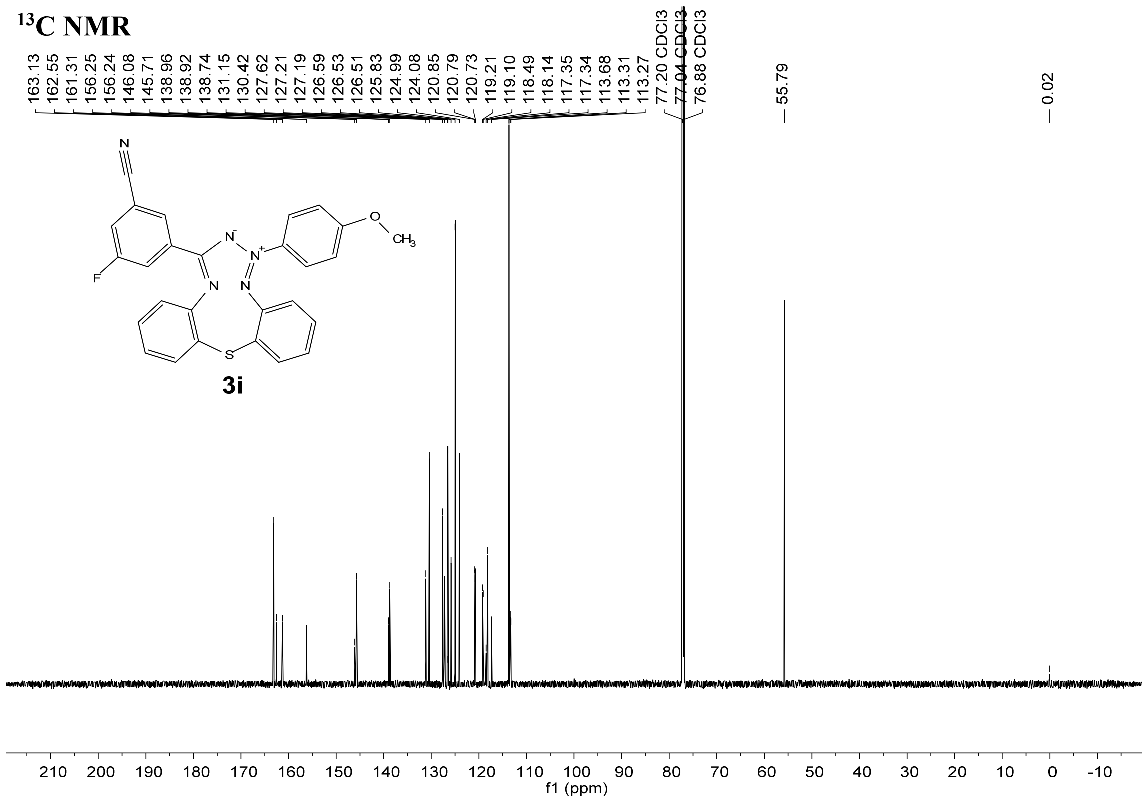

Supplementary Figure 85 ^{1}H NMR and ^{13}C NMR spectra of Compound **3i**.

^{19}F NMR

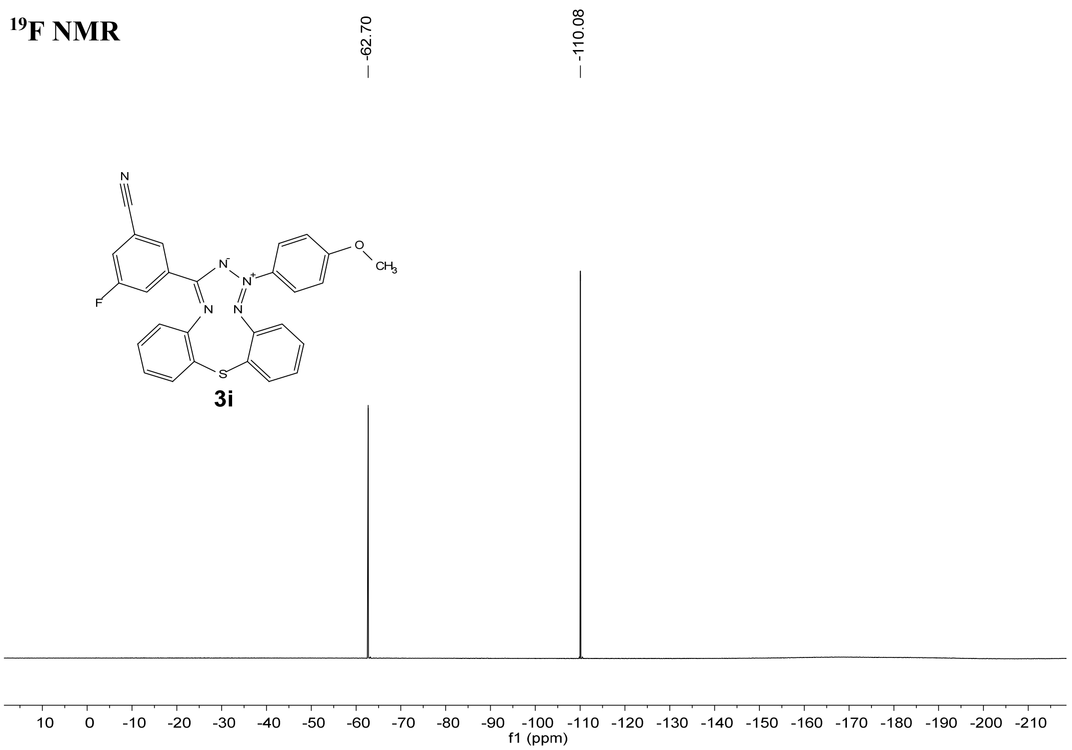

Supplementary Figure 86 ^{19}F NMR spectra of Compound **3i**.

^{1}H NMR

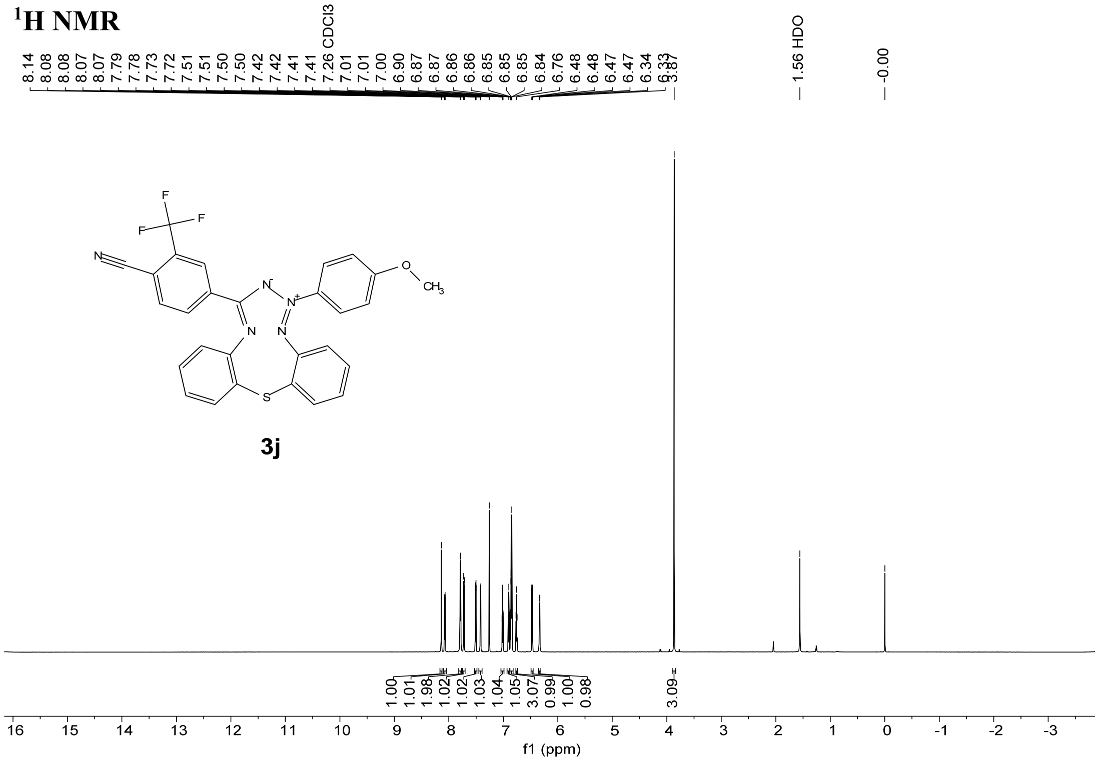

Supplementary Figure 87 ^{1}H NMR spectra of Compound **3j**.

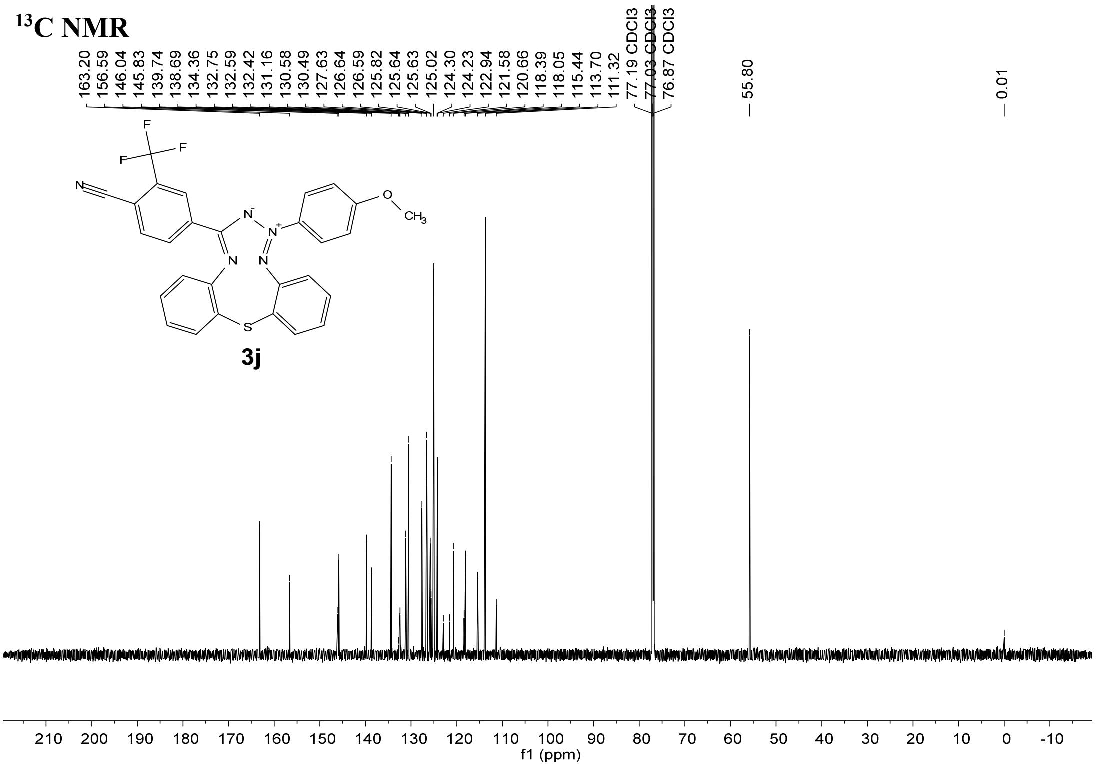

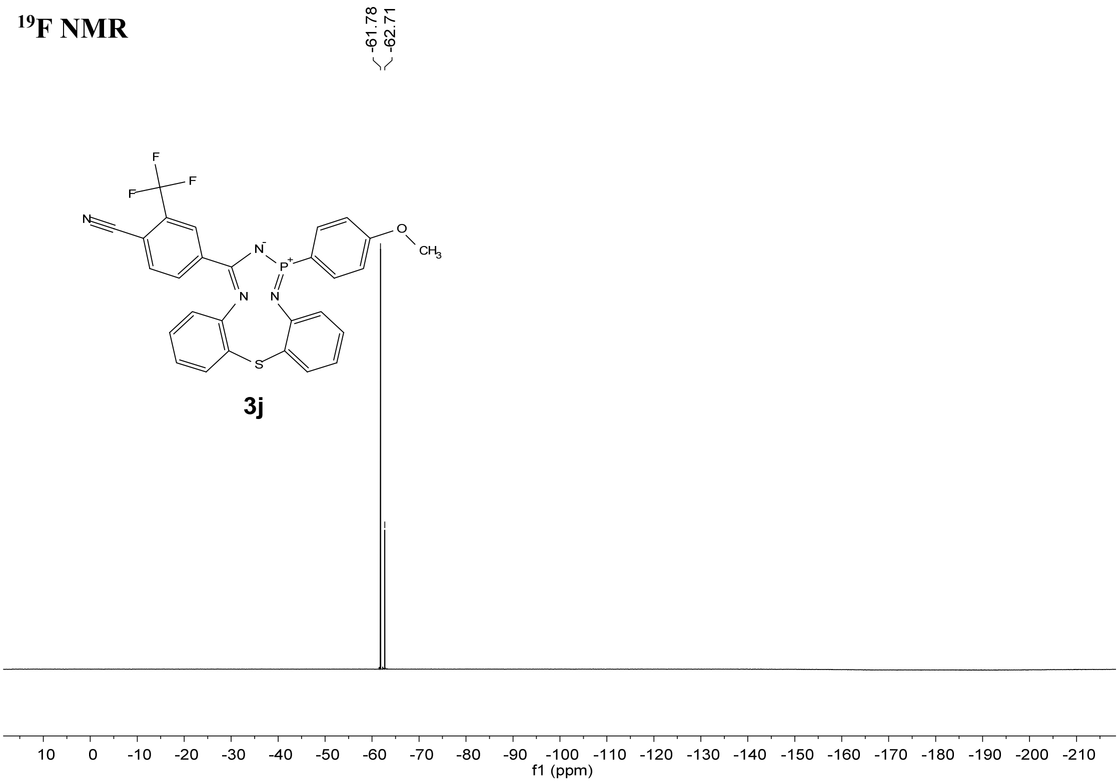

Supplementary Figure 88 ^{13}C NMR and ^{19}F NMR spectra of Compound **3j**.

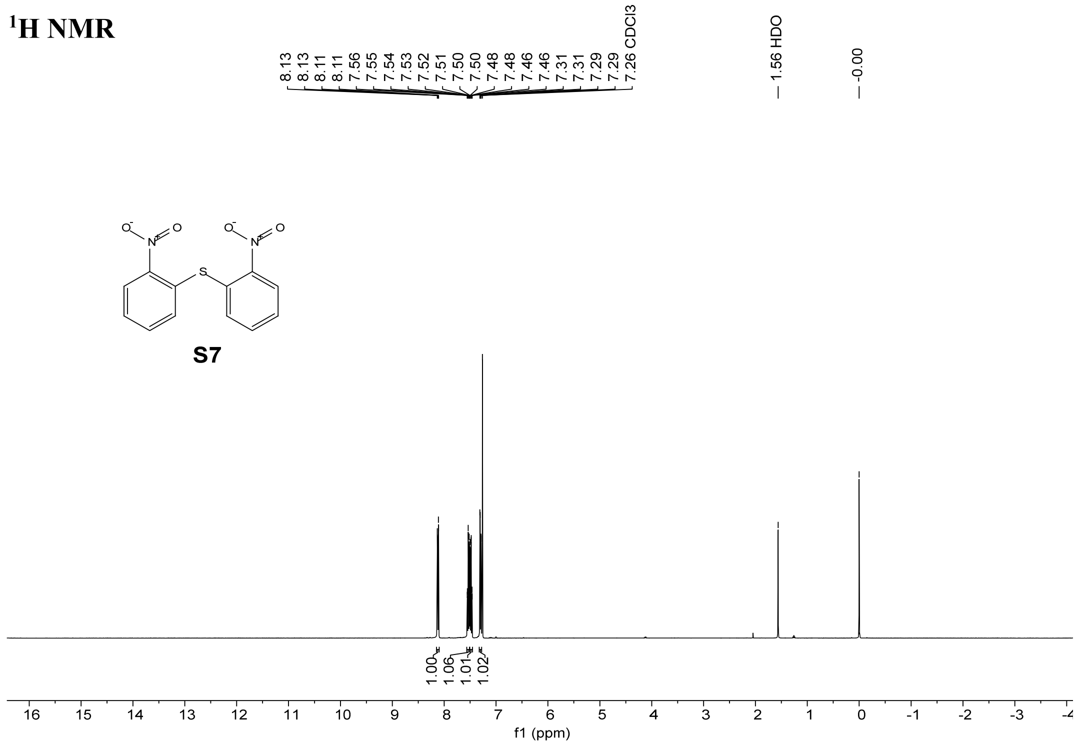

Supplementary Figure 89 ¹H NMR spectra of Compound **S7**.

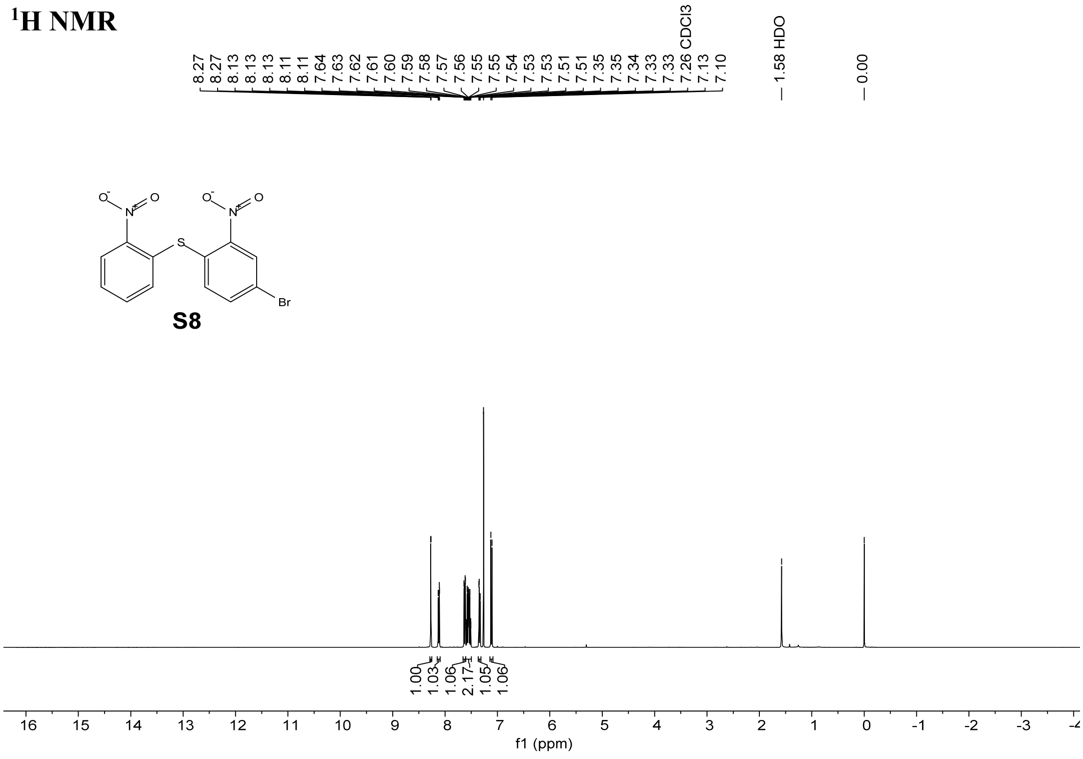

Supplementary Figure 90 ¹H NMR spectra of Compound **S8**.

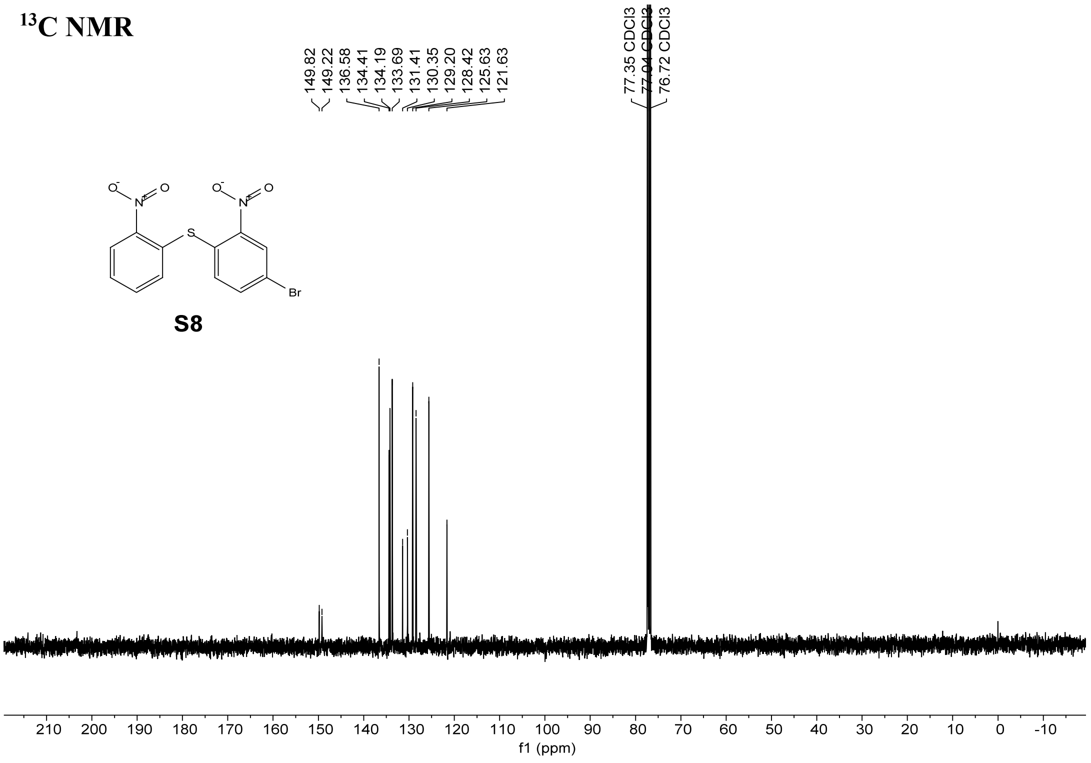

Supplementary Figure 91 ^{13}C NMR spectra of Compound **S8**.

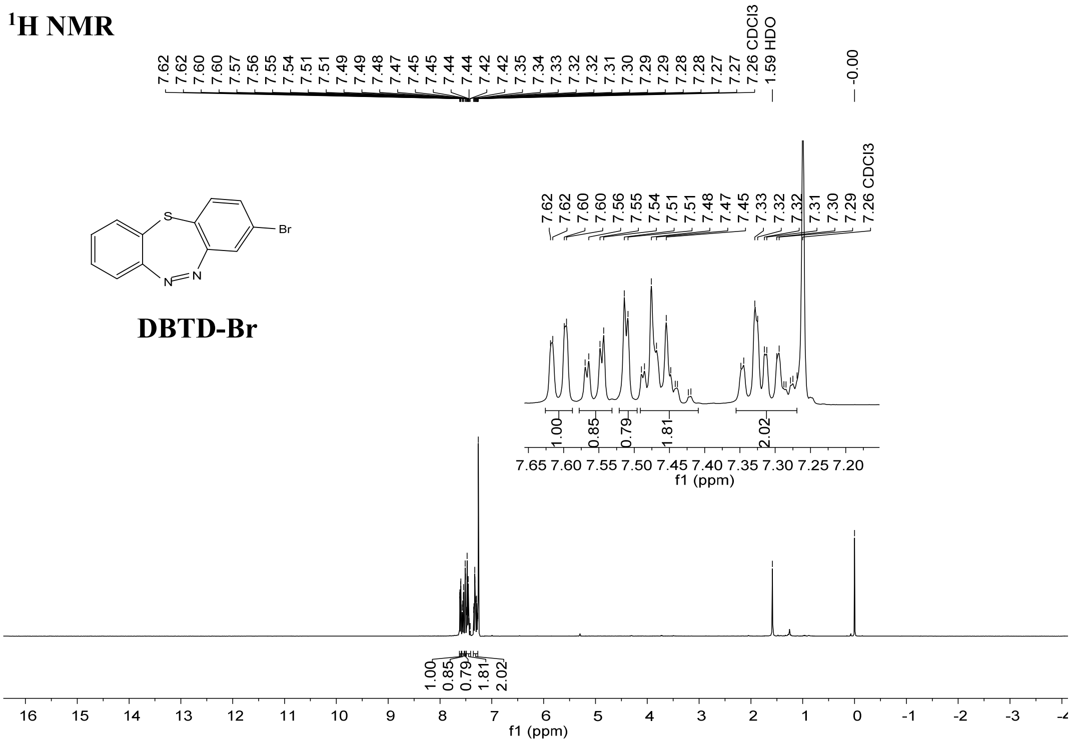

Supplementary Figure 92 ^{1}H NMR spectra of Compound **DBTD-Br**.

^{13}C NMR

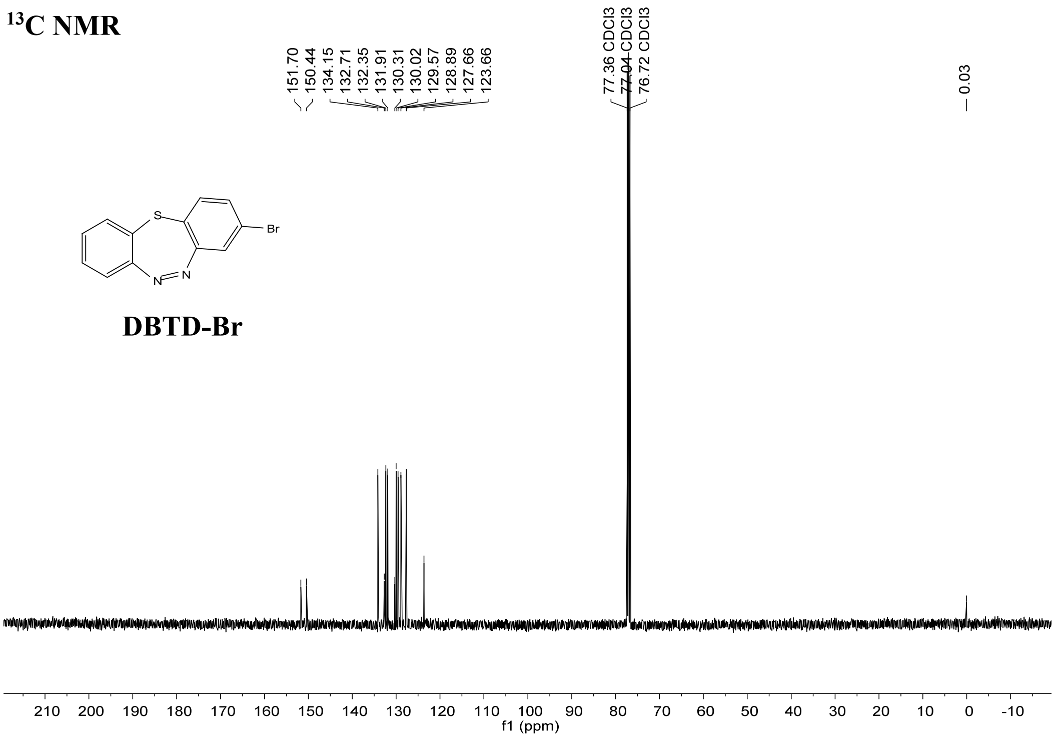

Supplementary Figure 93 ^{13}C NMR spectra of Compound **DBTD-Br**.

^{1}H NMR

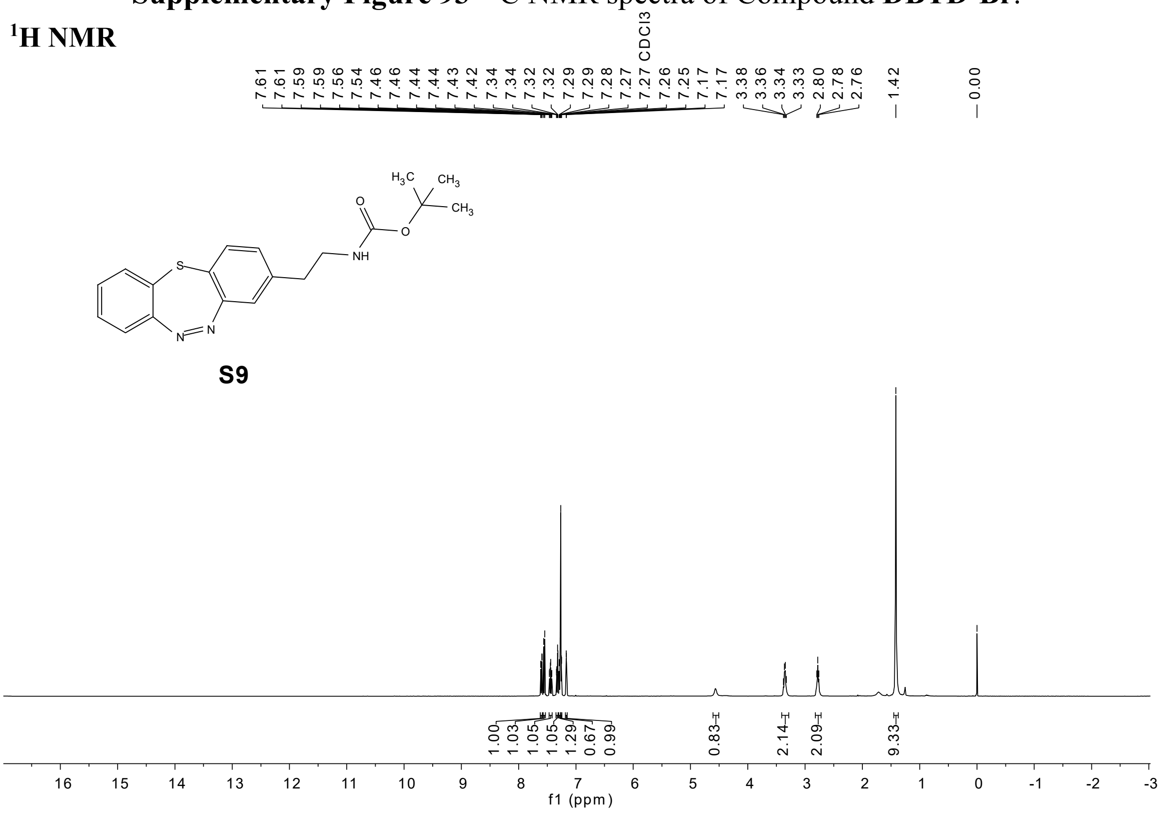

Supplementary Figure 94 ^{1}H NMR spectra of Compound **S9**.

^{13}C NMR

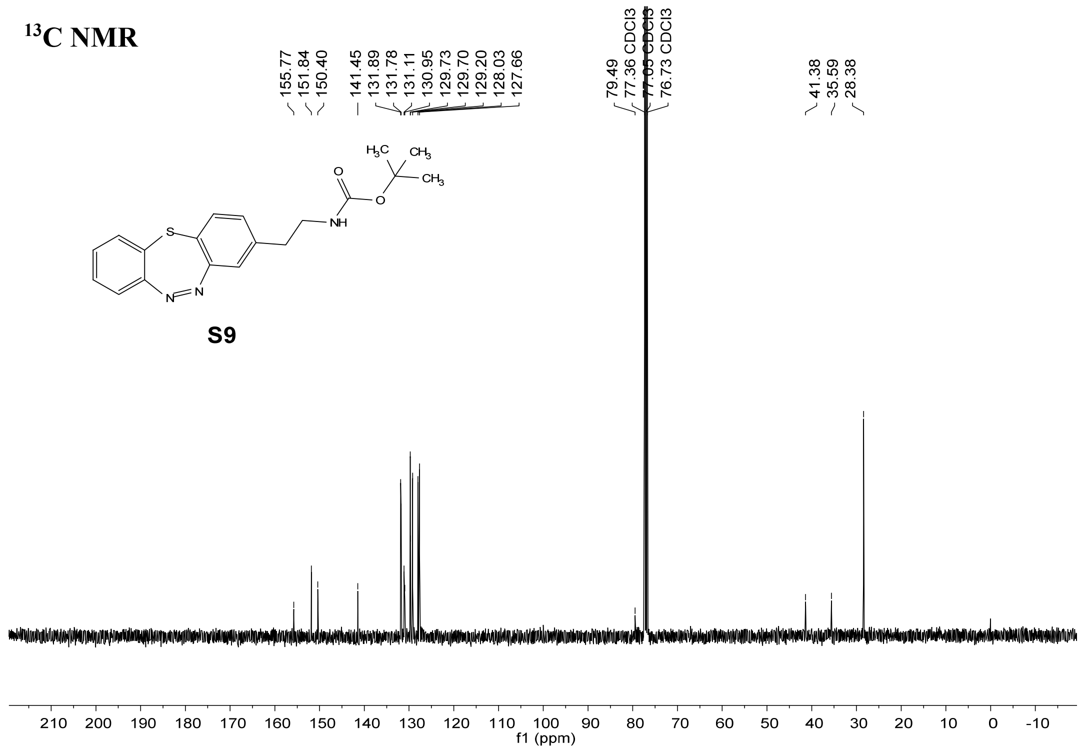

Supplementary Figure 95 ^{13}C NMR spectra of Compound **S9**.

^{1}H NMR

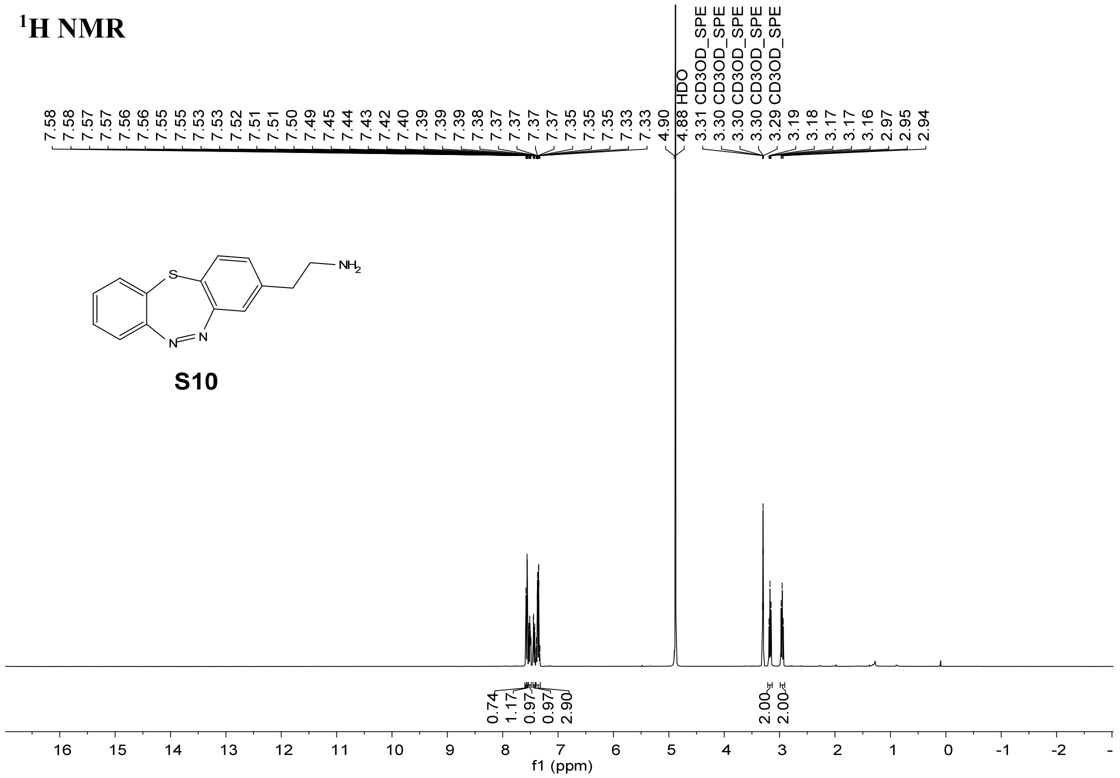

Supplementary Figure 96 ^{1}H NMR spectra of Compound **S10**.

^{13}C NMR

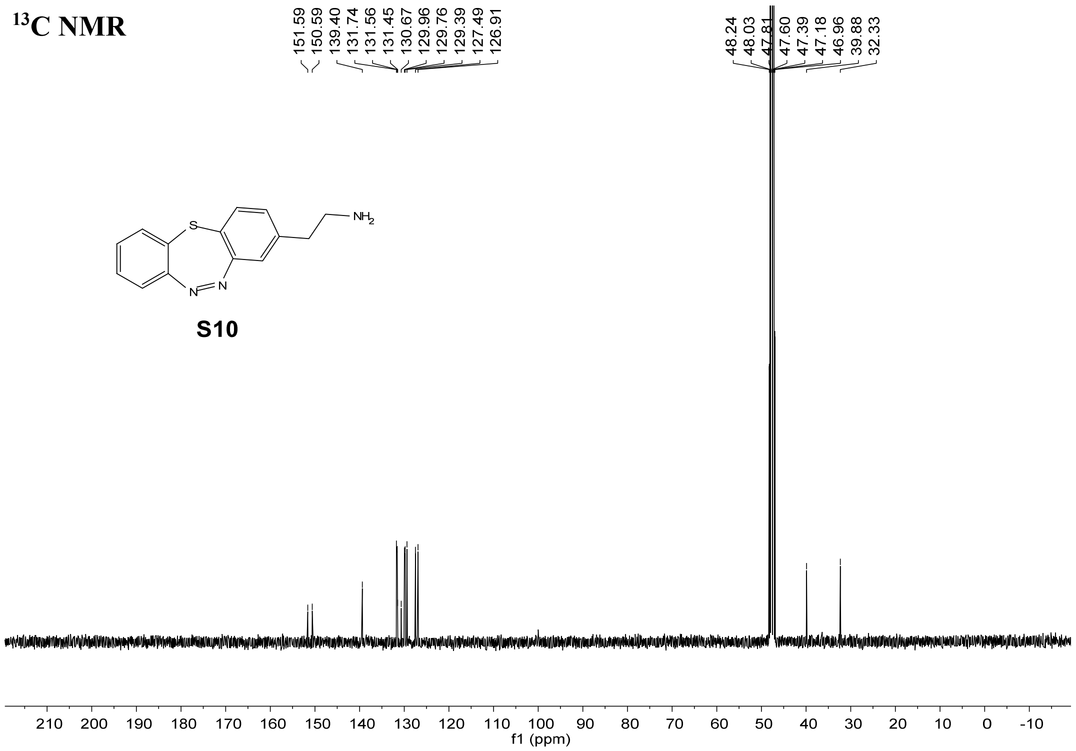

Supplementary Figure 97 ^{13}C NMR spectra of Compound **S10**.

^{1}H NMR

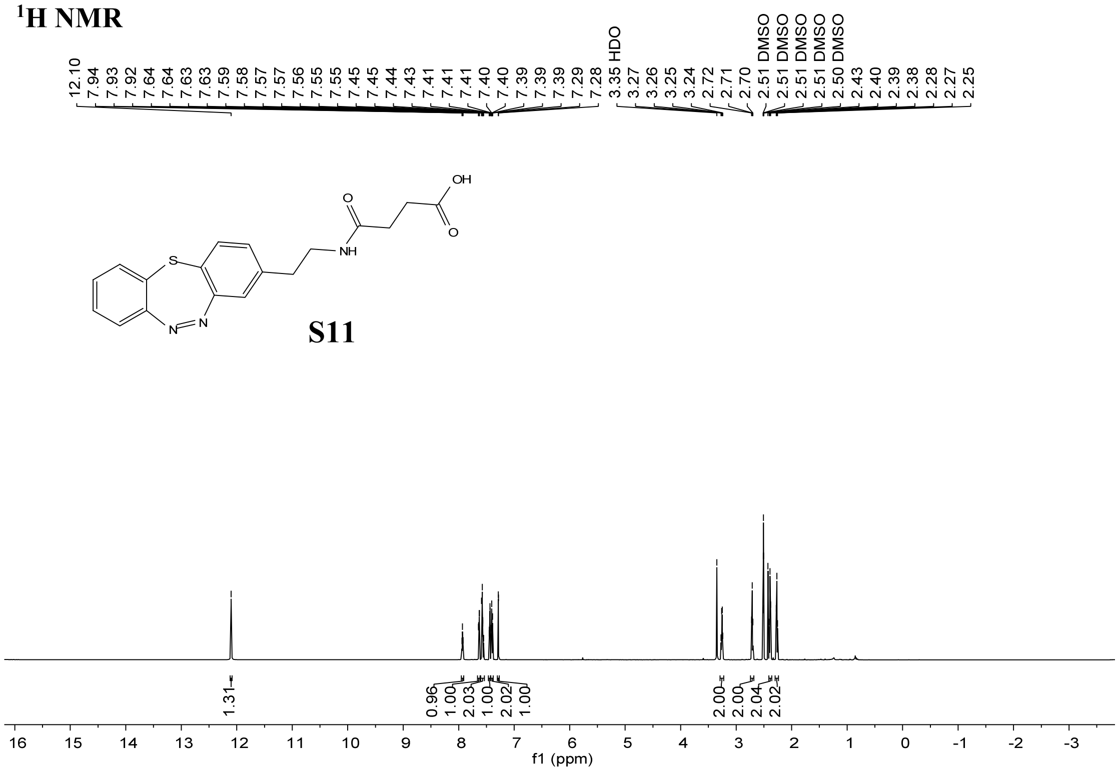

Supplementary Figure 98 ^{1}H NMR spectra of Compound **S11**.

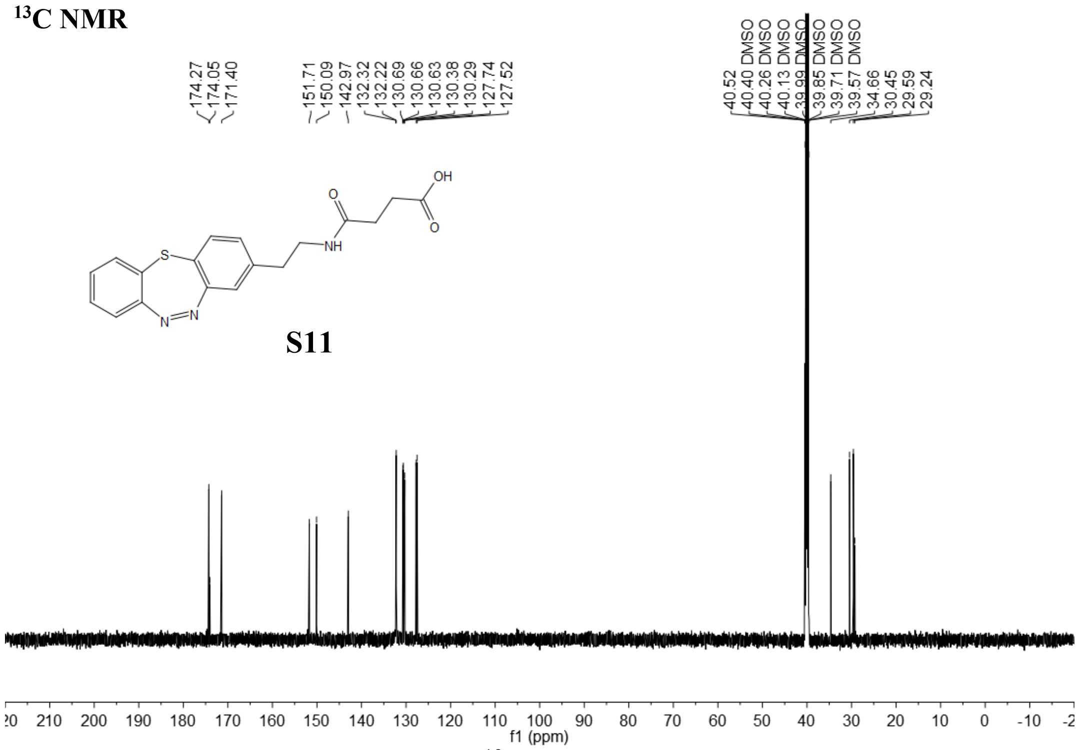

Supplementary Figure 99 ^{13}C NMR spectra of Compound **S11**.

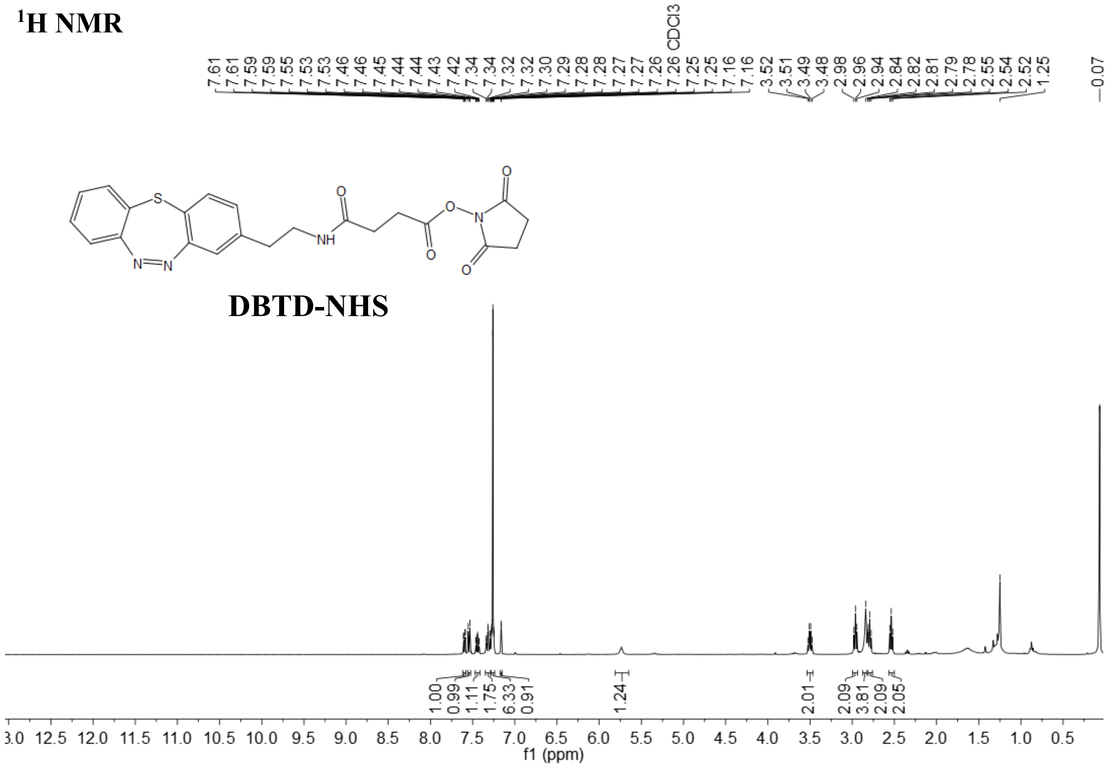

Supplementary Figure 100 ^{1}H NMR spectra of Compound **DBTD-NHS**.

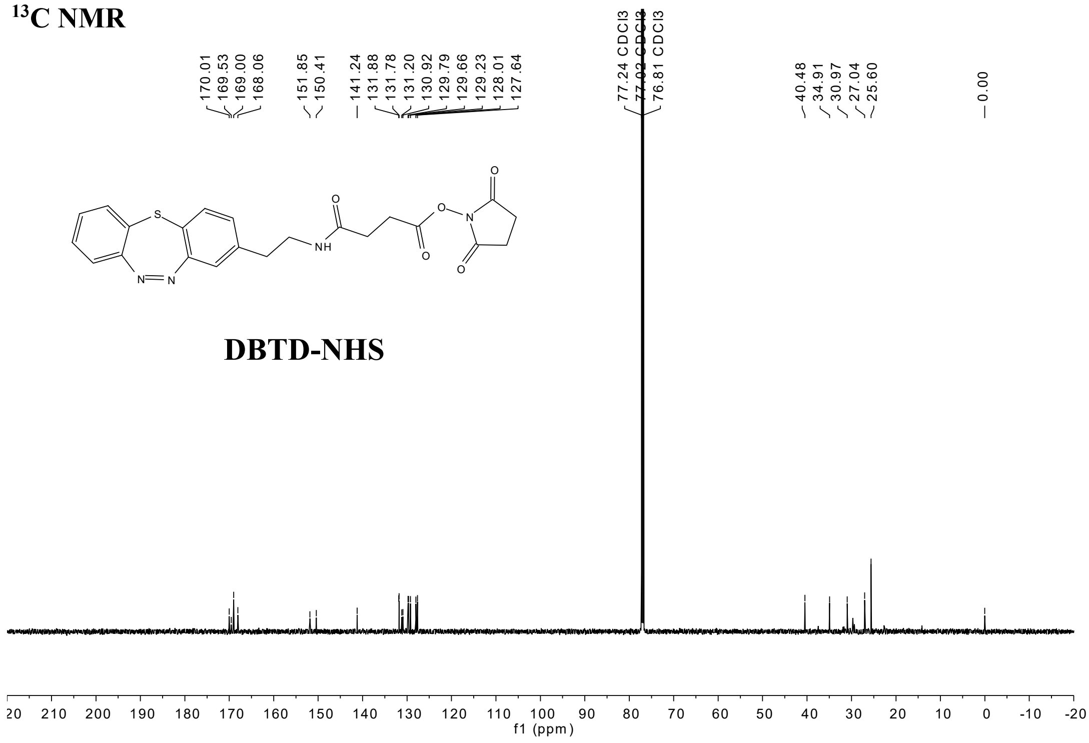

Supplementary Figure 101 ^{13}C NMR spectra of Compound **DBTD-NHS**.

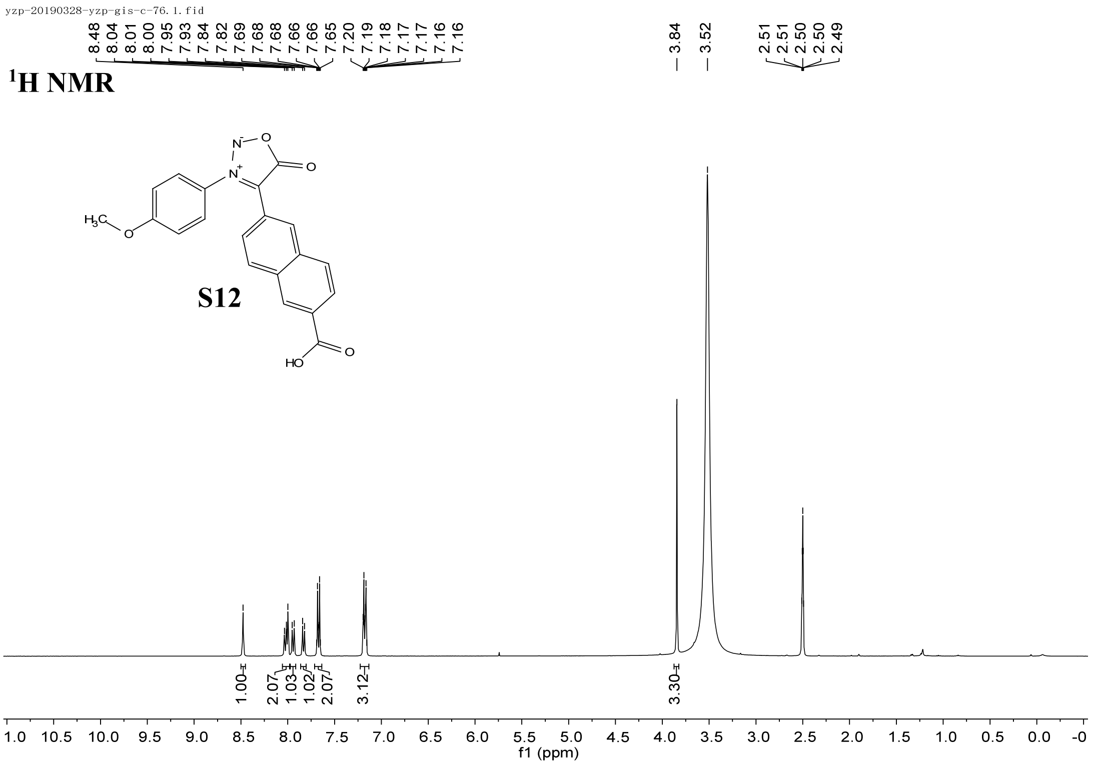

Supplementary Figure 102 ^{1}H NMR spectra of Compound **S12**.

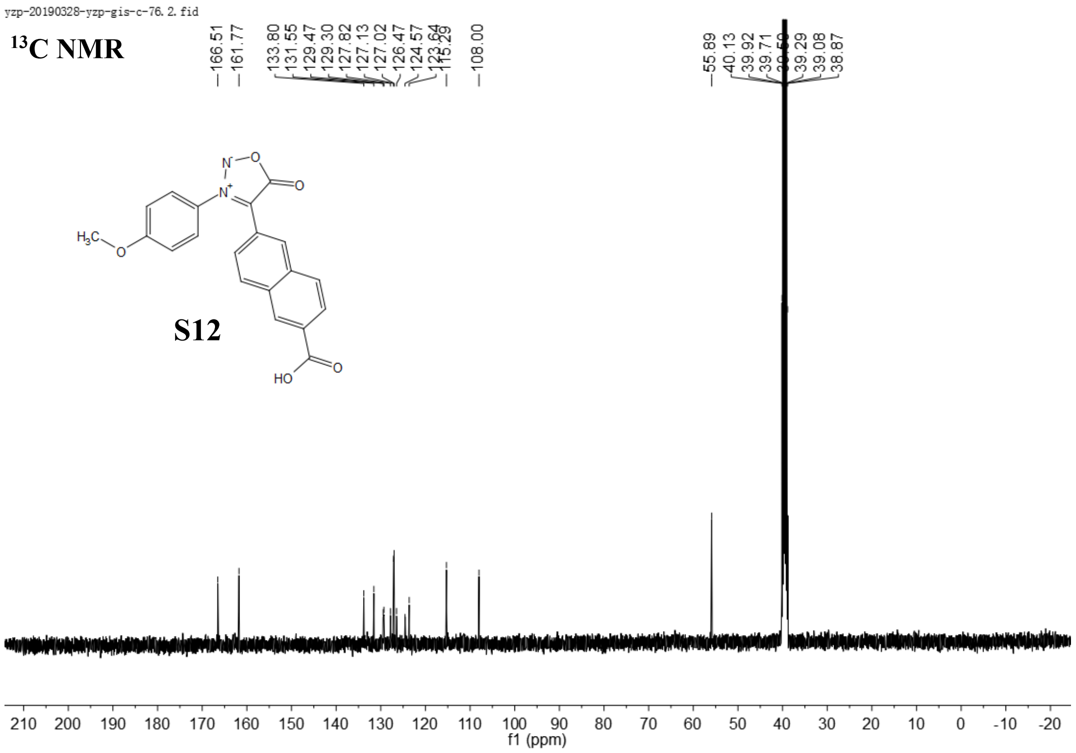

Supplementary Figure 103 ^{13}C NMR spectra of Compound **S12**.

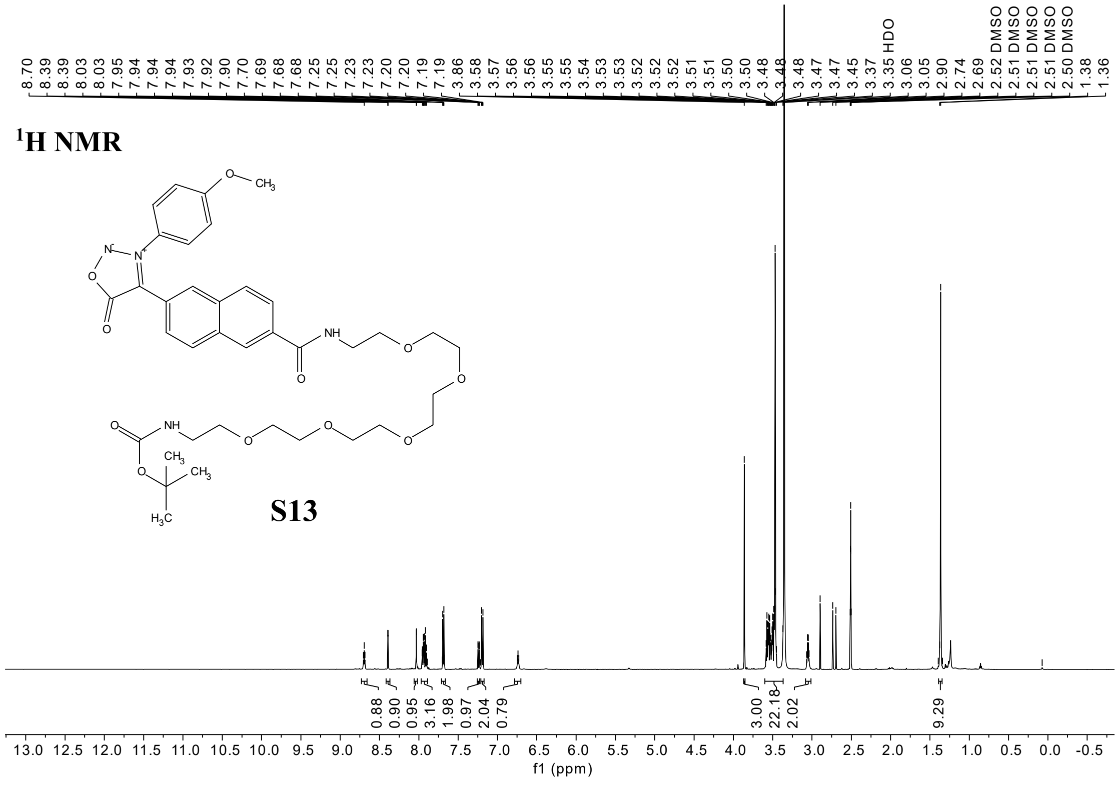

Supplementary Figure 104 ^{1}H NMR spectra of Compound **S13**.

¹³C NMR

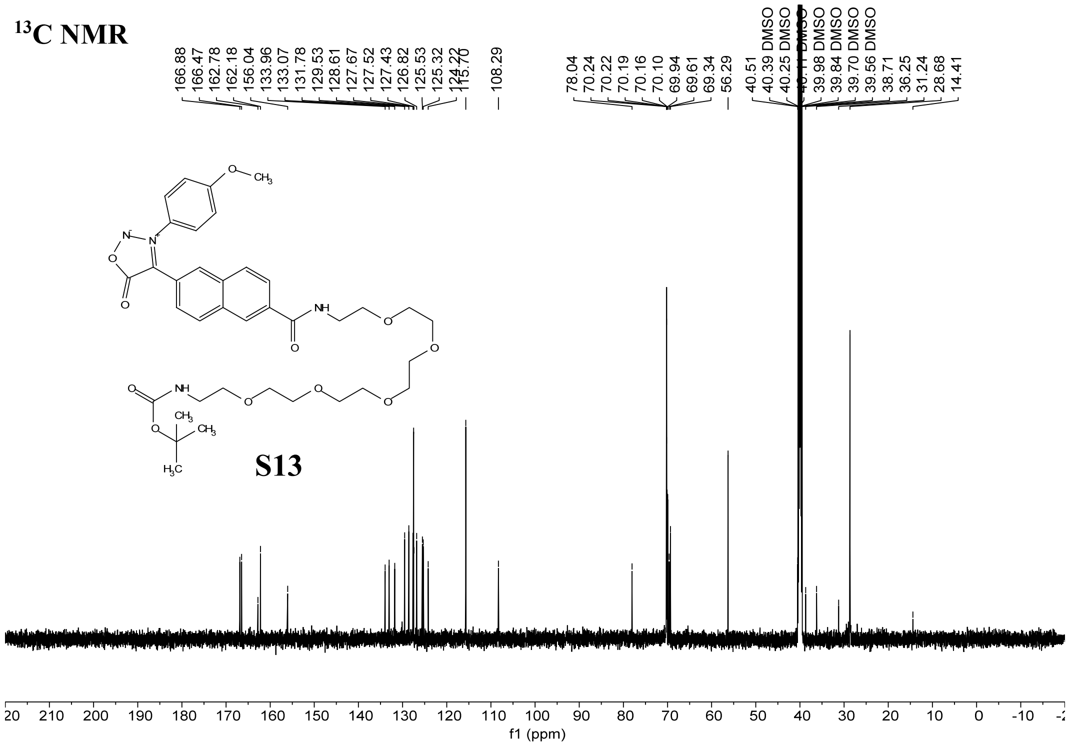

Supplementary Figure 105 ^{13}C NMR spectra of Compound **S13**.

¹H NMR

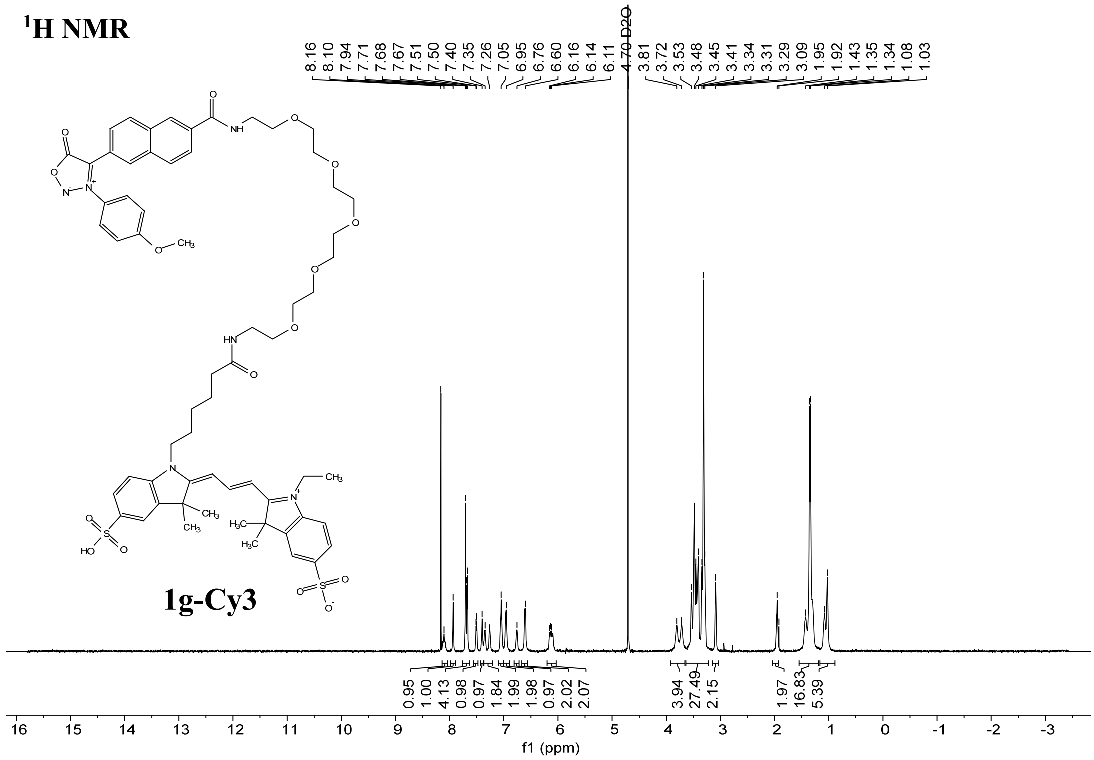

Supplementary Figure 106 ^{1}H NMR spectra of Compound **1g-Cy3**.
