# Supplementary material for: Direct ring-strain loading for visible-light accelerated bioorthogonal ligation via diarylsydnone-dibenzo[b,f ][1,4,5]thiadiazepine photo-click reactions
Source: Commun Chem. 2020 Mar 4;3:29. doi: 10.1038/s42004-020-0273-6 (PMC9814081; doi:10.1038/s42004-020-0273-6)
Supplement: Supplementary file 3 — Description of Additional Supplementary Files [file 42004_2020_273_MOESM3_ESM.pdf]

Description of Additional Supplementary Files

1. File Name: Supplementary Data 1
   Description: Crystallographic information file for compound **3e**.

2. File Name: Supplementary Data 2
   Description: Crystallographic information file for compound (*Z*)-DBTD.

3. File Name: Supplementary Movie 1
   Description: The spatial controlled switching of the DBTD for twice at 213 K in ethanol via an optical fiber guided 405 nm laser could also been observed.
