# Supplementary material for: Direct ring-strain loading for visible-light accelerated bioorthogonal ligation via diarylsydnone-dibenzo[b,f ][1,4,5]thiadiazepine photo-click reactions
Source: Commun Chem. 2020 Mar 4;3:29. doi: 10.1038/s42004-020-0273-6 (PMC9814081; doi:10.1038/s42004-020-0273-6)
Supplement: Supplementary file 4 — Supplementary Data 1 [file 42004_2020_273_MOESM4_ESM.zip › 3e.pdf]

Supplementary Data 1.
Crystallographic information file and structure refinement for **3e**.

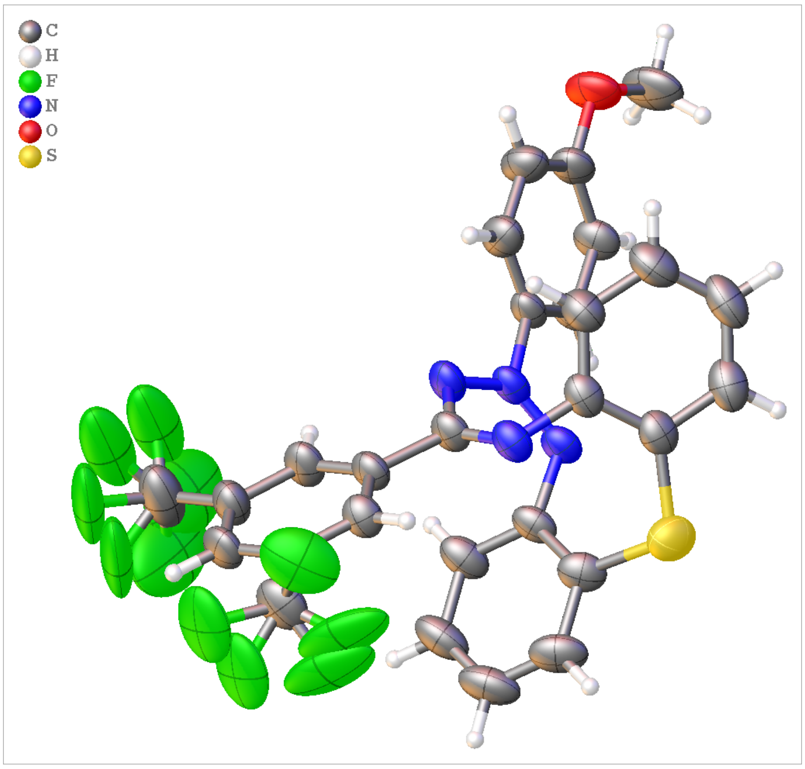

Identification code	180511_s2_gjs	
Empirical formula	$C_{28}H_{18}F_6N_4OS$	
Formula weight	572.52	
Temperature	293.15 K	
Crystal system	monoclinic	
Space group	C2/c	
Unit cell dimensions	*a* = 25.630(2) Å	α = 90 °
	b = 7.8333(5) Å	β = 111.329(10) °
	c = 27.839(2) Å	γ = 90 °
Volume	5206.2(8) $Å^3$	
Z	8	
Density (calculated)	1.461 g cm^{-3}	
Absorption coefficient	0.197 mm^{-1}	
F (000)	2336.0	
Crystal size	0.35 x 0.3 x 0.2 mm^3	

Radiation	MoKα (λ = 0.71073)
Theta range for data collection	5.864 to 52.744°
Index ranges	$-22 \leq h \leq 32$, $-9 \leq k \leq 9$, $-32 \leq l \leq 34$
Reflections collected	11181
Independent reflections	5317 [Rint = 0.0304, Rsigma = 0.0531]
Data / restraints / parameters	5317 / 6 / 380
Goodness-of-fit on F^2	1.018
Final R indices [I>2 sigma (I)]	R_1 = 0.0696, wR_2 = 0.1679
Final R indices (all data)	R_1 = 0.0865, wR_2 = 0.1976
Largest diff. peak and hole	0.37 and -0.36 e. Å^{-3}

Single crystal of **3e** [$C_{28}H_{18}F_6N_4OS$] was obtained by recrystallization in hexane/EtOAc. CCDC-1905280 (CIF) contains the supplementary crystallographic data which can be obtained free of charge from Cambrige Crystallographic Data Centere *via* www.ccdc.cam.ac.uk/data_request/cif.
