# Supplementary material for: Direct ring-strain loading for visible-light accelerated bioorthogonal ligation via diarylsydnone-dibenzo[b,f ][1,4,5]thiadiazepine photo-click reactions
Source: Commun Chem. 2020 Mar 4;3:29. doi: 10.1038/s42004-020-0273-6 (PMC9814081; doi:10.1038/s42004-020-0273-6)
Supplement: Supplementary file 5 — Supplementary Data 2 [file 42004_2020_273_MOESM5_ESM.zip › DBTD.pdf]

**Supplementary Data 2.**

Crystallographic information file and structure refinement for (*Z*)-DBTD.

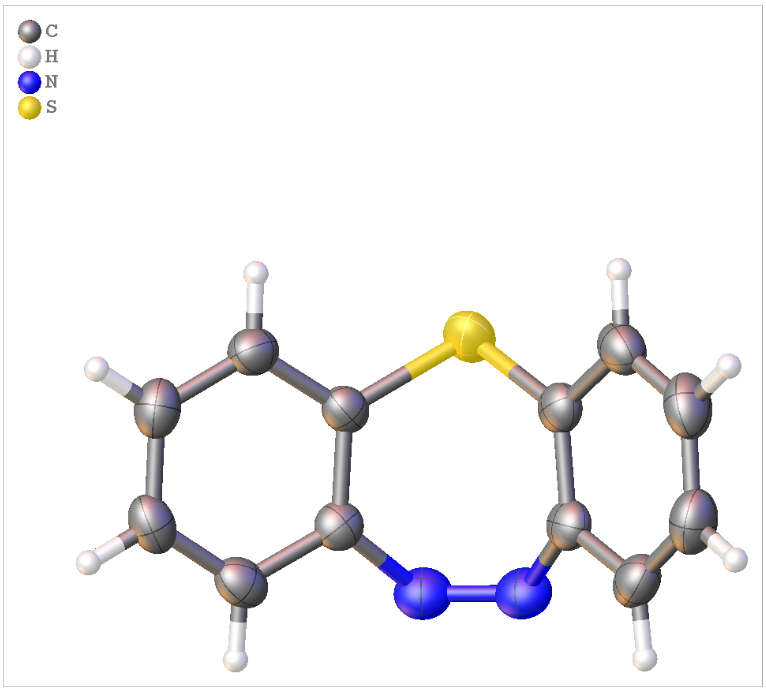


| | | |
|---|---|---|
| Identification code | 180917_s3_gjs | |
| Empirical formula | $C_{12}H_8N_2S$ | |
| Formula weight | 212.26 | |
| Temperature | 293.15 K | |
| Crystal system | monoclinic | |
| Space group | $P2_1/n$ | |
| Unit cell dimensions | $a$ = 5.8600(5) Å | α = 90 ° |
| | $b$ = 12.4535(12) Å | β = 91.561(7) ° |
| | $c$ = 13.6689(10) Å | γ = 90 ° |
| Volume | 997.16(14) $Å^3$ | |
| $Z$ | 4 | |
| Density (calculated) | 1.414 g $cm^{-3}$ | |
| Absorption coefficient | 0.286 $mm^{-1}$ | |
| $F$ (000) | 440.0 | |
| Crystal size | 0.4 x 0.4 x 0.35 $mm^3$ | |
| Radiation | MoKα (λ = 0.71073) | |

| | |
|---|---|
| 2Θ range for data collection | 5.964 to 58.738° |
| Index ranges | $-8 \le h \le 5$, $-15 \le k \le 10$, $-11 \le l \le 17$ |
| Reflections collected | 4596 |
| Independent reflections | 2293 [Rint = 0.0178, Rsigma = 0.0343] |
| Data / restraints / parameters | 2293/0/136 |
| Goodness-of-fit on $F^2$ | 1.033 |
| Final $R$ indices [$I$>2 sigma ($I$)] | $R_1 = 0.0409$, $wR_2 = 0.0898$ |
| Final $R$ indices (all data) | $R_1 = 0.0611$, $wR_2 = 0.1028$ |
| Largest diff. peak and hole | 0.17 and -0.29 e. Å$^{-3}$ |

Single crystal of (*Z*)-DBTD [$C_{12}H_8N_2S$] was obtained by recrystallization in hexane/EtOAc. CCDC-1905279 (CIF) contains the supplementary crystallographic data which can be obtained free of charge from Cambrige Crystallographic Data Centere *via* www.ccdc.cam.ac.uk/data_request/cif.
